# Elucidating tumour‐associated microglia/macrophage diversity along glioblastoma progression and under *ACOD1* deficiency

**DOI:** 10.1002/1878-0261.13287

**Published:** 2022-08-15

**Authors:** Yolanda Pires‐Afonso, Arnaud Muller, Kamil Grzyb, Anaïs Oudin, Yahaya A. Yabo, Carole Sousa, Andrea Scafidi, Aurélie Poli, Antonio Cosma, Rashi Halder, Djalil Coowar, Anna Golebiewska, Alexander Skupin, Simone P. Niclou, Alessandro Michelucci

**Affiliations:** ^1^ Neuro‐Immunology Group, Department of Cancer Research Luxembourg Institute of Health Luxembourg; ^2^ Doctoral School of Science and Technology University of Luxembourg Esch‐sur‐Alzette Luxembourg; ^3^ Quantitative Biology Unit, Bioinformatics Platform Luxembourg Institute of Health Luxembourg; ^4^ Luxembourg Centre for Systems Biomedicine University of Luxembourg Esch‐Belval Luxembourg; ^5^ NORLUX Neuro‐Oncology Laboratory, Department of Cancer Research Luxembourg Institute of Health Luxembourg; ^6^ Quantitative Biology Unit, National Cytometry Platform Luxembourg Institute of Health Luxembourg; ^7^ National Centre for Microscopy and Imaging Research University of California San Diego La Jolla CA USA; ^8^ KG Jebsen Brain Tumour Research Center, Department of Biomedicine University of Bergen Norway

**Keywords:** ACOD1/IRG1, glioblastoma, heterogeneity, metabolic reprogramming, single‐cell RNA‐sequencing, tumour‐associated microglia/macrophages

## Abstract

In glioblastoma (GBM), tumour‐associated microglia/macrophages (TAMs) represent the major cell type of the stromal compartment and contribute to tumour immune escape mechanisms. Thus, targeting TAMs is emerging as a promising strategy for immunotherapy. However, TAM heterogeneity and metabolic adaptation along GBM progression represent critical features for the design of effective TAM‐targeted therapies. Here, we comprehensively study the cellular and molecular changes of TAMs in the GL261 GBM mouse model, combining single‐cell RNA‐sequencing with flow cytometry and immunohistological analyses along GBM progression and in the absence of *Acod1* (also known as *Irg1*), a key gene involved in the metabolic reprogramming of macrophages towards an anti‐inflammatory phenotype. Similarly to patients, we identify distinct TAM profiles, mainly based on their ontogeny, that reiterate the idea that microglia‐ and macrophage‐like cells show key transcriptional differences and dynamically adapt along GBM stages. Notably, we uncover decreased antigen‐presenting cell features and immune reactivity in TAMs along tumour progression that are instead enhanced in *Acod1‐*deficient mice. Overall, our results provide insight into TAM heterogeneity and highlight a novel role for *Acod1* in TAM adaptation during GBM progression.

## Graphical abstract

By studying the education of tumour‐associated microglia/macrophages (TAMs) in the glioblastoma mouse model GL261 combining single‐cell RNA‐sequencing with immune‐profiling analyses, we show that resident microglia and monocytic‐derived macrophages within the tumour display key transcriptional differences and dynamically adapt along GBM stages. Specifically, TAMs exhibit decreased immune reactivity along tumour progression that is instead enhanced in *Acod1‐*deficient mice.

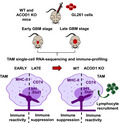

**Abbreviations**
ACOD1: aconitate decarboxylase 1
BMDM: bone marrow‐derived macrophage
Cd36: CD36 molecule
Cd74: CD74 molecule
CD74: HLA class II histocompatibility antigen gamma chain
Clec7a: C‐type lectin domain containing 7A
CNS: central nervous system
Cxcl9: C‐X‐C motif chemokine ligand 9
DAVID: database for annotation, visualization and integrated discovery
GBM: glioblastoma
GO: gene ontology
H2‐Aa: major histocompatibility complex, class II
H2‐Ab1: major histocompatibility complex, class II
H2‐K1: major histocompatibility complex, class I, A
H2‐T23: major histocompatibility complex, class I, E
IBA1: allograft inflammatory factor 1
IRF1: interferon Regulatory Factor 1
IRG1: immunoresponsive gene 1
KO: knock‐out
LGG: low grade glioma
MHC‐II: major histocompatibility complex class II molecules
MRI: magnetic resonance imaging
MvP: microvascular proliferation
OPC: oligodendrocyte precursor cell
PCAN: pseudopalisading cells around necrosis
PDOX: patient‐derived orthotopic xenograft
ScRNA‐seq: Single‐cell RNA sequencing
SEM: standard error of the mean
STAT1: signal transducer and activator of transcription 1
TAM: tumour‐associated microglia/macrophage
TCGA: The Cancer Genome Atlas
TME: tumour microenvironment
UMAP: uniform manifold approximation and projection
WT: wild‐type

## 1 Introduction

Complex interactions between neoplastic cells and their microenvironment sustain cancer heterogeneity and evolution [1, 2]. In the brain, tumours develop within a network of resident central nervous system (CNS) cells, including neurons, astrocytes, oligodendrocytes, endothelial cells and microglia, together with peripheral infiltrating immune components. These cells, together with the extracellular matrix, constitute the tumour microenvironment (TME), which drives disease progression by affecting tumour growth, patient survival and response to therapy. In glioblastoma (GBM), the most aggressive brain tumour in adults, the TME is mainly composed of tumour‐associated microglia/macrophages (TAMs), which can represent up to 40% of the tumour mass, creating a supportive milieu that facilitates tumour proliferation, survival and migration [3]. TAMs are either resident parenchymal microglia, whose progenitors migrated to the CNS during early development [4, 5] or peripheral monocyte‐derived cells that have crossed the blood–brain barrier [6]. Once in the CNS, the latter differentiate into tumour‐associated macrophages becoming nearly indistinguishable from activated resident microglia [7]. Thus, how ontogeny contributes to TAM education has only been started to be described in GBM transgenic mouse models [8] or in patients [9, 10] as a result of recently discovered specific markers.

Glioblastoma recruits TAMs, which in turn release growth factors and cytokines that affect the tumour. TAMs display specific immune properties that are different from classical pro‐inflammatory activated (immune‐permissive) M1 or alternatively activated (immune‐suppressive) M2 reactive profiles [11, 12] or even exhibit nonpolarized M0 features [13]. The complex interplay between pro‐ and anti‐tumour processes depending on the molecular signals within the TME, both within and across cell types, contributes to the difficulty in interpreting tissue‐resolution bulk signatures of GBM. In this context, single‐cell RNA‐sequencing (scRNA‐seq) provides a remarkable method to depict heterogeneous cell populations and measure cell‐to‐cell expression variability of thousands of genes [14, 15, 16, 17]. Specifically, in GBM patients, scRNA‐seq has emerged as a critical tool to discriminate TAM heterogeneity and their contribution to distinct glioma subtypes [9, 18]. Notably, scRNA‐seq analyses enabled to discover that TAMs frequently co‐express canonical M1 and M2 genes in individual cells [10].

Here, we combine scRNA‐seq analyses with flow cytometry and immunofluorescence studies to elucidate the cellular and molecular properties of the TME, with a specific focus on TAMs. Following the discrimination of microglia from monocyte‐derived macrophages and the characterization of their transcriptional programmes along tumour progression, we assess the role of aconitate decarboxylase 1/immunoresponsive gene 1 (*Acod1/Irg1*) in TAM polarization. The ACOD1/IRG1 enzyme catalyses the production of the anti‐microbial immunometabolite itaconate from *cis*‐aconitate in the tricarboxylic acid (TCA) cycle [19]. In macrophages, the induction of itaconate under inflammatory conditions reprograms them into a more pronounced anti‐inflammatory phenotype, participating to the resolution of inflammation [20, 21]. Notably, the induction of the ACOD1/IRG1‐itaconate axis in monocytes contributes to the immune paralysis in sepsis [22], while its inhibition in macrophages reduces the tumour burden in peritoneal tumours [23]. Here, we identify discrete TAM profiles, which reiterate microglia‐ versus macrophage‐like features showing key transcriptional differences and dynamically adapting along GBM stages. Notably, we demonstrate that TAMs display decreased antigen‐presenting cell features and immune reactivity along tumour progression, which are enhanced in *Acod1/Irg1‐*deficient mice.

The understanding of TAM diversity, and more systematically of TME heterogeneity, which significantly contributes to GBM growth, is of utmost relevance for the discovery of novel immunotherapeutic opportunities [24]. Hence, our results point to important aspects to take into consideration when targeting TAMs and highlight a novel role for *Acod1/Irg1* in TAM adaptation during GBM progression.

## 2 Materials and methods

### 2.1 Animals

*Acod1 KO* mice were generated by Dr. Haruhiko Koseki at the RIKEN Institute using embryonic stem cells purchased from the Knockout Mouse Project Repository (KOMP, University of California, DAVIS) under strain ID Irg1^tm1a(KOMP)Wtsi^ containing an insertion cassette between exons 3 and 5. Briefly, *Acod1 KO* C57BL/6N ESCs were injected into recipient female C57BL/6N mouse blastocysts and selected females were subsequently bred with wild‐type C57BL/6N mice [25]. For the experiments, heterozygote animals were crossed to generate homozygote *Acod1 KO* mice and *WT* C57BL/6N littermate controls. We confirmed their genotype by PCR and we used a mix of male and female littermates for experiments. Mice were housed in 12 h light/dark cycle and had free access to sterile food and water. All animal procedures were approved by the national authorities and the animal welfare structure of LIH under the reference LUPA 2017/20. The animal work of this study has been conducted and reported in accordance to the ARRIVE (Animal Research: Reporting of *In Vivo* Experiments) guidelines to improve the design, analysis and reporting of research using animals, maximizing information published and minimizing unnecessary studies.

### 2.2 Glioma cell line

Mouse glioma 261 (GL261) cells were kindly provided by Dr. Poli (Neuro‐Immunology Group, Luxembourg Institute of Health) and were maintained at 37 °C with 5% CO_2_ in culture medium [Dulbecco's Modified Eagle's Medium (DMEM; Gibco/Life Technologies, Waltham, MA, USA)] supplemented with 10% Fetal Bovine Serum (FBS; Gibco/Life Technologies) and pen‐strep (100 U·mL^−1^; Gibco/Life Technologies, Waltham, MA, USA). Cells at 80% confluence were dissociated with 0.05% Trypsin–EDTA (Gibco/Life Technologies) and tested for mycoplasma (MycoAlert PLUS Mycoplasma Detection Kit, Westburg, The Netherlands) before mice implantation. For mice orthotopic implantation, GL261 cells were re‐suspended in serum‐free medium.

### 2.3 Differentiation of murine bone marrow‐derived macrophages and co‐culture experiments with GL261 cells

Bone‐marrow cells were obtained by flushing the tibia and femurs of WT and *Acod1* KO adult mice. Briefly, mice were euthanized and their legs were removed. Bone marrow precursors were flushed out and cell suspension was further incubated with red blood cells hypotonic lysis buffer. After washing, cells were plated in DMEM media containing 10% FBS supplemented with 20% of L929 supernatant for 7 days for full differentiation of bone marrow‐derived macrophages (BMDMs).

GL261 and BMDMs were co‐cultured in 1 : 1 mix in DMEM medium containing 10% FBS. GL261 cells were plated on top of 1 μm pore size Boyden chambers (Thincert, Greiner, Kremsmünster, Austria), whereas BMDMs were plated on the bottom of the 6‐well plates. The mRNA was isolated from BMDMs at 0, 24 and 48 h using the RNeasy mini kit according to the manufacturer’ instructions (Qiagen, Germantown, MD, USA).

### 2.4 GL261 orthotopic implantation and tumour volume measurement

Before the implantation, mice were intraperitoneally anesthetized with a mixture of ketamine (100 mg·kg^−1^) and xylazine (10 mg·kg^−1^) and placed in a stereotactic frame. A local anaesthetic was administered subcutaneously (Marcain 0.25% with Adrenalin) and 1 μL containing 500 GL261 cells were implanted into the frontal cortex of the brain using a Hamilton syringe (Hamilton, Reno, NV, USA). Mice were monitored weekly for the first 2 weeks and daily from day 15 postimplantation. Magnetic resonance imaging (MRI) was performed weekly upon 15 postimplantation to assess tumour volume, using a 3T preclinical horizontal bore scanner (MR Solutions, Guilford, UK), equipped with a quadrature volume coil designed for mouse head imaging. Animals were placed prone in the cradle and maintained asleep during the duration of the scans, using 2–3% isoflurane mixed with oxygen. The body temperature was kept constant at 37 °C and breathing was monitored throughout the scan sessions. Anatomical series were used to screen the animals and calculate tumour volumes. The Fast Spin Echo T2‐weighted MRI sequence was acquired, with the following acquisition parameters: TE: 68 ms, TR: 3000 ms, echo train: 8, averages: 4, plane resolution: 256 μm, slice thickness: 1 mm, slices: 15, orientation: coronal. Tumour volume was measured on ImageJ software (NIH, Bethesda, MD, USA) as the sum of area obtained by delineating the tumour in each slice and multiplying by slice thickness. Tumour volume quantification was normalized to the initial tumour take.

### 2.5 GBM patient‐derived orthotopic xenograft (PDOX) mouse model

Human glioma biopsy from Patient 13 diagnosed as grade IV GBM IDH wild type was obtained from the Haukeland University Hospital (Bergen, Norway) and used for the generation of patient‐derived orthotopic xenografts upon approval of the local ethics committees (Haukeland University Hospital, Bergen, and Luxembourg National Research Ethics Committee, CNER). The study methodologies conformed to the standards set by the Declaration of Helsinki and the experiments were undertaken with the understanding and written consent of the subject.

3D organoids were prepared as previously described [26, 27]. Fresh human biopsy was mechanically minced and seeded on agar‐coated flasks (0.85%) allowing the formation of spheroids until up to 2 weeks at 37 °C under 5% CO_2_ and atmospheric oxygen in DMEM medium, 10% FBS, 2 mm of L‐Glutamine, 0.4 mm of NEAA and 100 U·mL^−1^ Pen–Strep (all from Lonza). Re‐suspended in serum‐free medium, viable organoids of approximately 300–1000 μm size were collected and used for *in vivo* implantation (6 organoids per mouse) in the right frontal cortex of immunodeficient Nu/Nu Nude mice (Charles River Laboratories, Saint Germain Nuelles, France). Animals were maintained under specific‐pathogen‐free conditions and sacrificed at the appearance of neurological or behavioural abnormalities and weight loss. MRI was performed weekly upon tumour implantation, as described above.

### 2.6 Brain tissue processing and dissociation

Animals were intraperitoneally anaesthetized with a mixture of ketamine (100 mg·kg^−1^) with medetomidine (0.5 mg·kg^−1^) and buprenorphine (0.05 mg·kg^−1^) before intracardiac perfusion with ice‐cold phosphate‐buffered saline (PBS). Brain samples were isolated and processed according to the different applications. For immunofluorescence staining, brains were fixed in 4% PFA for 48 h at room temperature, immersed in 30% sucrose (dissolved in PBS) for 48 h at 4 °C, embedded in optimal cutting temperature (OCT, Tissue‐Tek) solution, sectioned (12 μm), slide mounted and stored at −20 °C. For *ex vivo* studies, naïve brains and tumour‐bearing brains (demarcated taking the tumour core region based on MRI scan) were dissociated using the Neural Dissociation Kit P (MACS Miltenyi Biotec, Bergisch Gladbach, Germany) accordingly to the manufacturer's instructions. Briefly, the cell pellet was re‐suspended in prewarmed EM1 solution (50 μL of Enzyme P, 1900 μL of Buffer X and 2.5 μL of 2‐mercaptoethanol) and incubated for 15 min at 37 °C by reverting tube every 5 min. Next, freshly prepared EM2 solution (20 μL of Buffer Y and 10 μL of Enzyme A) was added to the cell pellet and tissue was mechanically dissociated using glass pipettes and incubated for 10 min at 37 °C to yield a single‐cell suspension. The resultant single‐cell suspension was filtered through a 50 μm and centrifuged at 300 ***g***, 4 °C for 10 min. Next, we removed the myelin from the single cell suspension using the myelin removal beads kit (Myelin Removal Beads II, MACS Miltenyi Biotec) accordingly to the manufacturer's instruction for 500 mg of tissue. Briefly, brain tissue was suspended in 1800 μL of MACS buffer and incubated with 200 μL of myelin Microbeads (MACS Miltenyi Biotec) at 4 °C for 15 min. Cells were washed, centrifuged for 10 min at 300 ***g*** and re‐suspended in MACS buffer (3 × 1000 μL per mouse brain).

For the GBM PDOX model, tissue dissociation was performed using the Neural Dissociation Kit P (MACS Miltenyi Biotec) followed by myelin removal beads kit (Myelin Removal Beads II, MACS Miltenyi Biotec), as described above. An additional step was performed using the mouse cell depletion kit (MACS Miltenyi Biotec) following the manufacturer's protocol. Specifically, this step allowed to enrich murine stromal cells over human patient tumour cells. Briefly, the cell pellet was re‐suspended in 80 μL of cold HBSS with 0.5% BSA (Sigma‐Aldrich, Overijse, Belgium) and incubated with 20 μL of cell depletion cocktail for 1 × 10^7^ total cells at 4 °C for 15 min.

### 2.7 Single‐cell RNA‐sequencing using drop‐sequencing

Single‐cell suspensions derived from both naïve and GL261‐tumour‐bearing mice (Table 1) were obtained using an adapted protocol from MACS Miltenyi.

**Table 1:** Tumour volume measurement by MRI for biopsy collection at early, intermediate and late stage in GL261 tumour‐bearing WT and Acod1 KO mice used for scRNA‐seq analyses (1 mouse per condition).

Time‐point (stage)	Weeks post implantation	Tumour volume (mm^3^)	
		WT (gender)	*Acod1* KO (gender)
Early	2	6.11 (female)	9.61 (male)
Intermediate	3/4	22.63 (male)	20.48 (female)
Late	4/5	33.14 (male)	33.83 (male)

Specifically, tissue enzymatic dissociation was performed using the Neural Dissociation Kit P (MACS Miltenyi Biotec) (as described above) and the cell suspension was subsequently added into ‘C tubes’ for the gentle MACS Dissociator (gentleMACS™ Octo Dissociator with Heaters, Miltenyi Biotec). The 37C_ABDK_01 program was used to dissociate the brain tissue (> 100 mg). We centrifuged the cellular suspension and removed myelin using the myelin removal beads kit (Myelin Removal Beads II, MACS Miltenyi Biotec) accordingly to the manufacturer's instruction for 500 mg tissue. The eluted fraction was collected in 2% BSA RNase‐free solution. Cell viability and counting was assessed prior injection into Drop‐seq. A total of 5659 single cells were successfully sequenced and analysed. Cell handling, microfluidics fabrication, single cell droplet encapsulation and next‐generation sequencing preparation for Drop‐seq libraries were done as previously described [28].

### 2.8 Single‐cell RNA‐sequencing bioinformatics processing, data and statistical analyses

The FASTQ files were assembled from the raw BCL files using Illumina's bcl2fastq converter and ran through the FASTQC codes [Babraham bioinformatics; https://www.bioinformatics.babraham.ac.uk/projects/fastqc/] to check for the consistency in the library qualities. The monitored quality assessment parameters were: (a) quality per base sequence (especially for the read 2 of the gene); (b) per base N content; (c) per base sequence content and (d) over‐represented sequences. The libraries, which showed significant deviation, were re‐sequenced. Then, the FASTQ files were merged and converted to binaries using PICARD's fastqtosam algorithm. We have applied the Drop‐seq bioinformatics pipeline [15]. The sequencing reads were converted to digital gene expression (DGE) matrix. To normalize for the transcript loading between the beads, the averaged normalized expression levels (log2 (TPM + 1)) were calculated. To distinguish between cell‐containing and empty beads, a cumulative function of the total number of transcripts per barcode was plotted. Then, a threshold was applied empirically on the resulting ‘knee plot’ to estimate the beads exposed to the cell content. For each experimental batch, we retained top 1000 cell barcodes based on the cumulative distribution, leading to 8000 cells. We removed low‐abundance genes and only genes that were expressed in at least 30 cells were considered for further analysis. We additionally removed cells expressing < 1000 genes. Lastly, we concatenated each batch in a single matrix of the following dimensions: 5659 cells × 18 338 genes. These preanalytical filtering steps were processed using r environment (version 3.4.4) with the tidyverse package (version 1.3.1) [29]. The tSNE projection was processed with the rtsne package (version 0.16) [30] with a perplexity = 50, followed by a topological clustering with the library HDBSCAN (version 1.1‐5) [31] (Hierarchical DBSCAN with a minimum of 19 points – cells – for a cluster to be considered). We conducted statistical analysis for significant expression between groups using pairwise Wilcoxon test (wilcox.test from the r base package), while *P*‐values were adjusted with Benjamini Hochberg (BH) method.

Data visualization and downstream investigations were performed with tableau desktop software (Seattle, WA, USA) and r environment (R Core Team, Vienna, Austria).

In the PDOX experiment, we conducted scRNA‐seq data preprocessing as described above and used the seurat package (v4.0.5). For each sample, QC thresholds were empirically applied to exclude low‐quality cells based on the number of counts and features in the digital gene expression matrix before being merged to preserve unique reads from each sample. Only genes expressed in at least three cells, cells expressing at least 200 features and cells with < 30% mitochondrial reads were selected for further analysis. A total of 4448 cells (naïve nude = 1692, PDOX = 2756) with 24 067 genes were used for further analysis. The data were normalized using Seurat‐based ‘LogNormalize’ method. Dimensionality reduction was done using Uniform Manifold Approximation and Projection (UMAP) implemented in the seurat package (v4.0.5). We identified cell clusters based on the expression of known marker genes and the list of marker genes generated using the ‘FindAllMarkers’ function in Seurat. Differential expression analysis was done between selected clusters of cell types of interest using the Wilcoxon rank‐sum test and *P*‐values were adjusted using Bonferroni correction.

### 2.9 Kaplan–Meier survival curves

The Cancer Genome Atlas (TCGA) low and high grade glioma raw data together with the respective sample annotation were extracted from the GlioVis platform. Data were further normalized based on the library size (DESeq2) followed by a log2 transformation. Tumour‐associated microglia‐ and macrophage‐like transcriptional signatures were used to assign a score for each patient. Signatures were obtained from identified TAM I and TAM II profiles in the GL261 mouse model, converting the corresponding mouse genes into human genes accessing Biomart from Ensembl using the r package biomart (version 2.44.1) and identifying shared genes between the GBM syngeneic murine model and patients from Muller et al. dataset [10] . In total, 702 TCGA patients (LGG: 351 and GBM: 351) have been stratified based on their score, with 50% of the highest and lowest score selected to calculate the Kaplan–Meier survival curves using the r software packages *survival* and *ggplot2* for data visualization. The corresponding *P*‐value was computed based on a log rank test.

Survival analyses conducted in the GL261 mouse model were performed according to humane endpoints guidelines, including loss of locomotor activity, weight loss (up to 20%) and central nervous system symptoms. The survival time was measured from the day of tumour cell implantation until the day of euthanasia and median mouse survival time was calculated in graphpad for each group (WT mice = 8; *Acod1* KO mice = 8 mice) using the Mantel‐Cox signed‐rank statistical test.

### 2.10 Single cell trajectory inference analysis

Single cell trajectory inference analysis was done with Monocle 2 in r (version 3.6.3) using default parameters [32, 33]. The branching method orders cells along a trajectory based on gene expression similarities. Monocle 2 uses reversed graph embedding to describe multiple fate decisions in a fully unsupervised manner. Branches in the trajectory represent cell fate decisions through a developmental process. To test genes underlying the differences observed along the trajectory, ‘differentialGeneTest’ function was used to identify genes showing significant changes between the different states as a function of pseudotime, while ‘plot_genes_in_pseudotime’ function was used to plot the expression levels of identified genes.

### 2.11 Gene ontology analyses

DAVID (Database for Annotation, Visualization and Integrated Discovery) gene functional classification tool (http://david.abcc.ncifcrf.gov) was used to investigate and interpret the respective functional biological terms from the large gene lists of differentially expressed genes. We represented Gene Ontology (GO) terms enrichment using cytoscape software (National Institute of General Medical Sciences, https://cytoscape.org/). Each node represents a GO term and the size of each node is proportional to the number of nodes from the correspondent query set with that term. Only nodes with *P*‐value < 0.001 were chosen for network representation.

### 2.12 Mouse brain CD11b ^+^ cell isolation

Murine brain CD11b^+^ isolated cells were enriched by magnetic separation using CD11b beads (MACS Miltenyi Biotec) for RNA extraction or for flow cytometry phenotyping experiments. Briefly, 1 × 10^7^ cells were re‐suspended in 90 μL of PBS supplemented with 0.5% BSA (Sigma‐Aldrich) and 2 mm EDTA (MACS buffer) and incubated with 10 μL of CD11b beads (MACS Miltenyi Biotec) at 4 °C for 20 min. Cells were washed with MACS buffer, centrifuged for 10 min at 300 ***g*** and re‐suspended in 500 μL of MACS buffer at a density of 1 × 10^8^ cells. The cell suspension was applied into the LS columns (MACS Miltenyi Biotec) and the CD11b^+^ fraction was eluted. Flow cytometry experiments to evaluate the lymphocytic population were performed without prior CD11b^+^ beads isolation. Flow cytometry acquisition was performed using a FACSAria IIu SORP cytometer (Becton Dickinson, Franklin Lakes, NJ, USA) and data were further analysed using flowjo version 10.6.1 (Becton Dickinson).

### 2.13 Flow cytometry analyses

Single‐cell suspension was obtained as previously described. The cells were re‐suspended in ice‐cold HBSS with 2% FBS and 10 mm HEPES (FACS buffer) and filtered through a 70 μm nylon mesh (CellTrics, Norderstedt, Germany). For multicolour phenotyping, cells were blocked with Fc receptor‐binding inhibitor (anti‐mouse CD16/CD32 monoclonal antibody; 1 : 100; eBioscience, Waltham, MA, USA) for 15 min at 4 °C to reduce binding of nonspecific Fc‐gamma receptors, and then stained with fluorochrome‐conjugated antibodies for 30 min at 4 °C in the dark. The following antibodies were used in this study: rat anti‐mouse CD45 monoclonal antibody (clone 30‐F11), FITC; rat anti‐mouse CD74 monoclonal antibody (clone In‐1), FITC; rat anti‐mouse CD11b monoclonal antibody (clone M1/70), Percp‐Cy5.5; rat anti‐mouse P2RY12 monoclonal antibody (clone S16007D) PE and mouse anti‐mouse MHC‐II (clone AF6‐1201) APC. Unstained (control) and stained cells were washed and re‐suspended in 100 μL of FACS buffer prior acquisition. Before acquisition, the performance of the instrument was assessed using CS&T beads according to the manufacturer's instructions. Single‐stain controls were prepared with UltraComp eBeads (eBioscience) following the manufacturer's instructions and thus used to calculate the compensation matrix. Hoechst (0.1 μg·mL^−1^, Bisbenzimide, 33342; Sigma) or Zombie NIR (1 : 1000 dilution in PBS, Biolegend, Amsterdam, The Netherlands) was added for dead cell discrimination. Samples were run on FACSAria IIu SORP cytometer (Becton Dickinson) and flow cytometry data was analysed using flowjo software (v. 10.6.1, Becton Dickinson).

### 2.14 RNA extraction and qPCR analyses

Total RNA was extracted from BMDMs and freshly isolated CD11b^+^ cells from tumour‐bearing mice at late stage using the RNeasy Mini Kit (Qiagen), according to the manufacturer's instructions. RNA concentration was quantified by NanoDrop (NanoDrop Technologies, Wilmington, DE, USA) and RNA quality was assessed by the quotient of the 28S to 18S ribosomal RNA electropherogram peak using a bioanalyser (Agilent 2100; Agilent Technologies, Diegem, Belgium). For cDNA synthesis, RNA was reverse‐transcribed using SuperScript™ III reverse transcriptase (10 000 U; Invitrogen/Life Technologies) with 1 μL (50 μm)/reaction oligo(dT)20 (25 μm; Invitrogen/Life Technologies) as primer according to the manufacturer's instructions. Reverse transcription was performed at 50 °C for 60 min. Gene expression reaction mixtures contained 2 μL of diluted cDNA, 10 μL of Fast SYBR Green Master Mix (Applied Biosystems/Thermo Fisher Scientific, Waltham, MA, USA) and 0.5 μL of each 10 μm forward and reverse primers. PCRs were carried out in 384‐well plates on a ViiA™ 7 real‐time PCR system (Applied Biosystems/Thermo Fisher Scientific, Waltham, MA, USA) using the following programme: 95 °C for 20 s, 40 cycles at 95 °C for 1 s and 60 °C for 20 s. Samples were run in triplicates, and the mean *C*_t_ (threshold cycle) values were used to calculate the relative amount of product by the ΔΔ*C*_t_ method using 60S ribosomal protein L27 (Rpl27) as housekeeping gene. The specific primer sequences were as follows: Acod1 forward: 5′ GCA ACA TGA TGC TCA AGT CTG 3′; Acod1 reverse: 5′ TGC TCC TCC GAA TGA TAC CA 3′; Cd74 forward: 5′ GAC CCA GGA CCA TGT GAT GC 3′; Cd74 reverse: 5′ TTC CTG GCA CTT GGT CAG TAC TTT A 3′; H2‐Ab1 forward: 5′ TCA CTG TGG AGT GGA GGG CA 3′; H2‐Ab1 reverse: 5′ GGC AGT CAG GAA TTC GGA GC 3′; H2‐Aa forward: 5′ TCT GTG GAG GTG AAG ACG AC 3′; H2‐Aa reverse: 5′ AGG AGC CTC ATT GGT AGC TGG 3′; Irf1 forward: 5′ ACT CGA ATG CGG ATG AGA CC 3′; Irf1 reverse: 5′ GCT TTG TAT CGG CCT GTG TG 3′; RpL27 forward: 5′ TGG AAT TGA CCG CTA TCC CC 3′; Rpl27 reverse: 5′ CCT GTC TTG TAT CGC TCC TCA A 3′.

### 2.15 Immunofluorescence staining and microscopy imaging acquisition

Coronal sections of 12 μm thickness were prepared adopting the standard protocol with minor modifications [34]. Briefly, sections were washed (PBS with 0.1% Triton X‐100), permeabilized (PBS with 1.5% Triton X‐100), blocked (PBS with 5% BSA) and incubated with the following primary antibodies: rabbit anti‐Iba1 (1 : 1000; Biocare Medical, Antwerpen, Belgium), rat anti‐MHC‐II (1 : 100; Abcam, Cambridge, UK), rat anti‐CD74 FITC (1 : 50; eBioscience) and mouse anti‐IRF1 (1 : 100; Santa Cruz Biotechnology, Heidelberg, Germany). Secondary antibodies against the appropriate species were incubated for 2 h at room temperature. Cell nuclei were counterstained with Hoechst (1 mg·mL^−1^; Sigma). Sections were mounted on glass slides cover slipped using FluoromountTM Aqueous Mounting Medium (Sigma). For each brain section, at least 5 random 40× and 63× confocal images along the tumour margin and the tumour core were acquired with a Zeiss LSM880 microscope (Jena, Germany). High‐resolution XYZ stack images (1.024 × 1.024 pixels per Z step) were taken with a step size of 0.50 μm. Cell quantifications were performed using NIH imagej software (NIH, Bethesda, MD, USA) and values for single mouse are represented with distinct shape. Hoechst staining was used as reference for tumour localization.

### 2.16 SDS/PAGE and western blotting analyses

Cells were collected in 600 μL RIPA lysis buffer and stored at −80 °C before protein extraction. Samples were centrifuged at 10 000 ***g*** for 10 min at 4 °C and supernatants were harvested. Protein concentrations were measured with Bio‐Rad Protein Assay Dye Reagent Concentrate (500‐0006, Bio‐Rad, Temse, Belgium). Proteins were diluted in RIPA lysis and loading buffers. Heat‐denatured protein samples were separated on 4–12% BisTris‐polyacrylamide gel electrophoresis (NP0322BOX, Invitrogen) followed by transfer to polyvinylidene flouride (PVDF) membranes 0.2 μm (LC2005, Invitrogen). After blocking with 5% (wt/vol) dry milk in TBS containing 0.1% triton (TBST), the membrane was incubated overnight at 4 °C with primary anti‐IRG1 antibody (Ab222411, Abcam) diluted 1 : 250 in 1% (wt/vol) BSA in TBST with constant shaking. After three washing steps with TBST, the membrane was incubated with anti‐rabbit antibody coupled to horseradish peroxidase and revealed by chemoluminescence using Pierce™ ECL detection reagents (Thermo Fisher Scientific). For the second hybridization, the membrane was incubated with anti‐actin antibody (MAB 1501, Millipore, Overijse, Belgium) for 90 min at RT in 1% (wt/vol) BSA in TBST with constant shaking. After three washing steps with TBST, the membrane was incubated with anti‐mouse antibody coupled to horseradish peroxidase and revealed by chemoluminescence.

### 2.17 Raw data files

All relevant datasets are within the paper and its supporting information files (Figs S1–S10) and (Tables S1–S6). We deposited the raw scRNA‐seq data in Gene Expression Omnibus (GEO) database under the accession number GSE158016.

### 2.18 Statistical analyses

Data were analysed using the graphpad prism 8 software (GraphPad software, La Jolla, CA, USA) and R environment (R Core Team). Unless otherwise indicated, all data are presented as mean ± standard error of the mean (SEM) of at least three independent biological experiments. Statistical analysis was performed using Unpaired *t* test or Two‐way ANOVA. All differences were considered significantly different at *P*‐value < 0.05 and were annotated as follows: *< 0.05, **< 0.01, ***< 0.001, ns > 0.05.

## 3 Results and Discussion

### 3.1 Single‐cell transcriptomics reveals cellular diversity and cell type‐specific differential gene expression in naïve and GL261 tumour‐bearing wild type and ACOD1/IRG1 knock‐out mice

**Fig. 1:**
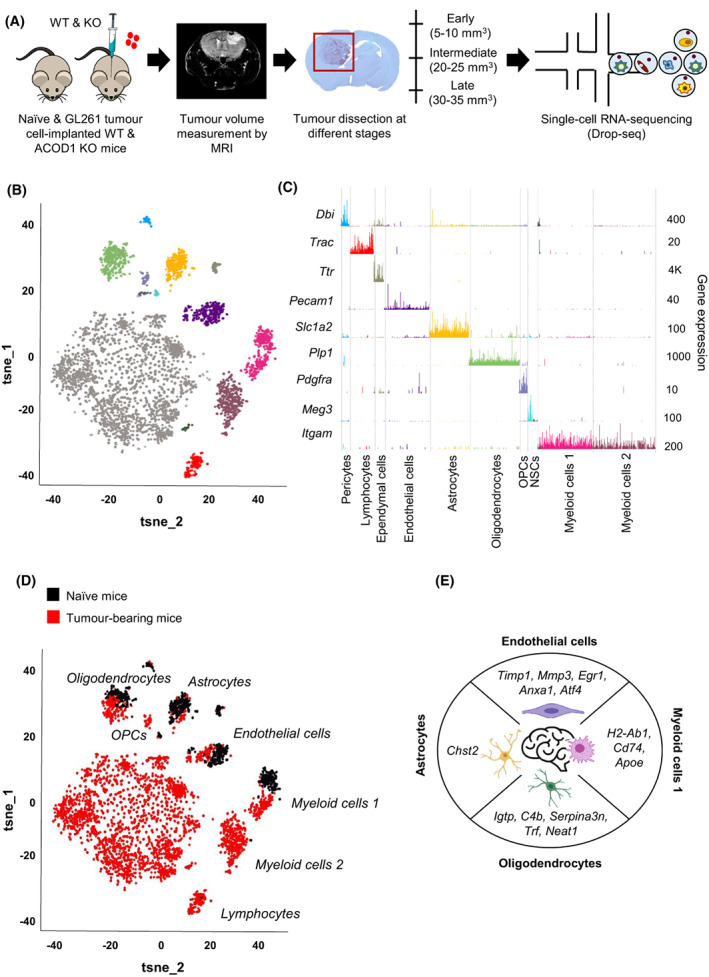
Cell‐type diversity in naïve and GL261 tumour‐bearing mice at different tumour stages, both from wild type and ACOD1/IRG1 knock‐out mice. (A) Flowchart depicting the overall design of the scRNA‐seq analyses. Naïve‐ and macro‐dissected brain tumour regions from both wild type and ACOD1/IRG1 knock‐out (KO) mice were processed by scRNA‐seq analyses. Samples were collected at different time points (early: 5–10 mm^3^; intermediate: 20–25 mm^3^; late: 30–35 mm^3^) according to tumour volume measured by magnetic resonance imaging (MRI). One biological replicate per experimental condition (WT/naïve; WT/early; WT/intermediate; WT/late; ACOD1/IRG1 KO/naïve; ACOD1/IRG1 KO/early; ACOD1/IRG1 KO/intermediate; ACOD1/IRG1 KO/late) has been taken into account for subsequent analyses. (B) 2D‐tSNE representation of all single cells included in the study (*n* = 5659 cells) grouped within 12 cell clusters. (C) Cell type‐specific markers allowing the identification of stromal cell types: Pericytes (*Dbi* ^+^), lymphocytes (*Trac* ^+^), ependymal cells (*Ttr* ^+^), endothelial cells (*Pecam1* ^+^), astrocytes (*Slc1a2* ^+^), oligodendrocytes (*Plp1* ^+^), oligodendrocyte precursor cells (OPCs, *Pdgfra* ^+^), neural stem cells (NSCs, *Meg3* ^+^), myeloid cells 1 (*Itgam* ^+^) and myeloid cells 2 (*Itgam* ^+^). See Fig. S1 for additional cell type‐specific markers used for clusters annotation. (D) 2D‐tSNE representation showing naïve (in black) and tumour‐associated (in red) cells. (E) Examples of the most up‐regulated genes (*P*‐value < 0.01, log2 FC > 0.5) per cell type in tumour‐bearing mice.

To investigate the heterogeneity of the TME in GBM, both at baseline and under ACOD1/IRG1 deficiency, we dissected brain tissue from naïve and GL261 tumour‐bearing mice at early (5–10 mm^3^), intermediate (20–25 mm^3^) and late (30–35 mm^3^) stage of tumour progression, both from wild type (WT) and age‐matched ACOD1/IRG1 knock‐out (KO) C57BL/6N mice. Briefly, we took advantage of the GL261 (mouse glioma 261) syngeneic murine model as a widely used paradigm for immunotherapy studies in GBM [35]. This model allows the engraftment of immortalized tumour cells from the same strain with low immune rejection, thus enabling the investigation of an immunocompetent TME *in vivo*, including functional T and B cells [36, 37, 38]. Recent studies aimed at comparing datasets obtained in GBM patients with distinct GBM syngeneic mouse models identified high correlation levels with both the 005 and GL261 models, thus serving as reliable preclinical models recapitulating several GBM patient features [39]. For our aims, the tissue was digested to a single‐cell suspension and analysed using scRNA‐seq to profile hundreds of cells isolated from the corresponding naïve and orthotopic syngeneic GL261‐implanted mice (Fig. 1A). Following preanalytical filtering of the scRNA‐seq experiments, we obtained a matrix composed of 5659 single cells (*n* = 18 338 genes). In order to reduce the dimensionality of the matrix, we applied t‐Distributed Stochastic Neighbourhood Embedding followed by unsupervised topological clustering with DBSCAN on the 2D projection of the tSNE. We identified 12 cell clusters with distinct gene expression signatures, irrespective of the tumour burden and genotype (Fig. 1B). We annotated 11 of them (*n* > 30 cells) based on cell type‐specific gene markers [40, 41] and gene set enrichment analysis (GO) of up‐regulated genes in the correspondent clusters. Specifically, in addition to tumour cells (*Cd44* ^+^, *n* = 3332 cells), we identified 10 stromal clusters that we classified as pericytes (*Dbi* ^+^, *n* = 61 cells), lymphocytes (*Trac* ^+^, *n* = 178 cells), ependymal cells (*Ttr* ^+^, *n* = 73 cells), endothelial cells (*Pecam1* ^+^, *n* = 328 cells), astrocytes (*Slc1a2* ^+^, *n* = 289 cells), oligodendrocytes (*Plp1* ^+^, *n* = 365 cells), oligodendrocyte precursor cells (OPCs, *Pdgfra* ^+^, *n* = 60 cells), neural stem cells (NSCs, *Meg3* ^+^, *n* = 36 cells) and myeloid cells 1 and 2 (*Itgam* ^+^, *n* = 836 cells) (Fig. 1C, Fig. S1A). Cells in the additional small subset (*n* = 20 cells) expressed myeloid markers (e.g. *Itgam, Aif1*), but clustered independently from the annotated main myeloid clusters (Fig. 1B). The analysis of additional specific markers provided robust molecular definitions of the major cell types present in the brain of naïve and tumour‐bearing mice (Fig. S1). Notably, identities, markers and proportions of cell types in naïve mice matched previous single‐cell droplet‐based sequencing data from mouse brain tissue [42], indicating that our results were robust to the inclusion of tumour‐affected brains. In addition, the proportion of the cell types identified here were similar to the ones described in recent single‐cell studies conducted in GBM patients [18, 43, 44]. Lastly, GBM is an archetypal heterogeneous tumour characterized by a significant extent of common genetic alterations affecting tumour progression [45]. In line with previous studies [46], *Myc* and *Trp53* were the main highly overexpressed genes in tumour cells compared to nonmalignant cells (Fig. S2).

Focusing on the TME, we first observed that lymphocytes, OPCs and a subset of myeloid cells were solely present in tumour‐bearing mice (Fig. 1D). Next, a direct comparison of tumour‐associated cells versus the corresponding cells in naïve mice enabled to identify differentially expressed genes (*P*‐value < 0.01; log2 FC > ± 0.5) (Fig. 1E, Table S1) according to the defined cell types. We observed a prominent transcriptional adaptation in tumour‐associated endothelial cells, oligodendrocytes as well as in the myeloid subset (Fig. 1D), which has been described also in patients [18, 44]. We detected cell‐type‐specific up‐regulated genes across the four CNS resident cells (Fig. 1E, Fig. S3A). Notably, all four cell types displayed a shared antigen processing and presentation gene signature (e.g. *H2‐D1*, *H2‐K1* and *B2m*) (Table S1). Specifically, approximately 15% of the genes, representing more than 90 genes (e.g. *Junb*, *Spp1*, *Cd74, B2m, H2‐K1 and H2‐Q7*), were up‐regulated in both tumour‐associated endothelial and myeloid cells 1 compared to the corresponding naïve cells (Fig. S4A), indicating that endothelial cells are also active immune modulators in the TME of GBM. Indeed, the tumour vasculature is a key element of the TME, which largely contributes to the immunosuppressive features of GBM [47]. We corroborated these observations in a patient‐derived orthotopic xenograft (PDOX) preclinical mouse model characterized by its angiogenic nature, as previously described [26]. By conducting scRNA‐seq analyses of the tumour biopsy and the whole brain, respectively from the PDOX model and naïve nude mouse, we discriminated naïve from tumour‐associated cells (Fig. S4B) and, in line with the results obtained in the GL261 model, we identified the corresponding myeloid cell subsets (naïve, myeloid cells 1 and 2) as well as the endothelial cluster (Fig. S4C). In the PDOX model, we detected 27% shared up‐regulated genes (*n* = 335) between tumour‐educated myeloid cells 1 and endothelial cells (Fig. S4D). Taken together, we detected 8% shared up‐regulated genes (*n* = 32 genes) between myeloid cells 1 and tumour endothelial cells across syngeneic GL261 and PDOX GBM murine models (Fig. S4E). Specifically, we identified genes involved in antigen presentation via MHC class I (e.g. *B2m* and *H2‐K1*) (Fig. S4C), thus indicating that endothelial cells display immunological signatures maintained across various GBM murine models.

Overall, these results show that, in analogy to GBM patients, the growing tumour in the analysed syngeneic mouse model induces the emergence of lymphocytes, OPCs and a subset of myeloid cells in the TME that are normally absent in the homeostatic CNS. Further, it specifically affects the transcriptional signature of the major resident CNS cell types, with the myeloid compartment displaying high heterogeneity, major tumour‐associated education and specific gene expression signatures shared with endothelial cells.

### 3.2 Tumour‐associated myeloid cells in glioblastoma are heterogeneous and display distinct transcriptional programmes

**Fig. 2:**
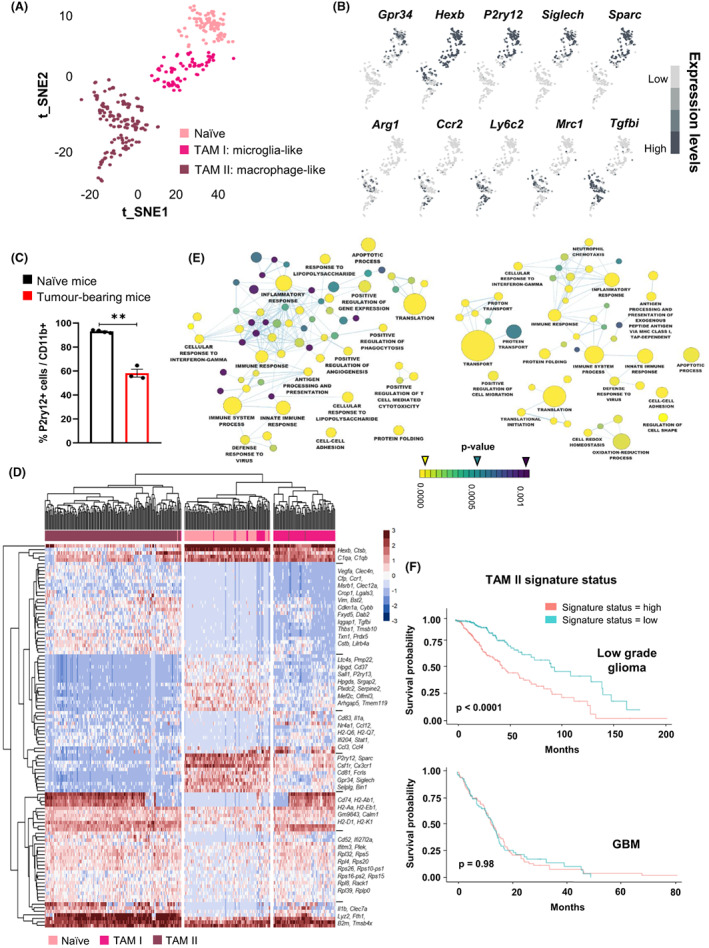
Microglia‐ (TAM I) and macrophage‐like (TAM II) subsets display discrete functional adaptation in the GBM syngeneic GL261 murine model. (A, B) Colour‐coded 2D‐tSNE representation showing (A) three distinct myeloid cell subsets in WT mice: naïve, TAM I and TAM II clusters and (B) the expression of microglia homeostatic genes (*Gpr34*, *Hexb*, *P2ry12*, *Siglech* and *Sparc*) and macrophage‐like markers (*Arg1, Ccr2, Ly6c2, Mrc1 and Tgfbi*), irrespective of the tumour stage. Results show one biological replicate per experimental condition (WT/naïve; WT/early; WT/intermediate; WT/late). (C) Percentage of CD11b^+^ P2ry12^+^ cells in naïve and tumour‐bearing mice quantified by flow cytometry at late stage of the disease. (D) Two‐way hierarchical heat‐map clustering analyses of the most differentially expressed genes (*P*‐value <0.01) for each myeloid cluster: naïve, TAM I and TAM II, irrespective of the tumour stage, Table S2). Results show one biological replicate per experimental condition (WT/naïve; WT/early; WT/intermediate; WT/late). Scale bar represents colour‐coded *z*‐scores. (E) Corresponding gene ontology functional network of TAM I (left graph) and TAM II (right graph) versus naïve microglia. Node size correlates to gene set numbers and annotated nodes defined as containing ≥ 15 genes. (F) Kaplan–Meier survival analysis in low and high grade glioma patients (TCGA‐LGG and TCGA‐GBM databases) with high and low TAM II‐enriched signature (*n* = 84 genes; e.g. *TGFBI, THBS1, VIM, IL1B, IL1RN, F13A1, CYBB*). Statistical analysis for (C) Unpaired Student *t* test (WT = 4, *Acod1* KO *n* = 3), mean ± SEM, **P < 0.01.

Similar to GBM patients, the myeloid compartment constituted the biggest cluster in the TME of the GL261 GBM mouse model (39.3% of the TME) (Fig. S3B) and displayed prominent transcriptional adaptation and heterogeneity, thus representing a relevant paradigm to deepen and address its molecular profile. Resident parenchymal microglia are difficult to segregate from peripheral monocyte‐derived cells, which prevalently constitute the myeloid compartment in GBM. Thus, we took advantage of our scRNA‐seq dataset obtained in WT mice to analyse the expression of known microglia and monocyte‐derived macrophage markers across naïve and the two TAM subsets identified by 2D‐tSNE, irrespective of the tumour stage (Fig. 2A). Naïve and TAM I clusters showed high expression levels of the microglia homeostatic genes (e.g. *Gpr34, Hexb, P2ry12, Siglech, Sparc*), while these genes were almost undetectable (except *Hexb*) in the TAM II cluster. Accordingly, the TAM II cluster exhibited high levels of peripheral monocytic‐derived macrophage markers (e.g. *Arg1, Ccr2, Ly6c2, Mrc1, Tgfbi*) (Fig. 2B). These observations were supported by flow cytometry analyses of the macro‐dissected tumour region to discriminate CD11b^+^ P2ry12^+^ from CD11b^+^ P2ry12^−^/low cells (Fig. S5A). Compared to naïve mice, where more than 95% of CD11b^+^ cells were P2ry12^+^ resident microglial cells, the amount of CD11b^+^ P2ry12^+^ cells in tumour‐bearing mice was significantly reduced (mean 58.16 ± 5.6%) (Fig. 2C). These analyses allowed to discriminate microglia‐like (TAM I) from macrophage‐like (TAM II) cells in the GL261 syngeneic model. Notably, our results are in line with recent single‐cell profiling studies of myeloid cells uncovering similar cellular distributions in the corresponding GBM mouse model and patients [48, 49].

Two‐way hierarchical heat‐map clustering analyses of the most differentially expressed genes (*P*‐value < 0.01) between naïve, microglia‐ and macrophage‐like cells (Table S2) revealed, in agreement with their different ontogeny, a less pronounced difference between naïve and tumour‐associated microglia compared to the monocyte/macrophage cluster (Fig. 2D). In line with the decrease of homeostatic genes in microglia under inflammatory conditions [28], tumour‐associated microglia displayed a decreased expression of these genes (e.g. *P2ry12, Sparc*, *Csf1r, Cx3Cr1, Fcrls, Gpr34, Siglech, Mef2c, Olfml3, Tmem119*) when compared to the naïve group (Fig. 2D). Moreover, as expected these genes were not detected in the TAM II population (Fig. 2D). On the other hand, TAM II subset showed up‐regulated genes associated with positive regulation of angiogenesis (e.g. *Vegfa*, *Lgals3, Il1β, Cybb, Thbs1, Plek, Vim, Stat1*) and metabolic redox metabolism (e.g. *Cybb, Msrb1*) (Fig. 2D). Both TAM I and TAM II exhibited increased expression levels of genes associated with antigen presentation (e.g. *Cd74, H2‐Ab1, H2‐Aa, H2‐Eb1, H2‐D1 and H2‐K1*) compared to the naïve group (Fig. 2D). Overall, these results point towards the heterogeneous composition of TAMs and their distinct adaptation profiles in the TME of GBM.

Gene set enrichment analysis of tumour‐associated microglia or tumour‐associated‐monocyte/macrophage transcriptional programmes revealed immunological terms shared by both cell types (e.g. inflammatory response and innate immune response). We also identified terms specifically associated with TAM I (e.g. positive regulation of phagocytosis and T cell‐mediated cytotoxicity) or TAM II (e.g. positive regulation of cell migration and oxidation–reduction process), suggesting distinct ontogeny‐based functional adaptations to the tumour (Fig. 2E). The comparison of the TAM I signature with distinct microglia‐like clusters identified by Ochocka et al. [48] uncovered similarities with the defined microglial group 7 (MG7), independently from the gender, and with the female‐associated MG2 cluster (Fig. S5B). The MG7 cluster exhibits overexpression of genes encoding components of MHC class I (e.g. *H2‐D1, H2‐K1, B2m*), while the MG2 cluster is characterized by high expression levels of early activation genes (e.g. *Nfkbia*, *Ccl3*, *Ccl12*). By conducting a similar comparison for the TAM II signature, we found great similarity with the defined intermediate state of monocyte and macrophage (intMoMΦ) cluster, characterized by specific genetic markers (e.g. *Lyz2*, *Tgfbi*, *Fth1*), in both male and female mice (Fig. S5C). As the intMoMΦ cluster represents the main cluster of infiltrative cells to the tumour described by Ochocka et al. [48], it corroborates the relevance of our identified TAM II signature.

Next, to strengthen our findings obtained in the GL261 syngeneic mouse model, we compared microglia‐like (TAM I) and monocyte/macrophage‐like (TAM II) transcriptional signatures with putative corresponding cell types recently described in GBM patients at single‐cell resolution [10]. Overall, 8.6% of up‐regulated genes in TAM I (*P* < 0.01; Log2 FC > 0.5) were shared with tumour‐associated microglia‐like cells in GBM patients. In addition, 7% of differentially expressed genes characterizing TAM II (*P* < 0.01; Log2 FC > 0.5) were mutually up‐regulated in blood monocyte‐derived macrophage‐like cells in GBM patients (Fig. S6A). We used the identified transcriptional signatures discriminating tumour‐associated microglia (*n* = 21 genes; e.g. *CCL4, CCL3, P2RY12, CX3CR1, BIN1, SELPLG, CD83, SALL1*) and macrophages (*n* = 84 genes; e.g. *TGFBI, THBS1, VIM, IL1B, IL1RN, F13A1, CYBB*) both in the GBM syngeneic murine model and in patients (Fig. S6A, Table S3), to verify their prognostic value in low and high grade glioma patients. For this, we took advantage of The Cancer Genome Atlas (TCGA) datasets allowing to link patient survival with corresponding bulk transcriptional data from two publicly available TCGA‐databases (TCGA‐LGG: low grade glioma and TCGA‐GBM: high grade glioma). Notably, a macrophage‐like enriched signature correlated with a worse patient survival compared to a microglia‐like enhanced programme in LGG patients. Nevertheless, our signatures did not allow to stratify GBM patients, which are overall characterized by higher levels of peripheral monocytic infiltration associated with a worse survival compared to LGG patients (Fig. 2F). Of note, we verified these signatures in our PDOX model and identified corresponding up‐regulated genes in TAM I and TAM II compared to naïve microglia (*P* < 0.01; Log2 FC > 0.5). Similarly to the GL261 model, we found microglia‐ and macrophage‐like signatures shared between the PDOX model and GBM patients (TAM I, *n* = 15 genes; e.g. *CCL4, CCL3, P2RY12, CX3CR1, BIN1*; TAM II, *n* = 9 genes; e.g. *TGFBI, PLAC8, IFITM3, TMSB10*) (Fig. S6B).

Taken together, our scRNA‐seq analyses enabled a clear separation of microglia from peripheral monocytic‐derived macrophages displaying key transcriptional and functional differences along their adaptation to the tumour, both in the GBM syngeneic mouse model and patients. Our results are in agreement with recent prognostic studies conducted in GBM patients showing that immunosuppressive immune cell infiltrates increase from grade II to grade IV [50] and a reduced immune suppressive phenotype correlates with extended survival, as observed in LGG patients [51]. Collectively, we demonstrate the relevance of discriminating between microglia and monocyte‐derived macrophages for prognostic purposes in glioma patients. We take advantage of this critical distinction to separately characterizing tumour‐associated microglia and macrophage subsets along GBM progression.

### 3.3 TAMs rapidly infiltrate the tumour and adapt along GBM progression

**Fig. 3:**
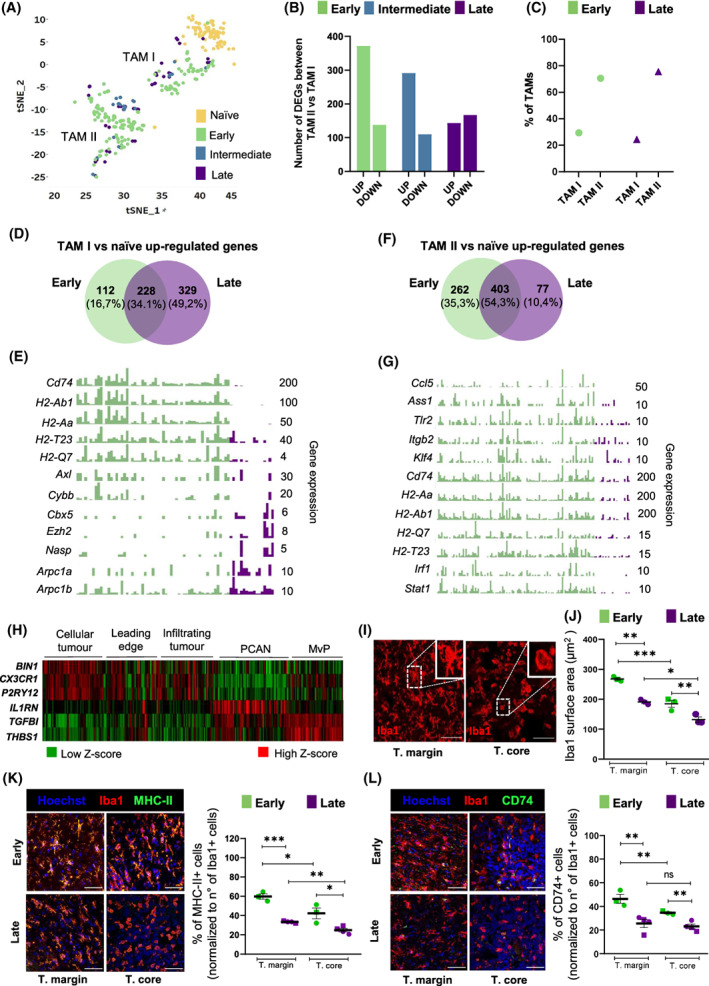
TAM subsets spatial and temporal characterization along glioblastoma development. (A–G) Results show one biological replicate per experimental condition (WT/naïve; WT/early; WT/intermediate; WT/late). (A) Myeloid tSNE plot colour‐ coded representation for tumour progression (green: early; blue: intermediate; purple: late stage). (B) Number of up‐ and down‐regulated genes (*P*‐value < 0.01, ¦log2 FC¦ > 0.5) between TAM II and TAM I along GBM progression. (C) Relative proportions of TAM I and TAM II subsets at early and late GBM stages obtained by scRNA‐seq analysis. (D) Venn diagram representation showing TAM I shared (*n* = 228) and exclusively up‐regulated genes at early (*n* = 112) and late (*n* = 329) stages versus naïve microglia. (E) Single‐cell bar plots showing selected top differentially expressed genes in TAM I between early and late GBM stages. Gene expression numbers on the right axis represent gene counts per cell. (F) Venn diagram representation showing TAM II shared (*n* = 403) and exclusively up‐regulated genes at early (*n* = 262) and late (*n* = 77) stages versus naïve microglia. (G) Single‐cell bar plots showing selected top differentially expressed genes in TAM II between early and late GBM stages. Gene expression numbers on the right axis represent gene counts per cell. (H) RNA‐sequencing profiles of laser‐microdissected regions of GBM patients for microglia (*BIN1, CX3CR1, P2RY12*) and peripheral monocyte‐derived cell (*IL1RN, TGFBI, THBSI*) marker genes. Data extracted from the Ivy Glioblastoma Atlas Project (PCAN: pseudopalisading cells around necrosis; MvP: microvascular proliferation). (I) Representative immunofluorescence pictures of Iba1 positive cells in the tumour margin and core in murine brain sections. (J) Quantification of Iba1 surface area in the tumour margin and tumour core at early and late stages. (K–L) Representative immunofluorescence pictures and quantification for (K) MHC‐II and (L) CD74 staining in the tumour margin and core at early and late stages. In (J–L), circles represent females and squares symbolize males. Statistical analysis for (J) Two‐way ANOVA with Sidak's multiple comparison corrections [early *n* = 3 (2 females and 1 male) and late *n* = 3 (females)], (K, L) Two‐way ANOVA with Sidak's multiple comparison corrections [early *n* = 3 (2 females and 1 male) and late *n* = 4 (2 females and 2 males)], mean ± SEM, *P < 0.05; **P < 0.01; ***P < 0.001; ns = not significant. Scale bars in I, K and L = 50 μm. CD74, HLA class II histocompatibility antigen gamma chain; Iba1, Allograft inflammatory factor 1; MHC‐II, Major histocompatibility complex class II molecules; MvP, Microvascular proliferation; PCAN, Pseudopalisading cells around necrosis; T. core, tumour core; T. margin, tumour margin; TAM I, tumour‐associated microglia; TAM II, tumour‐associated macrophage.

By studying TAM heterogeneity along GBM progression in WT mice at single‐cell resolution, we detected microglia‐like and macrophage‐like cell subsets in all analysed tumour stages (i.e. early, intermediate and late time points), indicating that, in agreement with prior observations [8], in this model the infiltration of monocyte‐derived macrophages occurs early during tumour growth (Fig. 3A). Notably, we observed a gradual decrease in the number of up‐regulated genes (early *n* = 372, intermediate *n* = 291 and late *n* = 143) and a relatively constant number of down‐regulated genes (early *n* = 138; intermediate *n* = 110 and late *n* = 167) between macrophage‐like and microglia‐like cells along tumour stages. These results indicate that the transcriptional programmes of microglia and peripheral infiltrated macrophages converge over time (Fig. 3B). Overall, the ratios of microglia‐like and macrophage‐like cells in the GBM TME did not change across early (TAM I: 29.35%; TAM II: 70.65%) and late (TAM I: 24.43%; TAM II: 75.57%) stages (Fig. 3C). Next, we sought to investigate microglia‐like and peripheral macrophage‐like cell transcriptional programmes along tumour progression separately, with a special focus at early and late stages.

Two‐way hierarchical heat‐map clustering analyses of the most differentially expressed genes (*P*‐value < 0.01) in TAM I across the tumour stages revealed three clusters mainly represented by naïve microglia, tumour‐associated microglia at early stages and an intermediate/late‐enriched group (Fig. S7A). We analysed up‐regulated genes characterizing microglia‐like cells at early and late tumour stages versus naïve microglia (Fig. 3D). We found great overlap (34.1%) of genes expressed by microglia‐like cells between the two stages (e.g. *H2‐D1, H2‐K1, Cd83, Il1b, Ccl12, Ccl4, Lyz2, Fth1, Ctsb, Atf3, Cst7, B2m, Cd52, Nfkbia*), indicating a core transcriptional programme maintained along GBM progression (Table S4). When comparing the levels of specific differentially expressed genes between early (*n* = 112) and late (*n* = 329) tumour stages, markers associated with antigen processing and presentation (e.g. *Cd74*, *H2‐Ab1*, *H2‐Aa*) or T‐cell activation and cytotoxicity (e.g. *H2‐T23*, *H2‐Q7*) and inflammatory response (e.g. *Axl*, *Cybb*) were largely decreased at later tumour stages (Fig. 3E, Fig. S7B). In parallel, genes associated with chromatin remodelling (e.g. *Cbx5*, *Ezh2, Nasp)* and actin nucleation/polymerization (e.g. *Arpc1a, Arpc1b*) were enhanced at later stages (Fig. 3E). In particular, we found a subset of microglia‐like cells up‐regulating *Ezh2* expression at late stage. Although studies have demonstrated that *Ezh2* is frequently overexpressed in a wide variety of cancers, mechanistic links of *Ezh2* expression in TAMs to cancer progression remains to be elucidated. In ovarian cancer, *Ezh2* has direct roles on T cell response and inhibition of *Ezh2* in tumour‐specific T cells increases the tumour burden *in vivo* [52].

We conducted similar analyses for the macrophage‐like subset. Two‐way hierarchical heat‐map clustering analyses of the most differentially expressed genes (*P*‐value < 0.01) in TAM II along the tumour stages revealed two main clusters represented by tumour‐associated macrophages at early stage and an intermediate/late‐enriched group (Fig. S7C, Table S4). We found prominent overlap (54.3%) of genes up‐regulated both at early and late tumour stages expressed by macrophage‐like cells compared to naïve microglia (e.g*. Lyz2, Apoe, Fth1, Il1β, H2‐K1, H2‐D1, Vim, Cd14, Cybb, Tgfbi*) indicating, similarly to microglia‐like cells, a main transcriptional programme preserved along GBM progression (Fig. 3F).

The comparison of the levels of specific differentially expressed genes between early and late tumour stages revealed the decrease of macrophage activation markers (e.g. *Ccl5, Ass1, Tlr2, Itgb2, Klf4*) as well as, similarly to microglia‐like cells, the down‐regulation of genes associated with antigen processing and presentation (e.g. *Cd74, H2‐Ab1, H2‐Aa*) and regulation of T‐helper cells (e.g. *H2‐Q7, H2‐T23*). In addition, type I interferon genes (e.g. *Irf1, Stat1*) were drastically reduced at late stage (Fig. 3G, Fig. S7D). Taken together, the reduced antigen cross‐presentation ability of both microglia‐ and macrophage‐like cells at later time points may add to the recognized poor recruitment of T cells to the tumour site in GBM [53], thus dampening potential T‐cell‐mediated tumour eradication along its progression.

To corroborate these results at the protein level, we compared the expression levels of CD74 and MHC‐II (encoded by *H2‐Ab1*) at early and late stages in corresponding tissue sections. To discriminate brain‐resident microglia and blood derived‐monocytes/macrophages in immunohistological analyses, we took advantage of the Ivy Glioblastoma Atlas Project to infer TAM spatial localization in laser‐micro‐dissected regions of GBM patients [54]. Here, we observed an enrichment of microglia‐like cells (expressing *BIN1, CX3CR1, P2RY12*) at the leading edge of the tumour, while macrophage‐like cells (expressing *IL1RN, TGFBI, THBS1*) were mostly detected in the microvascular compartment (Fig. 3H). Similar findings were described by spatial scRNA‐seq of the myeloid compartment in GBM patients where *TGFBI*, *VEGFA* and *IL1RN* were mainly expressed by macrophages in the tumour core, while microglial cells enriched in the tumour periphery displayed a reduced expression of these genes [18]. Supporting these observations, 2‐photon microscopy in murine GBM sections recently revealed two distinct cell types with different morphological properties composing TAMs. Specifically, cells with reduced branching and increased surface area compared to naïve resident parenchymal cells mainly accumulated at the tumour margins and represented tumour‐associated microglia, while monocyte‐derived macrophages displaying shrank surface area and increased migratory properties were mainly located in the tumour core [55]. In agreement with this, we observed a significant reduction of the surface area of macrophage‐like infiltrative cells in the tumour core compared to larger and branched microglia‐like enriched cells in the tumour margin independent of tumour stage (Fig. 3I,J and Fig. S7E). In line with our scRNA‐seq data, we observed a significant decrease of the antigen presenting cell markers MHC‐II (Fig. 3K) and CD74 (Fig. 3L) at late GBM stage in both the tumour margin and core. Notably, we observed a higher percentage of Iba1^+^ MHC‐II^+^ cells in the tumour margin compared to tumour core both at early and late stages (Fig. 3K), highlighting spatial heterogeneity of TAMs at the protein level. These differences were independent from the mouse gender.

Collectively, these analyses show that TAMs display distinct transcriptional programmes along GBM progression, with both microglia and monocytic‐derived macrophages exhibiting decreased antigen presenting cell features at later tumour stages compared to earlier phases.

### 3.4 TAMs display higher immunological reactivity under aconitate decarboxylase 1 deficiency affecting T cell recruitment

**Fig. 4:**
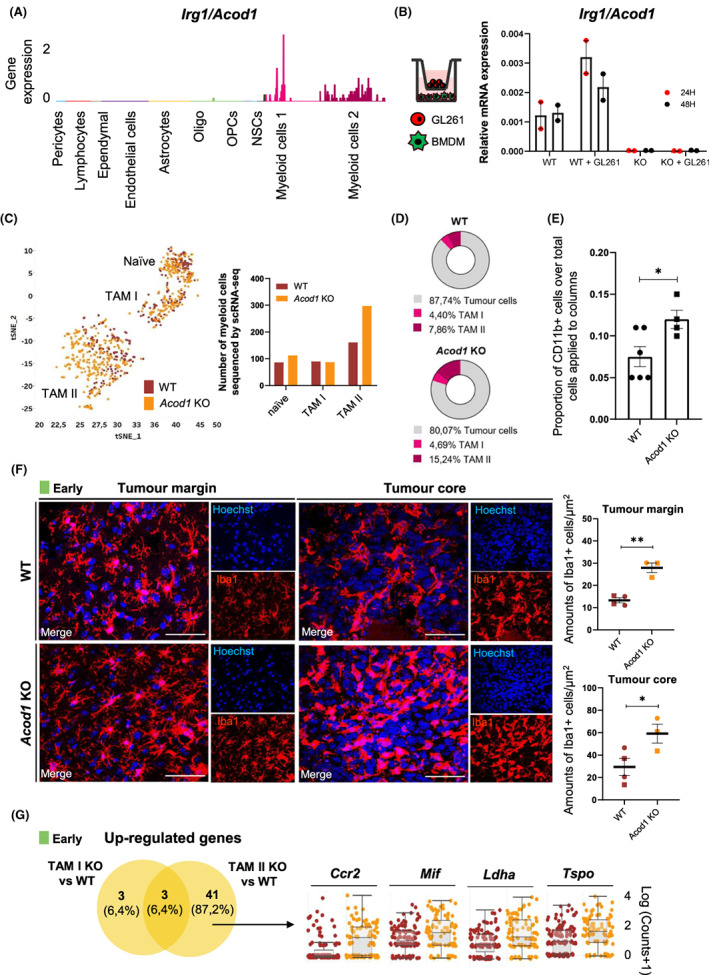
*Acod1* expression is induced in TAMs and its deficiency affects their recruitment. (A) *Irg1/Acod1* expression levels across the main 10 stromal cell‐types identified by scRNA‐seq. Results show one biological replicate per experimental condition (WT/naïve; WT/early; WT/intermediate; WT/late; ACOD1/IRG1 KO/naïve; ACOD1/IRG1 KO/early; ACOD1/IRG1 KO/intermediate; ACOD1/IRG1 KO/late). (B) Analysis by qPCR of the expression levels of *Acod1* (normalized using *Rpl27* as housekeeping gene) in BMDMs from WT and *Acod1* KO mice upon co‐culture with GL261 tumour cells at 24 and 48 h (WT *n* = 2, *Acod1* KO *n* = 2). (C) Myeloid tSNE plot colour coded (brown: WT; orange: *Acod1* KO) and respective number of myeloid cells sequenced by scRNA‐seq according to the genotype (WT, naïve: 86 cells; TAM I: 87 cells; TAM II 159 cells. *Acod1* KO, naïve: 112 cells; TAM I: 91 cells; TAM II: 298 cells) taking into account all the tumour stages. (D) Graphical representation depicting the proportions of TAMs and tumour cells in WT (upper) and *Acod1* KO (bottom) mice taking into account all the tumour stages (WT/naïve; WT/early; WT/intermediate; WT/late; ACOD1/IRG1 KO/naïve; ACOD1/IRG1 KO/early; ACOD1/IRG1 KO/intermediate; ACOD1/IRG1 KO/late). (E) Number of CD11b^+^ cells isolated from WT and *Acod1* KO from tumour‐bearing mouse brains at late stage. Bars represent the ratio of the number of CD11b^+^ cells over the number of total cells applied to the columns. (F) Immunofluorescence pictures depicting Iba1 positive cells in the tumour margin (left) and core (right). Number of Iba1 positive cells were quantified in WT and *Acod1* KO mice at early GBM stage. (G) Venn diagram representation showing shared and exclusive up‐regulated genes in *Acod1* KO TAM I (*n* = 3) and TAM II (*n* = 41) at early stage versus their respective counterparts in age‐matched WT cells. Notch plot representation of selected genes exclusively up‐regulated by TAM II in *Acod1* KO mice compared with WT mice at early stage. In (E, F), circles represent females and squares denote males. Statistical analysis for (A) pairwise Wilcoxon test with *P*‐value adjusted with Benjamini Hochberg method; (E) Unpaired Student *t* test (WT = 6, *Acod1* KO *n* = 4); (F) Unpaired Student *t* test (WT early *n* = 4*; Acod1* KO early *n* = 3), mean ± SEM. *P < 0.05, **P < 0.01. Scale bars in F = 50 μm. *Acod1*, aconitate decarboxylase 1; BMDMs, bone marrow‐derived macrophages; *Ccr2*, C‐C chemokine receptor type 2; Iba1, Allograft inflammatory factor 1; KO, knock‐out; *Ldha*, Lactate dehydrogenase A; *Mif*, Macrophage migration inhibitory factor; TAM I, tumour‐associated microglia; TAM II, tumour‐associated macrophage; *Tspo*, Translocator protein; WT, wild‐type.

In mammals, immune‐responsive gene 1 protein (IRG1), encoded by aconitate decarboxylase 1/immunoresponsive gene 1 (*Acod1/Irg1*), catalyses the production of itaconate from the decarboxylation of cis‐aconitate, an intermediate metabolite of the TCA cycle [19, 56]. Itaconate is one of the most up‐regulated metabolites in activated macrophages [57] exhibiting anti‐inflammatory properties, thus contributing to the resolution of inflammation [20, 21]. Interestingly, it has been recently shown that low doses of itaconate inhibits inflammation, while it promotes inflammation at high doses [58]. Due to the emerging role of various immune metabolites in macrophage reprogramming towards specific phenotypes, we sought to analyse the role of *Acod1/Irg1* in TAM adaptation along GBM progression and characterize TAM subsets under ACOD1 deficiency at single cell resolution. *Acod1* deficiency did not affect the distinct cell types identified by scRNA‐seq (Fig. S8A). In the GL261 model, we exclusively detected *Acod1*/*Irg1* induction across the myeloid compartment and, at a larger extent, within the macrophage‐like subset (Fig. 4A). We observed similar results in the Brain Tumour Immune Micro Environment dataset acquired in GBM patients by RNA‐seq [59]. Indeed, *ACOD1*/*IRG1* expression was up‐regulated in both CD49D^low^ microglial cells and CD49D^high^ macrophages, with higher expression levels in IDH‐wild type compared to IDH‐mutant gliomas (Fig. S8B). Microarray analysis of RNA extracted from CD11b^+^ MACS‐isolated cells from naïve and GL261‐implanted mouse brains showed also a significant increase of *Acod1/Irg1* expression in tumour‐bearing (*n* = 3) compared to naïve mice (*n* = 3) (Fig. S8C) [11]. Bone marrow‐derived macrophages (BMDMs) co‐cultured with GL261 tumour cells *in vitro* showed increased expression of *Acod1* compared to mono‐cultured BMDMs, while its expression was undetectable in BMDMs obtained from *Acod1* KO mice (Fig. 4B). However, in these co‐culture conditions, contrarily to BMDMs treated with LPS (100 ng·mL^−1^) for 6 h, IRG1/ACOD1 protein was not detectable (Fig. S8D). These results are in agreement with its weak induction at the mRNA level in these co‐culture conditions.

The analysis of TAM subsets by scRNA‐seq across all the stages suggested an over‐representation of the macrophage‐like population in *Acod1* KO mice (81.15%, 298 cells sequenced) compared to age‐matched WT mice (63.11%, 159 cells sequenced) (Fig. 4C). Moreover, a higher proportion of TAM II cells versus tumour cells was observed in *Acod1* KO compared to WT mice, while we found no differences for TAM I cells (Fig. 4D). Albeit we did not detect differences in the total number of bone‐marrow precursors between naïve WT and *Acod1* KO mice (Fig. S8E), we observed an increase in the number of CD11b^+^ cells in the brain of *Acod1* KO compared to WT tumour‐bearing mice (Fig. 4E). Indeed, immunofluorescence analyses revealed a significant increase in the number of Iba1^+^ cells at early stages at both the tumour margin and core, thus confirming enhanced infiltration of myeloid cells in *Acod1* KO mice (Fig. 4F). Investigation of the exclusively up‐regulated genes in microglia‐like and macrophage‐like cells at early stages in *Acod1* KO mice versus their corresponding counterparts in WT mice identified a major transcriptional effect on macrophage‐like (*n* = 41 genes) compared to microglia‐like (*n* = 3 genes) cells (Fig. 4G). Genes associated with TAM recruitment, such as *Ccr2, Mif, Ldha and Tspo*, were uniquely overexpressed in macrophage‐like cells from *Acod1* KO mice (Fig. 4G). Specifically, the CCL2/CCR2 axis is essential for monocyte migration into the inflamed CNS [60, 61]. Further, macrophage migration inhibitory factor (MIF) plays an important role in regulating inflammatory responses in innate immune cells [62] and can directly interact with CXCR2 and CXCR4 promoting inflammatory activity and leukocyte chemotaxis in cancer [63].

**Fig. 5:**
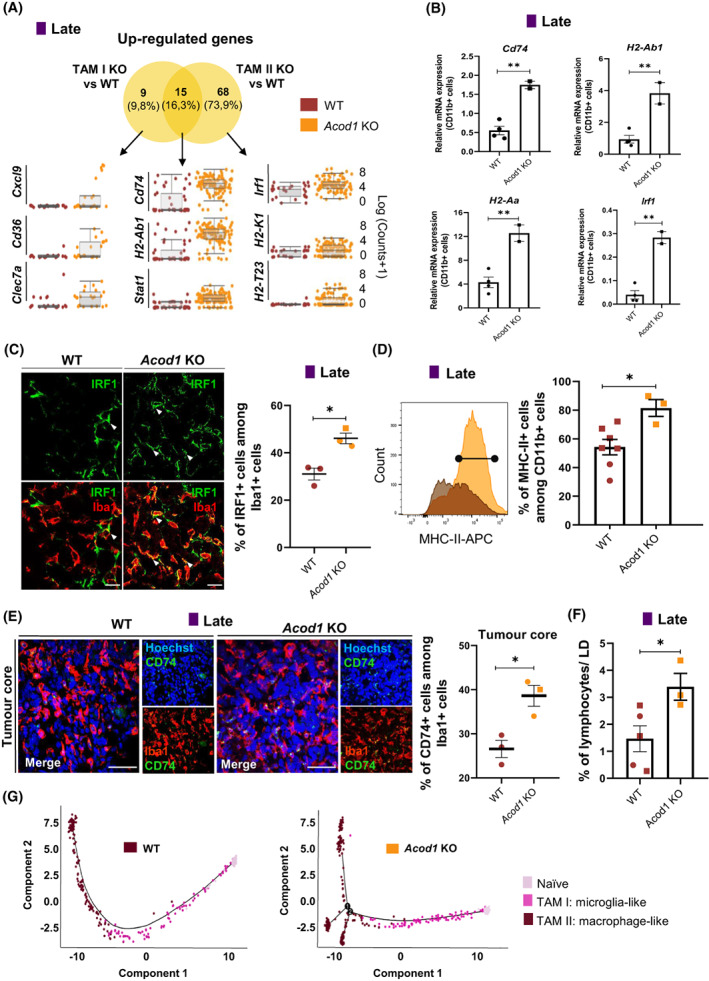
TAMs under *Acod1* deficiency display higher antigen presenting cell programmes associated with increased lymphocytic recruitment at late GBM stage. (A) Venn diagram representation showing shared (*n* = 15) and exclusive up‐regulated genes in *Acod1* KO TAM I (*n* = 9) and TAM II (*n* = 68) at late stage versus their respective counterparts in age‐matched WT cells (Table S5). Corresponding notch plot representations of selected shared or unique genes up‐regulated in TAM I and TAM II cells in *Acod1* KO mice compared to age‐matched WT mice at late stage. Results show one biological replicate per experimental condition (WT/naïve; WT/early; WT/late; ACOD1/IRG1 KO/naïve; ACOD1/IRG1 KO/early; ACOD1/IRG1 KO/late). (B) Analysis by qPCR of the expression levels of *Cd74, H2‐Ab1, H2‐Aa* and *Irf1* genes (normalized using *Rpl27* as housekeeping gene) in CD11b^+^ cells isolated from WT and *Acod1* KO mice at late stages. (C) Immunofluorescence pictures (left) and quantification (right) of IRF1 expression in Iba1^+^ cells in the tumour core region at late stage in *Acod1* KO and WT mouse brain sections. Arrowheads indicate examples of co‐localization of IRF1 with Iba1 staining. (D) Representative overlay histogram (left) and quantification (right) of MHC‐II expression in TAMs analysed in WT and *Acod1* KO mice at late stage by flow cytometry. (E) Immunofluorescence pictures (left) and quantification (right) of CD74 expression in Iba1^+^ cells in the tumour core region at late stage in *Acod1* KO and WT mouse brain sections. (F) Percentage of CD11b^−^ CD45^+^ lymphocytes at late stage quantified by flow cytometry. (G) Single cell trajectory inference analysis of 335 myeloid cells from WT naïve and tumour‐bearing mice (left graph) and 501 myeloid cells from *Acod1* KO naïve and tumour‐bearing mice (right graph). In (C–F), circles represent females and squares symbolize males. Statistical analysis for (B) Unpaired Student *t* test (WT *n* = 4 (3 females and 1 male), *Acod1* KO *n* = 2 (females)); (C) Unpaired Student *t* test (WT *n* = 3 (females), *Acod1* KO *n* = 3 (2 females and 1 male); (D) Unpaired Student *t* test (WT *n* = 7 (2 females and 5 males), *Acod1* KO *n* = 3 (males)), (E) Unpaired Student *t* test (WT *n* = 3 (females), *Acod1* KO *n* = 3 (2 females and 1 male)), (F) Unpaired Student *t* test (WT *n* = 5 (2 females and 3 males), *Acod1* KO *n* = 3 (males)), mean ± SEM, *P < 0.05; **P < 0.01. Scale bars = 20 μm in (C) and 50 μm in (E). *Cd36*, CD36 molecule; *Cd74*, CD74 molecule; *Clec7a*, C‐type lectin domain containing 7A; *Cxcl9*, C‐X‐C motif chemokine ligand 9; *H2‐aa*, major histocompatibility complex, class II; *H2‐Ab1*, major histocompatibility complex, class II; *H2‐K1*, major histocompatibility complex, class I, a; *H2‐T23*, major histocompatibility complex, class I, E; *Irf1*, interferon regulatory factor 1; KO, knock‐out; *Stat1*, signal transducer and activator of transcription 1; TAM I, tumour‐associated microglia; TAM II, tumour‐associated macrophage; WT, wild‐type.

Similarly to early stages, the number of exclusively up‐regulated genes was higher in macrophage‐like (*n* = 68 genes) compared to microglia‐like (*n* = 9 genes) cells when comparing *Acod1* KO with WT tumour‐bearing mice at late stage (Fig. 5A, Table S5), confirming that the lack of *Acod1/Irg1* mainly affected the transcriptional programme of peripheral infiltrating macrophages compared to microglia. Gene set enrichment analysis of macrophage‐like cell exclusively up‐regulated genes at late GBM stage in *Acod1 KO* compared to WT mice uncovered enrichment of terms associated with inflammation (e.g. *Irf1*), antigen processing and presentation via MHC class I (e.g. *H2‐K1*) and T cell‐mediated cytotoxicity (e.g. *H2‐T23*) (Fig. 5A, Fig. S9A). The common 15 microglia‐like and macrophage‐like cell up‐regulated genes in *Acod1* KO compared to WT mice were associated with antigen presenting cell (e.g. *Cd74, H2‐Ab1*) and inflammatory (*Stat1*) markers (Fig. 5A), reflecting an enhanced immune activation at late stage in *Acod1* KO mice. In agreement with these results at single‐cell resolution, we detected a higher induction of antigen presentation (e.g. *Cd74*, *H2‐Ab1*, *H2‐Aa*) and inflammatory (e.g. *Irf1*) transcripts in *ex vivo* CD11b^+^ isolated TAMs from *Acod1* KO compared to WT tumour‐bearing mice at late stages (Fig. 5B). IRF family members play essential roles in regulating immune responses [64, 65] and seminal work has shown that *Irf1* KO mice exhibit impaired NK cell maturation and defective Th1 responses [66, 67]. Additionally, IRF1 operates as a tumour suppressor and its inactivation has been shown to significantly increase risk of malignancy [68]. To investigate the expression of IRF1 at the protein level, we conducted immunofluorescence analysis and detected higher numbers of IBA1^+^ IRF1^+^ positive cells in the tumour core in *Acod1* KO compared to WT mice (Fig. 5C). Amongst the downstream targets of IRF1, we detected by flow cytometry an increased expression of MHC‐II in TAMs isolated at late stage from *Acod1* KO compared to WT mice (Fig. 5D, Fig. S9B). Additionally, in brain sections from *Acod1* KO tumour‐bearing mice, we detected a significant increase of CD74 expressed by macrophage‐like cells, which were enriched in the tumour core, compared to WT mice (Fig. 5E). As gliomas are characterized as ‘immunologically silent’ in IDH‐mutant or ‘lymphocyte‐depleted’ in IDH‐wild‐type subtypes [69], we sought to investigate whether the ablation of *Acod1*, which induces an enhanced TAM immunogenic phenotype, could influence the recruitment of T cells to the tumour site. Indeed, we observed an increase of the lymphocytic population in *Acod1 KO* compared to WT mice, both in our scRNA‐seq dataset (Fig. S9C) and by flow cytometry (Fig. 5F, Fig. S9B), thus suggesting an effective crosstalk between TAMs and the adaptive immune cell compartment. We further identified up‐regulated genes comparing lymphocytes isolated from *Acod1* KO and WT mice at early stages (*P* < 0.01; Log2 FC > 0.5; *n* = 17 genes; e.g. *Dbi, Ifitm3, Lgals1, Mt1, Pfn1*) (Fig. S9D, Table S6), while we did not conduct the corresponding analysis at late stage due to the low number of gathered lymphocytes in WT mice (Fig. S9C).

Lastly, in order to elucidate if specific TAM subsets under ACOD1 deficiency display enhanced immunogenic phenotypes, we conducted single cell trajectory inference analyses. We showed higher macrophage‐like cell heterogeneity in *Acod1 KO* compared to WT mice, thus suggesting that ACOD1 deficiency also supports TAM diversity (Fig. 5G). Specifically, pseudo‐time analyses uncovered four distinct cellular states across the TAM II subset under *Acod1* deficiency (Fig. S10A,B). Further analysis of exclusive genes driving the most prominent cellular state (cellular state four) revealed a TAM II subset exclusively present in *Acod1* deficient tumour‐bearing mice, which might support leukocyte migration and T cell activation (e.g. *Ccl17, Ccl22, Ccr7, IL12b, Cd1d1*) to the tumour site (Fig. S10C). This subset was also characterized by higher expression levels of genes encoding serine proteinase inhibitors (e.g. *Serpinb6b* and *Serpinb9*) (Fig. S10C), which have been described to play a critical role in T lymphocyte‐mediated immunity [70]. Although *Acod1/Irg1* silencing in macrophages has been shown to significantly reduce the peritoneal tumour burden [23], the analysis of tumour growth in GL261 tumour‐bearing mice did not show significant differences between WT and *Acod1* KO (data not shown), neither we detected differences in the mouse survival (Fig. S10D), most probably due to the very high aggressiveness of the tumour in the analysed model.

## 4 Conclusion

In summary, despite scRNA‐seq analyses conducted in one biological replicate per experimental condition represent a limitation of the study, we here elucidated the diversity of the myeloid compartment along GBM progression and under ACOD1 deficiency by corroborating the main findings by flow cytometry, immunohistological and targeted gene expression analyses. We demonstrate that the myeloid compartment is the most affected and heterogeneous stromal cell component in GBM, with microglia and macrophages acquiring key transcriptional differences and rapidly adapting along GBM progression. Specifically, we show that TAMs display a decreased antigen‐presenting cell signature along GBM progression, which is retained under ACOD1 deficiency. Collectively, these results are in line with the anti‐inflammatory role of ACOD1/itaconate [71], since their absence skewed TAMs in GBM towards a more reactive and immunogenic phenotype. Mechanistically, itaconate modifies a range of proteins in macrophages, including KEAP1, which leads to NRF2 activation and induction of NRF2‐dependent genes encoding anti‐inflammatory and antioxidant factors. Similarly, itaconate might also modify GILT (IFI30), a protein that regulates antigen presentation [71]. However, how itaconate and GILT might potentially contribute to the decrease of antigen presentation marks warrants further investigation. From a therapeutic point of view, although immune checkpoint blockade therapy has markedly improved survival in several immunogenic cancers, such as melanoma, its efficacy has not been extended to GBM patients, as observed in a randomized phase III clinical trial for recurrent GBM (CheckMate 143; Identifier NCT 02017717) [72]. As it is becoming increasingly evident that a mono‐therapeutic approach is unlikely to provide anti‐tumour efficacy, the combination of ACOD1 suppression in TAMs, which enables to harness both the innate and adaptive immune systems, together with the inhibition of immune checkpoints may advance therapeutic successes against GBM and other solid tumours.

## Conflict of interest

The authors declare no conflict of interest.

## Author contributions

YPA, SPN and AlM designed the project; YPA, KG, AO, YAY, CS, AS and RH performed experiments; YPA, ArM, KG, YAY, AC, AG and AlM analysed experiments; AP supported *in silico* analyses; DC supported *in vivo* mouse experiments; AS set up and supervised scRNA‐seq analyses; YPA and AlM wrote the manuscript; all the authors edited and approved the manuscript.

### Peer review

The peer review history for this article is available at https://publons.com/publon/10.1002/1878‐0261.13287.

## Supporting information


**Fig. S1.** Gene expression of distinct cell‐types identified by scRNA‐seq in the GL261 syngeneic murine model and naïve mice, related to Figure 1.
**Fig. S2.** Expression of *Myc* and *Trp53* genes in the GL261 GBM murine model, related to Figure 1.
**Fig. S3.** Gene expression of distinct cell‐types present in naïve and tumour‐bearing mice, related to Figure 1.
**Fig. S4.** Comparisons of gene expression profiles between myeloid cells 1 and tumour endothelial cells in the GBM syngeneic GL261 and patient‐derived orthotopic xenograft (PDOX) mouse models, related to Figure 1.
**Fig. S5.** Characterization of TAM I and TAM II subsets by FACS and by comparing their gene expression signatures with datasets gathered from the literature, related to Figure 2.
**Fig. S6.** Microglia‐ versus macrophage‐like features in GBM, related to Figure 2.
**Fig. S7.** Differential microglia and monocytic‐derived macrophage transcriptional adaptation along GBM progression, related to Figure 3.
**Fig. S8.** *Acod1* expression levels in TAMs, related to Figure 4.
**Fig. S9.** TAM and lymphocytic signatures under *Acod1* deficiency, related to Figure 5.
**Fig. S10.** TAM II cellular state diversity under *Acod1* deficiency, related to Figure 5.

**Table S1.** Up‐regulated differentially expressed genes in tumour‐associated clusters (astrocytes, endothelial, oligodendrocytes, myeloid) versus correspondent naïve cells (p‐value < 0.01 and log2 FC > 0.5), related to Figure 1.

**Table S2.** List of the most differentially expressed genes across the myeloid clusters (Naïve, TAM I and TAM II), irrespective of the tumour stage (p‐value < 0.01), related to Figure 2.

**Table S3.** Common transcriptional signatures between tumour‐associated microglia and macrophages in the GBM syngeneic murine model and in patients used to assign a score for each TCGA patient, related to Figure 2.

**Table S4.** Up‐regulated differentially expressed genes at early and late stages for TAM I and TAM II versus naïve cells (p‐value < 0.001 and log2 FC > 0.5), related to Figure 3.

**Table S5.** Up‐regulated differentially expressed genes at late stage for TAM I KO and TAM II KO versus correspondent WT cells (p‐value < 0,001 and log2 FC > 0,5), related to Figure 5.

**Table S6.** Up‐regulated differentially expressed genes at early stage comparing lymphocytes from KO and WT mice (p‐value < 0.01 and Log2 FC > 0.5), related to Supplementary Figure 9.

## Data Availability

The data that support the findings of this study are openly available in NCBI's Gene Expression Omnibus (GEO) and are accessible through. https://www.ncbi.nlm.nih.gov/geo/query/acc.cgi?acc=GSE158016, GEO Series accession number GSE158016.
