# Supplementary material for: Elucidating tumour‐associated microglia/macrophage diversity along glioblastoma progression and under ACOD1 deficiency
Source: Mol Oncol. 2022 Aug 15;16(17):3167–91. doi: 10.1002/1878-0261.13287 (PMC9441003; doi:10.1002/1878-0261.13287)
Supplement: Supplementary file 1 — Fig. S1. Gene expression of distinct cell‐types identified by scRNA‐seq in the GL261 syngeneic murine model and naïve mice, related to Figure 1. Fig. S2. Expression of Myc and Trp53 genes in the GL261 GBM murine model, related to Figure 1. Fig. S3. Gene expression of distinct cell‐types present in naïve and tumour‐bearing mice, related to Figure 1. Fig. S4. Comparisons of gene expression profiles between myeloid cells 1 and tumour endothelial cells in the GBM syngeneic GL261 and patient‐derived orthotopic xenograft (PDOX) mouse models, related to Figure 1. Fig. S5. Characterization of TAM I and TAM II subsets by FACS and by comparing their gene expression signatures with datasets gathered from the literature, related to Figure 2. Fig. S6. Microglia‐ versus macrophage‐like features in GBM, related to Figure 2. Fig. S7. Differential microglia and monocytic‐derived macrophage transcriptional adaptation along GBM progression, related to Figure 3. Fig. S8. Acod1 expression levels in TAMs, related to Figure 4. Fig. S9. TAM and lymphocytic signatures under Acod1 deficiency, related to Figure 5. Fig. S10. TAM II cellular state diversity under Acod1 deficiency, related to Figure 5. [file MOL2-16-3167-s003.pdf]

Supplementary Figure 1

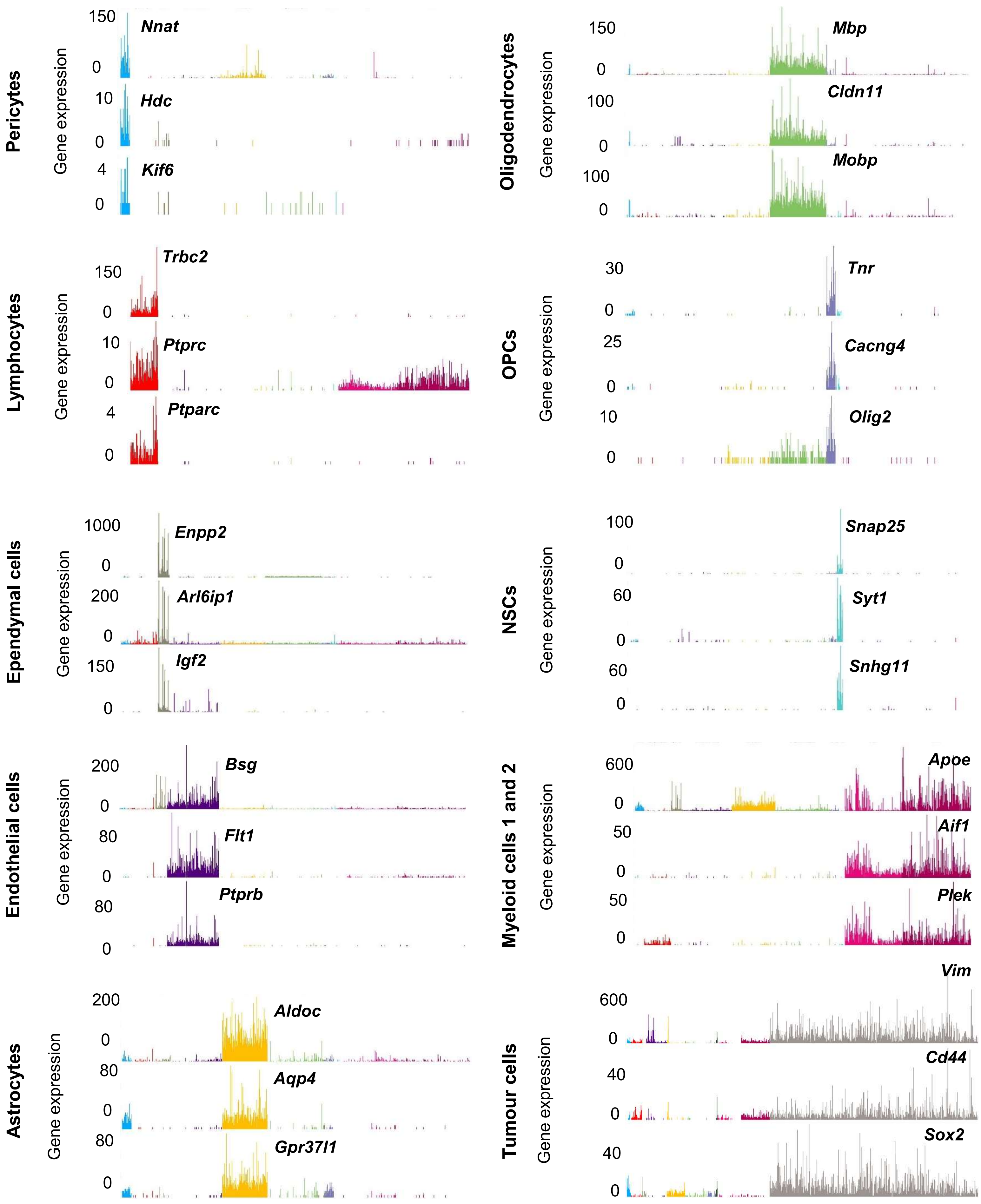

Supplementary Figure 1. Gene expression of distinct cell-types identified by scRNA-seq in the GL261 syngeneic murine model and naïve mice, related to figure 1.

Bar plots of additional cell type-specific markers. Pericytes (*Nnat, Hdc, Kif6*), lymphocytes (*Trbc2, Ptprcap, Ptprc*), ependymal cells (*Enpp2, Arl6ip1, Igf2*), endothelial cells (*Bsg, Flt1, Ptprb*), astrocytes (*Aldoc, Aqp4, Gpr37l1*), oligodendrocytes (*Mbp, Cldn11, Mobp*), OPCs (*Tnr, Cacng4, Olig2*), NSCs (*Snap25, Syt1, Snhg11*), myeloid cells 1 and 2 (*Apoe, Aif1, Plek*), tumour cells (*Vim, Cd44, Sox2*). Abbreviations: OPCs, oligodendrocyte precursor cells; NSCs, neural stem cells.

Supplementary Figure 2

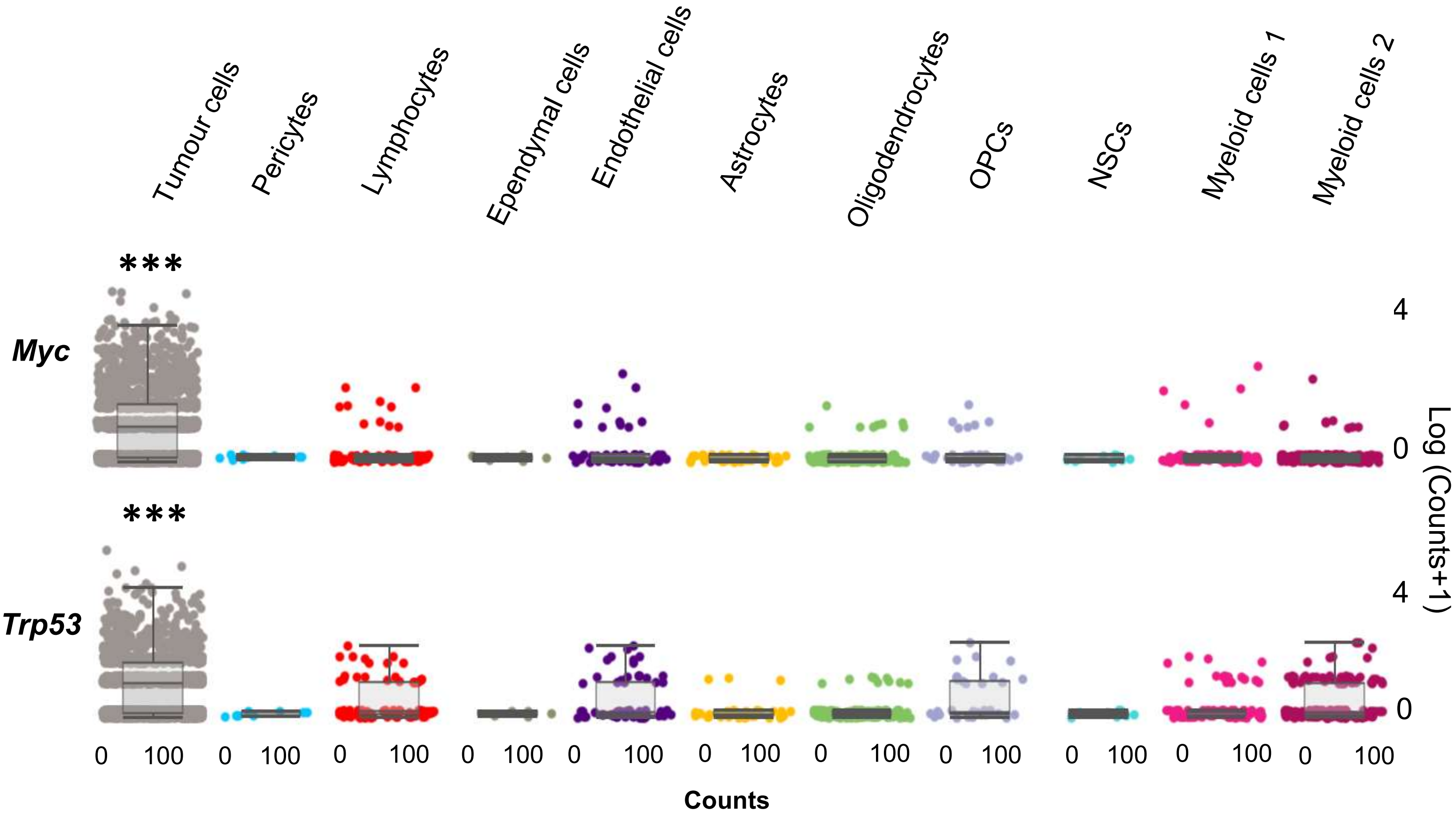

Supplementary Figure 2. Expression of *Myc* and *Trp53* genes in the GL261 GBM murine model, related to figure 1.

Myc and *Trp53* gene expression levels across tumour cells and 10 main stromal cell-types identified by scRNA-seq analyses. Data are represented as mean ± SEM, *** $p < 0.001$.

Supplementary Figure 3

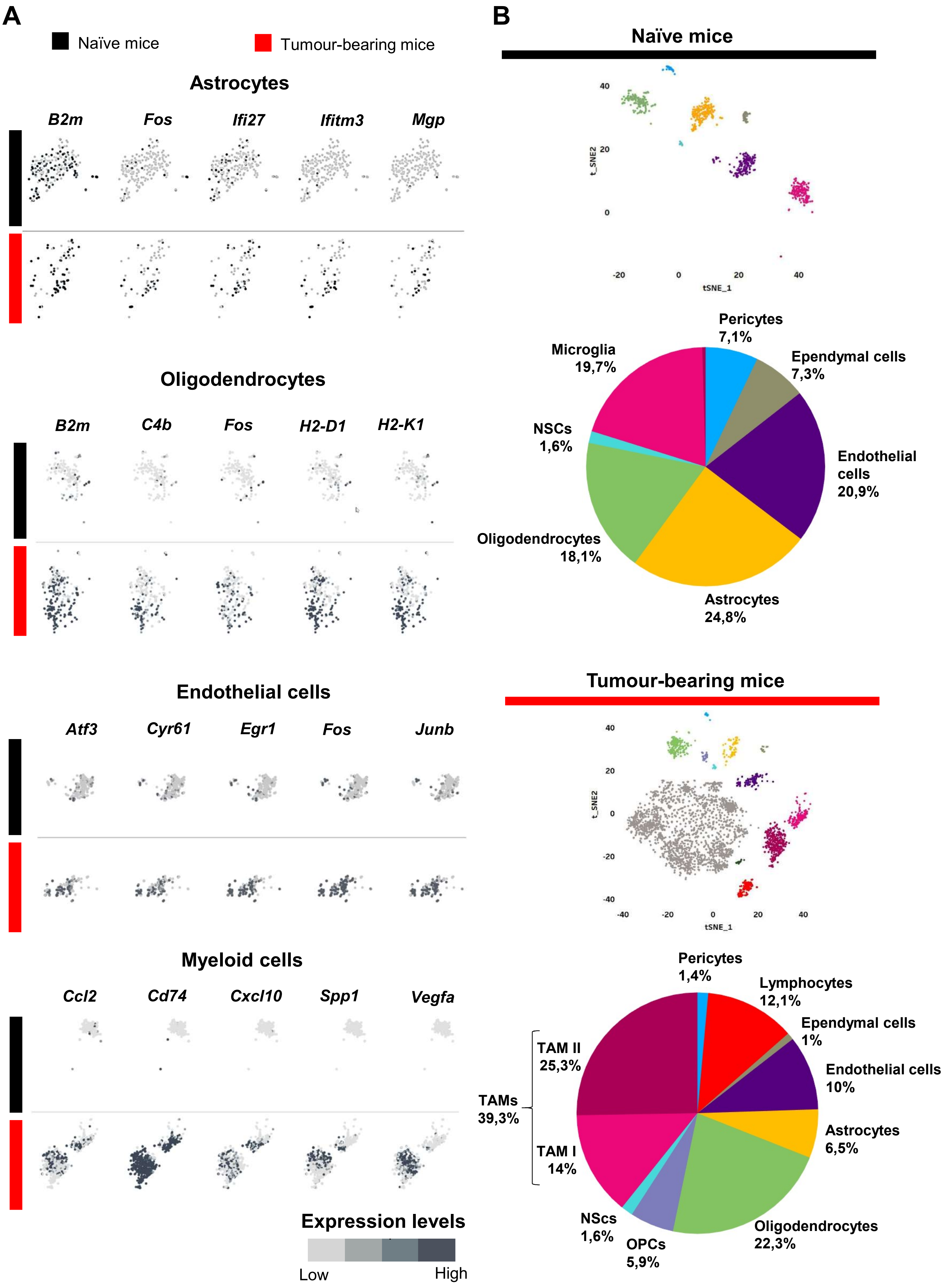

Supplementary Figure 3. Gene expression of distinct cell-types present in naïve and tumour-bearing mice, related to figure 1.

(A) 2D-tSNE representation of the top up-regulated genes in tumour-associated clusters (astrocytes, oligodendrocytes, endothelial cells, myeloid cells) compared to their naïve counterparts. **(B)** 2D-tSNE plot and respective cell-type proportion shown in pie chart of 2’282 isolated cells from naïve samples (upper panels) and 3’377 isolated cells from tumour-bearing samples (lower panels).

Supplementary Figure 4

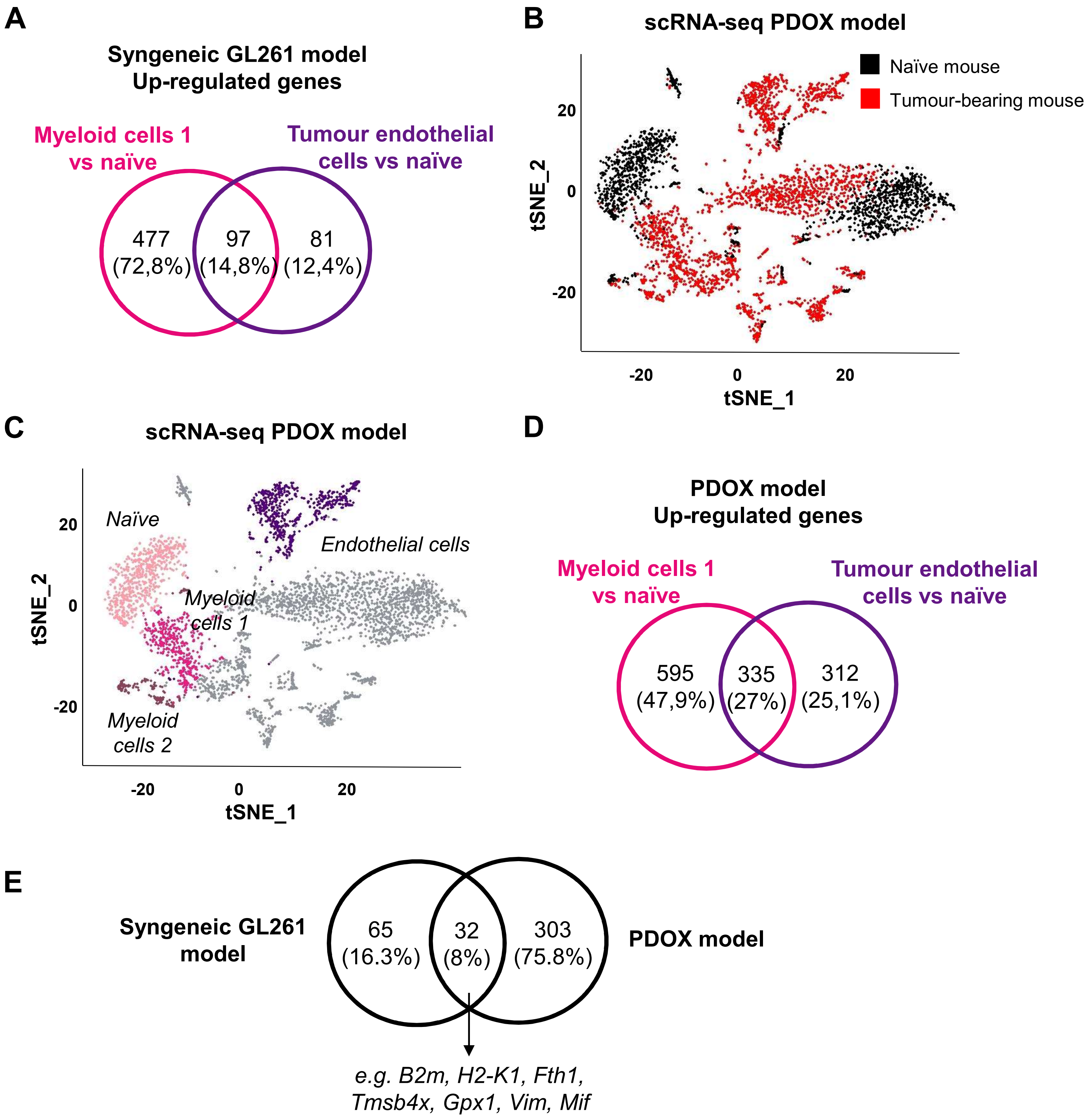

Supplementary Figure 4. Comparisons of gene expression profiles between myeloid cells 1 and tumour endothelial cells in GBM syngeneic GL261 and patient-derived orthotopic xenograft (PDOX) mouse models, related to figure 1.

(A) Venn diagram representation showing shared and exclusive up-regulated genes in myeloid cells 1 and tumour endothelial cells in syngeneic GL261 model. **(B)** 2D-tSNE representation showing naïve (in black) and tumour-associated (in red) cells in the PDOX preclinical model. **(C)** Colour-coded 2D-tSNE representation showing three distinct myeloid cell subsets (naïve, myeloid cells 1 and 2) and endothelial cells in naïve and PDOX model. **(D)** Venn diagram representation showing shared and exclusive up-regulated genes in myeloid cells 1 and tumour endothelial cells in PDOX model. **(E)** Venn diagram representation of common up-regulated genes in myeloid cells 1 and tumour endothelial cells across the syngeneic GL261 and PDOX GBM murine models.

Supplementary Figure 5

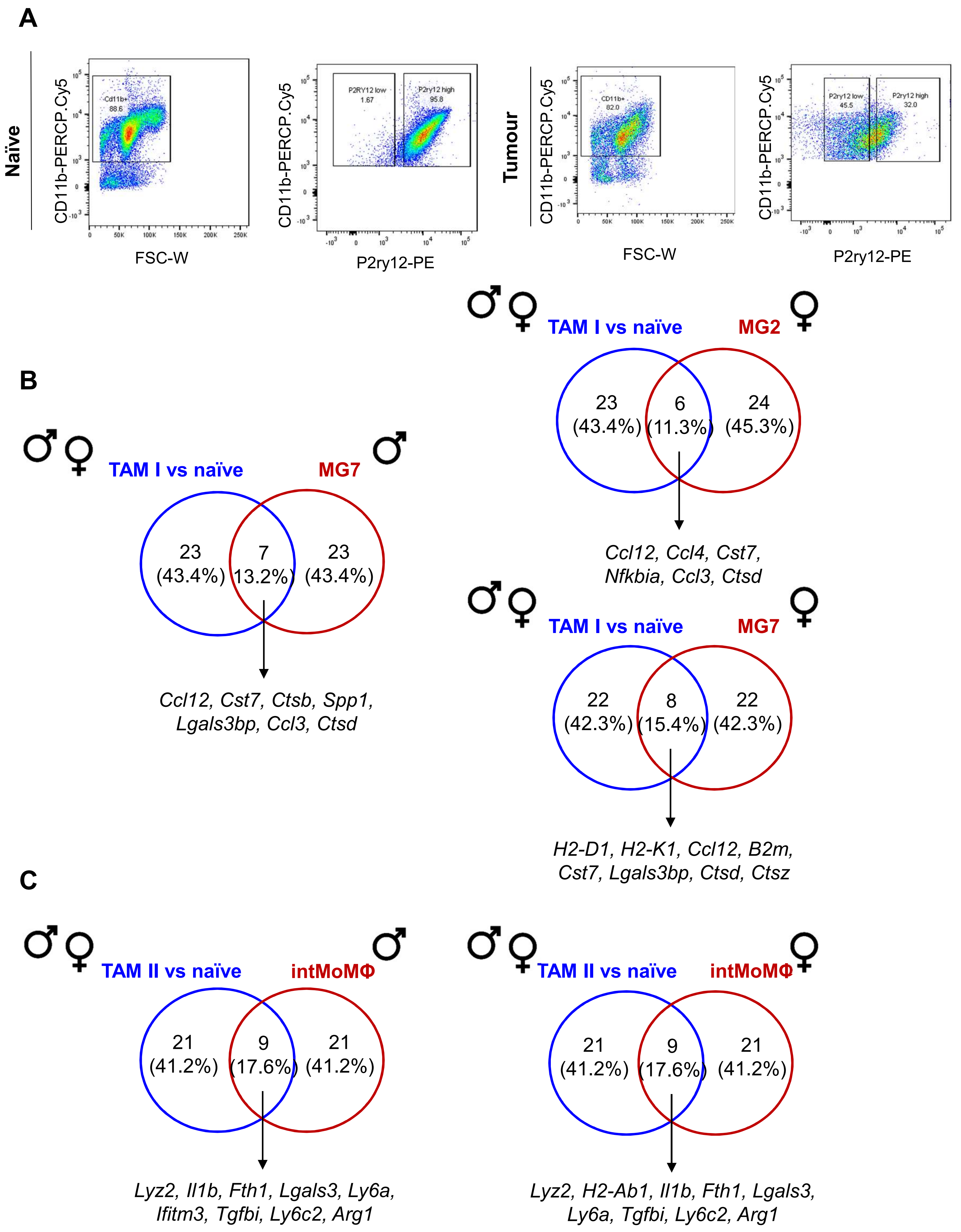

Supplementary Figure 5. Characterization of TAM I and TAM II subsets by FACS and by comparing their gene expression signatures with datasets gathered from the literature, related to figure 2.

(A) Gating strategy used to discriminate CD11b+ P2ry12+ and CD11b+ P2ry12-/low cells in naïve and syngeneic GL261 tumour-bearing mice by flow cytometry. **(B-C)** Venn diagram representations showing shared and exclusive genes with the study by Ochocka and colleagues [48] comparing **(B)** microglia-like cells and **(C)** macrophage-like cells. Comparisons are based on top 30 up-regulated genes defining TAM I, TAM II and the various myeloid clusters described in the syngeneic GL261 GBM model by Ochocka and colleagues [48] . Comparisons resulting in more than 10% overlap are represented.

Supplementary Figure 6

A

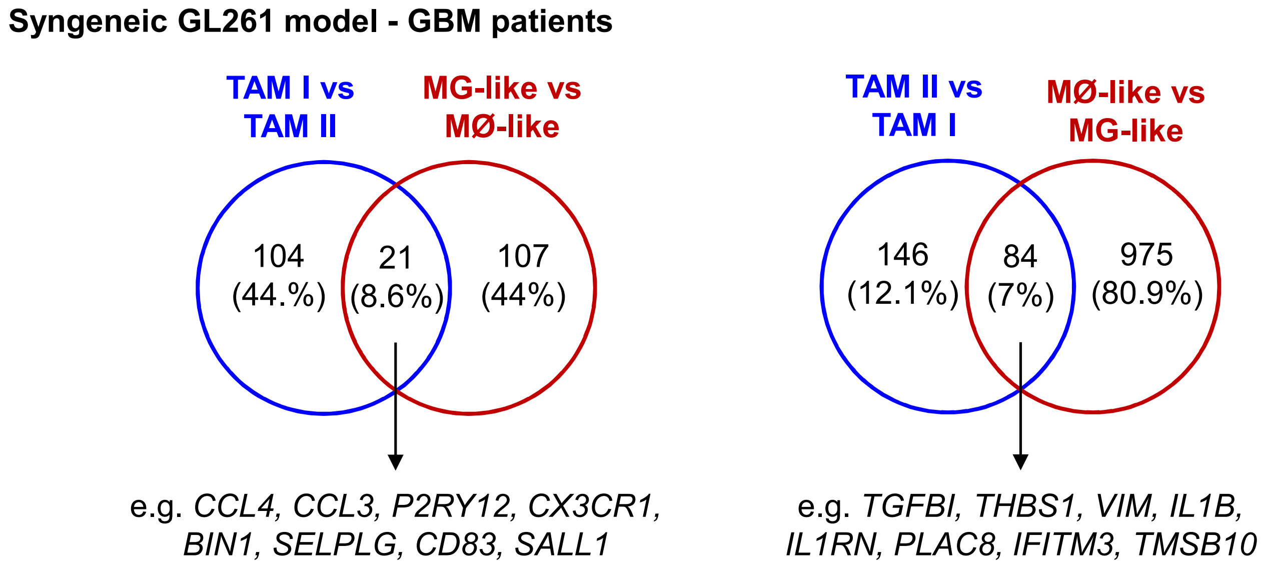

B

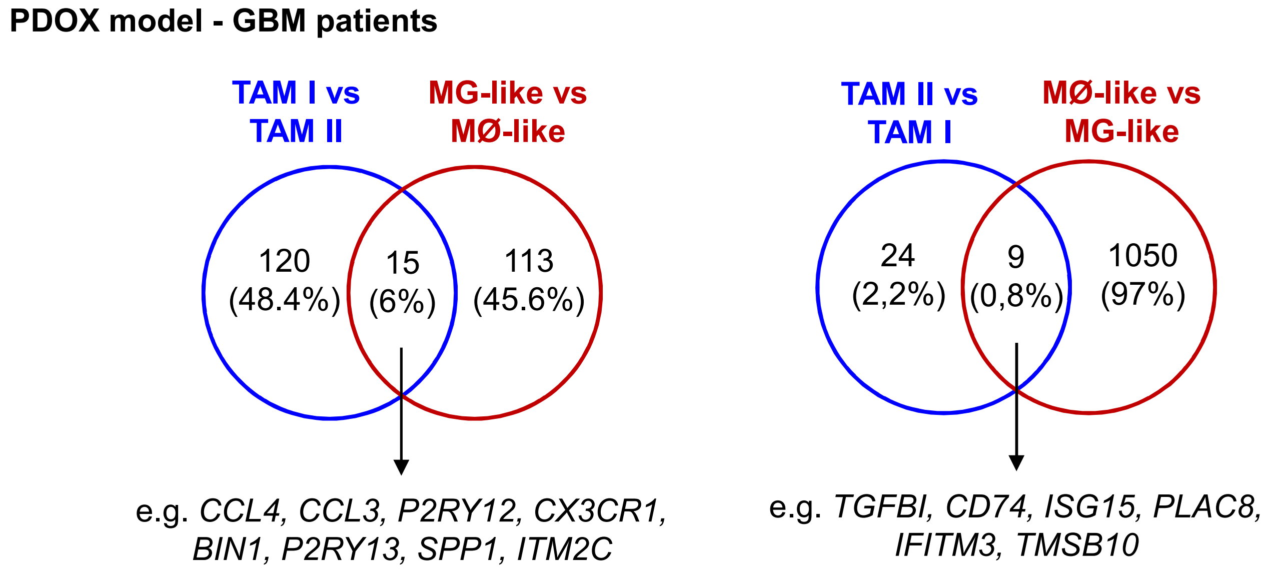

Supplementary Figure 6. Microglia- versus macrophage-like features in GBM, related to figure 2.

(A-B) Venn diagram representations showing shared and exclusive genes in microglia-like (TAM I) and macrophage-like (TAM II) cells in **(A)** syngeneic GL261 and **(B)** PDOX models (blue circles) with putative corresponding cell types described in GBM patients (red circles) from Muller and colleagues [10]. A selection of shared genes is annotated. MG: microglia; MØ: macrophage.

Supplementary Figure 7

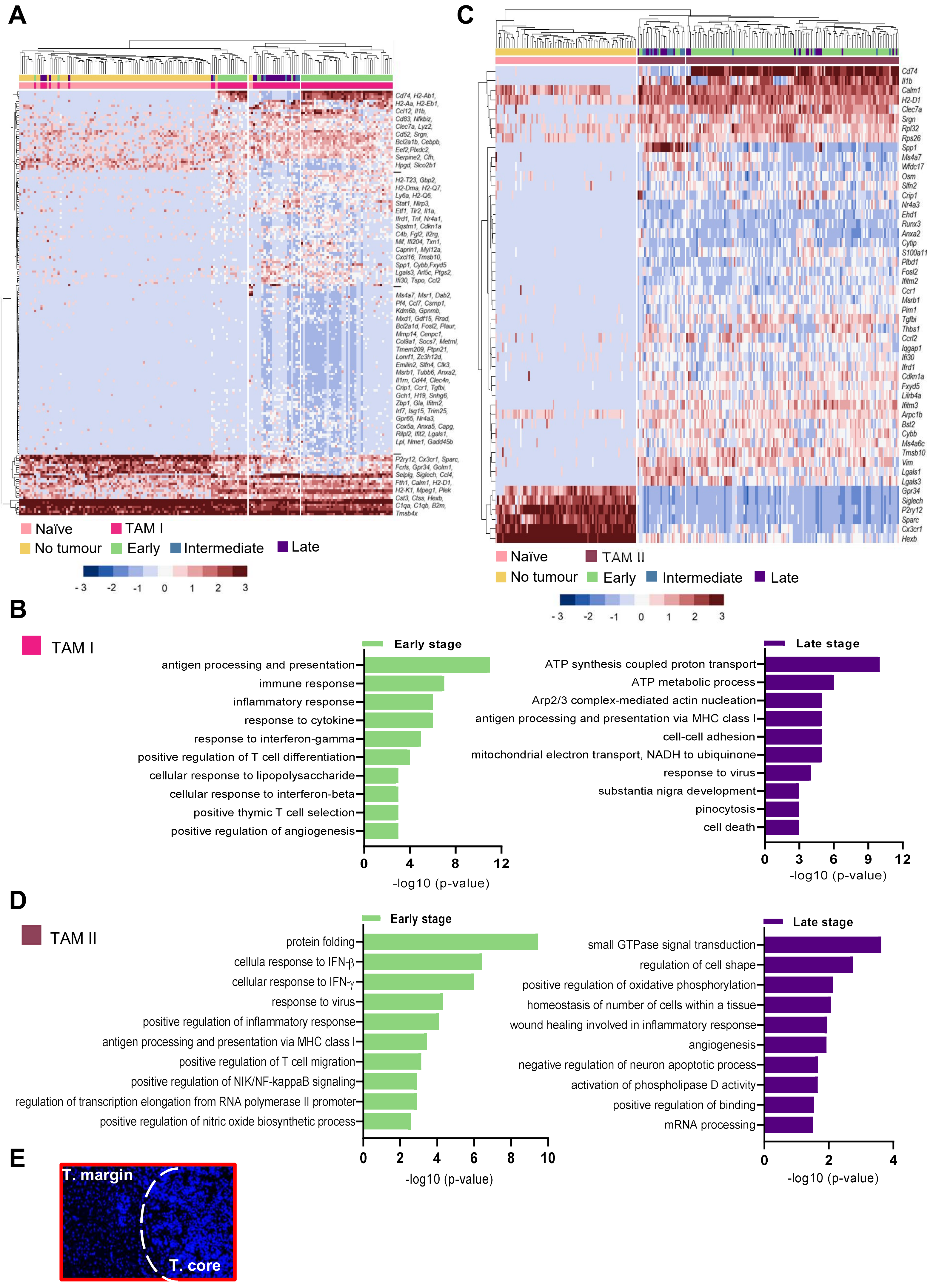

Supplementary Figure 7. Differential microglia and monocytic-derived macrophage transcriptional adaptation along GBM progression, related to figure 3.

(A) Two-way hierarchical heat-map clustering analyses of the most differentially expressed genes (p value < 0.01) in TAM I along tumour progression. Scale bar represents colour-coded z-scores. (**B)** Gene ontology terms of TAM I exclusive up-regulated genes at early (left) and late (right) GBM stages. **(C)** Two-way hierarchical heat-map clustering analyses of the most differentially expressed genes (p value < 0.01) in TAM II along tumour progression. Scale bar represents colour-coded z-scores. **(D)** Gene ontology terms of TAM II exclusive up-regulated genes at early (left) and late (right) GBM stages. **(E)** Picture representing Hoechst-stained nuclei used to discriminate tumour margin and core in mouse brain sections.

Supplementary Figure 8

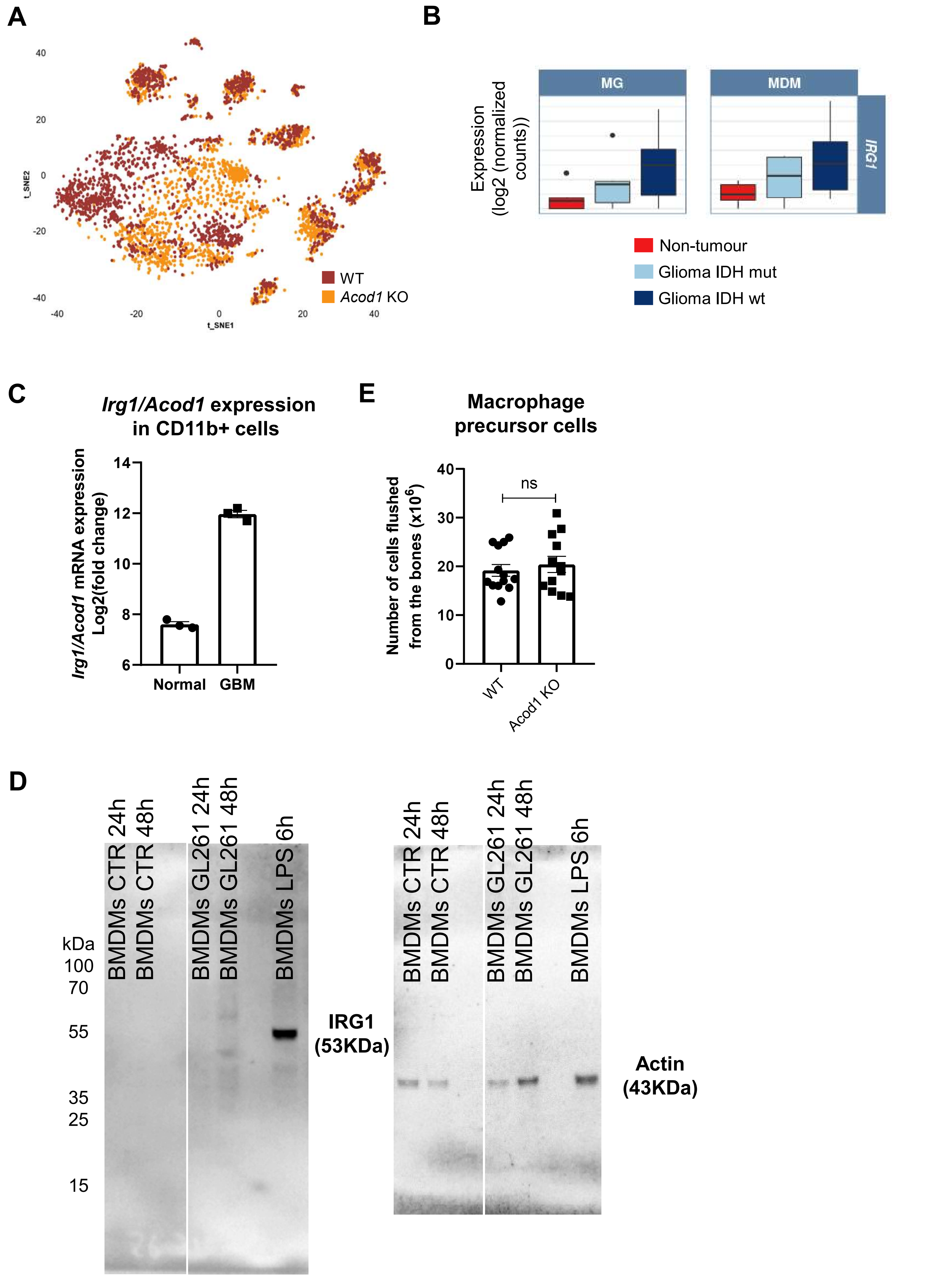

Supplementary Figure 8. IRG1/ACOD1 expression levels in TAMs, related to figure 4.

(A) 2D-tSNE representation of all single cells included in the study (n = 5’659 cells) grouped within 12 cell clusters (brown: WT; orange: *Acod1* KO). **(B)** *IRG1/ACOD1* expression in both microglia (MG) and macrophages (MDM) in GBM patients from Brain Tumor Immune Micro Environment dataset [59]. **(C)** *Irg1/Acod1* expression in CD11b+ cells isolated from naïve (n=3) and GL261-implanted mice (n=3) (E-MTAB-2660 dataset) [11]. **(D)** Western blot analysis showing IRG1/ACOD1 expression in BMDMs at baseline and in co-culture with GL261 cells for 24 and 48h. BMDMs treated with LPS (100 ng/ml) for 6h were used as positive control. **(E)** Total number of bone marrow precursor cells flushed from the legs of WT and *Acod1* KO mice. Data are not normalized and are represented as mean ± SEM (n.s. - not significant).

Supplementary Figure 9

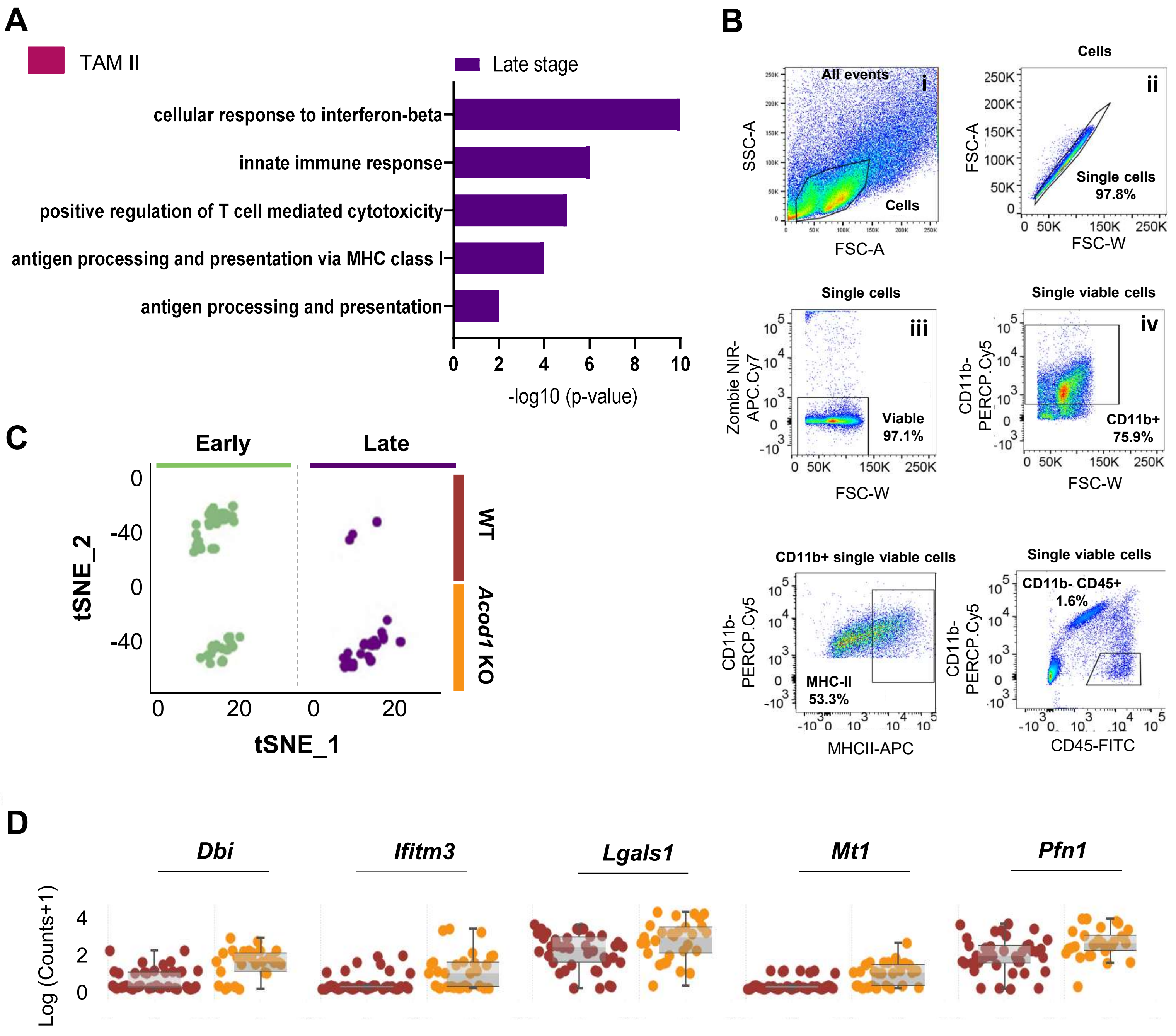

Supplementary Figure 9. TAM and lymphocytic signatures under *Acod1* deficiency, related to figure 5.

(A) Gene set enrichment analysis of TAM II uniquely up-regulated genes in Acod1 KO mice versus WT mice at late stages. **(B)** Flow cytometry gating strategy. (i) Cells of interest were gated based on forward (FSC) and side scatter (SSC). (ii) Doublets were excluded based on the forward scatter height (FSC-H) versus forward scatter area (FSC-A). (iii) Zombie NIR-APC.cy7 was used to discriminate living cells. (iv) CD11b-PERCP.cy5 was used to gate the myeloid compartment. Lastly, we gated MHC-II-expressing cells and lymphocytes (CD11b- CD45+). **(C)** 2D-tSNE representation showing the lymphocytic population detected at early and late stages of tumour development by scRNA-seq. **(D)** Notch plot representation of selected genes up-regulated by lymphocytes in *Acod1* KO mice compared with WT mice at early stage.

Supplementary Figure 10

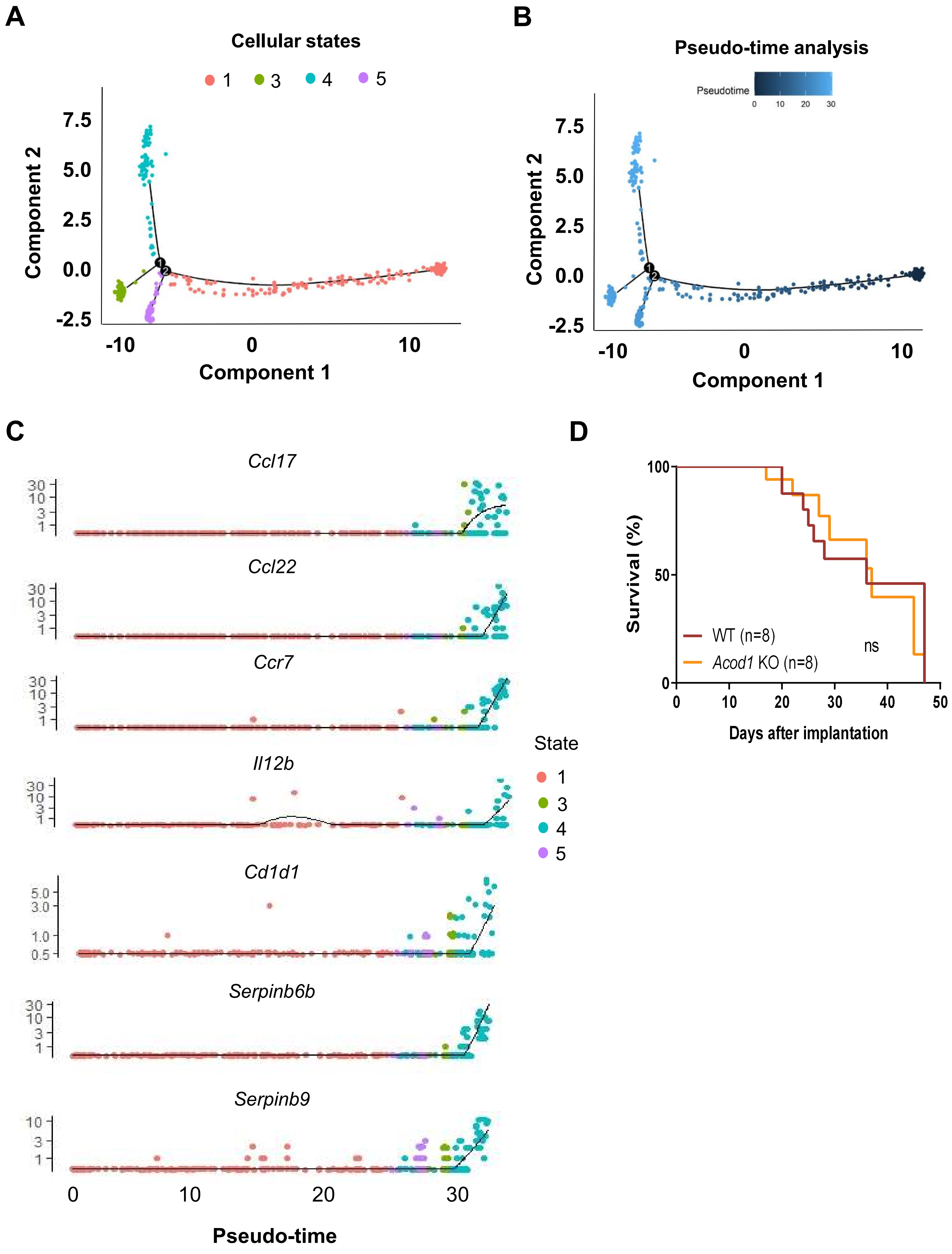

Supplementary Figure 10. TAM II cellular state diversity under *Acod1* deficiency, related to figure 5.

(A) Pseudo-time analysis of TAM II from *Acod1* KO showing four distinct cellular states in a two-dimensional state space (see Materials and Methods). **(B)** Pseudo-time analysis showing each cell along the inferred cell trajectory state. **(C)** Relative expression of exclusive genes driving the correspondent cellular state in TAM II subset under *Acod1* deficiency. (**D)** Kaplan-Meier curves showing survival rates in WT (n = 8) and *Acod1 KO* (n = 8) mice.
