# Supplementary material for: Elucidating tumour‐associated microglia/macrophage diversity along glioblastoma progression and under ACOD1 deficiency
Source: Mol Oncol. 2022 Aug 15;16(17):3167–91. doi: 10.1002/1878-0261.13287 (PMC9441003; doi:10.1002/1878-0261.13287)
Supplement: Supplementary file 2 — Table S1. Up‐regulated differentially expressed genes in tumour‐associated clusters (astrocytes, endothelial, oligodendrocytes, myeloid) versus correspondent naïve cells (p‐value < 0.01 and log2 FC > 0.5), related to Figure 1. [file MOL2-16-3167-s004.docx]

**Table S1. Up-regulated differentially expressed genes in tumour-associated clusters (astrocytes, endothelial cells, myeloid cells 1 and oligodendrocytes) versus correspondent naïve cells (p-value < 0.01 and Log2 FC > 0.5), related to figure 1.**

| Gene symbol | p-value | logFC | Cell type |
| --- | --- | --- | --- |
| *Xist* | 3.1658204431624202e-21 | 0.947022775 | Astrocytes |
| *Ifitm3* | 1.1474481062129597e-15 | 0.677613583 | Astrocytes |
| *H2-D1* | 9.820522742619489e-14 | 0.719144973 | Astrocytes |
| *B2m* | 2.1591126263125014e-10 | 1.033085604 | Astrocytes |
| *Vim* | 1.5429299982012794e-8 | 0.767820102 | Astrocytes |
| *Chst2* | 4.869372380516648e-7 | 0.560850092 | Astrocytes |
| *H2-K1* | 7.871752954048982e-7 | 0.607269337 | Astrocytes |
| *Fos* | 3.0428227111335604e-34 | 2.65499757 | Endothelial cells |
| *Jun* | 4.272030023716601e-20 | 1.726750097 | Endothelial cells |
| *Egr1* | 1.2199699765746246e-27 | 1.661625148 | Endothelial cells |
| *Junb* | 8.518318655222963e-26 | 1.628396994 | Endothelial cells |
| *Fosb* | 3.826259848797234e-33 | 1.520891558 | Endothelial cells |
| *Ifitm3* | 2.9390264079632917e-16 | 1.441143459 | Endothelial cells |
| *B2m* | 1.5464731108605614e-16 | 1.375578886 | Endothelial cells |
| *Mgp* | 1.336945236277008e-9 | 1.317219417 | Endothelial cells |
| *Lgals1* | 1.0539705534686815e-16 | 1.306877235 | Endothelial cells |
| *Cyr61* | 3.188687757056264e-20 | 1.302852227 | Endothelial cells |
| *Ubc* | 1.253831172467816e-16 | 1.226309586 | Endothelial cells |
| *H2-K1* | 6.806254481277561e-13 | 1.196811282 | Endothelial cells |
| *S100a6* | 5.7388510534183e-15 | 1.161835717 | Endothelial cells |
| *Dusp1* | 1.9276704282677199e-16 | 1.146623061 | Endothelial cells |
| *Hspa5* | 1.9385363200170873e-12 | 1.129099802 | Endothelial cells |
| *Atf3* | 9.753674269818955e-17 | 1.103690569 | Endothelial cells |
| *Hspa1a* | 6.157922381026194e-11 | 1.100499931 | Endothelial cells |
| *Vim* | 3.4672191172791153e-8 | 1.09323892 | Endothelial cells |
| *Col3a1* | 5.226507128304877e-12 | 1.086651622 | Endothelial cells |
| *Bst2* | 1.1983824012575099e-15 | 1.064353937 | Endothelial cells |
| *Igfbp7* | 7.976891171394065e-9 | 1.062671597 | Endothelial cells |
| *Zfp36* | 2.4893183614360817e-15 | 1.058139318 | Endothelial cells |
| *Apold1* | 5.090976952400756e-8 | 1.052353266 | Endothelial cells |
| *Sparc* | 8.531706770918473e-8 | 1.033715366 | Endothelial cells |
| *Cald1* | 2.1176918394749595e-7 | 1.032814321 | Endothelial cells |
| *Calr* | 8.799887120692809e-12 | 1.023232057 | Endothelial cells |
| *Btg2* | 3.493242899674608e-14 | 1.017234817 | Endothelial cells |
| *Nr4a1* | 3.7172370907240346e-20 | 0.982070623 | Endothelial cells |
| *S100a11* | 6.442388697276775e-7 | 0.970231519 | Endothelial cells |
| *Tmsb10* | 4.250374000960199e-7 | 0.956132451 | Endothelial cells |
| *H2-D1* | 1.4832437633009822e-9 | 0.941400653 | Endothelial cells |
| *Adamts1* | 1.0612450212399936e-7 | 0.93668509 | Endothelial cells |
| *Ifi27l2a* | 4.9713142922451475e-17 | 0.931695207 | Endothelial cells |
| *Tmsb4x* | 2.0065115706215883e-9 | 0.929825045 | Endothelial cells |
| *Col4a1* | 1.6186687472065814e-7 | 0.9221973 | Endothelial cells |
| *Fth1* | 5.061472340312547e-7 | 0.907072078 | Endothelial cells |
| *Anxa2* | 1.1110308007982245e-8 | 0.898262325 | Endothelial cells |
| *Ier2* | 1.520352023588159e-10 | 0.885870794 | Endothelial cells |
| *Cdkn1a* | 4.18512721703245e-10 | 0.878435893 | Endothelial cells |
| *Jund* | 2.507546855512438e-13 | 0.878047763 | Endothelial cells |
| *Spp1* | 3.843651857683122e-8 | 0.876726069 | Endothelial cells |
| *Ier3* | 7.514776403457301e-12 | 0.876673902 | Endothelial cells |
| *Rpl32* | 4.675198889336071e-8 | 0.87631326 | Endothelial cells |
| *Ppp1r15a* | 8.161167511701156e-19 | 0.875550545 | Endothelial cells |
| *Fn1* | 6.547988980830562e-7 | 0.871781909 | Endothelial cells |
| *Serpinh1* | 4.899843117812787e-8 | 0.866214103 | Endothelial cells |
| *Sat1* | 2.0955380929712588e-9 | 0.862602665 | Endothelial cells |
| *Col1a2* | 2.116018041611752e-6 | 0.824341883 | Endothelial cells |
| *Fstl1* | 2.5949206144024333e-10 | 0.819381005 | Endothelial cells |
| *Rplp1* | 7.595196756148617e-7 | 0.81323947 | Endothelial cells |
| *Hsp90b1* | 1.1743803897268763e-6 | 0.792558428 | Endothelial cells |
| *Rps26* | 6.187874839575988e-6 | 0.778870854 | Endothelial cells |
| *Ncl* | 5.183874667815194e-7 | 0.777542453 | Endothelial cells |
| *Pdia6* | 1.2853906207959565e-10 | 0.770923646 | Endothelial cells |
| *Apod* | 2.1634091256829786e-6 | 0.769075585 | Endothelial cells |
| *Hes1* | 4.360401811301791e-9 | 0.765809382 | Endothelial cells |
| *Tuba1a* | 1.2022419199956144e-7 | 0.765260579 | Endothelial cells |
| *Wdr89* | 2.4558980187512706e-7 | 0.76219489 | Endothelial cells |
| *Isg15* | 5.194499192648038e-13 | 0.743393037 | Endothelial cells |
| *Timp1* | 1.3392661267928597e-13 | 0.742549885 | Endothelial cells |
| *Marcks* | 2.4529528455748956e-7 | 0.741902845 | Endothelial cells |
| *Rplp0* | 3.158862554442211e-6 | 0.733787477 | Endothelial cells |
| *Psmb8* | 1.2617791858996336e-10 | 0.72728608 | Endothelial cells |
| *Txn1* | 9.986151695444905e-7 | 0.716703034 | Endothelial cells |
| *Ccnd1* | 8.618602541687116e-10 | 0.715529303 | Endothelial cells |
| *Ctla2a* | 0.0050391980867401445 | 0.711911026 | Endothelial cells |
| *Rps20* | 2.7078764723365797e-6 | 0.711536166 | Endothelial cells |
| *Iigp1* | 2.297195034376265e-7 | 0.709560354 | Endothelial cells |
| *Wbp5* | 1.0480852753074433e-6 | 0.704999784 | Endothelial cells |
| *Klf4* | 7.1725255459277494e-6 | 0.704853102 | Endothelial cells |
| *Rrbp1* | 7.418319426526113e-7 | 0.703247703 | Endothelial cells |
| *Phlda1* | 9.215470500653013e-15 | 0.69880322 | Endothelial cells |
| *Rps3* | 1.052413857181943e-5 | 0.696590333 | Endothelial cells |
| *Irgm1* | 1.245503013318223e-10 | 0.696238212 | Endothelial cells |
| *Anxa1* | 1.0019690237608955e-14 | 0.688581341 | Endothelial cells |
| *Bgn* | 6.995831870520038e-10 | 0.681045651 | Endothelial cells |
| *Itgb1* | 1.7279784202938427e-4 | 0.678929002 | Endothelial cells |
| *Ctsb* | 8.291208118347434e-7 | 0.678694684 | Endothelial cells |
| *Gbp2* | 2.2105847866340853e-5 | 0.675819742 | Endothelial cells |
| *Col4a2* | 3.152186322737533e-4 | 0.670416852 | Endothelial cells |
| *Ckap4* | 2.251455484661851e-11 | 0.667913911 | Endothelial cells |
| *Rpl39* | 1.5660056537588392e-5 | 0.664740642 | Endothelial cells |
| *Hspa1b* | 6.204384459061471e-8 | 0.663280723 | Endothelial cells |
| *Xist* | 5.973006914015487e-20 | 0.657941885 | Endothelial cells |
| *Cd63* | 8.767996857776142e-9 | 0.656712416 | Endothelial cells |
| *Pfn1* | 6.295289703312638e-5 | 0.650033801 | Endothelial cells |
| *Rpl4* | 1.3162775672998623e-4 | 0.639905256 | Endothelial cells |
| *Tpm1* | 6.0950231730597104e-5 | 0.636576914 | Endothelial cells |
| *Col1a1* | 1.5293690080497196e-7 | 0.633617696 | Endothelial cells |
| *Rps2* | 2.1350013166078466e-4 | 0.632733929 | Endothelial cells |
| *Tm4sf1* | 4.845775558935363e-4 | 0.630048771 | Endothelial cells |
| *Hnrnpab* | 2.0532831090211054e-5 | 0.625528607 | Endothelial cells |
| *Gpx1* | 4.543054725700441e-7 | 0.622297935 | Endothelial cells |
| *Dcn* | 2.1143704704864233e-5 | 0.61912842 | Endothelial cells |
| *Gas5* | 8.198384575260836e-6 | 0.615546874 | Endothelial cells |
| *Plk2* | 4.5333970117569066e-7 | 0.61341075 | Endothelial cells |
| *Igfbp3* | 1.782273255331839e-5 | 0.611770668 | Endothelial cells |
| *Hnrnpu* | 9.206932435437538e-6 | 0.610772703 | Endothelial cells |
| *Tpm4* | 7.901077192158158e-4 | 0.604783811 | Endothelial cells |
| *Rack1* | 2.361346760799203e-4 | 0.604772776 | Endothelial cells |
| *H2-Q7* | 4.011111219733985e-8 | 0.604566097 | Endothelial cells |
| *Tpr* | 3.7675950805861295e-6 | 0.604429449 | Endothelial cells |
| *P4hb* | 1.1252027450114287e-6 | 0.603234406 | Endothelial cells |
| *Tagln2* | 2.607972318137872e-5 | 0.597708258 | Endothelial cells |
| *Top2a* | 8.570029915516537e-13 | 0.594512367 | Endothelial cells |
| *Mif* | 3.7452812282844003e-6 | 0.593856574 | Endothelial cells |
| *sept.07* | 7.816146747847199e-5 | 0.589583769 | Endothelial cells |
| *Nid1* | 2.4959775544791684e-8 | 0.588622365 | Endothelial cells |
| *Pabpc1* | 7.572336357792248e-4 | 0.5849135 | Endothelial cells |
| *Actb* | 7.626655857551868e-4 | 0.582562237 | Endothelial cells |
| *Eef1b2* | 3.549022221305748e-5 | 0.581442282 | Endothelial cells |
| *S100a10* | 3.0752178104274427e-6 | 0.57894666 | Endothelial cells |
| *Shfm1* | 4.635034329809125e-6 | 0.578642753 | Endothelial cells |
| *Myl12a* | 2.9607551100736265e-4 | 0.578187407 | Endothelial cells |
| *Rgs16* | 3.91313142121192e-12 | 0.576390775 | Endothelial cells |
| *Gng11* | 1.2568746220225515e-4 | 0.571973794 | Endothelial cells |
| *Plat* | 4.718344970890314e-6 | 0.570581395 | Endothelial cells |
| *Gadd45b* | 8.128095162309888e-9 | 0.56988604 | Endothelial cells |
| *Krtcap2* | 5.516864902320292e-8 | 0.568766307 | Endothelial cells |
| *Psme2b* | 4.253210008291341e-8 | 0.568557124 | Endothelial cells |
| *Anp32b* | 2.0566943014595698e-5 | 0.568417355 | Endothelial cells |
| *Zfas1* | 1.9652505157252744e-11 | 0.567776655 | Endothelial cells |
| *Rps9* | 6.816862512540929e-4 | 0.563626797 | Endothelial cells |
| *Msn* | 3.142609832916596e-4 | 0.563152307 | Endothelial cells |
| *Pdia3* | 1.8873603411452815e-4 | 0.561062477 | Endothelial cells |
| *Tubb5* | 6.641708912128627e-4 | 0.559246179 | Endothelial cells |
| *Canx* | 7.833470596066036e-4 | 0.558791669 | Endothelial cells |
| *Atf4* | 5.742247984659831e-7 | 0.556197443 | Endothelial cells |
| *Cebpd* | 6.984602274366399e-7 | 0.554692493 | Endothelial cells |
| *Mmp3* | 1.645206341706406e-8 | 0.554096061 | Endothelial cells |
| *Gbp3* | 6.732407577501276e-5 | 0.553390005 | Endothelial cells |
| *Hbegf* | 2.456035663160284e-6 | 0.550977589 | Endothelial cells |
| *Psmb1* | 2.0228178752436567e-6 | 0.550284443 | Endothelial cells |
| *Akap12* | 0.004596240882850671 | 0.546837177 | Endothelial cells |
| *Lgals9* | 2.878370766794641e-7 | 0.546677207 | Endothelial cells |
| *Gbp7* | 3.434197303345539e-5 | 0.546000267 | Endothelial cells |
| *Rps5* | 8.363182100786648e-4 | 0.543050096 | Endothelial cells |
| *Prdx1* | 0.0016082912419221366 | 0.54214879 | Endothelial cells |
| *Laptm4a* | 2.57101991721105e-4 | 0.542117685 | Endothelial cells |
| *Col5a2* | 6.925144116975989e-9 | 0.539620456 | Endothelial cells |
| *Myh9* | 7.800163069516715e-5 | 0.539165443 | Endothelial cells |
| *Arf4* | 3.87105036474341e-6 | 0.539102667 | Endothelial cells |
| *Gnb1* | 9.724986219638744e-8 | 0.536764859 | Endothelial cells |
| *Tubb6* | 4.818020137290043e-11 | 0.53646938 | Endothelial cells |
| *Zfp36l1* | 4.002462150110841e-5 | 0.536464912 | Endothelial cells |
| *Klf6* | 1.281363125601531e-4 | 0.536421929 | Endothelial cells |
| *Klf2* | 2.7166157849158257e-4 | 0.536010399 | Endothelial cells |
| *Nedd4* | 8.053320975679022e-5 | 0.535719237 | Endothelial cells |
| *Ifitm2* | 2.309568473416466e-4 | 0.535289353 | Endothelial cells |
| *Rock2* | 3.617928217957266e-5 | 0.535139857 | Endothelial cells |
| *Lgals3bp* | 9.947723390516821e-12 | 0.534102522 | Endothelial cells |
| *Atpif1* | 1.7287730521004832e-5 | 0.533680791 | Endothelial cells |
| *Litaf* | 1.1626662555283612e-9 | 0.532929352 | Endothelial cells |
| *Ppic* | 4.2545406715249534e-7 | 0.531505899 | Endothelial cells |
| *Lama4* | 2.1674767710467707e-6 | 0.530649936 | Endothelial cells |
| *Ifit3* | 1.3257451937149932e-9 | 0.529642261 | Endothelial cells |
| *2410006H16Rik* | 4.472213428218854e-9 | 0.527813989 | Endothelial cells |
| *Cd63-ps* | 2.0980046644077486e-6 | 0.521142723 | Endothelial cells |
| *Hint1* | 1.0179006462211693e-4 | 0.520287587 | Endothelial cells |
| *Cd74* | 3.3333185218071807e-12 | 0.519218338 | Endothelial cells |
| *Cxcl10* | 4.181699410133659e-10 | 0.518937108 | Endothelial cells |
| *Socs3* | 1.0356973064624074e-11 | 0.518469248 | Endothelial cells |
| *Tuba1c* | 1.3223729524859259e-9 | 0.518142187 | Endothelial cells |
| *Npc2* | 4.0891465321940564e-5 | 0.517091169 | Endothelial cells |
| *Rbms1* | 8.769372794502072e-6 | 0.516723853 | Endothelial cells |
| *Rpl8* | 4.3051580612240155e-4 | 0.515042304 | Endothelial cells |
| *Ptgs2* | 5.488154129506169e-10 | 0.513073049 | Endothelial cells |
| *Ifitm1* | 9.013702899384846e-7 | 0.513025615 | Endothelial cells |
| *Lmna* | 1.5758271426209467e-6 | 0.507935011 | Endothelial cells |
| *Selenof* | 1.0070708103706379e-4 | 0.50778021 | Endothelial cells |
| *Rhoc* | 3.0996831698975714e-5 | 0.506286982 | Endothelial cells |
| *Mmp14* | 7.524361930683186e-8 | 0.50478086 | Endothelial cells |
| *Gbp4* | 2.0483641957177895e-4 | 0.503005018 | Endothelial cells |
| *Cebpb* | 3.591135570537019e-7 | 0.501965867 | Endothelial cells |
| *Dbi* | 6.187411334099987e-4 | 0.500981755 | Endothelial cells |
| *Rpsa-ps10* | 3.695187687385306e-4 | 0.500806502 | Endothelial cells |
| *Ier5* | 2.9889726571487584e-6 | 0.500419967 | Endothelial cells |
| *Cxcl9* | 8.646390754850696e-8 | 0.5004082 | Endothelial cells |
| *Cd74* | 1.4050998142594664e-86 | 4.53017349 | Myeloid cells 1 |
| *H2-Ab1* | 6.567632677664073e-81 | 4.029010413 | Myeloid cells 1 |
| *H2-Aa* | 2.5270057650487195e-81 | 3.723362725 | Myeloid cells 1 |
| *H2-Eb1* | 1.1775596587587698e-79 | 3.654464025 | Myeloid cells 1 |
| *Lyz2* | 1.1771327235947642e-58 | 3.617541924 | Myeloid cells 1 |
| *H2-K1* | 7.90572625797621e-93 | 2.93229588 | Myeloid cells 1 |
| *H2-D1* | 2.6921807634688864e-91 | 2.914305134 | Myeloid cells 1 |
| *Il1b* | 3.124514249417684e-58 | 2.888165878 | Myeloid cells 1 |
| *Fth1* | 1.40351575618166e-72 | 2.85742628 | Myeloid cells 1 |
| *Apoe* | 1.535066161137984e-27 | 2.513456176 | Myeloid cells 1 |
| *Clec7a* | 4.7813905543520916e-60 | 2.422755992 | Myeloid cells 1 |
| *Srgn* | 9.987424654475959e-72 | 2.355036274 | Myeloid cells 1 |
| *Calm1* | 4.751862691786506e-66 | 2.309203115 | Myeloid cells 1 |
| *B2m* | 5.273959765191587e-83 | 2.270939726 | Myeloid cells 1 |
| *Cd52* | 2.8209672637910824e-68 | 2.119938856 | Myeloid cells 1 |
| *Ifitm3* | 2.026307583751523e-69 | 2.032357233 | Myeloid cells 1 |
| *Vim* | 6.154756601774954e-66 | 1.952639822 | Myeloid cells 1 |
| *Ifi27l2a* | 1.5045325084024788e-62 | 1.892491276 | Myeloid cells 1 |
| *Ly6a* | 1.135972184864838e-56 | 1.86416209 | Myeloid cells 1 |
| *Rpl32* | 6.71889657615995e-64 | 1.827252856 | Myeloid cells 1 |
| *Cebpb* | 8.59420080109086e-54 | 1.726791222 | Myeloid cells 1 |
| *Cd14* | 4.698597180607599e-35 | 1.72493965 | Myeloid cells 1 |
| *Rps20* | 1.6807655599498945e-65 | 1.713557757 | Myeloid cells 1 |
| *Ctsc* | 5.187624787408037e-47 | 1.679366015 | Myeloid cells 1 |
| *Lgals3* | 3.582436335909289e-53 | 1.677803707 | Myeloid cells 1 |
| *Cdkn1a* | 2.0428310919036314e-57 | 1.676358009 | Myeloid cells 1 |
| *Npc2* | 2.162149554934563e-50 | 1.648990769 | Myeloid cells 1 |
| *Rps26* | 2.238930670775144e-58 | 1.600077045 | Myeloid cells 1 |
| *Txn1* | 4.985204694729257e-58 | 1.586701022 | Myeloid cells 1 |
| *Lilrb4a* | 5.964438540614418e-55 | 1.575449012 | Myeloid cells 1 |
| *Psmb8* | 2.7850300691100695e-59 | 1.566916104 | Myeloid cells 1 |
| *Rps3* | 1.7757826919048797e-61 | 1.566277579 | Myeloid cells 1 |
| *Tmsb10* | 2.0655628944827132e-50 | 1.539624335 | Myeloid cells 1 |
| *Rps5* | 8.846235334067114e-55 | 1.528040343 | Myeloid cells 1 |
| *Prdx5* | 2.352463192664843e-45 | 1.518185305 | Myeloid cells 1 |
| *Lgals1* | 1.2139868016503542e-53 | 1.502358363 | Myeloid cells 1 |
| *Rpl4* | 8.234331566275191e-54 | 1.479391453 | Myeloid cells 1 |
| *Tmsb4x* | 1.3553015412885812e-54 | 1.460975072 | Myeloid cells 1 |
| *Gpx1* | 4.314203581572502e-50 | 1.457797812 | Myeloid cells 1 |
| *AW112010* | 1.031300501432891e-44 | 1.442696949 | Myeloid cells 1 |
| *Bst2* | 1.2322939179429388e-53 | 1.437005467 | Myeloid cells 1 |
| *Fxyd5* | 3.3609289667118406e-55 | 1.436891953 | Myeloid cells 1 |
| *Tgfbi* | 6.208646753627593e-40 | 1.434791283 | Myeloid cells 1 |
| *Rps9* | 1.414757905158856e-56 | 1.433800262 | Myeloid cells 1 |
| *Ccrl2* | 1.0607356627673125e-44 | 1.431578893 | Myeloid cells 1 |
| *Gm9794* | 3.235090704777343e-50 | 1.417527839 | Myeloid cells 1 |
| *Cstb* | 3.581889777112769e-46 | 1.410659323 | Myeloid cells 1 |
| *Cybb* | 5.582069986176802e-49 | 1.409556154 | Myeloid cells 1 |
| *Rpl8* | 8.582826773913067e-51 | 1.406864454 | Myeloid cells 1 |
| *Rplp1* | 2.1445653685556968e-48 | 1.391469739 | Myeloid cells 1 |
| *Gm6977* | 6.691262971558613e-33 | 1.382843508 | Myeloid cells 1 |
| *Rps14* | 1.6866471706867743e-46 | 1.365753839 | Myeloid cells 1 |
| *Arpc1b* | 5.493033041254037e-49 | 1.348775483 | Myeloid cells 1 |
| *Bcl2a1b* | 2.0756424621627622e-43 | 1.34875136 | Myeloid cells 1 |
| *Ncl* | 4.288488336398002e-44 | 1.344790815 | Myeloid cells 1 |
| *Wdr89* | 1.5112304503881962e-45 | 1.334691302 | Myeloid cells 1 |
| *Rack1* | 1.3018054013793263e-47 | 1.330696317 | Myeloid cells 1 |
| *Ybx1* | 2.4666465765509797e-43 | 1.329733296 | Myeloid cells 1 |
| *Rplp0* | 7.435728042917132e-49 | 1.328376665 | Myeloid cells 1 |
| *Hsp90ab1* | 1.8123246661689078e-40 | 1.316937182 | Myeloid cells 1 |
| *Nfkbia* | 4.06393541872909e-22 | 1.308976633 | Myeloid cells 1 |
| *Rpl39* | 7.470097017864866e-48 | 1.3062983 | Myeloid cells 1 |
| *Ptgs2* | 1.702391216051156e-39 | 1.305662793 | Myeloid cells 1 |
| *Gbp2* | 1.5482480744335516e-46 | 1.304708887 | Myeloid cells 1 |
| *Ms4a6c* | 1.063241092508019e-42 | 1.286842595 | Myeloid cells 1 |
| *H2-Q7* | 2.9700417050567214e-54 | 1.264725416 | Myeloid cells 1 |
| *Rps19* | 3.222964207689374e-45 | 1.255486256 | Myeloid cells 1 |
| *Ly6e* | 8.782027534515674e-39 | 1.249722907 | Myeloid cells 1 |
| *Ifi30* | 1.0211107197694653e-46 | 1.23180337 | Myeloid cells 1 |
| *Rps2* | 2.0486138893276283e-45 | 1.231548492 | Myeloid cells 1 |
| *Crip1* | 1.0044209166102008e-39 | 1.22842221 | Myeloid cells 1 |
| *Rps24* | 2.844920189499767e-42 | 1.21983282 | Myeloid cells 1 |
| *Ifrd1* | 6.257410925974726e-44 | 1.207979836 | Myeloid cells 1 |
| *Pkm* | 5.359019936757965e-39 | 1.198274009 | Myeloid cells 1 |
| *Shfm1* | 4.1857408623947154e-43 | 1.197412062 | Myeloid cells 1 |
| *AA467197* | 1.4722037294459038e-33 | 1.186464404 | Myeloid cells 1 |
| *Rpl14* | 4.715554672081356e-45 | 1.1840035 | Myeloid cells 1 |
| *Arg1* | 1.493628723168825e-19 | 1.181564624 | Myeloid cells 1 |
| *Cd83* | 8.66770876444529e-33 | 1.170864868 | Myeloid cells 1 |
| *Prdx1* | 1.4369589892703414e-33 | 1.164788076 | Myeloid cells 1 |
| *Rpl22* | 8.950225230482753e-39 | 1.164162958 | Myeloid cells 1 |
| *Sh3bgrl3* | 1.4148669833150089e-37 | 1.154473897 | Myeloid cells 1 |
| *H2-DMa* | 8.15379478801163e-37 | 1.149769166 | Myeloid cells 1 |
| *Cxcl2* | 7.236623510788333e-21 | 1.148836745 | Myeloid cells 1 |
| *Pfn1* | 4.233841965839157e-35 | 1.146741842 | Myeloid cells 1 |
| *Slfn2* | 1.2715482839975322e-40 | 1.139283065 | Myeloid cells 1 |
| *Pabpc1* | 6.790558218007202e-38 | 1.131825964 | Myeloid cells 1 |
| *Cyba* | 1.8378883812522527e-33 | 1.130651566 | Myeloid cells 1 |
| *Psme2b* | 3.974451874615079e-39 | 1.122457801 | Myeloid cells 1 |
| *Cxcl9* | 6.589938178826631e-28 | 1.117263113 | Myeloid cells 1 |
| *Anp32b* | 1.7907306776611792e-39 | 1.112791901 | Myeloid cells 1 |
| *Rpl26* | 2.1605180469764933e-40 | 1.111597113 | Myeloid cells 1 |
| *Tspo* | 1.9899478163066882e-44 | 1.108851373 | Myeloid cells 1 |
| *Hspa5* | 4.866904015310046e-26 | 1.106102609 | Myeloid cells 1 |
| *Stat1* | 3.521572738122852e-45 | 1.097472667 | Myeloid cells 1 |
| *Cxcl16* | 3.3108259987729014e-43 | 1.088322974 | Myeloid cells 1 |
| *Rpl41* | 1.3968116930001714e-34 | 1.086943938 | Myeloid cells 1 |
| *Pim1* | 3.8926887895619406e-47 | 1.085359471 | Myeloid cells 1 |
| *Atox1* | 2.1677565896812323e-38 | 1.084045154 | Myeloid cells 1 |
| *Rps10-ps1* | 1.6545306235306064e-39 | 1.08385498 | Myeloid cells 1 |
| *Fgl2* | 1.2580402482472646e-41 | 1.083552815 | Myeloid cells 1 |
| *Ifi204* | 5.5863538782460915e-43 | 1.073501317 | Myeloid cells 1 |
| *Ldha* | 4.9950483813071396e-40 | 1.073476616 | Myeloid cells 1 |
| *Rpl23* | 1.3469880421403585e-40 | 1.071271125 | Myeloid cells 1 |
| *Gm5963* | 5.90032050387129e-36 | 1.064141952 | Myeloid cells 1 |
| *Mif* | 3.0957376663122374e-37 | 1.061778689 | Myeloid cells 1 |
| *Rps24-ps3* | 5.74955278127184e-35 | 1.057346798 | Myeloid cells 1 |
| *Rps15* | 1.463772617444673e-34 | 1.053969965 | Myeloid cells 1 |
| *Arpc2* | 8.829261590919203e-33 | 1.049462482 | Myeloid cells 1 |
| *Iqgap1* | 9.251247939945173e-40 | 1.047074632 | Myeloid cells 1 |
| *Rps15a* | 3.4185095633426345e-36 | 1.042302943 | Myeloid cells 1 |
| *Mcl1* | 8.764269998169597e-33 | 1.034676406 | Myeloid cells 1 |
| *Msr1* | 1.618993268635828e-33 | 1.02612067 | Myeloid cells 1 |
| *Cox6a1* | 8.006277789319446e-36 | 1.022841333 | Myeloid cells 1 |
| *Gm16580* | 7.673167944666215e-33 | 1.022313622 | Myeloid cells 1 |
| *Cox5a* | 1.250365316021082e-37 | 1.017477327 | Myeloid cells 1 |
| *Gm5905* | 1.6492305850064338e-34 | 1.014355581 | Myeloid cells 1 |
| *Gm9843* | 7.450990906795134e-32 | 1.012632657 | Myeloid cells 1 |
| *Rps16-ps2* | 1.0759085480975715e-34 | 1.012319732 | Myeloid cells 1 |
| *Rps13-ps2* | 8.512593966745947e-33 | 1.007846773 | Myeloid cells 1 |
| *Aldoa* | 8.699836649941135e-31 | 1.004388715 | Myeloid cells 1 |
| *Cox4i1* | 1.0280593668155361e-29 | 1.003214759 | Myeloid cells 1 |
| *Gm10288* | 1.2046763868422208e-34 | 1.002314839 | Myeloid cells 1 |
| *Plek* | 6.710086335763385e-24 | 1.000263707 | Myeloid cells 1 |
| *Actb* | 1.0248877577412908e-33 | 0.998544123 | Myeloid cells 1 |
| *Thbs1* | 1.6189797294444157e-27 | 0.995680328 | Myeloid cells 1 |
| *Rpsa-ps10* | 7.1465718589614e-35 | 0.995483807 | Myeloid cells 1 |
| *Fcgr2b* | 4.3062919102229386e-24 | 0.995404483 | Myeloid cells 1 |
| *App* | 1.544910416458949e-27 | 0.98910281 | Myeloid cells 1 |
| *S100a11* | 7.163139435494424e-37 | 0.986915681 | Myeloid cells 1 |
| *Ccl5* | 5.0034225537872415e-025 | 0.983602208 | Myeloid cells 1 |
| *Cox6b1* | 4.551955758338924e-31 | 0.982698558 | Myeloid cells 1 |
| *H2-DMb1* | 2.3303763771092663e-35 | 0.975639382 | Myeloid cells 1 |
| *Ccl6* | 1.503815958253823e-16 | 0.975057452 | Myeloid cells 1 |
| *Myl12a* | 1.2869138278198042e-36 | 0.974877573 | Myeloid cells 1 |
| *Nampt* | 5.314811289528195e-38 | 0.970801551 | Myeloid cells 1 |
| *Ly6c2* | 1.2526401555402308e-23 | 0.959382656 | Myeloid cells 1 |
| *Anxa5* | 8.842903812489756e-35 | 0.956982056 | Myeloid cells 1 |
| *Serbp1* | 4.728914825851608e-27 | 0.953937818 | Myeloid cells 1 |
| *Ccl12* | 4.352164618676179e-18 | 0.94729037 | Myeloid cells 1 |
| *Psma7* | 1.1540558451184754e-27 | 0.945771979 | Myeloid cells 1 |
| *Fcer1g* | 2.1644637803182218e-23 | 0.943633668 | Myeloid cells 1 |
| *H2-T23* | 1.1821205686601029e-31 | 0.938465342 | Myeloid cells 1 |
| *Psap* | 2.9060312036887834e-22 | 0.936367177 | Myeloid cells 1 |
| *Gm11478* | 3.7611801610775726e-27 | 0.933994416 | Myeloid cells 1 |
| *Akr1a1* | 2.0444979122093033e-27 | 0.933665556 | Myeloid cells 1 |
| *Psme1* | 1.5691517653286768e-28 | 0.931738237 | Myeloid cells 1 |
| *Rpl14-ps1* | 3.0749414813738124e-27 | 0.927159096 | Myeloid cells 1 |
| *Rps27l* | 1.8872163118430182e-33 | 0.922564603 | Myeloid cells 1 |
| *Atp5e* | 5.949314943035221e-34 | 0.920052208 | Myeloid cells 1 |
| *Tagln2* | 1.289154129743598e-35 | 0.919526718 | Myeloid cells 1 |
| *Sdcbp* | 5.148048698225118e-30 | 0.917685583 | Myeloid cells 1 |
| *Rpl34* | 1.1853617503900864e-30 | 0.916653317 | Myeloid cells 1 |
| *Samhd1* | 3.037469244071419e-29 | 0.914291214 | Myeloid cells 1 |
| *Eef1b2* | 4.2698396339395596e-29 | 0.914288169 | Myeloid cells 1 |
| *Gm4332* | 1.4846484877296376e-31 | 0.913309678 | Myeloid cells 1 |
| *Rps18* | 1.128063842599257e-30 | 0.906037137 | Myeloid cells 1 |
| *Lgals3bp* | 2.4410838594604566e-28 | 0.90218762 | Myeloid cells 1 |
| *Tgm2* | 6.041210225021305e-33 | 0.900250011 | Myeloid cells 1 |
| *Rpl37rt* | 1.0511528302870186e-27 | 0.899601805 | Myeloid cells 1 |
| *Actr3* | 8.537740147434311e-30 | 0.895317385 | Myeloid cells 1 |
| *Tnfaip2* | 4.4930210039413584e-29 | 0.894138593 | Myeloid cells 1 |
| *Psmb9* | 1.804344377390202e-36 | 0.892939675 | Myeloid cells 1 |
| *Dab2* | 1.1281096307076124e-26 | 0.892225784 | Myeloid cells 1 |
| *Nfe2l2* | 1.356660423257386e-23 | 0.887473423 | Myeloid cells 1 |
| *Rpl3-ps1* | 7.175128955317498e-25 | 0.88698386 | Myeloid cells 1 |
| *Spp1* | 5.239525626683955e-19 | 0.884691437 | Myeloid cells 1 |
| *Plbd1* | 1.6942595349784405e-33 | 0.883860836 | Myeloid cells 1 |
| *Gm5805* | 3.32928285555474e-26 | 0.880272897 | Myeloid cells 1 |
| *Hnrnpab* | 3.43548282944383e-28 | 0.877961638 | Myeloid cells 1 |
| *Cfp* | 1.1413327548175106e-29 | 0.876956836 | Myeloid cells 1 |
| *Cotl1* | 2.1809044872425725e-23 | 0.87399598 | Myeloid cells 1 |
| *Plac8* | 3.7832198949845197e-23 | 0.873155911 | Myeloid cells 1 |
| *Cox6c* | 5.360507859762348e-27 | 0.871630262 | Myeloid cells 1 |
| *Cxcl10* | 8.860880269714295e-23 | 0.871066936 | Myeloid cells 1 |
| *Hint1* | 2.7554822994969002e-30 | 0.870895745 | Myeloid cells 1 |
| *Cox8a* | 3.5567318498245596e-24 | 0.868088424 | Myeloid cells 1 |
| *Rps11* | 1.7686077683135276e-25 | 0.867430174 | Myeloid cells 1 |
| *Nfkbiz* | 1.652475993952648e-20 | 0.864883286 | Myeloid cells 1 |
| *Cox7b* | 2.3024440564700572e-27 | 0.86413008 | Myeloid cells 1 |
| *Gm14303* | 1.3804971019238213e-24 | 0.864070603 | Myeloid cells 1 |
| *Gm6030* | 2.3229579126170392e-35 | 0.862128296 | Myeloid cells 1 |
| *Rpl35a* | 1.2134215996018267e-24 | 0.862023333 | Myeloid cells 1 |
| *Rpl31-ps8* | 1.330305812557618e-28 | 0.861490592 | Myeloid cells 1 |
| *Eif3a* | 1.0779254905704422e-23 | 0.857452347 | Myeloid cells 1 |
| *Ctsb* | 6.9960225881065215e-12 | 0.856832531 | Myeloid cells 1 |
| *Npm1* | 6.312091551998613e-29 | 0.849784566 | Myeloid cells 1 |
| *Wfdc17* | 3.851638034893894e-25 | 0.836909468 | Myeloid cells 1 |
| *Sh3bgrl* | 6.819821887665371e-32 | 0.83447378 | Myeloid cells 1 |
| *Il2rg* | 9.72007156445771e-39 | 0.834336866 | Myeloid cells 1 |
| *Tpm4* | 4.450835041288182e-34 | 0.831816787 | Myeloid cells 1 |
| *Arpc5* | 2.5083567742638232e-23 | 0.831076315 | Myeloid cells 1 |
| *Gm10275* | 4.061625960610231e-24 | 0.827244515 | Myeloid cells 1 |
| *Sub1* | 1.3098838348534955e-29 | 0.82614218 | Myeloid cells 1 |
| *Il1rn* | 3.060309570961212e-25 | 0.820217044 | Myeloid cells 1 |
| *Tpr* | 5.928188519594232e-24 | 0.820017625 | Myeloid cells 1 |
| *2010107E04Rik* | 7.554738029338575e-30 | 0.816876833 | Myeloid cells 1 |
| *Eef2* | 1.449811084022181e-27 | 0.814558276 | Myeloid cells 1 |
| *Ccl2* | 4.70674419883347e-19 | 0.812971816 | Myeloid cells 1 |
| *Gm15427* | 2.2949789357444344e-23 | 0.809903177 | Myeloid cells 1 |
| *Ctsz* | 1.7275895585739973e-17 | 0.809788168 | Myeloid cells 1 |
| *Gm5835* | 6.364453735537242e-28 | 0.809382863 | Myeloid cells 1 |
| *Rps8* | 2.0329361484782834e-26 | 0.808930736 | Myeloid cells 1 |
| *Gadd45b* | 1.6735399168878465e-29 | 0.808565449 | Myeloid cells 1 |
| *Psmb1* | 7.303780186299398e-27 | 0.807747336 | Myeloid cells 1 |
| *Fos* | 2.772334255055987e-11 | 0.803059469 | Myeloid cells 1 |
| *Ccr1* | 1.1253475442294052e-28 | 0.802622527 | Myeloid cells 1 |
| *Chchd2* | 1.085928399072751e-22 | 0.802375136 | Myeloid cells 1 |
| *Isg15* | 8.227545521197512e-32 | 0.801401891 | Myeloid cells 1 |
| *Clec4n* | 5.532320175145543e-25 | 0.800958162 | Myeloid cells 1 |
| *Lcp1* | 6.263420871907087e-21 | 0.799643562 | Myeloid cells 1 |
| *Rps21* | 2.7599002472738147e-21 | 0.794640062 | Myeloid cells 1 |
| *Gbp7* | 5.148435474281492e-20 | 0.793892447 | Myeloid cells 1 |
| *Mndal* | 5.391477687765743e-31 | 0.791350805 | Myeloid cells 1 |
| *Ptprc* | 1.9968682371991977e-20 | 0.787879431 | Myeloid cells 1 |
| *Fosb* | 2.79728296998182e-18 | 0.78679152 | Myeloid cells 1 |
| *Junb* | 5.104384525915671e-12 | 0.782808131 | Myeloid cells 1 |
| *Gm9385* | 1.341513669262312e-21 | 0.772155077 | Myeloid cells 1 |
| *Gm14586* | 1.6034034506723168e-22 | 0.771965998 | Myeloid cells 1 |
| *Capg* | 6.444692938678501e-28 | 0.771896369 | Myeloid cells 1 |
| *Gabarap* | 6.628968640635645e-22 | 0.770923911 | Myeloid cells 1 |
| *Rps26-ps1* | 1.302508892758557e-26 | 0.770556343 | Myeloid cells 1 |
| *Msrb1* | 2.085461587481757e-30 | 0.767011526 | Myeloid cells 1 |
| *Emp3* | 4.700809397005768e-29 | 0.766123919 | Myeloid cells 1 |
| *Dek* | 1.8464645529652974e-19 | 0.766061982 | Myeloid cells 1 |
| *Psma2* | 3.535798603739104e-27 | 0.765206583 | Myeloid cells 1 |
| *Rps10* | 3.556479916889996e-24 | 0.763407602 | Myeloid cells 1 |
| *Tpi1* | 4.900451486634967e-25 | 0.762177174 | Myeloid cells 1 |
| *Fcgr4* | 4.536908737000183e-28 | 0.760642885 | Myeloid cells 1 |
| *Nr4a1* | 6.822931886429848e-27 | 0.758136165 | Myeloid cells 1 |
| *Slc25a3* | 1.011904411409721e-24 | 0.756973418 | Myeloid cells 1 |
| *Vegfa* | 1.9385920232939056e-26 | 0.754678826 | Myeloid cells 1 |
| *Eef1a1* | 1.3203199324831846e-18 | 0.754023983 | Myeloid cells 1 |
| *Rps3a1* | 1.0394696484074611e-19 | 0.750507114 | Myeloid cells 1 |
| *Rpl36al* | 2.2642442410488992e-26 | 0.748653235 | Myeloid cells 1 |
| *Ifitm2* | 5.956673821581223e-30 | 0.747748393 | Myeloid cells 1 |
| *Slc25a5* | 1.3901892279487856e-17 | 0.745948469 | Myeloid cells 1 |
| *Arhgdib* | 1.6255365862334774e-20 | 0.745331702 | Myeloid cells 1 |
| *Ms4a4c* | 2.1429274095080295e-27 | 0.744343796 | Myeloid cells 1 |
| *S100a10* | 1.7469737667347527e-29 | 0.740358823 | Myeloid cells 1 |
| *Tiparp* | 5.823785077968717e-27 | 0.738979226 | Myeloid cells 1 |
| *Lrrfip1* | 1.043465948587555e-26 | 0.738354764 | Myeloid cells 1 |
| *Xist* | 7.096006335246285e-25 | 0.737278335 | Myeloid cells 1 |
| *Nr4a3* | 3.3975661568189985e-27 | 0.736666414 | Myeloid cells 1 |
| *Osm* | 1.2118347345876117e-22 | 0.735895059 | Myeloid cells 1 |
| *Gm4204* | 6.91660206869346e-27 | 0.735299839 | Myeloid cells 1 |
| *Cox7a2* | 4.92643725639087e-21 | 0.734526074 | Myeloid cells 1 |
| *Chd4* | 1.8675491405737134e-20 | 0.730613413 | Myeloid cells 1 |
| *Rps25-ps1* | 1.621963447988527e-20 | 0.729571298 | Myeloid cells 1 |
| *Gm8995* | 1.8291333718894306e-21 | 0.726296219 | Myeloid cells 1 |
| *Pdia3* | 1.2292955718986293e-14 | 0.725895834 | Myeloid cells 1 |
| *Tubb5* | 3.432097412279972e-19 | 0.725125049 | Myeloid cells 1 |
| *Dbi* | 4.609183253587831e-21 | 0.722776298 | Myeloid cells 1 |
| *Calr* | 8.468726743943979e-15 | 0.721360301 | Myeloid cells 1 |
| *Gatm* | 1.2058885695646914e-18 | 0.718624374 | Myeloid cells 1 |
| *Litaf* | 3.9748895559093746e-26 | 0.718027438 | Myeloid cells 1 |
| *Id2* | 6.341954600269342e-16 | 0.717166114 | Myeloid cells 1 |
| *Gbp3* | 6.912188497143024e-27 | 0.71582024 | Myeloid cells 1 |
| *Rpl36a-ps2* | 1.649247901592055e-22 | 0.715260833 | Myeloid cells 1 |
| *Atp5b* | 6.801428911497304e-21 | 0.714598383 | Myeloid cells 1 |
| *Psme2* | 1.0082977257106887e-23 | 0.712815193 | Myeloid cells 1 |
| *Bcl2a1a* | 6.851890533759369e-26 | 0.708903133 | Myeloid cells 1 |
| *Ccr2* | 2.6700546534246096e-20 | 0.706726722 | Myeloid cells 1 |
| *Iigp1* | 1.688385736343041e-25 | 0.706175507 | Myeloid cells 1 |
| *Hnrnpu* | 8.006799139116972e-18 | 0.705543138 | Myeloid cells 1 |
| *Pla2g7* | 2.4062913613020672e-17 | 0.704440198 | Myeloid cells 1 |
| *Minos1* | 2.4419260609122142e-24 | 0.702272408 | Myeloid cells 1 |
| *Ly6i* | 1.6377249187686368e-20 | 0.701051653 | Myeloid cells 1 |
| *Psmb10* | 2.4776620341261825e-24 | 0.700461774 | Myeloid cells 1 |
| *Pomp* | 7.415569980741828e-23 | 0.700043596 | Myeloid cells 1 |
| *Fosl2* | 7.929400168643533e-32 | 0.696440743 | Myeloid cells 1 |
| *Hif1a* | 5.940901618565381e-21 | 0.69467191 | Myeloid cells 1 |
| *M6pr* | 4.5310316222658404e-021 | 0.690002138 | Myeloid cells 1 |
| *Uqcrq* | 3.807733842391633e-21 | 0.688094617 | Myeloid cells 1 |
| *Arpc3* | 1.0101234578654793e-21 | 0.688021332 | Myeloid cells 1 |
| *Tuba1b* | 8.385091435464381e-16 | 0.686828287 | Myeloid cells 1 |
| *Cfl1* | 4.346291703746097e-18 | 0.685225386 | Myeloid cells 1 |
| *Tlr2* | 4.276065844480243e-24 | 0.683103478 | Myeloid cells 1 |
| *Hspa8* | 3.857264534157602e-14 | 0.68210139 | Myeloid cells 1 |
| *Tax1bp1* | 3.0556874426731687e-14 | 0.681604878 | Myeloid cells 1 |
| *Ms4a7* | 3.056794936956406e-19 | 0.680917025 | Myeloid cells 1 |
| *Atp5j2* | 5.041003471455446e-20 | 0.676943949 | Myeloid cells 1 |
| *Itgb1* | 4.826034232898927e-17 | 0.676905448 | Myeloid cells 1 |
| *Zbp1* | 5.256982223753683e-30 | 0.675746385 | Myeloid cells 1 |
| *Slc15a3* | 4.821591585987509e-17 | 0.672377826 | Myeloid cells 1 |
| *Tomm7* | 1.0463315397362984e-23 | 0.668099557 | Myeloid cells 1 |
| *Grn* | 4.149558212525972e-11 | 0.666998625 | Myeloid cells 1 |
| *S100a6* | 5.947595894886187e-22 | 0.666156884 | Myeloid cells 1 |
| *Rel* | 2.2947645202772947e-16 | 0.659552564 | Myeloid cells 1 |
| *Aif1* | 9.628272218654878e-13 | 0.658172579 | Myeloid cells 1 |
| *Clta* | 4.340738710103486e-15 | 0.657166464 | Myeloid cells 1 |
| *Bcl2a1d* | 6.495810698975768e-28 | 0.656528087 | Myeloid cells 1 |
| *Chmp4b* | 1.4626600731155062e-20 | 0.654675478 | Myeloid cells 1 |
| *Eprs* | 2.3974109466352344e-19 | 0.653976895 | Myeloid cells 1 |
| *Atp5j* | 9.257937307064444e-18 | 0.653730555 | Myeloid cells 1 |
| *Gm10250* | 8.920038438224638e-20 | 0.652573596 | Myeloid cells 1 |
| *Tpt1-ps3* | 8.51275083735579e-18 | 0.650153735 | Myeloid cells 1 |
| *Trim30a* | 1.9700533755005092e-17 | 0.648263469 | Myeloid cells 1 |
| *Atp1b3* | 1.9243464644227332e-19 | 0.643333299 | Myeloid cells 1 |
| *Tpd52* | 6.085027873183847e-21 | 0.642490811 | Myeloid cells 1 |
| *Btg1* | 5.505741606313466e-15 | 0.641858547 | Myeloid cells 1 |
| *Ier3* | 4.214258432435489e-12 | 0.640668929 | Myeloid cells 1 |
| *Rpl13* | 4.853553381085214e-21 | 0.63948325 | Myeloid cells 1 |
| *Cyp4f18* | 2.332561557148978e-28 | 0.638522172 | Myeloid cells 1 |
| *Gm4149* | 8.798925528460215e-18 | 0.638372965 | Myeloid cells 1 |
| *Psma4* | 7.451947255077903e-21 | 0.636379433 | Myeloid cells 1 |
| *Bag1* | 7.876383131609549e-20 | 0.633349193 | Myeloid cells 1 |
| *Pde4b* | 7.248503774517327e-25 | 0.632392147 | Myeloid cells 1 |
| *Ccl4* | 2.3621358863156535e-4 | 0.630550982 | Myeloid cells 1 |
| *Gm8730* | 6.784431612302365e-21 | 0.626453606 | Myeloid cells 1 |
| *Anxa2* | 2.9246310955726614e-23 | 0.624925654 | Myeloid cells 1 |
| *Rpl37* | 5.9789315770646945e-18 | 0.622228747 | Myeloid cells 1 |
| *Hsp90b1* | 2.2331248553792914e-11 | 0.621513431 | Myeloid cells 1 |
| *Slamf7* | 1.420061155844235e-25 | 0.620866421 | Myeloid cells 1 |
| *Ms4a6d* | 1.606006517582222e-19 | 0.620588028 | Myeloid cells 1 |
| *Il1a* | 2.0745740998742763e-13 | 0.619420855 | Myeloid cells 1 |
| *Atf3* | 5.522380693089714e-9 | 0.619248542 | Myeloid cells 1 |
| *H2-Q6* | 5.202306997299875e-27 | 0.618618468 | Myeloid cells 1 |
| *Ifi209* | 1.015307694736565e-23 | 0.617078683 | Myeloid cells 1 |
| *Nfil3* | 3.829194159442258e-27 | 0.616481055 | Myeloid cells 1 |
| *Snx3* | 7.263709716772098e-20 | 0.616079861 | Myeloid cells 1 |
| *Sec61b* | 1.1893108301021363e-20 | 0.614685412 | Myeloid cells 1 |
| *Etf1* | 1.8110999906271912e-23 | 0.614447322 | Myeloid cells 1 |
| *Hnrnpa2b1* | 2.062999435838645e-12 | 0.61432153 | Myeloid cells 1 |
| *Gm6863* | 1.7016909226152257e-17 | 0.61371993 | Myeloid cells 1 |
| *Uqcrh* | 4.841032192708351e-20 | 0.611588047 | Myeloid cells 1 |
| *Nr4a2* | 1.1782338591857571e-23 | 0.610027206 | Myeloid cells 1 |
| *Gpr65* | 1.7446768296549004e-22 | 0.609164313 | Myeloid cells 1 |
| *Atp5c1* | 8.0556127481805e-18 | 0.607836466 | Myeloid cells 1 |
| *Hnrnpm* | 2.8565925275426222e-15 | 0.607629837 | Myeloid cells 1 |
| *Msn* | 1.8749006163396172e-15 | 0.606842376 | Myeloid cells 1 |
| *Psmb4* | 2.5224446240775347e-21 | 0.60577471 | Myeloid cells 1 |
| *Cox17* | 5.190000373691451e-18 | 0.603906422 | Myeloid cells 1 |
| *Rplp2* | 8.635384833050668e-15 | 0.603819954 | Myeloid cells 1 |
| *Aprt* | 8.118194813931497e-24 | 0.603068505 | Myeloid cells 1 |
| *Cd63* | 3.371042015709994e-7 | 0.602789026 | Myeloid cells 1 |
| *Tap1* | 7.529576201257506e-26 | 0.602188057 | Myeloid cells 1 |
| *Atp5g3* | 3.780673680655737e-19 | 0.599899521 | Myeloid cells 1 |
| *Ifi207* | 1.6950958475708363e-23 | 0.599025969 | Myeloid cells 1 |
| *Clec12a* | 3.2283805868483306e-25 | 0.598892666 | Myeloid cells 1 |
| *Taldo1* | 3.258761130624936e-17 | 0.597422595 | Myeloid cells 1 |
| *Clic4* | 2.709045397716188e-26 | 0.597411478 | Myeloid cells 1 |
| *Atpif1* | 2.154764302634064e-13 | 0.59613997 | Myeloid cells 1 |
| *Naaa* | 5.766744949731405e-17 | 0.595510736 | Myeloid cells 1 |
| *Ms4a6b* | 1.0319120250429743e-15 | 0.595185509 | Myeloid cells 1 |
| *Cdc42* | 2.336650755091767e-12 | 0.594641936 | Myeloid cells 1 |
| *Pfdn5* | 1.9576615265463008e-17 | 0.594440667 | Myeloid cells 1 |
| *Cd300lf* | 2.3242760437991855e-26 | 0.592128682 | Myeloid cells 1 |
| *Mt1* | 7.027380845184037e-7 | 0.590820842 | Myeloid cells 1 |
| *Csf2ra* | 4.627326571608364e-20 | 0.588812145 | Myeloid cells 1 |
| *Txn-ps1* | 3.7432528033393394e-21 | 0.588764559 | Myeloid cells 1 |
| *Atp6v0e* | 4.001199935957803e-18 | 0.587758501 | Myeloid cells 1 |
| *Gm6472* | 1.0353643978937763e-16 | 0.586980004 | Myeloid cells 1 |
| *Ifi203* | 2.0224460532453998e-21 | 0.585222417 | Myeloid cells 1 |
| *Ctsh* | 4.855385211614428e-13 | 0.585206879 | Myeloid cells 1 |
| *Prrc2c* | 8.795212095700735e-13 | 0.584363127 | Myeloid cells 1 |
| *Ndufa13* | 3.115113671895034e-16 | 0.58302329 | Myeloid cells 1 |
| *Nme1* | 5.189726013225814e-23 | 0.581571119 | Myeloid cells 1 |
| *Tyrobp* | 1.6529709074032013e-12 | 0.580824774 | Myeloid cells 1 |
| *Psmb6* | 3.8005747957024255e-16 | 0.579576923 | Myeloid cells 1 |
| *Ifi211* | 2.5153298742771527e-25 | 0.579468642 | Myeloid cells 1 |
| *Uqcr10* | 1.0023549675342264e-19 | 0.579276825 | Myeloid cells 1 |
| *Gm10169* | 6.1930887583713e-21 | 0.578931278 | Myeloid cells 1 |
| *Plaur* | 1.05826038895745e-23 | 0.577770405 | Myeloid cells 1 |
| *Gnai2* | 1.149629560828571e-14 | 0.577262741 | Myeloid cells 1 |
| *Mmp14* | 1.4083225603606195e-22 | 0.577030402 | Myeloid cells 1 |
| *Myo5a* | 4.4097780973432985e-23 | 0.576537286 | Myeloid cells 1 |
| *Abracl* | 5.596633440808008e-21 | 0.576379142 | Myeloid cells 1 |
| *Oaz1* | 3.048388503482112e-17 | 0.576334552 | Myeloid cells 1 |
| *Ets2* | 8.813806559662594e-25 | 0.573942664 | Myeloid cells 1 |
| *Rpl9-ps6* | 9.565308591253757e-18 | 0.571348989 | Myeloid cells 1 |
| *Eif5a* | 9.379746490233897e-15 | 0.570915452 | Myeloid cells 1 |
| *Gm11560* | 4.9699380634388836e-18 | 0.57028802 | Myeloid cells 1 |
| *Eif3f* | 8.64945516193246e-18 | 0.569790481 | Myeloid cells 1 |
| *Cd274* | 3.049803607552657e-24 | 0.566950194 | Myeloid cells 1 |
| *Dusp1* | 1.760844183071848e-11 | 0.565389754 | Myeloid cells 1 |
| *Mrc1* | 1.6800863118734617e-16 | 0.56356123 | Myeloid cells 1 |
| *Ccnd1* | 4.623654421360504e-16 | 0.563179324 | Myeloid cells 1 |
| *Tnfaip3* | 1.2216535851630165e-16 | 0.562535462 | Myeloid cells 1 |
| *Mxd1* | 2.1138508419204317e-21 | 0.561766747 | Myeloid cells 1 |
| *Atp5h* | 9.290963307739289e-19 | 0.561619395 | Myeloid cells 1 |
| *1810037I17Rik* | 2.570457599154668e-18 | 0.558220169 | Myeloid cells 1 |
| *Atp5d* | 1.4438444206948126e-16 | 0.557414979 | Myeloid cells 1 |
| *Actr2* | 7.196882015068062e-14 | 0.555717207 | Myeloid cells 1 |
| *Atp6v1f* | 9.275963666973587e-16 | 0.555688306 | Myeloid cells 1 |
| *Hspa4* | 2.604999364808551e-13 | 0.555473261 | Myeloid cells 1 |
| *Cytip* | 2.1844091474632344e-22 | 0.555451415 | Myeloid cells 1 |
| *Atp5f1* | 1.7287211195045554e-13 | 0.554893613 | Myeloid cells 1 |
| *Got1* | 4.868107828497906e-25 | 0.554829986 | Myeloid cells 1 |
| *Gm15500* | 1.923675114793351e-13 | 0.553407573 | Myeloid cells 1 |
| *C3* | 6.554593531276916e-22 | 0.553385991 | Myeloid cells 1 |
| *Lamp1* | 4.168133042114187e-10 | 0.552886171 | Myeloid cells 1 |
| *Rbms1* | 2.349930140958108e-21 | 0.552489133 | Myeloid cells 1 |
| *Sdhb* | 1.5099761521139458e-16 | 0.552150285 | Myeloid cells 1 |
| *Rpl38-ps2* | 4.4384978155094036e-17 | 0.551730725 | Myeloid cells 1 |
| *Atp2b1* | 1.244610073837793e-11 | 0.551366623 | Myeloid cells 1 |
| *Gbp8* | 4.535099759017002e-23 | 0.550163683 | Myeloid cells 1 |
| *Spop* | 8.900801537925547e-16 | 0.548766592 | Myeloid cells 1 |
| *Pdia6* | 2.6384618376408228e-11 | 0.547753406 | Myeloid cells 1 |
| *Eif3k* | 4.604443922973849e-20 | 0.547643141 | Myeloid cells 1 |
| *Tapbp* | 3.748231485063519e-16 | 0.546049897 | Myeloid cells 1 |
| *Slfn5* | 7.723781047801581e-20 | 0.545593591 | Myeloid cells 1 |
| *Sp100* | 8.181636521961667e-23 | 0.545325263 | Myeloid cells 1 |
| *Gm2a* | 2.2387565125464158e-13 | 0.544426677 | Myeloid cells 1 |
| *Cd47* | 8.567355004464041e-13 | 0.544290146 | Myeloid cells 1 |
| *Tceb2* | 3.4076844814761423e-15 | 0.543755829 | Myeloid cells 1 |
| *Eef1g* | 8.887552273578748e-18 | 0.542845292 | Myeloid cells 1 |
| *Ndufa3* | 2.9239359604150766e-16 | 0.542817939 | Myeloid cells 1 |
| *Zfas1* | 7.044409807196178e-17 | 0.542134347 | Myeloid cells 1 |
| *Ndufc1* | 3.7598833462658476e-18 | 0.541143235 | Myeloid cells 1 |
| *Efhd2* | 1.1728463370727916e-12 | 0.539794003 | Myeloid cells 1 |
| *Psma1* | 2.2860874763469016e-15 | 0.539530963 | Myeloid cells 1 |
| *Banf1* | 1.5279831740636917e-19 | 0.539525788 | Myeloid cells 1 |
| *Irf7* | 8.641031586783833e-24 | 0.538547412 | Myeloid cells 1 |
| *Eif3h* | 1.5177469661613754e-18 | 0.53814715 | Myeloid cells 1 |
| *Hspe1* | 1.847572948114149e-14 | 0.537983351 | Myeloid cells 1 |
| *Clic1* | 5.05004157575741e-11 | 0.533202457 | Myeloid cells 1 |
| *Sri* | 5.534734211397591e-19 | 0.531888597 | Myeloid cells 1 |
| *Csrnp1* | 5.439836911331041e-22 | 0.531540635 | Myeloid cells 1 |
| *D8Ertd738e* | 2.0243787060838766e-15 | 0.53034027 | Myeloid cells 1 |
| *Ndufa1* | 3.0015076991168706e-18 | 0.529926784 | Myeloid cells 1 |
| *Eif3c* | 4.701499971887491e-13 | 0.528896869 | Myeloid cells 1 |
| *H2afy* | 1.0507004276806399e-15 | 0.527460832 | Myeloid cells 1 |
| *Rpsa* | 2.7421246362042883e-17 | 0.527151126 | Myeloid cells 1 |
| *Axl* | 5.093516051170421e-24 | 0.523591419 | Myeloid cells 1 |
| *Gm4617* | 7.500116764320368e-12 | 0.523224781 | Myeloid cells 1 |
| *Gna13* | 2.3490144884721743e-15 | 0.522663022 | Myeloid cells 1 |
| *Shisa5* | 3.038987344838477e-15 | 0.522377824 | Myeloid cells 1 |
| *Sod2* | 3.685254591618332e-18 | 0.520987179 | Myeloid cells 1 |
| *Sp140* | 1.4771438617167746e-17 | 0.520859416 | Myeloid cells 1 |
| *Irf1* | 6.894591255406686e-16 | 0.520835689 | Myeloid cells 1 |
| *Ranbp1* | 3.745262023843872e-12 | 0.518864567 | Myeloid cells 1 |
| *Uqcrb* | 1.1592045419657496e-14 | 0.517366164 | Myeloid cells 1 |
| *Fabp5* | 3.7117115557579053e-13 | 0.514355879 | Myeloid cells 1 |
| *Nlrp3* | 1.517671069957796e-15 | 0.51415078 | Myeloid cells 1 |
| *Rpl37a* | 5.5185529049854326e-17 | 0.512281181 | Myeloid cells 1 |
| *Ccl7* | 3.3578769263437425e-12 | 0.511026159 | Myeloid cells 1 |
| *Tnfaip8* | 7.474400364262132e-15 | 0.510866669 | Myeloid cells 1 |
| *Irgm1* | 3.6217527635327e-17 | 0.510708706 | Myeloid cells 1 |
| *Rpl3* | 1.1351588599664242e-13 | 0.51036855 | Myeloid cells 1 |
| *Cd44* | 2.0206646540657114e-23 | 0.510234476 | Myeloid cells 1 |
| *Ppib* | 2.84671391707613e-12 | 0.509819213 | Myeloid cells 1 |
| *Gm14513* | 2.1789443085065573e-16 | 0.509727588 | Myeloid cells 1 |
| *Ndufa2* | 1.3388623635248004e-13 | 0.509569298 | Myeloid cells 1 |
| *Slk* | 8.250960143240202e-15 | 0.509010935 | Myeloid cells 1 |
| *Phf11b* | 8.409420675582838e-20 | 0.507883906 | Myeloid cells 1 |
| *Arl5c* | 1.8357273894101352e-16 | 0.507206779 | Myeloid cells 1 |
| *Prdx6* | 2.8748960793327237e-16 | 0.50714505 | Myeloid cells 1 |
| *Gm14539* | 4.051662142078099e-17 | 0.506794673 | Myeloid cells 1 |
| *Epsti1* | 1.4852963880109384e-16 | 0.506769659 | Myeloid cells 1 |
| *Gm10076* | 3.377216299032284e-17 | 0.506596946 | Myeloid cells 1 |
| *Gm42418* | 1.222051830690333e-4 | 0.506357359 | Myeloid cells 1 |
| *Gbp4* | 9.294034839373805e-21 | 0.505752929 | Myeloid cells 1 |
| *Tuba1c* | 6.859251825797768e-22 | 0.50558589 | Myeloid cells 1 |
| *Pla2g16* | 8.466219808234996e-22 | 0.505146167 | Myeloid cells 1 |
| *BC005537* | 8.996571093942199e-12 | 0.503530997 | Myeloid cells 1 |
| *Serp1* | 1.2946024540593076e-12 | 0.503308638 | Myeloid cells 1 |
| *Txnrd1* | 7.479328542861037e-17 | 0.503116223 | Myeloid cells 1 |
| *H2-T22* | 3.702085040994885e-20 | 0.50214894 | Myeloid cells 1 |
| *Gm14681* | 3.34535315670483e-16 | 0.501807446 | Myeloid cells 1 |
| *Ubl5* | 2.524251773073673e-15 | 0.50059829 | Myeloid cells 1 |
| *B2m* | 1.2354403996433616e-28 | 2.380794319 | Oligodendrocytes |
| *H2-D1* | 5.305244039036554e-24 | 1.906063592 | Oligodendrocytes |
| *H2-K1* | 1.996219503132167e-18 | 1.152424993 | Oligodendrocytes |
| *Xist* | 1.3643549742865497e-15 | 0.643061465 | Oligodendrocytes |
| *Igtp* | 1.3692818509858427e-15 | 0.672386381 | Oligodendrocytes |
| *Psmb8* | 1.256005228101512e-14 | 0.734597272 | Oligodendrocytes |
| *Irgm1* | 4.420194983082095e-14 | 0.677828969 | Oligodendrocytes |
| *C4b* | 2.263575774342385e-13 | 0.73452616 | Oligodendrocytes |
| *Stat1* | 1.3757669433420816e-12 | 0.526042334 | Oligodendrocytes |
| *Iigp1* | 1.862844588396277e-11 | 0.655983359 | Oligodendrocytes |
| *Fos* | 3.8388723711879715e-11 | 0.778377356 | Oligodendrocytes |
| *H2-T23* | 6.146000220942457e-10 | 0.596906195 | Oligodendrocytes |
| *Serpina3n* | 2.0358765818367528e-9 | 0.507374929 | Oligodendrocytes |
| *Ifi27* | 5.004682243903247e-9 | 0.557047943 | Oligodendrocytes |
| *Trf* | 4.907382749548947e-8 | 0.799588938 | Oligodendrocytes |
| *Ifi27l2a* | 1.302174731898946e-6 | 0.521746581 | Oligodendrocytes |
| *Jun* | 2.675120898370744e-5 | 0.509291982 | Oligodendrocytes |
| *Neat1* | 4.081500355688495e-5 | 0.532732686 | Oligodendrocytes |
