# Supplementary material for: Elucidating tumour‐associated microglia/macrophage diversity along glioblastoma progression and under ACOD1 deficiency
Source: Mol Oncol. 2022 Aug 15;16(17):3167–91. doi: 10.1002/1878-0261.13287 (PMC9441003; doi:10.1002/1878-0261.13287)
Supplement: Supplementary file 3 — Table S2. List of the most differentially expressed genes across the myeloid clusters (Naïve, TAM I and TAM II), irrespective of the tumour stage (p‐value < 0.01), related to Figure 2. [file MOL2-16-3167-s002.docx]

**Table S2. List of the most differentially expressed genes across the myeloid clusters (Naïve, TAM I and TAM II), irrespective of the tumour stage (p-value < 0.01), related to figure 2.**

| Represented genes (ordered as in the heat-map) |
| --- |
| Gene symbol |
| *Cst3* |
| *Hexb* |
| *Ctsb* |
| *C1qa* |
| *C1qb* |
| *Vegfa* |
| *Clec4n* |
| *Cfp* |
| *S100a11* |
| *Ccr1* |
| *Msrb1* |
| *Clec12a* |
| *S100a10* |
| *AA467197* |
| *Crip1* |
| *Lgals3* |
| *Vim* |
| *Bst2* |
| *Lgals1* |
| *Cdkn1a* |
| *Cybb* |
| *Fxyd5* |
| *Dab2* |
| *Iqgap1* |
| *Tgfbi* |
| *Thbs1* |
| *Tmsb10* |
| *Txn1* |
| *Prdx5* |
| *Cstb* |
| *Lilrb4a* |
| *Ltc4s* |
| *Pmp22* |
| *Hpgd* |
| *Cd37* |
| *Cfh* |
| *Sall1* |
| *P2ry13* |
| *Slco2b1* |
| *Hpgds* |
| *Srgap2* |
| *Plxdc2* |
| *Serpine2* |
| *Mef2c* |
| *Olfml3* |
| *Arhgap5* |
| *Tmem119* |
| *Cd83* |
| *Il1a* |
| *Nr4a1* |
| *Ccl12* |
| *Xist* |
| *H2-Q6* |
| *H2-Q7* |
| *Lgals3bp* |
| *Ifi204* |
| *Stat1* |
| *Ccl3* |
| *Ccl4* |
| *P2ry12* |
| *Sparc* |
| *Csf1r* |
| *Cx3cr1* |
| *Cd81* |
| *Fcrls* |
| *Gpr34* |
| *Siglech* |
| *Selplg* |
| *Bin1* |
| *Golm1* |
| *Cd74* |
| *H2-Ab1* |
| *H2-Aa* |
| *H2-Eb1* |
| *Rplp1* |
| *Rps14* |
| *Gm9843* |
| *Rps3* |
| *Calm1* |
| *H2-D1* |
| *H2-K1* |
| *Cd52* |
| *Ifi27l2a* |
| *Ifitm3* |
| *Plek* |
| *Wdr89* |
| *Gm9794* |
| *Rpl32* |
| *Rps5* |
| *Rpl4* |
| *Rps20* |
| *Rps26* |
| *Rps10-ps1* |
| *Gm10288* |
| *Rps16-ps2* |
| *Gm14303* |
| *Rps15* |
| *Rpl8* |
| *Rack1* |
| *Rpl39* |
| *Rplp0* |
| *Il1b* |
| *Clec7a* |
| *Srgn* |
| *Lyz2* |
| *Fth1* |
| *B2m* |
| *Tmsb4x* |
