# Supplementary material for: Elucidating tumour‐associated microglia/macrophage diversity along glioblastoma progression and under ACOD1 deficiency
Source: Mol Oncol. 2022 Aug 15;16(17):3167–91. doi: 10.1002/1878-0261.13287 (PMC9441003; doi:10.1002/1878-0261.13287)
Supplement: Supplementary file 4 — Table S3. Common transcriptional signatures between tumour‐associated microglia and macrophages in the GBM syngeneic murine model and in patients used to assign a score for each TCGA patient, related to Figure 2. [file MOL2-16-3167-s001.docx]

**Table S3. Shared tumour-associated microglia and macrophage gene signatures between GBM GL261 murine model and patients used to assign a score for each TCGA patient, related to figure 2.**

|  | **List name** | **Gene symbol** |
| --- | --- | --- |
| **1** | **TAM I** | *CCL4* |
| **2** | **TAM I** | *CCL3* |
| **3** | **TAM I** | *P2RY12* |
| **4** | **TAM I** | *EGR1* |
| **5** | **TAM I** | *CX3CR1* |
| **6** | **TAM I** | *JUN* |
| **7** | **TAM I** | *BIN1* |
| **8** | **TAM I** | *SELPLG* |
| **9** | **TAM I** | *CD83* |
| **10** | **TAM I** | *RHOB* |
| **11** | **TAM I** | *CKB* |
| **12** | **TAM I** | *SRGAP2* |
| **13** | **TAM I** | *MARCKS* |
| **14** | **TAM I** | *ENTPD1* |
| **15** | **TAM I** | *SMAP2* |
| **16** | **TAM I** | *ZFHX3* |
| **17** | **TAM I** | *P2RY13* |
| **18** | **TAM I** | *SALL1* |
| **19** | **TAM I** | *ITM2B* |
| **20** | **TAM I** | *JUND* |
| **21** | **TAM I** | *ITM2C* |
|  |  |  |
| **1** | **TAM II** | *TGFBI* |
| **2** | **TAM II** | *THBS1* |
| **3** | **TAM II** | *LGALS1* |
| **4** | **TAM II** | *S100A11* |
| **5** | **TAM II** | *MSR1* |
| **6** | **TAM II** | *VIM* |
| **7** | **TAM II** | *IL1B* |
| **8** | **TAM II** | *TMSB10* |
| **9** | **TAM II** | *IQGAP1* |
| **10** | **TAM II** | *IL1RN* |
| **11** | **TAM II** | *PRDX5* |
| **12** | **TAM II** | *PLBD1* |
| **13** | **TAM II** | *PLAC8* |
| **14** | **TAM II** | *FXYD5* |
| **15** | **TAM II** | *FABP5* |
| **16** | **TAM II** | *F13A1* |
| **17** | **TAM II** | *IFITM3* |
| **18** | **TAM II** | *ANXA5* |
| **19** | **TAM II** | *CYBB* |
| **20** | **TAM II** | *SH3BGRL* |
| **21** | **TAM II** | *COX5A* |
| **22** | **TAM II** | *PRDX6* |
| **23** | **TAM II** | *CSTB* |
| **24** | **TAM II** | *ISG15* |
| **25** | **TAM II** | *EMB* |
| **26** | **TAM II** | *FOSL2* |
| **27** | **TAM II** | *PIM1* |
| **28** | **TAM II** | *SAMHD1* |
| **29** | **TAM II** | *CTSC* |
| **30** | **TAM II** | *ARPC1B* |
| **31** | **TAM II** | *PLAUR* |
| **32** | **TAM II** | *PFN1* |
| **33** | **TAM II** | *MYL12A* |
| **34** | **TAM II** | *CD93* |
| **35** | **TAM II** | *NAMPT* |
| **36** | **TAM II** | *AHNAK* |
| **37** | **TAM II** | *IFITM2* |
| **38** | **TAM II** | *CD14* |
| **39** | **TAM II** | *CREM* |
| **40** | **TAM II** | *ANXA2* |
| **41** | **TAM II** | *METRNL* |
| **42** | **TAM II** | *MXD1* |
| **43** | **TAM II** | *LST1* |
| **44** | **TAM II** | *SOD2* |
| **45** | **TAM II** | *ATP5G3* |
| **46** | **TAM II** | *CCRL2* |
| **47** | **TAM II** | *EMP3* |
| **48** | **TAM II** | *S100A6* |
| **49** | **TAM II** | *TALDO1* |
| **50** | **TAM II** | *COX7B* |
| **51** | **TAM II** | *BHLHE40* |
| **52** | **TAM II** | *SNX2* |
| **53** | **TAM II** | *ANXA1* |
| **54** | **TAM II** | *RNF149* |
| **55** | **TAM II** | *PDE4B* |
| **56** | **TAM II** | *LSP1* |
| **57** | **TAM II** | *ARPC5* |
| **58** | **TAM II** | *ATP5E* |
| **59** | **TAM II** | *SDCBP* |
| **60** | **TAM II** | *PILRA* |
| **61** | **TAM II** | *LDHA* |
| **62** | **TAM II** | *BST2* |
| **63** | **TAM II** | *NFIL3* |
| **64** | **TAM II** | *FAM96A* |
| **65** | **TAM II** | *ATP5H* |
| **66** | **TAM II** | *NINJ1* |
| **67** | **TAM II** | *PSMB8* |
| **68** | **TAM II** | *PSMD14* |
| **69** | **TAM II** | *GPR65* |
| **70** | **TAM II** | *SEC61B* |
| **71** | **TAM II** | *CSF2RB* |
| **72** | **TAM II** | *PSMB1* |
| **73** | **TAM II** | *TAGLN2* |
| **74** | **TAM II** | *ITGB1* |
| **75** | **TAM II** | *H3F3A* |
| **76** | **TAM II** | *IFI30* |
| **77** | **TAM II** | *ALOX5AP* |
| **78** | **TAM II** | *CXCR4* |
| **79** | **TAM II** | *ARPC3* |
| **80** | **TAM II** | *COX6B1* |
| **81** | **TAM II** | *APRT* |
| **82** | **TAM II** | *PSME2* |
| **83** | **TAM II** | *CAPG* |
| **84** | **TAM II** | *CYBA* |
