# Supplementary material for: Elucidating tumour‐associated microglia/macrophage diversity along glioblastoma progression and under ACOD1 deficiency
Source: Mol Oncol. 2022 Aug 15;16(17):3167–91. doi: 10.1002/1878-0261.13287 (PMC9441003; doi:10.1002/1878-0261.13287)
Supplement: Supplementary file 5 — Table S4. Up‐regulated differentially expressed genes at early and late stages for TAM I and TAM II versus naïve cells (p‐value < 0.001 and log2 FC > 0.5), related to Figure 3. [file MOL2-16-3167-s005.docx]

**Table S4. Up-regulated differentially expressed genes at early and late stages for TAM I and TAM II versus naïve cells (p-value < 0.001 and log2 FC > 0.5), related to figure 3.**

| Gene symbol | p-value | logFC | Comparison |
| --- | --- | --- | --- |
| *Cd74* | 3.42842566652637e-29 | 4.8184728 | TAM I early vs naïve |
| *H2-Ab1* | 6.052854995142116e-29 | 4.05605287 | TAM I early vs naïve |
| *H2-Aa* | 3.2383700943954223e-29 | 3.71863876 | TAM I early vs naïve |
| *H2-Eb1* | 3.500266580254675e-28 | 3.58597478 | TAM I early vs naïve |
| *H2-D1* | 5.723278440105005e-23 | 3.27176871 | TAM I early vs naïve |
| *H2-K1* | 5.668189747762691e-24 | 3.16908824 | TAM I early vs naïve |
| *Apoe* | 1.772835648919748e-8 | 2.54118914 | TAM I early vs naïve |
| *Fth1* | 4.1255924135249015e-15 | 2.3993808 | TAM I early vs naïve |
| *B2m* | 2.694593413230408e-20 | 2.36729982 | TAM I early vs naïve |
| *Lyz2* | 2.1360505251226754e-10 | 1.88501176 | TAM I early vs naïve |
| *Rplp1* | 9.994148448368303e-19 | 1.87989496 | TAM I early vs naïve |
| *Clec7a* | 2.0730241619723105e-10 | 1.86350458 | TAM I early vs naïve |
| *Xist* | 6.27645900952657e-27 | 1.82713192 | TAM I early vs naïve |
| *Gm42418* | 3.1949012934739323e-14 | 1.78884161 | TAM I early vs naïve |
| *Ccl4* | 4.256064574553853e-9 | 1.75456882 | TAM I early vs naïve |
| *Rpl32* | 2.2990942944078886e-14 | 1.7235482 | TAM I early vs naïve |
| *Il1b* | 1.3874859869615306e-13 | 1.71378363 | TAM I early vs naïve |
| *Ccl12* | 2.1335070377057337e-15 | 1.70860981 | TAM I early vs naïve |
| *Rps20* | 5.761984487535602e-16 | 1.66853088 | TAM I early vs naïve |
| *Cd52* | 5.809841293438737e-12 | 1.6522946 | TAM I early vs naïve |
| *Plek* | 6.998663689490088e-14 | 1.63438058 | TAM I early vs naïve |
| *Ctsb* | 1.41402423913676e-10 | 1.60414356 | TAM I early vs naïve |
| *Gm9794* | 2.691421206449615e-14 | 1.58293121 | TAM I early vs naïve |
| *H2-Q7* | 4.1516358226402237e-22 | 1.57352889 | TAM I early vs naïve |
| *Cd83* | 2.7785740462325326e-13 | 1.55844788 | TAM I early vs naïve |
| *Npc2* | 8.627795649813469e-9 | 1.52021824 | TAM I early vs naïve |
| *Atf3* | 5.261451718662752e-8 | 1.51593423 | TAM I early vs naïve |
| *Rps5* | 5.843235280662749e-12 | 1.48395551 | TAM I early vs naïve |
| *Lgals3bp* | 1.2129162317144415e-14 | 1.44683717 | TAM I early vs naïve |
| *Gm14303* | 1.350595745779837e-13 | 1.44361988 | TAM I early vs naïve |
| *Cst7* | 8.759603404907849e-11 | 1.42068214 | TAM I early vs naïve |
| *Gm9843* | 1.9080580420683564e-13 | 1.40440852 | TAM I early vs naïve |
| *Rpl22* | 2.4335957686688937e-12 | 1.39989088 | TAM I early vs naïve |
| *C1qa* | 5.969088619459232e-14 | 1.39091265 | TAM I early vs naïve |
| *Rpl4* | 2.5811265151138374e-12 | 1.38924809 | TAM I early vs naïve |
| *Rps3* | 2.4820921782372494e-13 | 1.37884175 | TAM I early vs naïve |
| *Rps26* | 6.243607920299757e-11 | 1.37168309 | TAM I early vs naïve |
| *Gbp2* | 5.339908051034442e-15 | 1.37038136 | TAM I early vs naïve |
| *Nfkbia* | 6.063890778551266e-8 | 1.36947895 | TAM I early vs naïve |
| *Rps14* | 9.879402337791503e-11 | 1.36629885 | TAM I early vs naïve |
| *Mpeg1* | 2.3157499923113684e-12 | 1.35636407 | TAM I early vs naïve |
| *Ctsd* | 5.427853683448746e-8 | 1.34155488 | TAM I early vs naïve |
| *Ctsz* | 5.644468979272892e-10 | 1.34005559 | TAM I early vs naïve |
| *Wdr89* | 1.494357175509679e-11 | 1.3158359 | TAM I early vs naïve |
| *H2-T23* | 8.18281639369812e-15 | 1.30753072 | TAM I early vs naïve |
| *Tmsb4x* | 5.1139988932741955e-11 | 1.30629787 | TAM I early vs naïve |
| *Rps9* | 1.893835420204462e-11 | 1.29766661 | TAM I early vs naïve |
| *Ctss* | 3.6326448736667113e-14 | 1.29697354 | TAM I early vs naïve |
| *Rps24* | 6.317134993661623e-12 | 1.29000085 | TAM I early vs naïve |
| *Gm11478* | 1.2351952173054974e-10 | 1.28868666 | TAM I early vs naïve |
| *Srgn* | 3.402123429060815e-9 | 1.28536939 | TAM I early vs naïve |
| *H2-DMa* | 2.0072845076801338e-13 | 1.28134491 | TAM I early vs naïve |
| *Rpl39* | 1.9161152193349607e-13 | 1.27477842 | TAM I early vs naïve |
| *Ly86* | 4.1773763282557953e-10 | 1.2745801 | TAM I early vs naïve |
| *C1qb* | 6.642762710906134e-13 | 1.26831975 | TAM I early vs naïve |
| *Gm5963* | 2.5906844231030206e-10 | 1.26484184 | TAM I early vs naïve |
| *Ncl* | 1.470772976368067e-10 | 1.26407551 | TAM I early vs naïve |
| *Pabpc1* | 7.776998863957213e-11 | 1.25868458 | TAM I early vs naïve |
| *Rps15* | 1.4612222905390008e-11 | 1.23743302 | TAM I early vs naïve |
| *Ccl3* | 3.250506115060712e-7 | 1.23487963 | TAM I early vs naïve |
| *Ctsc* | 2.215378802927859e-8 | 1.22852481 | TAM I early vs naïve |
| *Cd63* | 9.404265636155693e-8 | 1.21220091 | TAM I early vs naïve |
| *Rps16-ps2* | 6.170440211542207e-14 | 1.21212386 | TAM I early vs naïve |
| *Rpl41* | 1.1286175013540502e-10 | 1.21178237 | TAM I early vs naïve |
| *Psmb8* | 4.179509910628366e-11 | 1.20642027 | TAM I early vs naïve |
| *Ly6e* | 1.6452253032725673e-9 | 1.20147755 | TAM I early vs naïve |
| *Rplp0* | 7.724736260884363e-11 | 1.17450584 | TAM I early vs naïve |
| *Gm6977* | 3.183294388157276e-6 | 1.16714327 | TAM I early vs naïve |
| *Rps19* | 1.741494060342758e-11 | 1.16616664 | TAM I early vs naïve |
| *Stat1* | 9.09000361984914e-17 | 1.16567723 | TAM I early vs naïve |
| *Ccl5* | 3.902528596001646e-11 | 1.16172166 | TAM I early vs naïve |
| *Gm8995* | 1.1842768018432856e-12 | 1.15876642 | TAM I early vs naïve |
| *Cd14* | 1.4180484192767218e-5 | 1.14846152 | TAM I early vs naïve |
| *Ifi27l2a* | 2.9962945660492826e-14 | 1.13849808 | TAM I early vs naïve |
| *Rps21* | 7.756420500383514e-9 | 1.13550911 | TAM I early vs naïve |
| *Gm10288* | 1.651822200509028e-11 | 1.13490781 | TAM I early vs naïve |
| *Cd63-ps* | 1.9971335262579386e-7 | 1.13381755 | TAM I early vs naïve |
| *Gm16580* | 1.111069934470339e-8 | 1.13363478 | TAM I early vs naïve |
| *Rpl26* | 2.217468359067899e-12 | 1.12885703 | TAM I early vs naïve |
| *C1qc* | 9.049475382386727e-9 | 1.124726 | TAM I early vs naïve |
| *Tyrobp* | 3.269726070699912e-10 | 1.12249798 | TAM I early vs naïve |
| *Psap* | 6.618920098245555e-7 | 1.1184259 | TAM I early vs naïve |
| *Eef1a1* | 2.3930086393317583e-8 | 1.10415011 | TAM I early vs naïve |
| *Rps10-ps1* | 1.2613700788068206e-10 | 1.09349002 | TAM I early vs naïve |
| *Rpl14-ps1* | 1.2540923904976205e-8 | 1.09294403 | TAM I early vs naïve |
| *Nfkbiz* | 7.366181734209625e-9 | 1.08417937 | TAM I early vs naïve |
| *Rpl8* | 9.45451097796713e-10 | 1.06770684 | TAM I early vs naïve |
| *Gm5805* | 4.388394578655797e-9 | 1.06361404 | TAM I early vs naïve |
| *Mcl1* | 5.693644483392923e-11 | 1.05865418 | TAM I early vs naïve |
| *Aif1* | 3.321792547442538e-8 | 1.05705259 | TAM I early vs naïve |
| *Nfe2l2* | 1.0446106077279066e-7 | 1.05226587 | TAM I early vs naïve |
| *AU020206* | 1.7476657320255098e-10 | 1.05040904 | TAM I early vs naïve |
| *Cebpb* | 3.8933203739935333e-10 | 1.04847528 | TAM I early vs naïve |
| *Rps13-ps2* | 1.346621207249483e-9 | 1.04635209 | TAM I early vs naïve |
| *Rps15a* | 8.191951142144165e-10 | 1.04513278 | TAM I early vs naïve |
| *Gm15427* | 4.052281419676528e-9 | 1.04144468 | TAM I early vs naïve |
| *Grn* | 8.467853543011257e-7 | 1.03587847 | TAM I early vs naïve |
| *Gm4332* | 6.004734490857987e-10 | 1.03441236 | TAM I early vs naïve |
| *Cxcl10* | 1.7395056062452947e-9 | 1.03136335 | TAM I early vs naïve |
| *Gm5905* | 7.947560422747839e-9 | 1.02159803 | TAM I early vs naïve |
| *Rack1* | 9.079772838208605e-10 | 1.01780921 | TAM I early vs naïve |
| *Cxcl9* | 7.423573573252092e-9 | 1.01598074 | TAM I early vs naïve |
| *Aldoa* | 1.1971633219339935e-10 | 1.00925607 | TAM I early vs naïve |
| *Rps2* | 6.972793036388488e-9 | 1.00631358 | TAM I early vs naïve |
| *Bst2* | 7.165567149484619e-11 | 1.00246393 | TAM I early vs naïve |
| *Slc15a3* | 9.320708857756109e-9 | 1.00221524 | TAM I early vs naïve |
| *Rps25-ps1* | 2.3482701545692735e-11 | 1.00056788 | TAM I early vs naïve |
| *Il1a* | 2.1396844076857053e-11 | 0.99459506 | TAM I early vs naïve |
| *Hsp90ab1* | 1.1161648837781169e-6 | 0.98374566 | TAM I early vs naïve |
| *Eif3a* | 3.234280127089125e-8 | 0.98303357 | TAM I early vs naïve |
| *Fcer1g* | 2.783542337071728e-7 | 0.97804947 | TAM I early vs naïve |
| *Bcl2a1b* | 4.981841919008188e-8 | 0.97772645 | TAM I early vs naïve |
| *Rpl23* | 1.1843741316319143e-9 | 0.97588034 | TAM I early vs naïve |
| *Ccrl2* | 4.0598990377410945e-9 | 0.97205234 | TAM I early vs naïve |
| *Rpl34* | 1.3809115371936364e-8 | 0.9652638 | TAM I early vs naïve |
| *Rps24-ps3* | 6.778161715318954e-8 | 0.96381738 | TAM I early vs naïve |
| *Cox4i1* | 2.8620537626674682e-8 | 0.96007431 | TAM I early vs naïve |
| *Gpx1* | 1.4642015737237522e-7 | 0.93910217 | TAM I early vs naïve |
| *Prdx1* | 9.436115243901349e-8 | 0.93686429 | TAM I early vs naïve |
| *Rpl37rt* | 3.9554476988140786e-8 | 0.93482767 | TAM I early vs naïve |
| *Prrc2c* | 1.4939386377584292e-8 | 0.92840303 | TAM I early vs naïve |
| *Ifitm3* | 9.604120694287919e-13 | 0.92816923 | TAM I early vs naïve |
| *Cybb* | 3.1864295165686286e-9 | 0.92159604 | TAM I early vs naïve |
| *Gm10275* | 2.4423853595391023e-10 | 0.92018734 | TAM I early vs naïve |
| *Rpl14* | 2.7335155964810532e-8 | 0.91799089 | TAM I early vs naïve |
| *Chd4* | 1.1709868135488898e-8 | 0.91793754 | TAM I early vs naïve |
| *Egr1* | 1.2066179874761819e-4 | 0.9112937 | TAM I early vs naïve |
| *C4b* | 7.888600362593126e-14 | 0.90332557 | TAM I early vs naïve |
| *Rpsa-ps10* | 1.8854311260611764e-7 | 0.90154961 | TAM I early vs naïve |
| *Eef1b2* | 5.274849207098021e-8 | 0.90018972 | TAM I early vs naïve |
| *Ifi204* | 1.8232375711202943e-13 | 0.89842921 | TAM I early vs naïve |
| *Cd9* | 1.234370509871478e-6 | 0.89684008 | TAM I early vs naïve |
| *Gbp7* | 1.674165654076198e-7 | 0.89627783 | TAM I early vs naïve |
| *Cox6a1* | 1.3243753841997105e-8 | 0.89580566 | TAM I early vs naïve |
| *Npm1* | 4.356304504697898e-11 | 0.8931714 | TAM I early vs naïve |
| *Gm5835* | 3.525505951005804e-9 | 0.89071935 | TAM I early vs naïve |
| *Nr4a1* | 1.605507164161184e-12 | 0.89013706 | TAM I early vs naïve |
| *H2-Q6* | 6.772559454651446e-15 | 0.88727578 | TAM I early vs naïve |
| *Lilrb4a* | 4.166858589796313e-10 | 0.88722507 | TAM I early vs naïve |
| *Timp2* | 0.0019595111668414917 | 0.88212367 | TAM I early vs naïve |
| *Rps18* | 3.3469127319182003e-8 | 0.88109557 | TAM I early vs naïve |
| *Ly6a* | 3.0433712118437926e-13 | 0.87999208 | TAM I early vs naïve |
| *Gm4149* | 6.368622114556244e-10 | 0.87767631 | TAM I early vs naïve |
| *Clta* | 1.1548975540088476e-6 | 0.87072955 | TAM I early vs naïve |
| *H2-Oa* | 2.344170640273488e-13 | 0.8629489 | TAM I early vs naïve |
| *Rpl35a* | 3.0672364084305087e-6 | 0.85986278 | TAM I early vs naïve |
| *Tpr* | 3.401436614482932e-9 | 0.85787326 | TAM I early vs naïve |
| *Eef2* | 3.918023496140191e-7 | 0.85596217 | TAM I early vs naïve |
| *Gm14586* | 8.831962603765702e-9 | 0.8550619 | TAM I early vs naïve |
| *Psme1* | 1.3408657052390138e-6 | 0.85232513 | TAM I early vs naïve |
| *Fgl2* | 7.253691617150215e-13 | 0.84671622 | TAM I early vs naïve |
| *Rpl3-ps1* | 2.547655838831198e-6 | 0.84659786 | TAM I early vs naïve |
| *Gm9385* | 4.874778685920514e-7 | 0.84501461 | TAM I early vs naïve |
| *Cdkn1a* | 3.7089388927307503e-10 | 0.84315965 | TAM I early vs naïve |
| *Tlr2* | 5.885426063388716e-10 | 0.84013881 | TAM I early vs naïve |
| *Cyba* | 2.8315485598347522e-6 | 0.83948917 | TAM I early vs naïve |
| *Slfn2* | 1.675574554159989e-8 | 0.83464239 | TAM I early vs naïve |
| *Ptgs2* | 3.199571851208624e-8 | 0.83165748 | TAM I early vs naïve |
| *Tspo* | 3.444876746351964e-10 | 0.82109291 | TAM I early vs naïve |
| *Sh3bgrl3* | 3.3658306809061473e-6 | 0.81797114 | TAM I early vs naïve |
| *Slc11a1* | 1.528632083293601e-5 | 0.81339094 | TAM I early vs naïve |
| *Cxcl16* | 6.3111751215982936e-9 | 0.80854917 | TAM I early vs naïve |
| *Pkm* | 2.7672080589886397e-6 | 0.80582249 | TAM I early vs naïve |
| *AW112010* | 3.095385097113047e-9 | 0.79847607 | TAM I early vs naïve |
| *Rps26-ps1* | 4.558789666167049e-9 | 0.7956178 | TAM I early vs naïve |
| *Cstb* | 5.533586476186773e-7 | 0.79539316 | TAM I early vs naïve |
| *Actb* | 4.4074652845215126e-8 | 0.79167578 | TAM I early vs naïve |
| *Tmsb10* | 3.941252807397093e-9 | 0.78392469 | TAM I early vs naïve |
| *Sat1* | 1.4356785972578095e-4 | 0.7808057 | TAM I early vs naïve |
| *Iigp1* | 5.728232573400017e-11 | 0.77904574 | TAM I early vs naïve |
| *Tnfaip3* | 2.8881398648793373e-8 | 0.77733601 | TAM I early vs naïve |
| *Ifrd1* | 5.2975499648929255e-9 | 0.77625343 | TAM I early vs naïve |
| *Ccl2* | 3.552665488178814e-8 | 0.76904388 | TAM I early vs naïve |
| *Lat2* | 1.0364461362946807e-7 | 0.76688519 | TAM I early vs naïve |
| *Vim* | 5.236370047549526e-8 | 0.76687173 | TAM I early vs naïve |
| *Serbp1* | 1.4396052393822983e-5 | 0.76650407 | TAM I early vs naïve |
| *Lgals3* | 7.4043532049879774e-9 | 0.74493472 | TAM I early vs naïve |
| *Psmb9* | 1.0380734320327733e-10 | 0.73847808 | TAM I early vs naïve |
| *Rps3a1* | 4.578846988058938e-6 | 0.73578021 | TAM I early vs naïve |
| *H2-DMb1* | 1.409825807492985e-10 | 0.73076204 | TAM I early vs naïve |
| *Spp1* | 7.149960902391902e-7 | 0.73005755 | TAM I early vs naïve |
| *Gm10073* | 9.983600749607742e-6 | 0.72855463 | TAM I early vs naïve |
| *Cd81* | 1.722593924168712e-6 | 0.72855019 | TAM I early vs naïve |
| *Pfdn5* | 7.582505590269834e-8 | 0.72837284 | TAM I early vs naïve |
| *2010107E04Rik* | 2.4111745597184593e-9 | 0.72566233 | TAM I early vs naïve |
| *Rps10* | 2.6011579880835876e-6 | 0.72522895 | TAM I early vs naïve |
| *Eprs* | 4.599473270817122e-8 | 0.72417301 | TAM I early vs naïve |
| *Hspe1* | 2.9867721990143025e-7 | 0.72254307 | TAM I early vs naïve |
| *Bag1* | 4.293546466086643e-8 | 0.71922404 | TAM I early vs naïve |
| *Hif1a* | 8.281914151211335e-6 | 0.71880712 | TAM I early vs naïve |
| *Rpl18-ps1* | 3.984550415932506e-6 | 0.71660739 | TAM I early vs naïve |
| *Psme2b* | 1.7113849415215463e-6 | 0.71406305 | TAM I early vs naïve |
| *Hnrnpu* | 1.0418431002024502e-5 | 0.70787549 | TAM I early vs naïve |
| *Fcgr2b* | 2.3138622419488905e-6 | 0.70645964 | TAM I early vs naïve |
| *Fam46c* | 8.452729980512037e-7 | 0.70499164 | TAM I early vs naïve |
| *Rps11* | 5.864863193937167e-5 | 0.70364612 | TAM I early vs naïve |
| *Fosb* | 2.645367680326986e-5 | 0.69956204 | TAM I early vs naïve |
| *Ybx1* | 9.261170575928455e-5 | 0.69532783 | TAM I early vs naïve |
| *Prdx5* | 7.685426671233407e-5 | 0.692761 | TAM I early vs naïve |
| *Rpl37* | 1.656617896944591e-7 | 0.69180734 | TAM I early vs naïve |
| *Slc25a5* | 4.7307796303026135e-5 | 0.69160205 | TAM I early vs naïve |
| *Gm15500* | 1.571132775385076e-5 | 0.69103204 | TAM I early vs naïve |
| *Cox6b1* | 2.6173195706062757e-5 | 0.68938063 | TAM I early vs naïve |
| *Junb* | 0.0012174068202391356 | 0.6892777 | TAM I early vs naïve |
| *Cotl1* | 1.7718942333803212e-5 | 0.68916452 | TAM I early vs naïve |
| *Rrbp1* | 4.077008737295818e-5 | 0.68872825 | TAM I early vs naïve |
| *Kif5b* | 7.974937604966649e-8 | 0.6884824 | TAM I early vs naïve |
| *Myo5a* | 1.0461458775531898e-9 | 0.6878073 | TAM I early vs naïve |
| *Rpl10a-ps1* | 2.5876231476283085e-6 | 0.68647927 | TAM I early vs naïve |
| *Il2rg* | 1.8499075317882803e-8 | 0.68600934 | TAM I early vs naïve |
| *Txn1* | 1.4422535521490512e-7 | 0.68083133 | TAM I early vs naïve |
| *Anp32b* | 4.601302090671239e-6 | 0.68046258 | TAM I early vs naïve |
| *Nampt* | 2.2576550476840665e-8 | 0.67885415 | TAM I early vs naïve |
| *Rpl31-ps8* | 5.109097843505832e-6 | 0.67617857 | TAM I early vs naïve |
| *Tubb5* | 2.0340152608142687e-6 | 0.67598726 | TAM I early vs naïve |
| *Calm1* | 3.7679449706895534e-4 | 0.67299994 | TAM I early vs naïve |
| *Id2* | 2.408354406551749e-4 | 0.67222839 | TAM I early vs naïve |
| *Rplp2* | 6.715124418750849e-5 | 0.66836845 | TAM I early vs naïve |
| *Tpt1-ps3* | 4.192781274209192e-6 | 0.66756711 | TAM I early vs naïve |
| *Msn* | 1.631925740024434e-4 | 0.66720386 | TAM I early vs naïve |
| *Pdia3* | 4.462803850014923e-4 | 0.66432561 | TAM I early vs naïve |
| *Sub1* | 2.4986741199601443e-7 | 0.66203262 | TAM I early vs naïve |
| *Fxyd5* | 5.073463156970538e-8 | 0.65812636 | TAM I early vs naïve |
| *Rpl13* | 1.569534404275414e-7 | 0.6568936 | TAM I early vs naïve |
| *Ptprc* | 3.344967791547884e-5 | 0.65672494 | TAM I early vs naïve |
| *Rpl36a-ps2* | 7.080113095892818e-5 | 0.65635158 | TAM I early vs naïve |
| *Zfp106* | 1.1794394074555727e-7 | 0.65332337 | TAM I early vs naïve |
| *Gm6030* | 4.6055578350688574e-7 | 0.65210742 | TAM I early vs naïve |
| *Lamp1* | 0.0021259692472745885 | 0.6478405 | TAM I early vs naïve |
| *Mif* | 4.486067768211669e-7 | 0.63884809 | TAM I early vs naïve |
| *Atox1* | 2.8625594586865337e-5 | 0.63725409 | TAM I early vs naïve |
| *Rpl3* | 1.8810300501069032e-6 | 0.63701329 | TAM I early vs naïve |
| *Csf1* | 1.1037134981375911e-5 | 0.63662686 | TAM I early vs naïve |
| *Rpl38-ps2* | 2.61995575478238e-7 | 0.63656925 | TAM I early vs naïve |
| *Arpc1b* | 1.9135310249620568e-4 | 0.63178649 | TAM I early vs naïve |
| *Rpl37a* | 1.43167186844608e-8 | 0.63172864 | TAM I early vs naïve |
| *Akr1a1* | 4.388647706106645e-5 | 0.63104732 | TAM I early vs naïve |
| *Lcp1* | 1.0177898363530466e-4 | 0.63048673 | TAM I early vs naïve |
| *App* | 1.2593075800935476e-4 | 0.62943874 | TAM I early vs naïve |
| *Mrpl52* | 7.495724105548996e-8 | 0.62845273 | TAM I early vs naïve |
| *Gm6204* | 1.3798694756100887e-4 | 0.62771202 | TAM I early vs naïve |
| *Itgb2* | 9.413231191807776e-6 | 0.62613908 | TAM I early vs naïve |
| *Tpd52* | 1.18719052148397e-6 | 0.62499458 | TAM I early vs naïve |
| *Ptms* | 4.918031899825085e-5 | 0.62412194 | TAM I early vs naïve |
| *Rel* | 2.5625347364378816e-4 | 0.62335961 | TAM I early vs naïve |
| *Lrrfip1* | 7.867172948033391e-7 | 0.62237625 | TAM I early vs naïve |
| *Tapbp* | 3.917364967785117e-7 | 0.6220356 | TAM I early vs naïve |
| *Rpl9-ps6* | 7.350046819868788e-7 | 0.61835909 | TAM I early vs naïve |
| *Sqstm1* | 9.468367386748251e-5 | 0.61737784 | TAM I early vs naïve |
| *Litaf* | 4.993147393275141e-7 | 0.61673809 | TAM I early vs naïve |
| *Eif3c* | 2.1863840078467293e-6 | 0.61484828 | TAM I early vs naïve |
| *Gatm* | 1.3299413141878369e-6 | 0.61384042 | TAM I early vs naïve |
| *Gm10076* | 2.982578666923811e-9 | 0.6121793 | TAM I early vs naïve |
| *Fos* | 0.009561287506771286 | 0.61195074 | TAM I early vs naïve |
| *Uqcrh* | 6.370389298647725e-6 | 0.61116521 | TAM I early vs naïve |
| *Canx* | 3.6530727290582293e-4 | 0.60632676 | TAM I early vs naïve |
| *Hspa5* | 0.00165443526458099 | 0.60483117 | TAM I early vs naïve |
| *Dek* | 2.566675528566215e-4 | 0.60388584 | TAM I early vs naïve |
| *Tmbim6* | 3.116003250400716e-4 | 0.6016218 | TAM I early vs naïve |
| *Shfm1* | 5.530349601678274e-5 | 0.60039089 | TAM I early vs naïve |
| *Atpif1* | 1.6995924482204072e-4 | 0.59970473 | TAM I early vs naïve |
| *Skil* | 1.542126142942568e-4 | 0.5982339 | TAM I early vs naïve |
| *Rps15a-ps5* | 1.1584178596629152e-6 | 0.59634557 | TAM I early vs naïve |
| *Irf1* | 8.921273205868588e-6 | 0.59483756 | TAM I early vs naïve |
| *Tgm2* | 2.6146139365725016e-6 | 0.59296943 | TAM I early vs naïve |
| *Nlrp3* | 6.507595649101439e-8 | 0.59100869 | TAM I early vs naïve |
| *Hspa8* | 2.6815667267250176e-4 | 0.5868086 | TAM I early vs naïve |
| *Smarca5* | 1.0843283044383585e-6 | 0.58470299 | TAM I early vs naïve |
| *Ifi30* | 6.354978986008614e-7 | 0.58242331 | TAM I early vs naïve |
| *Bcl2a1a* | 3.1531231916875556e-8 | 0.58159143 | TAM I early vs naïve |
| *Icam1* | 3.1515744211569794e-4 | 0.58012671 | TAM I early vs naïve |
| *Pfn1* | 8.770432995942512e-5 | 0.57924746 | TAM I early vs naïve |
| *Capg* | 1.0759755335470456e-7 | 0.57852147 | TAM I early vs naïve |
| *Tm9sf3* | 7.598958246585939e-5 | 0.57810334 | TAM I early vs naïve |
| *Serp1* | 8.63768878123525e-6 | 0.57805925 | TAM I early vs naïve |
| *Gabarap* | 6.314861561722545e-5 | 0.57640203 | TAM I early vs naïve |
| *Tnf* | 1.876125762241561e-8 | 0.57633297 | TAM I early vs naïve |
| *Efhd2* | 3.7273886047662616e-5 | 0.57460576 | TAM I early vs naïve |
| *Cd36* | 2.4813405859293133e-6 | 0.57307037 | TAM I early vs naïve |
| *Lgmn* | 4.105946770244317e-4 | 0.56877449 | TAM I early vs naïve |
| *Calr* | 0.0039744759798355745 | 0.56315954 | TAM I early vs naïve |
| *Serpine1* | 1.4317448872935295e-4 | 0.56290767 | TAM I early vs naïve |
| *Gm8276* | 2.1385065950104697e-4 | 0.56175762 | TAM I early vs naïve |
| *Gm12254* | 1.0514576091923553e-5 | 0.56170581 | TAM I early vs naïve |
| *Itm2b* | 0.0029063005620152487 | 0.55912793 | TAM I early vs naïve |
| *Aplp2* | 4.8018733489175424e-5 | 0.55726547 | TAM I early vs naïve |
| *Scpep1* | 2.2340505674242167e-5 | 0.55463054 | TAM I early vs naïve |
| *Oxct1* | 0.00025882515942259276 | 0.55447344 | TAM I early vs naïve |
| *Slc25a3* | 9.845226264564412e-6 | 0.55326462 | TAM I early vs naïve |
| *Eif4g1* | 1.4469420160076681e-5 | 0.55245736 | TAM I early vs naïve |
| *Gm14513* | 2.149900890522013e-6 | 0.55240012 | TAM I early vs naïve |
| *Rps10-ps2* | 5.503000251771733e-6 | 0.55202583 | TAM I early vs naïve |
| *Gm43712* | 5.1024945609554155e-6 | 0.55174216 | TAM I early vs naïve |
| *BC005537* | 2.563908203604597e-4 | 0.55133773 | TAM I early vs naïve |
| *Pomp* | 2.25514525049421e-5 | 0.55093149 | TAM I early vs naïve |
| *Gm8730* | 7.353324895369936e-7 | 0.54896978 | TAM I early vs naïve |
| *Rpl36* | 7.5561897657573845e-6 | 0.54809137 | TAM I early vs naïve |
| *Pdcd6ip* | 1.9432495171377176e-7 | 0.54771366 | TAM I early vs naïve |
| *Gm1966* | 2.2972709697701998e-8 | 0.54590277 | TAM I early vs naïve |
| *Ppt1* | 1.0729691331687268e-4 | 0.54550154 | TAM I early vs naïve |
| *Ldha* | 1.6347937382947037e-6 | 0.54530348 | TAM I early vs naïve |
| *Atp5e* | 5.266230485118214e-5 | 0.54332483 | TAM I early vs naïve |
| *Hnrnpab* | 7.557925623970476e-5 | 0.54148164 | TAM I early vs naïve |
| *Ncf1* | 0.0016532284773921452 | 0.540661 | TAM I early vs naïve |
| *Sh3glb1* | 5.251291939179645e-4 | 0.53994766 | TAM I early vs naïve |
| *Rpl36al* | 2.5824043638091034e-5 | 0.53865982 | TAM I early vs naïve |
| *Arl5c* | 6.664882690469689e-6 | 0.5386022 | TAM I early vs naïve |
| *Etf1* | 3.28411844321732e-7 | 0.53829345 | TAM I early vs naïve |
| *Atp6v1f* | 3.7941781774511674e-6 | 0.53821446 | TAM I early vs naïve |
| *Usp8* | 2.261391665578596e-5 | 0.53673663 | TAM I early vs naïve |
| *Gm14681* | 4.5208886577768995e-6 | 0.53594211 | TAM I early vs naïve |
| *Ranbp2* | 2.8864754332894146e-6 | 0.53576087 | TAM I early vs naïve |
| *Tnfaip2* | 1.2949110646256196e-5 | 0.5338899 | TAM I early vs naïve |
| *Zfas1* | 7.068795760224574e-4 | 0.53042606 | TAM I early vs naïve |
| *Maff* | 7.65194812704391e-10 | 0.52980223 | TAM I early vs naïve |
| *Gna13* | 3.2598543284068634e-5 | 0.52779917 | TAM I early vs naïve |
| *Caprin1* | 2.7049793594641975e-6 | 0.52603321 | TAM I early vs naïve |
| *Psme2* | 3.898495566958443e-6 | 0.52317889 | TAM I early vs naïve |
| *Cntrl* | 7.113973402542731e-5 | 0.52235507 | TAM I early vs naïve |
| *Vcam1* | 1.1978114798077272e-4 | 0.52233727 | TAM I early vs naïve |
| *Chd7* | 1.546275398998348e-5 | 0.52230149 | TAM I early vs naïve |
| *Golga4* | 9.58754098686116e-5 | 0.52146712 | TAM I early vs naïve |
| *Cd274* | 9.250316133005176e-9 | 0.52091505 | TAM I early vs naïve |
| *Eif3f* | 1.0209493607561234e-5 | 0.51898938 | TAM I early vs naïve |
| *Pim1* | 3.0041770160979195e-7 | 0.51737524 | TAM I early vs naïve |
| *Shisa5* | 5.036424797607788e-5 | 0.51595579 | TAM I early vs naïve |
| *Gnas* | 0.005612221962770707 | 0.51545435 | TAM I early vs naïve |
| *Rps24-ps2* | 1.6504502946124368e-6 | 0.51449829 | TAM I early vs naïve |
| *Axl* | 3.302761946428481e-9 | 0.5142209 | TAM I early vs naïve |
| *Eif5* | 5.342212498071316e-4 | 0.51329266 | TAM I early vs naïve |
| *Pfkfb3* | 9.097984631419309e-6 | 0.51290185 | TAM I early vs naïve |
| *Anxa3* | 3.59871716275079e-4 | 0.51284178 | TAM I early vs naïve |
| *Rbm25* | 0.0030324132335353355 | 0.51167911 | TAM I early vs naïve |
| *Gm6863* | 1.9480116623564e-4 | 0.50867129 | TAM I early vs naïve |
| *Os9* | 7.34970339981873e-4 | 0.50654013 | TAM I early vs naïve |
| *Rps25* | 1.97811941560399e-7 | 0.50624353 | TAM I early vs naïve |
| *Cpd* | 3.4084700200052103e-6 | 0.50531062 | TAM I early vs naïve |
| *Plekho2* | 8.462519237329534e-9 | 0.50507508 | TAM I early vs naïve |
| *Trim30a* | 2.165141671069976e-4 | 0.50482914 | TAM I early vs naïve |
| *Pld3* | 0.0012588440957578908 | 0.50357466 | TAM I early vs naïve |
| *Ccl4* | 2.015828573205592e-8 | 3.10092526 | TAM I late versus naïve |
| *Ccl3* | 6.445690422095445e-8 | 2.63727185 | TAM I late versus naïve |
| *Fth1* | 1.631237742037217e-8 | 2.39894815 | TAM I late versus naïve |
| *Ctsb* | 1.2269280413657366e-7 | 2.39535113 | TAM I late versus naïve |
| *Ccl12* | 8.562675061905351e-11 | 2.2124261 | TAM I late versus naïve |
| *Nfkbia* | 4.2847120568682757e-7 | 2.16913644 | TAM I late versus naïve |
| *H2-K1* | 1.1075495239616788e-7 | 2.10120733 | TAM I late versus naïve |
| *H2-D1* | 8.402713358974549e-6 | 2.01112269 | TAM I late versus naïve |
| *Rplp1* | 6.443969049277934e-11 | 1.98060656 | TAM I late versus naïve |
| *Rpl32* | 1.8797629010386647e-8 | 1.95200716 | TAM I late versus naïve |
| *Rpl8* | 5.417520327377327e-10 | 1.91379714 | TAM I late versus naïve |
| *Rps3* | 1.4418332878335172e-10 | 1.91360738 | TAM I late versus naïve |
| *Srgn* | 1.244279142695939e-8 | 1.88755385 | TAM I late versus naïve |
| *B2m* | 2.28446083737233e-5 | 1.88430299 | TAM I late versus naïve |
| *Cd83* | 2.3271723726625506e-8 | 1.86150617 | TAM I late versus naïve |
| *Atf3* | 3.0672163806116918e-6 | 1.84681663 | TAM I late versus naïve |
| *Plek* | 8.528542322664166e-8 | 1.84506596 | TAM I late versus naïve |
| *Ccl2* | 9.298741668571493e-11 | 1.83132664 | TAM I late versus naïve |
| *Ifi27l2a* | 3.0992969975970636e-13 | 1.81438948 | TAM I late versus naïve |
| *Ctsz* | 9.601496646310691e-6 | 1.80671104 | TAM I late versus naïve |
| *Grn* | 1.166380471110127e-6 | 1.76588599 | TAM I late versus naïve |
| *Cd63* | 1.3442903043266733e-6 | 1.76435732 | TAM I late versus naïve |
| *Lyz2* | 9.619880803592824e-8 | 1.7344666 | TAM I late versus naïve |
| *Nfkbiz* | 2.0806172540700408e-8 | 1.73438717 | TAM I late versus naïve |
| *Bst2* | 9.982958612701194e-11 | 1.67609494 | TAM I late versus naïve |
| *Rps20* | 7.960816828744973e-10 | 1.64997912 | TAM I late versus naïve |
| *Cd52* | 2.089443450709271e-6 | 1.63688111 | TAM I late versus naïve |
| *Ly86* | 5.188206868427892e-7 | 1.6204145 | TAM I late versus naïve |
| *Rpl4* | 2.0458944318539687e-8 | 1.61833617 | TAM I late versus naïve |
| *Fcer1g* | 6.323124262193324e-6 | 1.60818671 | TAM I late versus naïve |
| *Cd14* | 1.0735501512104797e-4 | 1.59893302 | TAM I late versus naïve |
| *Tmsb4x* | 1.9386629470964544e-8 | 1.59713358 | TAM I late versus naïve |
| *Ncl* | 3.3316538964954506e-6 | 1.57294165 | TAM I late versus naïve |
| *Rps26* | 1.7226016607869296e-7 | 1.56873794 | TAM I late versus naïve |
| *Ly6e* | 2.52329504920362e-6 | 1.56536052 | TAM I late versus naïve |
| *Wdr89* | 2.6374932357276685e-8 | 1.55030058 | TAM I late versus naïve |
| *Chd4* | 5.3802331341071055e-6 | 1.54938699 | TAM I late versus naïve |
| *Tnf* | 5.4339529493597654e-17 | 1.54632786 | TAM I late versus naïve |
| *Il1b* | 2.4023341451039032e-11 | 1.52427449 | TAM I late versus naïve |
| *Rplp0* | 2.4685387693710455e-8 | 1.51568092 | TAM I late versus naïve |
| *Cox4i1* | 2.890020301577688e-8 | 1.51202199 | TAM I late versus naïve |
| *Ctsl* | 2.3999022511815656e-5 | 1.51106709 | TAM I late versus naïve |
| *Il1a* | 9.794295697107595e-13 | 1.49708011 | TAM I late versus naïve |
| *Ifitm3* | 1.660016405545603e-11 | 1.49627249 | TAM I late versus naïve |
| *Lgals3bp* | 1.2125201634715153e-8 | 1.4846535 | TAM I late versus naïve |
| *Ctsd* | 1.1588987941731798e-5 | 1.48422521 | TAM I late versus naïve |
| *C1qc* | 2.2354787459319552e-7 | 1.47826505 | TAM I late versus naïve |
| *Lgmn* | 1.0228892503884607e-7 | 1.46798339 | TAM I late versus naïve |
| *Egr1* | 4.498580061281805e-5 | 1.46521256 | TAM I late versus naïve |
| *C1qa* | 1.6310280216119945e-7 | 1.46091599 | TAM I late versus naïve |
| *Rpl39* | 1.0487514518454462e-6 | 1.45561965 | TAM I late versus naïve |
| *Gm9794* | 6.774792464621868e-7 | 1.44173893 | TAM I late versus naïve |
| *Hsp90ab1* | 4.560079722315628e-6 | 1.43177001 | TAM I late versus naïve |
| *Rps5* | 4.018848384946615e-6 | 1.42062978 | TAM I late versus naïve |
| *Slfn2* | 2.128455202505065e-9 | 1.41679975 | TAM I late versus naïve |
| *Tyrobp* | 3.138109656077386e-6 | 1.41549509 | TAM I late versus naïve |
| *Rack1* | 3.9725988881327846e-8 | 1.40289599 | TAM I late versus naïve |
| *Gm5963* | 4.6110863036940683e-7 | 1.39889898 | TAM I late versus naïve |
| *Rps14* | 1.4230235295494077e-6 | 1.38824167 | TAM I late versus naïve |
| *Arpc1b* | 6.681018728885019e-8 | 1.36727893 | TAM I late versus naïve |
| *Actb* | 3.73593055073671e-8 | 1.35329798 | TAM I late versus naïve |
| *Cd63-ps* | 1.999751226336057e-5 | 1.34074685 | TAM I late versus naïve |
| *Sqstm1* | 3.887301246136359e-11 | 1.33876785 | TAM I late versus naïve |
| *C1qb* | 2.2690740660017456e-7 | 1.33759294 | TAM I late versus naïve |
| *Cox6a1* | 9.556343990605753e-9 | 1.32927002 | TAM I late versus naïve |
| *Npc2* | 1.3636651634619845e-4 | 1.32234547 | TAM I late versus naïve |
| *Cstb* | 1.2212909646359314e-9 | 1.3123011 | TAM I late versus naïve |
| *Cfl1* | 3.548665314312934e-6 | 1.30479206 | TAM I late versus naïve |
| *Lgals1* | 1.0401693235048634e-13 | 1.29698404 | TAM I late versus naïve |
| *Mcl1* | 1.0841850887681435e-6 | 1.28967753 | TAM I late versus naïve |
| *Hspa5* | 4.053601419902298e-4 | 1.28078498 | TAM I late versus naïve |
| *Lilrb4a* | 2.5317396774893853e-11 | 1.27856928 | TAM I late versus naïve |
| *Rpsa-ps10* | 1.9501653230247886e-7 | 1.27739319 | TAM I late versus naïve |
| *Rps19* | 1.393922246232078e-6 | 1.27286166 | TAM I late versus naïve |
| *Rps21* | 3.7722462042435235e-7 | 1.27211565 | TAM I late versus naïve |
| *Cox6c* | 6.995590431437438e-7 | 1.26333153 | TAM I late versus naïve |
| *Rps10-ps1* | 2.389099263847755e-8 | 1.26315267 | TAM I late versus naïve |
| *Gpx1* | 4.835747871211136e-7 | 1.25989921 | TAM I late versus naïve |
| *Rpl14* | 3.9088241752440323e-7 | 1.2520687 | TAM I late versus naïve |
| *Ifrd1* | 1.892907452718559e-7 | 1.24797954 | TAM I late versus naïve |
| *Rpl34* | 6.2731485538944545e-6 | 1.23155769 | TAM I late versus naïve |
| *Tuba1b* | 6.376059648885733e-7 | 1.22946845 | TAM I late versus naïve |
| *Gadd45b* | 9.22450887266874e-13 | 1.22632009 | TAM I late versus naïve |
| *Ctsc* | 6.0363035334375265e-6 | 1.22566001 | TAM I late versus naïve |
| *Rps15* | 5.93521584665487e-7 | 1.22264431 | TAM I late versus naïve |
| *Bcl2a1b* | 4.281202238630403e-6 | 1.20227624 | TAM I late versus naïve |
| *Ier3* | 3.146420773795607e-6 | 1.19342517 | TAM I late versus naïve |
| *Lamp1* | 6.840455204787423e-5 | 1.1932437 | TAM I late versus naïve |
| *Txn1* | 8.195564855257867e-12 | 1.19166103 | TAM I late versus naïve |
| *Cebpb* | 8.970483043261674e-5 | 1.1890636 | TAM I late versus naïve |
| *Sdcbp* | 6.008158811328331e-8 | 1.18837839 | TAM I late versus naïve |
| *Pfn1* | 5.6480500296020365e-5 | 1.18238991 | TAM I late versus naïve |
| *Clta* | 4.789905484416674e-6 | 1.17709699 | TAM I late versus naïve |
| *Eef2* | 1.0682224674894076e-8 | 1.17679157 | TAM I late versus naïve |
| *Ifi30* | 6.232602910360381e-9 | 1.17146116 | TAM I late versus naïve |
| *Calr* | 8.982492498808608e-4 | 1.16997887 | TAM I late versus naïve |
| *Rps24* | 6.586291707878019e-6 | 1.16827712 | TAM I late versus naïve |
| *Rpl41* | 2.755604096616096e-7 | 1.16574913 | TAM I late versus naïve |
| *Akr1a1* | 6.348855612660492e-6 | 1.16555713 | TAM I late versus naïve |
| *Gm9843* | 2.2159725424877306e-5 | 1.16310612 | TAM I late versus naïve |
| *Uqcrq* | 6.829005593629078e-8 | 1.1608895 | TAM I late versus naïve |
| *Gm14303* | 1.7508432142350003e-6 | 1.15800014 | TAM I late versus naïve |
| *Gusb* | 6.308441582401804e-8 | 1.15373161 | TAM I late versus naïve |
| *Ifi204* | 8.226898236848111e-10 | 1.14515134 | TAM I late versus naïve |
| *Gm10288* | 7.934444333263635e-7 | 1.13822435 | TAM I late versus naïve |
| *Nr4a1* | 1.6295223726835772e-9 | 1.13526065 | TAM I late versus naïve |
| *Cdkn1a* | 9.19603359318903e-8 | 1.13449925 | TAM I late versus naïve |
| *Rps18* | 1.592356672028703e-7 | 1.1342377 | TAM I late versus naïve |
| *Sh3bgrl3* | 1.2541848327832607e-5 | 1.12322798 | TAM I late versus naïve |
| *Mif* | 9.53617578014606e-10 | 1.12193964 | TAM I late versus naïve |
| *Tubb5* | 2.489893854403498e-7 | 1.11447876 | TAM I late versus naïve |
| *Sat1* | 3.5316001313173066e-5 | 1.1103075 | TAM I late versus naïve |
| *Zfp36* | 0.0014151207747178955 | 1.10807388 | TAM I late versus naïve |
| *Ubc* | 2.675056100610186e-4 | 1.10009909 | TAM I late versus naïve |
| *Hint1* | 1.3968528852245067e-7 | 1.09952316 | TAM I late versus naïve |
| *Gm5805* | 1.6452564280919745e-5 | 1.09775961 | TAM I late versus naïve |
| *Psma2* | 2.586060243952164e-8 | 1.09694152 | TAM I late versus naïve |
| *Gm10275* | 2.142236455638965e-6 | 1.09568106 | TAM I late versus naïve |
| *Cyba* | 8.544714394923205e-5 | 1.09498452 | TAM I late versus naïve |
| *C3ar1* | 1.2792727880535506e-6 | 1.09333883 | TAM I late versus naïve |
| *Ccrl2* | 1.4238598946225637e-6 | 1.09074277 | TAM I late versus naïve |
| *Rps13-ps2* | 1.3434368593908019e-5 | 1.09010146 | TAM I late versus naïve |
| *Pabpc1* | 1.0512345555987825e-5 | 1.08446062 | TAM I late versus naïve |
| *Ybx1* | 1.3759619718287967e-4 | 1.08325412 | TAM I late versus naïve |
| *Pkm* | 1.0644594675473765e-4 | 1.07917148 | TAM I late versus naïve |
| *Npm1* | 1.1995435961242007e-6 | 1.06799123 | TAM I late versus naïve |
| *Cd9* | 3.483830246195131e-4 | 1.06514841 | TAM I late versus naïve |
| *Aif1* | 2.0319755434481112e-4 | 1.06325861 | TAM I late versus naïve |
| *Cotl1* | 1.3078079172077076e-5 | 1.06176675 | TAM I late versus naïve |
| *Cd68* | 2.1547759268238833e-5 | 1.05574725 | TAM I late versus naïve |
| *Lcp1* | 6.811589234938206e-5 | 1.05260672 | TAM I late versus naïve |
| *Cxcl10* | 3.748810076477956e-9 | 1.0521577 | TAM I late versus naïve |
| *Junb* | 0.004111751987952254 | 1.05141803 | TAM I late versus naïve |
| *Hnrnpa2b1* | 1.6653359745688094e-4 | 1.04105592 | TAM I late versus naïve |
| *Aldoa* | 2.194870255412527e-5 | 1.03152154 | TAM I late versus naïve |
| *Timp2* | 4.581469819301606e-5 | 1.0288827 | TAM I late versus naïve |
| *Rps24-ps3* | 5.589849808347063e-6 | 1.02649908 | TAM I late versus naïve |
| *Lgals9* | 5.950264403057719e-7 | 1.02362482 | TAM I late versus naïve |
| *Mpeg1* | 2.2831776517516394e-4 | 1.01924776 | TAM I late versus naïve |
| *Anp32b* | 2.116923783241127e-6 | 1.017585 | TAM I late versus naïve |
| *Rps9* | 2.1144079563868037e-4 | 1.01263233 | TAM I late versus naïve |
| *Tspo* | 4.943183028244536e-9 | 1.00449685 | TAM I late versus naïve |
| *Oaz1* | 1.216472652194222e-7 | 1.00215159 | TAM I late versus naïve |
| *Pdia6* | 3.05358951907952e-5 | 1.00077702 | TAM I late versus naïve |
| *Ndufa4* | 3.7903454696410026e-8 | 1.00039862 | TAM I late versus naïve |
| *Itm2b* | 1.1474673391266986e-4 | 0.9981153 | TAM I late versus naïve |
| *Minos1* | 2.949634001395313e-7 | 0.9979884 | TAM I late versus naïve |
| *Tlr2* | 4.5882278783502925e-8 | 0.99006302 | TAM I late versus naïve |
| *Tnfaip3* | 5.167935848047842e-6 | 0.98735746 | TAM I late versus naïve |
| *Dek* | 7.883887344139646e-5 | 0.98292211 | TAM I late versus naïve |
| *Erp29* | 2.8820986128187096e-4 | 0.98205054 | TAM I late versus naïve |
| *Rpl22* | 9.768033909174072e-4 | 0.98076999 | TAM I late versus naïve |
| *Ptms* | 7.19785857524732e-5 | 0.97266878 | TAM I late versus naïve |
| *Atp5b* | 7.290752124437671e-6 | 0.97158848 | TAM I late versus naïve |
| *Rps15a* | 5.105282529547784e-6 | 0.97089281 | TAM I late versus naïve |
| *Ctsh* | 1.4553757074959443e-5 | 0.96710463 | TAM I late versus naïve |
| *Rpl37* | 1.4195090547440426e-4 | 0.96579916 | TAM I late versus naïve |
| *Rps8* | 7.895179317471517e-6 | 0.96243004 | TAM I late versus naïve |
| *Slc25a5* | 2.025075778601918e-4 | 0.96130046 | TAM I late versus naïve |
| *Atox1* | 1.1968214836874022e-5 | 0.96042612 | TAM I late versus naïve |
| *Eif3a* | 6.740909819444547e-5 | 0.957305 | TAM I late versus naïve |
| *Rps2* | 1.6784284439081356e-4 | 0.95682466 | TAM I late versus naïve |
| *Slc25a3* | 1.450630145428484e-5 | 0.95200011 | TAM I late versus naïve |
| *Rpl9-ps6* | 5.949059419082753e-8 | 0.94792804 | TAM I late versus naïve |
| *Pycard* | 2.5952007736076104e-5 | 0.94783237 | TAM I late versus naïve |
| *Bag1* | 5.9381255220456144e-5 | 0.94757859 | TAM I late versus naïve |
| *Gabarap* | 2.4093457724070084e-4 | 0.94714672 | TAM I late versus naïve |
| *Uqcr10* | 9.441001340197992e-8 | 0.9460093 | TAM I late versus naïve |
| *Serbp1* | 0.002362765114141644 | 0.9393805 | TAM I late versus naïve |
| *Cox6b1* | 4.18614071500223e-5 | 0.93698795 | TAM I late versus naïve |
| *Tagln2* | 2.5377376408424464e-8 | 0.93667988 | TAM I late versus naïve |
| *Atp6ap2* | 6.643846611106441e-6 | 0.9241861 | TAM I late versus naïve |
| *Gas5* | 4.774652102372013e-5 | 0.92349538 | TAM I late versus naïve |
| *Gnas* | 1.0836176734596542e-4 | 0.92219859 | TAM I late versus naïve |
| *Rps25-ps1* | 2.8274102380994525e-5 | 0.92213169 | TAM I late versus naïve |
| *Cd81* | 2.387819801127991e-5 | 0.9206579 | TAM I late versus naïve |
| *Rpl26* | 8.025312900872553e-4 | 0.91811445 | TAM I late versus naïve |
| *Nme1* | 3.998907855592469e-10 | 0.91451899 | TAM I late versus naïve |
| *Cox7a2* | 1.091557586110475e-5 | 0.91238575 | TAM I late versus naïve |
| *Rpl23* | 1.0228310993873454e-4 | 0.90694326 | TAM I late versus naïve |
| *Cox7b* | 5.027731399932273e-6 | 0.90412908 | TAM I late versus naïve |
| *Prdx1* | 0.0015202458351833855 | 0.90357542 | TAM I late versus naïve |
| *Rps10* | 8.80919375792242e-6 | 0.90157268 | TAM I late versus naïve |
| *Gna13* | 1.2798424136173759e-6 | 0.9013333 | TAM I late versus naïve |
| *Arpc5* | 3.653399401095561e-4 | 0.89630379 | TAM I late versus naïve |
| *Pfdn5* | 2.4153332565388725e-6 | 0.89488258 | TAM I late versus naïve |
| *Rpsa* | 4.866554483178072e-7 | 0.89458543 | TAM I late versus naïve |
| *Tpd52* | 7.009472319419326e-7 | 0.88680289 | TAM I late versus naïve |
| *Nfe2l2* | 0.005097806141957751 | 0.88617533 | TAM I late versus naïve |
| *Rpl14-ps1* | 5.676845541677323e-4 | 0.88218376 | TAM I late versus naïve |
| *Osm* | 8.621805250602484e-5 | 0.88153929 | TAM I late versus naïve |
| *Ms4a6c* | 2.771031660338857e-7 | 0.88057693 | TAM I late versus naïve |
| *Trim30a* | 5.238611730168405e-4 | 0.88021396 | TAM I late versus naïve |
| *Slc15a3* | 6.0638313304683546e-5 | 0.87980818 | TAM I late versus naïve |
| *Eif5b* | 1.223801346383745e-4 | 0.87817091 | TAM I late versus naïve |
| *Atpif1* | 4.688896292687404e-4 | 0.86894923 | TAM I late versus naïve |
| *Shfm1* | 1.0999165494136123e-4 | 0.86472247 | TAM I late versus naïve |
| *Eif3c* | 7.500905802053315e-5 | 0.86304145 | TAM I late versus naïve |
| *Gm11478* | 0.0023064728503342864 | 0.86118536 | TAM I late versus naïve |
| *Eif2s2* | 9.920196960246852e-7 | 0.86082573 | TAM I late versus naïve |
| *Chchd2* | 9.006516555130744e-4 | 0.8575337 | TAM I late versus naïve |
| *Rpl36a-ps2* | 2.0423575988952867e-7 | 0.85733361 | TAM I late versus naïve |
| *Smc1a* | 2.2539378259723757e-7 | 0.84811586 | TAM I late versus naïve |
| *Litaf* | 5.2743826078227925e-6 | 0.8480904 | TAM I late versus naïve |
| *Hnrnpd* | 1.2724497266525435e-4 | 0.84335666 | TAM I late versus naïve |
| *Atp5e* | 2.5372533583848755e-6 | 0.84090877 | TAM I late versus naïve |
| *Actr3* | 8.654780523337843e-5 | 0.83936567 | TAM I late versus naïve |
| *Rps16-ps2* | 9.457780411637181e-5 | 0.83386963 | TAM I late versus naïve |
| *Ran* | 8.131088674591154e-7 | 0.83375048 | TAM I late versus naïve |
| *Fam46c* | 9.677460929858462e-6 | 0.83319685 | TAM I late versus naïve |
| *Cox8a* | 2.5890796071453213e-4 | 0.83221135 | TAM I late versus naïve |
| *Ckb* | 1.1605857759070881e-4 | 0.83190675 | TAM I late versus naïve |
| *Slc25a4* | 0.003079502053571653 | 0.83015156 | TAM I late versus naïve |
| *Hnrnpab* | 7.38742750133483e-4 | 0.82744364 | TAM I late versus naïve |
| *Tpr* | 0.002322224291109464 | 0.82552741 | TAM I late versus naïve |
| *Serp1* | 4.95197637638128e-5 | 0.82530003 | TAM I late versus naïve |
| *Gnai2* | 8.08593800507667e-5 | 0.8248938 | TAM I late versus naïve |
| *Ssr4* | 3.516924234179237e-4 | 0.82268815 | TAM I late versus naïve |
| *Ldha* | 2.7319718491824544e-6 | 0.82239327 | TAM I late versus naïve |
| *Rab1a* | 7.267461292893077e-4 | 0.82221298 | TAM I late versus naïve |
| *Cct3* | 1.1926283907048653e-7 | 0.81912763 | TAM I late versus naïve |
| *Npl* | 4.901557802807749e-9 | 0.81893277 | TAM I late versus naïve |
| *Vim* | 5.002717819818041e-9 | 0.81888866 | TAM I late versus naïve |
| *Atp5j* | 3.8108906447830305e-4 | 0.8182309 | TAM I late versus naïve |
| *Atp5d* | 3.002870917266609e-7 | 0.81811892 | TAM I late versus naïve |
| *Eif4a1* | 1.2701342604853695e-5 | 0.81367212 | TAM I late versus naïve |
| *Eef1b2* | 5.368855941569037e-5 | 0.81327803 | TAM I late versus naïve |
| *Dynlrb1* | 4.0612437862498164e-8 | 0.81235482 | TAM I late versus naïve |
| *Tln1* | 1.0494579598615379e-4 | 0.81234475 | TAM I late versus naïve |
| *Edf1* | 1.325675586258052e-6 | 0.81082198 | TAM I late versus naïve |
| *Csf1* | 3.4736854860606763e-7 | 0.80644943 | TAM I late versus naïve |
| *Id2* | 0.005410629771337691 | 0.79915431 | TAM I late versus naïve |
| *Gas6* | 2.6269914396660396e-4 | 0.79744467 | TAM I late versus naïve |
| *Ier2* | 2.317483342850563e-4 | 0.79574481 | TAM I late versus naïve |
| *Chmp4b* | 1.230039710418057e-4 | 0.79545072 | TAM I late versus naïve |
| *Msn* | 5.321556181590338e-4 | 0.79297296 | TAM I late versus naïve |
| *Atp1b3* | 6.61989522888258e-5 | 0.79269262 | TAM I late versus naïve |
| *Myl12a* | 2.038894003754029e-5 | 0.79164791 | TAM I late versus naïve |
| *Ssb* | 8.276822553813501e-4 | 0.79125682 | TAM I late versus naïve |
| *Socs3* | 0.001961035301233912 | 0.79001406 | TAM I late versus naïve |
| *Hnrnpu* | 0.006773107746089708 | 0.78698162 | TAM I late versus naïve |
| *Creg1* | 6.747983314896454e-4 | 0.78555416 | TAM I late versus naïve |
| *Tmem258* | 6.466936594034485e-6 | 0.78091754 | TAM I late versus naïve |
| *App* | 0.0056129925125565725 | 0.77774078 | TAM I late versus naïve |
| *Neat1* | 0.0017458288243064502 | 0.77690589 | TAM I late versus naïve |
| *Rpl35a* | 2.6640170761317545e-4 | 0.77638394 | TAM I late versus naïve |
| *Canx* | 0.0019565364821479054 | 0.77380269 | TAM I late versus naïve |
| *Pdia3* | 0.006863411513614116 | 0.77193309 | TAM I late versus naïve |
| *Eif5* | 5.66644792309224e-4 | 0.7706551 | TAM I late versus naïve |
| *Syngr1* | 6.737524142427914e-4 | 0.76948775 | TAM I late versus naïve |
| *Cdc42* | 7.783470432302153e-4 | 0.76690677 | TAM I late versus naïve |
| *Rpl31-ps8* | 1.475536248157476e-4 | 0.76205129 | TAM I late versus naïve |
| *Pdap1* | 7.067681516005666e-6 | 0.76124365 | TAM I late versus naïve |
| *Ik* | 6.071940015490186e-4 | 0.7600925 | TAM I late versus naïve |
| *Ifi207* | 4.100812040719716e-7 | 0.75862051 | TAM I late versus naïve |
| *Rnf130* | 1.456044096706049e-4 | 0.75633232 | TAM I late versus naïve |
| *Psma1* | 5.309969692690127e-5 | 0.75445321 | TAM I late versus naïve |
| *Manf* | 1.9947188453747788e-4 | 0.75139648 | TAM I late versus naïve |
| *Prdx5* | 5.262615478734031e-4 | 0.74982563 | TAM I late versus naïve |
| *Ppib* | 5.523162482870403e-5 | 0.74975265 | TAM I late versus naïve |
| *Shisa5* | 3.8366539681959656e-5 | 0.74965629 | TAM I late versus naïve |
| *Evi2a* | 0.0017439197864215976 | 0.74894719 | TAM I late versus naïve |
| *Clic1* | 0.0033945739083157764 | 0.74860746 | TAM I late versus naïve |
| *Nars* | 0.001608875255109423 | 0.74638062 | TAM I late versus naïve |
| *Zfand5* | 0.004676488496648074 | 0.74400864 | TAM I late versus naïve |
| *Vdac2* | 1.3356515063172987e-4 | 0.74394522 | TAM I late versus naïve |
| *Ndufb7* | 1.2778604823597482e-4 | 0.74139016 | TAM I late versus naïve |
| *Ccnd1* | 3.808566867884203e-5 | 0.74122082 | TAM I late versus naïve |
| *Adamts1* | 0.002125969074360279 | 0.73937562 | TAM I late versus naïve |
| *Ndufa2* | 2.3315702680875342e-5 | 0.73773251 | TAM I late versus naïve |
| *Arpc2* | 0.004158786563562609 | 0.73689241 | TAM I late versus naïve |
| *Ntpcr* | 7.4339057342170945e-6 | 0.73578549 | TAM I late versus naïve |
| *Rnaset2a* | 8.078616670360972e-5 | 0.72998896 | TAM I late versus naïve |
| *Prkar1a* | 2.4818025124139847e-6 | 0.72982297 | TAM I late versus naïve |
| *Tmem14c* | 8.653307914870703e-5 | 0.7295674 | TAM I late versus naïve |
| *Hpf1* | 1.871597329199724e-5 | 0.72848907 | TAM I late versus naïve |
| *Selenop* | 0.0031323102979126578 | 0.72818174 | TAM I late versus naïve |
| *Eef1a1* | 5.272720428004401e-4 | 0.72723631 | TAM I late versus naïve |
| *Dbi* | 8.28377857018564e-5 | 0.72668055 | TAM I late versus naïve |
| *Gm15427* | 0.004462788603075756 | 0.72497577 | TAM I late versus naïve |
| *Fcgr2b* | 0.007670158197482632 | 0.72489896 | TAM I late versus naïve |
| *Psap* | 7.218936848853105e-4 | 0.7241138 | TAM I late versus naïve |
| *Pld3* | 2.3714795463537894e-4 | 0.72017093 | TAM I late versus naïve |
| *Mt1* | 0.006463571785132153 | 0.71962059 | TAM I late versus naïve |
| *Coro1a* | 5.179899139582956e-5 | 0.71913672 | TAM I late versus naïve |
| *Gm6863* | 7.433089955276289e-4 | 0.71436955 | TAM I late versus naïve |
| *Ppp1r15a* | 0.006250383529134676 | 0.71163738 | TAM I late versus naïve |
| *Gm11966* | 7.792369055511784e-5 | 0.70963542 | TAM I late versus naïve |
| *Ddost* | 6.7565276902424275e-6 | 0.70941581 | TAM I late versus naïve |
| *Gm14586* | 4.106732719558719e-4 | 0.70939121 | TAM I late versus naïve |
| *Rpl37rt* | 0.007649737638223174 | 0.70764719 | TAM I late versus naïve |
| *Arpc3* | 4.957158016467877e-4 | 0.70508067 | TAM I late versus naïve |
| *Slfn5* | 2.0381696160949037e-8 | 0.70108424 | TAM I late versus naïve |
| *Sub1* | 1.3103202718785085e-4 | 0.70070052 | TAM I late versus naïve |
| *Ssrp1* | 9.837132675708418e-6 | 0.70067426 | TAM I late versus naïve |
| *Sdf2l1* | 4.887921441712706e-5 | 0.7006628 | TAM I late versus naïve |
| *Csrnp1* | 3.960698809624431e-9 | 0.70041134 | TAM I late versus naïve |
| *Nap1l1* | 1.3845867915387844e-6 | 0.69958343 | TAM I late versus naïve |
| *Mxd1* | 3.3494361420122085e-10 | 0.69907745 | TAM I late versus naïve |
| *Cd300lf* | 3.008960918689086e-10 | 0.69817991 | TAM I late versus naïve |
| *Snx3* | 1.225503138479979e-4 | 0.6974798 | TAM I late versus naïve |
| *Gm14681* | 1.1418771976609499e-4 | 0.69589392 | TAM I late versus naïve |
| *Scaf11* | 0.004833412342004465 | 0.69491405 | TAM I late versus naïve |
| *Pomp* | 4.632394998827762e-4 | 0.69384432 | TAM I late versus naïve |
| *Rpl36al* | 0.0010744209844375367 | 0.69309014 | TAM I late versus naïve |
| *Ctsa* | 0.0032666094633544817 | 0.69251788 | TAM I late versus naïve |
| *D8Ertd738e* | 0.004183064185847227 | 0.691835 | TAM I late versus naïve |
| *Hspe1* | 2.2988141408523727e-4 | 0.69049598 | TAM I late versus naïve |
| *Gm6265* | 9.332645950735096e-5 | 0.68955747 | TAM I late versus naïve |
| *Gm14513* | 1.4060399330318497e-7 | 0.68794251 | TAM I late versus naïve |
| *Cndp2* | 1.9837428521832785e-5 | 0.68750615 | TAM I late versus naïve |
| *Selenof* | 9.792231841679598e-5 | 0.68665019 | TAM I late versus naïve |
| *Slk* | 5.028293294862701e-6 | 0.68591026 | TAM I late versus naïve |
| *Isg15* | 3.450519895407015e-9 | 0.68534845 | TAM I late versus naïve |
| *Gm11361* | 1.759598569455934e-7 | 0.68506484 | TAM I late versus naïve |
| *Gm4204* | 3.542132586135555e-6 | 0.68412744 | TAM I late versus naïve |
| *Cox5b* | 8.647546654853556e-4 | 0.68370358 | TAM I late versus naïve |
| *Wfdc17* | 2.2924850539040035e-6 | 0.68349623 | TAM I late versus naïve |
| *Eprs* | 5.172065031820515e-4 | 0.68308487 | TAM I late versus naïve |
| *Tomm7* | 3.975314920741211e-5 | 0.68289077 | TAM I late versus naïve |
| *Rpl3-ps1* | 0.004191364360119342 | 0.67800594 | TAM I late versus naïve |
| *Tgif1* | 6.559525071882544e-7 | 0.67736541 | TAM I late versus naïve |
| *Gm4617* | 0.0012794083540511073 | 0.67697079 | TAM I late versus naïve |
| *Rps27l* | 9.82509389666086e-5 | 0.67622626 | TAM I late versus naïve |
| *Gm10076* | 7.202605801789336e-8 | 0.67607563 | TAM I late versus naïve |
| *H19* | 1.9364858020721896e-12 | 0.67475833 | TAM I late versus naïve |
| *Cd84* | 0.0014937090200925185 | 0.67193991 | TAM I late versus naïve |
| *Gm9892* | 3.90286384723702e-5 | 0.67147355 | TAM I late versus naïve |
| *Selenot* | 3.3956450256091164e-005 | 0.66583622 | TAM I late versus naïve |
| *Atp5a1* | 0.0010717540227080048 | 0.66535948 | TAM I late versus naïve |
| *Ddx1* | 3.407026012174961e-6 | 0.66116705 | TAM I late versus naïve |
| *Tiparp* | 3.278705448741209e-6 | 0.66073495 | TAM I late versus naïve |
| *Mbp* | 3.886701387960877e-5 | 0.66065738 | TAM I late versus naïve |
| *Psmb6* | 4.0847630073704937e-4 | 0.65751808 | TAM I late versus naïve |
| *Srp72* | 2.4535237916338867e-5 | 0.65722198 | TAM I late versus naïve |
| *Gm6030* | 1.0632360534886537e-4 | 0.65632443 | TAM I late versus naïve |
| *Sp100* | 2.0391757570978696e-8 | 0.65520452 | TAM I late versus naïve |
| *Kif5b* | 0.0022351415859717073 | 0.65504939 | TAM I late versus naïve |
| *Vapa* | 9.435161538059842e-4 | 0.65454185 | TAM I late versus naïve |
| *Rsad2* | 3.8100644229388444e-8 | 0.6545159 | TAM I late versus naïve |
| *Gm10169* | 2.831254055118029e-6 | 0.65367324 | TAM I late versus naïve |
| *Ppp1ca* | 6.328092537449891e-4 | 0.65176723 | TAM I late versus naïve |
| *Rpl18* | 1.0119257344352758e-5 | 0.65100971 | TAM I late versus naïve |
| *Rpl13* | 7.3896343033398525e-6 | 0.65081232 | TAM I late versus naïve |
| *Top2a* | 7.34304439288871e-6 | 0.64910739 | TAM I late versus naïve |
| *C5ar1* | 2.96813383471441e-4 | 0.64877827 | TAM I late versus naïve |
| *Rps26-ps1* | 1.896776300104642e-5 | 0.64692796 | TAM I late versus naïve |
| *Atp5f1* | 0.0015320649562839826 | 0.64616445 | TAM I late versus naïve |
| *Ndufc1* | 8.53251794369677e-7 | 0.64597381 | TAM I late versus naïve |
| *Zfp622* | 2.547396219250515e-5 | 0.64577643 | TAM I late versus naïve |
| *Tbcb* | 0.001145805382940644 | 0.64562235 | TAM I late versus naïve |
| *Nhp2* | 2.8319427577828134e-6 | 0.64558158 | TAM I late versus naïve |
| *Cd300c2* | 0.0018756420990362272 | 0.64246268 | TAM I late versus naïve |
| *Ifit3* | 4.562248005386919e-8 | 0.64228483 | TAM I late versus naïve |
| *Tmem256* | 3.1447997879505905e-4 | 0.64226771 | TAM I late versus naïve |
| *Lamp2* | 0.003597165215463038 | 0.64188905 | TAM I late versus naïve |
| *Unc93b1* | 0.002430688847085361 | 0.64158828 | TAM I late versus naïve |
| *Ndufb8* | 6.994618741418372e-4 | 0.64048922 | TAM I late versus naïve |
| *Ubl5* | 5.976279666970062e-6 | 0.63438749 | TAM I late versus naïve |
| *Srp14* | 6.244097218020566e-4 | 0.63393879 | TAM I late versus naïve |
| *Hmox1* | 3.544584416251256e-6 | 0.63385283 | TAM I late versus naïve |
| *Psmb1* | 5.393183974650335e-4 | 0.63309995 | TAM I late versus naïve |
| *Brk1* | 1.6565801305459092e-4 | 0.63306885 | TAM I late versus naïve |
| *Ndufa1* | 7.228245859101284e-5 | 0.63235344 | TAM I late versus naïve |
| *Tpm4* | 2.8905637996994667e-5 | 0.63085378 | TAM I late versus naïve |
| *Ndufa3* | 0.0012333994779556551 | 0.63070051 | TAM I late versus naïve |
| *Ezh2* | 1.0955413632487232e-5 | 0.62953648 | TAM I late versus naïve |
| *Naca* | 0.0017184890482906262 | 0.62873789 | TAM I late versus naïve |
| *Csde1* | 7.585262800837099e-5 | 0.62866393 | TAM I late versus naïve |
| *Dnajc8* | 0.0019499734078384375 | 0.627569 | TAM I late versus naïve |
| *Swi5* | 1.1432510701142874e-4 | 0.62716427 | TAM I late versus naïve |
| *Lrrfip1* | 3.477635169742282e-4 | 0.62362803 | TAM I late versus naïve |
| *Bsg* | 0.004318658356386122 | 0.62103517 | TAM I late versus naïve |
| *Ehd4* | 0.003332465179252571 | 0.6198698 | TAM I late versus naïve |
| *Sri* | 4.680162877022316e-6 | 0.619016 | TAM I late versus naïve |
| *Adgre1* | 2.686333256260051e-4 | 0.61858136 | TAM I late versus naïve |
| *Prpf38b* | 0.00429236346748326 | 0.61604014 | TAM I late versus naïve |
| *Sf3b2* | 6.217998888967058e-4 | 0.61580719 | TAM I late versus naïve |
| *Cap1* | 8.508179142544599e-4 | 0.61577557 | TAM I late versus naïve |
| *Cox17* | 0.0037953855233138155 | 0.61503106 | TAM I late versus naïve |
| *Cd53* | 0.0019326395480869089 | 0.61438545 | TAM I late versus naïve |
| *Epb41l3* | 1.2556123788009288e-6 | 0.61437234 | TAM I late versus naïve |
| *Arl5c* | 1.7416107178624924e-5 | 0.61413326 | TAM I late versus naïve |
| *Irf7* | 3.446150062219624e-9 | 0.61353193 | TAM I late versus naïve |
| *Naa50* | 1.7773759952253206e-5 | 0.61111918 | TAM I late versus naïve |
| *Mocs2* | 1.7726744747934091e-6 | 0.60959043 | TAM I late versus naïve |
| *Pmp22* | 0.0065780303974981085 | 0.60956171 | TAM I late versus naïve |
| *Syncrip* | 0.0015677706867613067 | 0.60869461 | TAM I late versus naïve |
| *Snx5* | 0.00543079145030761 | 0.60800365 | TAM I late versus naïve |
| *Cox7a2l* | 3.5977807103308224e-4 | 0.60548132 | TAM I late versus naïve |
| *Itgb2* | 9.797026103216593e-5 | 0.60524311 | TAM I late versus naïve |
| *Ndufa7* | 1.5328668936454321e-4 | 0.60519824 | TAM I late versus naïve |
| *Nedd8* | 2.722182450716642e-4 | 0.60496352 | TAM I late versus naïve |
| *Capzb* | 0.0016292630459100353 | 0.60493513 | TAM I late versus naïve |
| *Myl12b* | 0.0010402409777666463 | 0.60486485 | TAM I late versus naïve |
| *Hsbp1* | 5.400294662309917e-4 | 0.60457003 | TAM I late versus naïve |
| *Bzw1* | 8.168143241304769e-4 | 0.60351847 | TAM I late versus naïve |
| *Ranbp2* | 1.35271750380342e-4 | 0.60169362 | TAM I late versus naïve |
| *Csnk1a1* | 0.0036539413784294258 | 0.60087939 | TAM I late versus naïve |
| *Ppp1r11* | 1.3635536057962795e-6 | 0.60055711 | TAM I late versus naïve |
| *Abcf1* | 0.0013859101900693936 | 0.60033235 | TAM I late versus naïve |
| *Ssr3* | 3.2067532200454193e-4 | 0.59710657 | TAM I late versus naïve |
| *Arhgdia* | 0.008470908771717882 | 0.59625061 | TAM I late versus naïve |
| *Ndufa13* | 0.0015178266050441998 | 0.59605218 | TAM I late versus naïve |
| *Pim1* | 8.125152941280323e-6 | 0.59595757 | TAM I late versus naïve |
| *Gpr84* | 5.562060555824602e-7 | 0.59491978 | TAM I late versus naïve |
| *Acaa1a* | 3.856745163281057e-7 | 0.59469688 | TAM I late versus naïve |
| *Gm15500* | 0.0015470387752736059 | 0.59443101 | TAM I late versus naïve |
| *Rpl18-ps1* | 0.0025224794714421173 | 0.5941054 | TAM I late versus naïve |
| *Utp3* | 2.7326962939679903e-4 | 0.59376771 | TAM I late versus naïve |
| *Slc3a2* | 0.0022510270813136 | 0.59350013 | TAM I late versus naïve |
| *Tapbp* | 4.5176142416168615e-4 | 0.59122974 | TAM I late versus naïve |
| *Cfdp1* | 3.1432372866622835e-4 | 0.59065557 | TAM I late versus naïve |
| *Ap2s1* | 6.323028076357456e-5 | 0.59012285 | TAM I late versus naïve |
| *Gtpbp4* | 2.2143406651216197e-4 | 0.5881217 | TAM I late versus naïve |
| *Higd2a* | 1.1448811389128596e-5 | 0.58786337 | TAM I late versus naïve |
| *Wdr1* | 9.553015882121735e-7 | 0.58785087 | TAM I late versus naïve |
| *Psmb3* | 7.281266697756635e-4 | 0.58768101 | TAM I late versus naïve |
| *Bmp2k* | 0.0059667762565471785 | 0.58708586 | TAM I late versus naïve |
| *Atp6v1a* | 0.0017547733543292244 | 0.58658832 | TAM I late versus naïve |
| *Arl8b* | 1.1250749398432795e-5 | 0.58657359 | TAM I late versus naïve |
| *Prdx4* | 4.9488390217862574e-6 | 0.5865611 | TAM I late versus naïve |
| *Stat3* | 2.4795885724997404e-5 | 0.58437124 | TAM I late versus naïve |
| *Etf1* | 3.246502642676885e-4 | 0.58430229 | TAM I late versus naïve |
| *Arhgdib* | 0.0026806398987476083 | 0.58171767 | TAM I late versus naïve |
| *Phf11d* | 0.0017622276480022479 | 0.58065317 | TAM I late versus naïve |
| *Rpn1* | 4.791959088877959e-4 | 0.57998158 | TAM I late versus naïve |
| *Trim25* | 2.99091975069458e-10 | 0.57952024 | TAM I late versus naïve |
| *M6pr* | 0.008391133094939534 | 0.57895628 | TAM I late versus naïve |
| *Psma4* | 8.868846465665619e-4 | 0.57762649 | TAM I late versus naïve |
| *Dnajc13* | 6.720956571684641e-5 | 0.57754026 | TAM I late versus naïve |
| *Eif3f* | 0.0014501405707527829 | 0.57752367 | TAM I late versus naïve |
| *Tbca* | 1.9180848249236e-4 | 0.57740001 | TAM I late versus naïve |
| *Gm5835* | 0.0023837145753463447 | 0.57497067 | TAM I late versus naïve |
| *Pcbp2* | 0.003958356102942269 | 0.57380679 | TAM I late versus naïve |
| *Arpc4* | 0.0018140999860841898 | 0.57299733 | TAM I late versus naïve |
| *Eif3h* | 4.192865352449069e-5 | 0.57274333 | TAM I late versus naïve |
| *Banf1* | 4.9907439040715745e-5 | 0.57250437 | TAM I late versus naïve |
| *Sdf4* | 0.003542539753446462 | 0.57079574 | TAM I late versus naïve |
| *Icam1* | 2.1975615065893478e-4 | 0.5705119 | TAM I late versus naïve |
| *Ldhb* | 0.004501625433234828 | 0.56817126 | TAM I late versus naïve |
| *Ubxn1* | 3.968082500715544e-5 | 0.56810244 | TAM I late versus naïve |
| *Rab5c* | 4.917816156750147e-6 | 0.56790482 | TAM I late versus naïve |
| *1810037I17Rik* | 1.4054697072717304e-5 | 0.56480494 | TAM I late versus naïve |
| *Kdm6b* | 2.193658212344271e-6 | 0.56440021 | TAM I late versus naïve |
| *Lamtor1* | 7.835514769328329e-4 | 0.56411727 | TAM I late versus naïve |
| *Capns1* | 0.0024176745062549046 | 0.56390763 | TAM I late versus naïve |
| *Kras* | 4.529588085405041e-4 | 0.56361388 | TAM I late versus naïve |
| *Ifitm2* | 5.084802219957492e-7 | 0.56345855 | TAM I late versus naïve |
| *Rbx1* | 5.073796651153224e-5 | 0.56327366 | TAM I late versus naïve |
| *Snrpf* | 1.7719730302410937e-6 | 0.56197139 | TAM I late versus naïve |
| *Supt16* | 0.0018402294481587552 | 0.56139546 | TAM I late versus naïve |
| *Dab2* | 5.3942227411420254e-5 | 0.56132583 | TAM I late versus naïve |
| *Mrfap1* | 6.664330558223122e-4 | 0.56132531 | TAM I late versus naïve |
| *Gla* | 6.096232118526838e-10 | 0.56109044 | TAM I late versus naïve |
| *Cct6a* | 0.008252313204282933 | 0.55998064 | TAM I late versus naïve |
| *Tuba1a* | 0.004309555897217571 | 0.55963179 | TAM I late versus naïve |
| *Cln5* | 1.632975474137244e-6 | 0.55737184 | TAM I late versus naïve |
| *Atp5c1* | 0.00819104841002419 | 0.55561858 | TAM I late versus naïve |
| *Eea1* | 0.0011205403689381157 | 0.5551529 | TAM I late versus naïve |
| *Psme2b* | 9.449595316480971e-4 | 0.55471827 | TAM I late versus naïve |
| *Sp110* | 1.0486419735363223e-6 | 0.55427196 | TAM I late versus naïve |
| *Adipor1* | 8.648988267690798e-4 | 0.55146339 | TAM I late versus naïve |
| *Ndufb9* | 0.0010387953491230568 | 0.5483801 | TAM I late versus naïve |
| *Atp5g3* | 5.077128945933564e-5 | 0.54727268 | TAM I late versus naïve |
| *Psmb2* | 8.415556141689713e-5 | 0.54707806 | TAM I late versus naïve |
| *Rab7* | 0.0022108578122152084 | 0.5466266 | TAM I late versus naïve |
| *Set* | 0.001034232105942807 | 0.54619278 | TAM I late versus naïve |
| *Degs1* | 1.5515160017438258e-5 | 0.54464901 | TAM I late versus naïve |
| *Hnrnpul2* | 2.4226714928778472e-4 | 0.54417408 | TAM I late versus naïve |
| *Gm8730* | 1.6213816599052093e-4 | 0.54222019 | TAM I late versus naïve |
| *Eif2ak2* | 4.677457996405918e-5 | 0.5417756 | TAM I late versus naïve |
| *Ndufs6* | 7.712762132338384e-6 | 0.54000535 | TAM I late versus naïve |
| *Hcar2* | 3.4374251669540505e-9 | 0.53999286 | TAM I late versus naïve |
| *Wdr26* | 0.004632198587835891 | 0.53915239 | TAM I late versus naïve |
| *Cope* | 2.9304829260892104e-5 | 0.5391369 | TAM I late versus naïve |
| *Calm3* | 1.138756781870626e-5 | 0.53889793 | TAM I late versus naïve |
| *Ndufv3* | 4.6454517545072265e-4 | 0.53888134 | TAM I late versus naïve |
| *Saraf* | 0.002997794958412039 | 0.53770859 | TAM I late versus naïve |
| *Skil* | 0.006220270409707517 | 0.53762388 | TAM I late versus naïve |
| *Tmed3* | 2.7785185964323638e-5 | 0.53655734 | TAM I late versus naïve |
| *Rab14* | 0.009178398290755698 | 0.53598718 | TAM I late versus naïve |
| *Ndufc2* | 0.0038813810488041185 | 0.53584263 | TAM I late versus naïve |
| *Caprin1* | 7.536798808487447e-4 | 0.53557582 | TAM I late versus naïve |
| *Zcchc17* | 4.453115102652803e-6 | 0.53522345 | TAM I late versus naïve |
| *2410006H16Rik* | 0.009674137761994878 | 0.53515196 | TAM I late versus naïve |
| *Snrpb* | 5.298294555013514e-4 | 0.53453325 | TAM I late versus naïve |
| *Gm5905* | 0.007745819414224771 | 0.53439733 | TAM I late versus naïve |
| *Tceb1* | 0.0013549657788348357 | 0.53390205 | TAM I late versus naïve |
| *Gls* | 0.0016292931578626112 | 0.53341334 | TAM I late versus naïve |
| *Gm2000* | 5.898790461697809e-6 | 0.53321849 | TAM I late versus naïve |
| *Snrpd3* | 0.004364647893512674 | 0.53211279 | TAM I late versus naïve |
| *Milr1* | 2.576504688066842e-7 | 0.53211107 | TAM I late versus naïve |
| *Eef1g* | 0.0029606534325060463 | 0.5315071 | TAM I late versus naïve |
| *Gm10250* | 0.00630197961206456 | 0.53121271 | TAM I late versus naïve |
| *Cpd* | 0.0013658591096548471 | 0.53118501 | TAM I late versus naïve |
| *Ctnnb1* | 1.9988752561163152e-4 | 0.53078967 | TAM I late versus naïve |
| *Baz1b* | 0.008441371166683483 | 0.5287179 | TAM I late versus naïve |
| *Aprt* | 3.517064614200616e-7 | 0.52866105 | TAM I late versus naïve |
| *Ola1* | 9.648597307798715e-6 | 0.52864803 | TAM I late versus naïve |
| *Rpl27a* | 7.762199666333714e-5 | 0.52842156 | TAM I late versus naïve |
| *Ppt1* | 0.005657770349102668 | 0.52753823 | TAM I late versus naïve |
| *Ndufa11* | 8.850139114570098e-6 | 0.52731414 | TAM I late versus naïve |
| *2010107E04Rik* | 0.0011078898042900034 | 0.52679224 | TAM I late versus naïve |
| *Dazap2* | 7.670838634122592e-4 | 0.5265023 | TAM I late versus naïve |
| *Atp5k* | 0.002954105623370024 | 0.52642909 | TAM I late versus naïve |
| *Gnl3* | 7.12236678159488e-5 | 0.52621921 | TAM I late versus naïve |
| *Cdc37* | 9.816061426359405e-4 | 0.52597614 | TAM I late versus naïve |
| *Fkbp3* | 0.005009394825318363 | 0.52551929 | TAM I late versus naïve |
| *Eif4h* | 0.00029154223544573165 | 0.52535076 | TAM I late versus naïve |
| *Atp6ap1* | 4.0269444500293185e-4 | 0.52532167 | TAM I late versus naïve |
| *Tkt* | 3.804336684586015e-5 | 0.52529258 | TAM I late versus naïve |
| *Mob1a* | 3.912622283382941e-5 | 0.52401687 | TAM I late versus naïve |
| *Vamp3* | 1.482331029272737e-4 | 0.52248812 | TAM I late versus naïve |
| *Aldh2* | 6.245555671905602e-6 | 0.52244401 | TAM I late versus naïve |
| *Mrpl52* | 0.005363942588107374 | 0.52206852 | TAM I late versus naïve |
| *Esd* | 3.0013636173402527e-5 | 0.52205602 | TAM I late versus naïve |
| *Bach1* | 0.006673227248117948 | 0.5210157 | TAM I late versus naïve |
| *Arpc1a* | 0.0012181514795379829 | 0.52088789 | TAM I late versus naïve |
| *Lman2* | 0.0015411850571229126 | 0.51829331 | TAM I late versus naïve |
| *9530068E07Rik* | 6.817466790466207e-5 | 0.51812755 | TAM I late versus naïve |
| *Hnrnpl* | 0.0052826939831214885 | 0.5172425 | TAM I late versus naïve |
| *Lcp2* | 6.176591650478298e-4 | 0.51701603 | TAM I late versus naïve |
| *Etfb* | 2.5759258866781304e-7 | 0.51678116 | TAM I late versus naïve |
| *Glud1* | 4.75150774781695e-4 | 0.5159252 | TAM I late versus naïve |
| *Tuba1c* | 4.380945077931419e-8 | 0.51588362 | TAM I late versus naïve |
| *Rps24-ps2* | 1.4345090376779404e-6 | 0.51568623 | TAM I late versus naïve |
| *Uqcrh* | 0.006159148371028323 | 0.5148999 | TAM I late versus naïve |
| *Ssr2* | 0.004315147295046679 | 0.51480119 | TAM I late versus naïve |
| *Asah1* | 0.0025074932725989768 | 0.51437904 | TAM I late versus naïve |
| *Srsf3* | 0.007451164835847741 | 0.51412782 | TAM I late versus naïve |
| *Polr2f* | 3.4445890826229114e-5 | 0.5134548 | TAM I late versus naïve |
| *Mrpl54* | 6.493188916886405e-7 | 0.51328904 | TAM I late versus naïve |
| *Ifit2* | 4.0645452454973803e-7 | 0.5121375 | TAM I late versus naïve |
| *Parp1* | 4.352875529498202e-4 | 0.51174698 | TAM I late versus naïve |
| *Zfp91* | 0.0037294960634577956 | 0.50977778 | TAM I late versus naïve |
| *Cpne3* | 0.0016343188710182777 | 0.50907943 | TAM I late versus naïve |
| *Mir703* | 4.685934358273379e-5 | 0.50888434 | TAM I late versus naïve |
| *Plau* | 3.609252128619836e-5 | 0.50885526 | TAM I late versus naïve |
| *Ncor1* | 0.0031344071861370538 | 0.50871366 | TAM I late versus naïve |
| *Gm10443* | 4.311204168417384e-5 | 0.50865787 | TAM I late versus naïve |
| *Rtcb* | 6.342158018433676e-4 | 0.50776033 | TAM I late versus naïve |
| *Gm1673* | 4.96686569339168e-8 | 0.50774784 | TAM I late versus naïve |
| *Atp6v0d1* | 6.073463452343037e-4 | 0.50753385 | TAM I late versus naïve |
| *Fip1l1* | 2.2365753548469336e-4 | 0.50689188 | TAM I late versus naïve |
| *Parp9* | 1.252984237568284e-5 | 0.506682 | TAM I late versus naïve |
| *Ndufb11* | 5.5337461215226454e-5 | 0.50645552 | TAM I late versus naïve |
| *Emp3* | 0.0010550211329038125 | 0.50621656 | TAM I late versus naïve |
| *Ifih1* | 0.007580807525642528 | 0.50555798 | TAM I late versus naïve |
| *BC031181* | 4.8055613541071965e-5 | 0.50539222 | TAM I late versus naïve |
| *Gm8692* | 1.7188239426742027e-4 | 0.50493927 | TAM I late versus naïve |
| *Vdac1* | 3.697848169575082e-4 | 0.50466044 | TAM I late versus naïve |
| *Nr4a3* | 3.427270971208307e-9 | 0.50404583 | TAM I late versus naïve |
| *Snx2* | 0.007732554096755926 | 0.50381983 | TAM I late versus naïve |
| *Pon3* | 0.0010557975310331502 | 0.50339975 | TAM I late versus naïve |
| *Eif4g3* | 0.0032956953951141874 | 0.5025859 | TAM I late versus naïve |
| *Hif1a* | 0.001094772206224878 | 0.50076105 | TAM I late versus naïve |
| *Lyz2* | 1.2576239485337237e-30 | 5.60047894 | TAM II early versus naïve |
| *Cd74* | 1.9428367450369708e-36 | 5.25308623 | TAM II early versus naïve |
| *H2-Ab1* | 2.1661683129611873e-33 | 4.85783875 | TAM II early versus naïve |
| *H2-Eb1* | 3.574450728974835e-34 | 4.38040046 | TAM II early versus naïve |
| *H2-Aa* | 4.5843325376689195e-33 | 4.2914053 | TAM II early versus naïve |
| *Apoe* | 9.660821687151923e-22 | 4.09201384 | TAM II early versus naïve |
| *Fth1* | 9.96490601164501e-32 | 3.92860584 | TAM II early versus naïve |
| *Clec7a* | 1.1744174069906354e-33 | 3.38128889 | TAM II early versus naïve |
| *Il1b* | 1.4675576875921256e-25 | 3.34468464 | TAM II early versus naïve |
| *H2-K1* | 4.579196619977748e-33 | 3.07411232 | TAM II early versus naïve |
| *H2-D1* | 1.098015980491415e-31 | 2.90236489 | TAM II early versus naïve |
| *Calm1* | 8.270544211533096e-32 | 2.82270154 | TAM II early versus naïve |
| *Ly6a* | 4.6633831347805354e-32 | 2.64070551 | TAM II early versus naïve |
| *B2m* | 1.222861834841741e-31 | 2.53701561 | TAM II early versus naïve |
| *Gm6977* | 2.7869861292253156e-27 | 2.46799039 | TAM II early versus naïve |
| *Srgn* | 6.256772164598801e-28 | 2.39544351 | TAM II early versus naïve |
| *Vim* | 9.485542352075128e-33 | 2.38324259 | TAM II early versus naïve |
| *Cd14* | 1.4942855661610695e-21 | 2.36998733 | TAM II early versus naïve |
| *Ifitm3* | 1.1397615012325963e-31 | 2.35272386 | TAM II early versus naïve |
| *Ifi27l2a* | 1.3999510306204119e-27 | 2.28696734 | TAM II early versus naïve |
| *Tmsb10* | 1.0370391885319619e-30 | 2.21902399 | TAM II early versus naïve |
| *Cd52* | 3.881007457378126e-28 | 2.19423841 | TAM II early versus naïve |
| *Prdx5* | 8.774089131137107e-27 | 2.18003238 | TAM II early versus naïve |
| *Tgfbi* | 9.69020308687646e-28 | 2.16008344 | TAM II early versus naïve |
| *Cebpb* | 4.72343511490829e-25 | 2.09202349 | TAM II early versus naïve |
| *Rpl32* | 2.8047551560154093e-24 | 2.08621865 | TAM II early versus naïve |
| *Ctsc* | 1.401465499018862e-22 | 2.05219132 | TAM II early versus naïve |
| *Npc2* | 1.4576565121163074e-24 | 2.02256657 | TAM II early versus naïve |
| *Cybb* | 6.79554519115371e-30 | 2.01379645 | TAM II early versus naïve |
| *Txn1* | 1.0860426159745726e-29 | 1.98797279 | TAM II early versus naïve |
| *Rps26* | 6.966023431023249e-28 | 1.98239368 | TAM II early versus naïve |
| *Rps20* | 4.2623828074359075e-27 | 1.98084049 | TAM II early versus naïve |
| *AW112010* | 8.464755518814685e-26 | 1.96383858 | TAM II early versus naïve |
| *Gm9794* | 3.5780276297580065e-26 | 1.94666463 | TAM II early versus naïve |
| *Lgals3* | 7.528865973627944e-29 | 1.92983033 | TAM II early versus naïve |
| *Rps3* | 6.733590826142221e-25 | 1.9079817 | TAM II early versus naïve |
| *Bst2* | 1.5048237041545474e-26 | 1.90495738 | TAM II early versus naïve |
| *Lilrb4a* | 1.4960903132108977e-27 | 1.88507621 | TAM II early versus naïve |
| *Thbs1* | 1.969680490961071e-22 | 1.87933579 | TAM II early versus naïve |
| *Cstb* | 4.5239312414700477e-26 | 1.84171004 | TAM II early versus naïve |
| *Rps5* | 8.463173384614158e-23 | 1.81825263 | TAM II early versus naïve |
| *Psmb8* | 7.8758394505053285e-25 | 1.79809938 | TAM II early versus naïve |
| *Rplp1* | 1.262780173241767e-21 | 1.77664186 | TAM II early versus naïve |
| *Fxyd5* | 1.2927019097725555e-028 | 1.75519269 | TAM II early versus naïve |
| *Ms4a6c* | 1.0004338476997945e-24 | 1.74943398 | TAM II early versus naïve |
| *Tmsb4x* | 2.3772202994665724e-23 | 1.73150612 | TAM II early versus naïve |
| *AA467197* | 5.845364637038082e-24 | 1.7121728 | TAM II early versus naïve |
| *Msr1* | 1.1832867055624636e-22 | 1.71160946 | TAM II early versus naïve |
| *Rpl22* | 2.167386832548197e-22 | 1.68848383 | TAM II early versus naïve |
| *Cdkn1a* | 2.9498937658036737e-24 | 1.67778754 | TAM II early versus naïve |
| *Ptgs2* | 8.876241527670776e-23 | 1.66869982 | TAM II early versus naïve |
| *Wdr89* | 6.648198112034924e-22 | 1.64777412 | TAM II early versus naïve |
| *Rpl39* | 3.406778066349327e-23 | 1.64334625 | TAM II early versus naïve |
| *Rplp0* | 1.2002268771677553e-21 | 1.63978036 | TAM II early versus naïve |
| *Rps24* | 6.097132204129039e-22 | 1.61310148 | TAM II early versus naïve |
| *Lgals1* | 1.008111865353486e-23 | 1.60438216 | TAM II early versus naïve |
| *Rpl41* | 5.8005808672143344e-21 | 1.59664984 | TAM II early versus naïve |
| *Prdx1* | 4.767829663499621e-19 | 1.56371537 | TAM II early versus naïve |
| *Ly6e* | 9.031192838388935e-20 | 1.56105317 | TAM II early versus naïve |
| *Rps19* | 1.350445818219935e-23 | 1.56033383 | TAM II early versus naïve |
| *Clec4n* | 4.8253599842073215e-22 | 1.55735588 | TAM II early versus naïve |
| *Rpl4* | 1.6440898109103236e-17 | 1.5552614 | TAM II early versus naïve |
| *Psap* | 7.821322955061603e-17 | 1.54250795 | TAM II early versus naïve |
| *Ly6c2* | 6.030686896234067e-18 | 1.53917262 | TAM II early versus naïve |
| *Rpl8* | 3.0587837573457234e-20 | 1.53640176 | TAM II early versus naïve |
| *Rps14* | 8.301482747527001e-19 | 1.53366616 | TAM II early versus naïve |
| *Ncl* | 4.14531225666543e-19 | 1.52296559 | TAM II early versus naïve |
| *Rps9* | 8.02365551333813e-21 | 1.50715902 | TAM II early versus naïve |
| *Bcl2a1b* | 8.850085661864775e-21 | 1.50465413 | TAM II early versus naïve |
| *Gm9843* | 1.1594972017918515e-20 | 1.50394021 | TAM II early versus naïve |
| *Ccrl2* | 6.534300288286408e-17 | 1.50278039 | TAM II early versus naïve |
| *Shfm1* | 1.8176259318255248e-19 | 1.48877549 | TAM II early versus naïve |
| *Nampt* | 6.598317876017751e-24 | 1.48053948 | TAM II early versus naïve |
| *Gm5963* | 1.6157245297257922e-19 | 1.47445561 | TAM II early versus naïve |
| *Rps13-ps2* | 6.16164372626404e-20 | 1.46645553 | TAM II early versus naïve |
| *Cxcl9* | 1.9227367175455575e-15 | 1.46320725 | TAM II early versus naïve |
| *Arpc1b* | 3.30464334910969e-19 | 1.4565656 | TAM II early versus naïve |
| *Rpl14* | 3.2775227486024682e-21 | 1.44506241 | TAM II early versus naïve |
| *Rack1* | 2.4075782259471854e-18 | 1.43612519 | TAM II early versus naïve |
| *Dab2* | 5.515563770424874e-20 | 1.43405924 | TAM II early versus naïve |
| *Iqgap1* | 1.3375340085031378e-21 | 1.43318725 | TAM II early versus naïve |
| *Vegfa* | 2.345447927187972e-22 | 1.42528995 | TAM II early versus naïve |
| *Rps24-ps3* | 1.5563403666576e-19 | 1.42055944 | TAM II early versus naïve |
| *Rps2* | 2.826780451741917e-17 | 1.4188776 | TAM II early versus naïve |
| *Cyba* | 1.6636468316832417e-17 | 1.4171232 | TAM II early versus naïve |
| *Tnfaip2* | 8.074716428086317e-20 | 1.41441297 | TAM II early versus naïve |
| *Pfn1* | 1.6080771615754777e-17 | 1.41256667 | TAM II early versus naïve |
| *Rpl34* | 2.04054839285459e-21 | 1.40945215 | TAM II early versus naïve |
| *Rps15* | 1.0176195661203636e-18 | 1.40622562 | TAM II early versus naïve |
| *Ccl6* | 1.760491073740846e-15 | 1.39964308 | TAM II early versus naïve |
| *Ifrd1* | 3.8989362266247724e-20 | 1.39101288 | TAM II early versus naïve |
| *Rpl37rt* | 2.813810319788316e-20 | 1.38387717 | TAM II early versus naïve |
| *Cfp* | 2.0714832607732366e-21 | 1.38265092 | TAM II early versus naïve |
| *Gm14303* | 1.092626638296739e-16 | 1.37882439 | TAM II early versus naïve |
| *Gm14586* | 6.89430105278104e-21 | 1.37708765 | TAM II early versus naïve |
| *Mcl1* | 1.4924143249541915e-19 | 1.37376334 | TAM II early versus naïve |
| *Gbp2* | 3.8263794710918104e-20 | 1.37358936 | TAM II early versus naïve |
| *Ybx1* | 1.12179733972526e-15 | 1.37065266 | TAM II early versus naïve |
| *Cox4i1* | 4.569247238990474e-17 | 1.37020724 | TAM II early versus naïve |
| *S100a11* | 6.710048101266617e-24 | 1.36169111 | TAM II early versus naïve |
| *Gpx1* | 9.516695020967905e-16 | 1.35618035 | TAM II early versus naïve |
| *App* | 2.7366393616087043e-16 | 1.35568235 | TAM II early versus naïve |
| *Pim1* | 4.653311909871524e-23 | 1.35560203 | TAM II early versus naïve |
| *Rpl23* | 5.689108373291951e-19 | 1.35112688 | TAM II early versus naïve |
| *Aldoa* | 6.00593744314005e-21 | 1.34888743 | TAM II early versus naïve |
| *Rps21* | 3.619212764909799e-16 | 1.34289405 | TAM II early versus naïve |
| *Stat1* | 6.400344597052448e-24 | 1.34223044 | TAM II early versus naïve |
| *Mif* | 1.5612283304188905e-22 | 1.33664675 | TAM II early versus naïve |
| *Cox6b1* | 1.3508801693950538e-17 | 1.33192653 | TAM II early versus naïve |
| *Fosb* | 1.085699725993562e-14 | 1.33160181 | TAM II early versus naïve |
| *Sh3bgrl3* | 8.11704654653762e-18 | 1.3291336 | TAM II early versus naïve |
| *Msrb1* | 1.5740106434987558e-23 | 1.3259638 | TAM II early versus naïve |
| *Fgl2* | 1.8795525592802726e-20 | 1.32576115 | TAM II early versus naïve |
| *Hsp90ab1* | 1.4294577878590895e-13 | 1.31398615 | TAM II early versus naïve |
| *Nfe2l2* | 4.574766141002707e-16 | 1.3119918 | TAM II early versus naïve |
| *Anxa5* | 3.9627852244475896e-22 | 1.31024059 | TAM II early versus naïve |
| *Pabpc1* | 5.329731348726616e-15 | 1.30775417 | TAM II early versus naïve |
| *Gm9385* | 7.553433470487533e-18 | 1.30540361 | TAM II early versus naïve |
| *Rps15a* | 1.8378932417727734e-18 | 1.29969963 | TAM II early versus naïve |
| *Rpl26* | 4.325469012605133e-17 | 1.29316616 | TAM II early versus naïve |
| *Crip1* | 3.1769311941027124e-20 | 1.29249433 | TAM II early versus naïve |
| *Actb* | 4.942881178999885e-15 | 1.29182602 | TAM II early versus naïve |
| *Rpl14-ps1* | 1.850690885106569e-15 | 1.28851676 | TAM II early versus naïve |
| *Rps16-ps2* | 8.064770569956954e-17 | 1.28085116 | TAM II early versus naïve |
| *Slfn2* | 3.6616216375209354e-17 | 1.27509579 | TAM II early versus naïve |
| *Rps10-ps1* | 9.765717255058345e-16 | 1.27021055 | TAM II early versus naïve |
| *Gm42418* | 1.5445869315147925e-10 | 1.2690845 | TAM II early versus naïve |
| *Ly6i* | 3.9635153558788765e-18 | 1.26737784 | TAM II early versus naïve |
| *Xist* | 8.800137237296306e-22 | 1.26096643 | TAM II early versus naïve |
| *H2-DMb1* | 1.3764990859794657e-19 | 1.25838622 | TAM II early versus naïve |
| *Fos* | 7.561650184615552e-9 | 1.24597971 | TAM II early versus naïve |
| *Fcgr2b* | 1.794971500515885e-11 | 1.24090503 | TAM II early versus naïve |
| *Cxcl2* | 6.375271290581221e-11 | 1.23065564 | TAM II early versus naïve |
| *Gm10288* | 8.749163927261086e-17 | 1.22954648 | TAM II early versus naïve |
| *Eef1a1* | 1.2451908531504683e-14 | 1.22251563 | TAM II early versus naïve |
| *Cox5a* | 1.9801773510673073e-19 | 1.22109705 | TAM II early versus naïve |
| *Ifi204* | 4.071058488616334e-19 | 1.22049234 | TAM II early versus naïve |
| *Fcer1g* | 6.532440554045553e-12 | 1.21862963 | TAM II early versus naïve |
| *Anp32b* | 4.196457010782797e-16 | 1.21834454 | TAM II early versus naïve |
| *Rpl31-ps8* | 3.482608438259544e-18 | 1.21768572 | TAM II early versus naïve |
| *H2-Q7* | 1.1333629464922313e-21 | 1.21713643 | TAM II early versus naïve |
| *Psme1* | 2.762427674669286e-16 | 1.21699089 | TAM II early versus naïve |
| *Serbp1* | 1.1729780418117195e-12 | 1.21461052 | TAM II early versus naïve |
| *Ifi30* | 2.9070375934380183e-18 | 1.21418422 | TAM II early versus naïve |
| *Gm11478* | 6.539861558545462e-13 | 1.21088938 | TAM II early versus naïve |
| *Pkm* | 4.136419987169858e-16 | 1.20963168 | TAM II early versus naïve |
| *Gm16580* | 5.92108916528181e-14 | 1.20594265 | TAM II early versus naïve |
| *Wfdc17* | 4.78731951092888e-16 | 1.19839013 | TAM II early versus naïve |
| *Atox1* | 2.2553376973574905e-16 | 1.19577983 | TAM II early versus naïve |
| *H2-T23* | 2.6694377618446473e-15 | 1.1935869 | TAM II early versus naïve |
| *Cxcl16* | 5.183626242108647e-18 | 1.19085732 | TAM II early versus naïve |
| *Lcp1* | 9.38442454585236e-13 | 1.18919788 | TAM II early versus naïve |
| *Ms4a7* | 8.457849355494837e-16 | 1.17843838 | TAM II early versus naïve |
| *Cox6a1* | 3.058548725141007e-15 | 1.16932429 | TAM II early versus naïve |
| *Tspo* | 1.1092805033863393e-17 | 1.16773442 | TAM II early versus naïve |
| *Rpl35a* | 2.2947983970466873e-13 | 1.16741059 | TAM II early versus naïve |
| *Rpsa-ps10* | 4.552222977461509e-15 | 1.16667827 | TAM II early versus naïve |
| *Psme2b* | 1.4096296670354914e-13 | 1.15807888 | TAM II early versus naïve |
| *Gm5905* | 1.827135142427785e-13 | 1.15265588 | TAM II early versus naïve |
| *Gm5805* | 1.8639983500910702e-14 | 1.1518006 | TAM II early versus naïve |
| *Mndal* | 1.569282811245737e-20 | 1.15149674 | TAM II early versus naïve |
| *Rps26-ps1* | 3.57720745040527e-17 | 1.14445102 | TAM II early versus naïve |
| *Actr3* | 1.6666635749730946e-14 | 1.13664093 | TAM II early versus naïve |
| *Psma7* | 3.089223423500558e-13 | 1.13561202 | TAM II early versus naïve |
| *Cox8a* | 2.450586172680409e-13 | 1.13330409 | TAM II early versus naïve |
| *Gm4332* | 3.771685006336245e-15 | 1.13325634 | TAM II early versus naïve |
| *Tpt1-ps3* | 1.4379626697215083e-14 | 1.12726666 | TAM II early versus naïve |
| *Fosl2* | 3.280629840887981e-22 | 1.12643057 | TAM II early versus naïve |
| *Il2rg* | 6.149709194903085e-22 | 1.12602606 | TAM II early versus naïve |
| *Pla2g7* | 9.049710379828402e-15 | 1.12469485 | TAM II early versus naïve |
| *Gm15427* | 1.3076389577632678e-13 | 1.12460428 | TAM II early versus naïve |
| *Tgm2* | 1.2179658865262801e-17 | 1.12390786 | TAM II early versus naïve |
| *Hnrnpab* | 1.7409670816135065e-14 | 1.12317457 | TAM II early versus naïve |
| *Il1rn* | 1.4759067960436327e-14 | 1.12210166 | TAM II early versus naïve |
| *Nfkbia* | 5.245021589186593e-7 | 1.12097882 | TAM II early versus naïve |
| *Gm6030* | 4.2314008862373886e-20 | 1.12012514 | TAM II early versus naïve |
| *Arg1* | 1.0042911065710163e-10 | 1.11551267 | TAM II early versus naïve |
| *Eef1b2* | 2.200162759516016e-13 | 1.10337718 | TAM II early versus naïve |
| *Rps3a1* | 2.8833162128114387e-13 | 1.09833516 | TAM II early versus naïve |
| *Rps8* | 9.4309360428101e-16 | 1.09616909 | TAM II early versus naïve |
| *Akr1a1* | 2.386570649259938e-13 | 1.09144325 | TAM II early versus naïve |
| *Hif1a* | 3.585145115003136e-14 | 1.08984343 | TAM II early versus naïve |
| *Cox7b* | 1.7034106973224494e-13 | 1.08953879 | TAM II early versus naïve |
| *Sh3bgrl* | 1.3441940054890968e-17 | 1.0887025 | TAM II early versus naïve |
| *Sdcbp* | 2.5883862176122227e-13 | 1.0857821 | TAM II early versus naïve |
| *Slc25a5* | 5.054678029037236e-10 | 1.08185672 | TAM II early versus naïve |
| *Rps18* | 1.4020827254392648e-13 | 1.07827066 | TAM II early versus naïve |
| *Ccl5* | 1.1390550688191826e-11 | 1.07661928 | TAM II early versus naïve |
| *Gbp7* | 2.59888394404907e-11 | 1.07655839 | TAM II early versus naïve |
| *Rps27l* | 1.3369431939479518e-16 | 1.07409454 | TAM II early versus naïve |
| *Ccr1* | 4.236615114561968e-17 | 1.06834912 | TAM II early versus naïve |
| *Rps25-ps1* | 1.8200137930539644e-15 | 1.06769795 | TAM II early versus naïve |
| *Atp5e* | 3.625728671258498e-14 | 1.06677913 | TAM II early versus naïve |
| *Cox6c* | 5.838494411983375e-14 | 1.06337611 | TAM II early versus naïve |
| *Rpl36a-ps2* | 9.571682870259057e-16 | 1.05464053 | TAM II early versus naïve |
| *Ldha* | 1.971222015772679e-17 | 1.05192799 | TAM II early versus naïve |
| *H2-DMa* | 1.735116992066793e-11 | 1.04747795 | TAM II early versus naïve |
| *Eif3a* | 5.294892115963762e-11 | 1.04140693 | TAM II early versus naïve |
| *Gm4149* | 7.050284238302863e-14 | 1.04092873 | TAM II early versus naïve |
| *2010107E04Rik* | 2.2624540342088107e-15 | 1.04026993 | TAM II early versus naïve |
| *Hspa5* | 1.5117405437672204e-7 | 1.0337019 | TAM II early versus naïve |
| *Gm5835* | 7.614349868672652e-13 | 1.03297497 | TAM II early versus naïve |
| *Arpc2* | 8.001032682218772e-11 | 1.03212522 | TAM II early versus naïve |
| *Isg15* | 2.2460538008656798e-17 | 1.02979387 | TAM II early versus naïve |
| *Txn-ps1* | 3.064031710470133e-17 | 1.02543376 | TAM II early versus naïve |
| *Eef2* | 2.597224662861521e-15 | 1.02456267 | TAM II early versus naïve |
| *Lrrfip1* | 5.769667396708678e-15 | 1.01613935 | TAM II early versus naïve |
| *Arpc5* | 5.130994315731853e-13 | 1.01520013 | TAM II early versus naïve |
| *Rpl36al* | 3.286348983519767e-16 | 1.01383505 | TAM II early versus naïve |
| *Gm10275* | 1.1070661709648005e-11 | 1.01244103 | TAM II early versus naïve |
| *Hint1* | 1.721705044871384e-13 | 1.00195735 | TAM II early versus naïve |
| *Rpl3-ps1* | 2.8053402065996926e-9 | 0.99947305 | TAM II early versus naïve |
| *Slc15a3* | 4.7899554704557594e-12 | 0.99686815 | TAM II early versus naïve |
| *Rplp2* | 3.137447441378157e-12 | 0.99389502 | TAM II early versus naïve |
| *Nr4a2* | 5.296611760974609e-18 | 0.99345741 | TAM II early versus naïve |
| *Cfl1* | 1.1166933258307573e-11 | 0.99029836 | TAM II early versus naïve |
| *Ms4a4c* | 1.712694502889384e-16 | 0.98851722 | TAM II early versus naïve |
| *Minos1* | 2.2571689697384144e-14 | 0.98472998 | TAM II early versus naïve |
| *Npm1* | 1.8628188928243e-12 | 0.98147561 | TAM II early versus naïve |
| *Psma2* | 4.548650015814697e-15 | 0.98098682 | TAM II early versus naïve |
| *Tiparp* | 1.0264810677253903e-14 | 0.98040861 | TAM II early versus naïve |
| *Myl12a* | 1.1361385335423327e-13 | 0.9798699 | TAM II early versus naïve |
| *Ctsz* | 5.775201450949244e-9 | 0.9777998 | TAM II early versus naïve |
| *Nr4a3* | 3.661059801262659e-15 | 0.97365794 | TAM II early versus naïve |
| *Capg* | 1.229359983020971e-15 | 0.97172197 | TAM II early versus naïve |
| *Samhd1* | 5.205411188400401e-11 | 0.97135479 | TAM II early versus naïve |
| *Osm* | 2.2640852012721887e-14 | 0.96205718 | TAM II early versus naïve |
| *C3* | 4.250197386519727e-16 | 0.96181488 | TAM II early versus naïve |
| *Tomm7* | 3.95855018907018e-15 | 0.95920509 | TAM II early versus naïve |
| *Gm4204* | 1.3576885896961014e-14 | 0.95726443 | TAM II early versus naïve |
| *Atpif1* | 2.0616121788795922e-11 | 0.95592171 | TAM II early versus naïve |
| *Plek* | 1.8985843012848016e-8 | 0.95314425 | TAM II early versus naïve |
| *Psme2* | 2.357627311664569e-13 | 0.95261 | TAM II early versus naïve |
| *Tpr* | 2.0757040318458072e-10 | 0.95206404 | TAM II early versus naïve |
| *Tagln2* | 1.6825940972583008e-14 | 0.95198317 | TAM II early versus naïve |
| *Mxd1* | 8.040638244173622e-17 | 0.9441192 | TAM II early versus naïve |
| *S100a10* | 5.929734010710998e-18 | 0.94129097 | TAM II early versus naïve |
| *Plbd1* | 8.809106240338535e-16 | 0.93995783 | TAM II early versus naïve |
| *Gpr65* | 3.1151989470374534e-14 | 0.93936385 | TAM II early versus naïve |
| *Cd63* | 2.1510770921698364e-7 | 0.93777759 | TAM II early versus naïve |
| *Rps10* | 2.6813742387161796e-12 | 0.93522425 | TAM II early versus naïve |
| *Tpm4* | 9.431038987522639e-14 | 0.93393938 | TAM II early versus naïve |
| *Gm4617* | 5.57806956703168e-11 | 0.93239808 | TAM II early versus naïve |
| *Atf3* | 9.373173188732904e-8 | 0.93020999 | TAM II early versus naïve |
| *Chchd2* | 7.28480913941945e-10 | 0.92497082 | TAM II early versus naïve |
| *Psmb9* | 3.018881420422588e-15 | 0.92404611 | TAM II early versus naïve |
| *Psmb1* | 9.822453998425482e-12 | 0.92261281 | TAM II early versus naïve |
| *H3f3b* | 1.0118264259280354e-7 | 0.91985004 | TAM II early versus naïve |
| *Hspa8* | 1.710329402563111e-8 | 0.91950539 | TAM II early versus naïve |
| *Bcl2a1d* | 2.1887937780260316e-17 | 0.91929902 | TAM II early versus naïve |
| *Gm6472* | 1.0561310158965604e-12 | 0.91756829 | TAM II early versus naïve |
| *Rpl37* | 8.712906734430931e-14 | 0.91562945 | TAM II early versus naïve |
| *Tyrobp* | 6.891881477741374e-10 | 0.91209508 | TAM II early versus naïve |
| *Rpl38-ps2* | 1.0891633418591168e-13 | 0.90840559 | TAM II early versus naïve |
| *Cotl1* | 1.6850410037937855e-8 | 0.90355131 | TAM II early versus naïve |
| *Gm15500* | 9.891005418098779e-11 | 0.90313749 | TAM II early versus naïve |
| *Atp5j2* | 4.329608690983456e-12 | 0.90118168 | TAM II early versus naïve |
| *Hnrnpm* | 1.2088364286254199e-10 | 0.89870198 | TAM II early versus naïve |
| *Bcl2a1a* | 8.428667096586021e-14 | 0.89747689 | TAM II early versus naïve |
| *Ms4a6b* | 1.0228836520420782e-10 | 0.89742859 | TAM II early versus naïve |
| *Plaur* | 1.0365078128361236e-17 | 0.89639797 | TAM II early versus naïve |
| *Rps11* | 2.9540265420913114e-9 | 0.89414403 | TAM II early versus naïve |
| *Gm14539* | 5.940281331758289e-16 | 0.89349065 | TAM II early versus naïve |
| *Lgals3bp* | 4.132970268563801e-12 | 0.8931739 | TAM II early versus naïve |
| *M6pr* | 1.1267359786994077e-10 | 0.89071696 | TAM II early versus naïve |
| *Gabarap* | 9.36848980056759e-11 | 0.89027006 | TAM II early versus naïve |
| *Ifi209* | 3.207557735819415e-16 | 0.88945324 | TAM II early versus naïve |
| *Gm14513* | 1.7630827275197313e-14 | 0.88408397 | TAM II early versus naïve |
| *Actr2* | 2.573564040592517e-9 | 0.88146198 | TAM II early versus naïve |
| *Tlr2* | 4.625004697599498e-13 | 0.88088324 | TAM II early versus naïve |
| *Btg1* | 6.753261738584596e-10 | 0.87885 | TAM II early versus naïve |
| *Gm8995* | 1.78899425699888e-11 | 0.87842848 | TAM II early versus naïve |
| *Nfil3* | 2.051583250812747e-17 | 0.87827313 | TAM II early versus naïve |
| *Plac8* | 9.958041723033368e-11 | 0.87716488 | TAM II early versus naïve |
| *Arhgdib* | 1.244001327294051e-9 | 0.87583612 | TAM II early versus naïve |
| *Fabp5* | 2.8423901887146053e-11 | 0.8749293 | TAM II early versus naïve |
| *Gm10250* | 4.1787015050624786e-11 | 0.87401956 | TAM II early versus naïve |
| *Klra2* | 1.723439107718158e-15 | 0.87194821 | TAM II early versus naïve |
| *Ctsb* | 5.382524055021582e-5 | 0.86973367 | TAM II early versus naïve |
| *Hnrnpu* | 1.3941314218332266e-8 | 0.86838437 | TAM II early versus naïve |
| *Ptprc* | 1.5932691168418343e-7 | 0.86837631 | TAM II early versus naïve |
| *Hspa4* | 3.6242586091088186e-9 | 0.8647026 | TAM II early versus naïve |
| *Acp5* | 5.31868279069425e-14 | 0.86434293 | TAM II early versus naïve |
| *Mrc1* | 5.159058521085004e-11 | 0.86111651 | TAM II early versus naïve |
| *Gm8730* | 1.9493488999633772e-11 | 0.86058899 | TAM II early versus naïve |
| *Gm10076* | 4.731480877351011e-15 | 0.85926194 | TAM II early versus naïve |
| *Pilra* | 6.724720426759018e-17 | 0.85891563 | TAM II early versus naïve |
| *Emp3* | 7.536923468500556e-15 | 0.85877111 | TAM II early versus naïve |
| *Slfn5* | 1.3869317827860763e-13 | 0.85673593 | TAM II early versus naïve |
| *Cd63-ps* | 3.4397710229590816e-7 | 0.85408241 | TAM II early versus naïve |
| *Prrc2c* | 2.9247479846528937e-8 | 0.84838286 | TAM II early versus naïve |
| *Sub1* | 1.0019667275418029e-11 | 0.8480531 | TAM II early versus naïve |
| *Aif1* | 3.3405071467407705e-6 | 0.84672073 | TAM II early versus naïve |
| *Iigp1* | 3.8186860151766727e-13 | 0.84068904 | TAM II early versus naïve |
| *Cox17* | 3.2979930686211566e-12 | 0.83777416 | TAM II early versus naïve |
| *Pde4b* | 4.759922529170564e-14 | 0.83754367 | TAM II early versus naïve |
| *Ifi203* | 4.815303384721456e-14 | 0.83692164 | TAM II early versus naïve |
| *Itgb1* | 5.19085895563213e-8 | 0.83613917 | TAM II early versus naïve |
| *Rpl9-ps6* | 2.2412460845041644e-13 | 0.8345525 | TAM II early versus naïve |
| *Clec12a* | 1.8438464077007507e-16 | 0.83418251 | TAM II early versus naïve |
| *Ets2* | 1.7720694788423037e-15 | 0.83207427 | TAM II early versus naïve |
| *Slc25a3* | 1.5602741902733746e-9 | 0.83100528 | TAM II early versus naïve |
| *Ms4a6d* | 4.105793814231424e-12 | 0.82852617 | TAM II early versus naïve |
| *Arpc3* | 2.8130803448925166e-10 | 0.82849493 | TAM II early versus naïve |
| *Prdx6* | 1.086703797904768e-14 | 0.82826737 | TAM II early versus naïve |
| *Chmp4b* | 5.69762628818864e-11 | 0.82813237 | TAM II early versus naïve |
| *Rpl18-ps1* | 2.328195321744297e-11 | 0.82653687 | TAM II early versus naïve |
| *Cox7a2* | 3.2919392375810704e-9 | 0.82564525 | TAM II early versus naïve |
| *Slamf7* | 8.624633771486169e-17 | 0.82457642 | TAM II early versus naïve |
| *Pdia3* | 2.404147656326969e-6 | 0.8238293 | TAM II early versus naïve |
| *Cyp4f18* | 1.589681633688677e-15 | 0.82176579 | TAM II early versus naïve |
| *Gm6863* | 6.808381842421213e-10 | 0.82099552 | TAM II early versus naïve |
| *Dek* | 2.714925492627854e-8 | 0.82078406 | TAM II early versus naïve |
| *Mmp14* | 4.018740944498746e-15 | 0.81745363 | TAM II early versus naïve |
| *Atp5j* | 5.038576065668053e-10 | 0.81744118 | TAM II early versus naïve |
| *BC005537* | 4.3076216581534165e-9 | 0.81435376 | TAM II early versus naïve |
| *Crem* | 8.79385602496854e-14 | 0.81325972 | TAM II early versus naïve |
| *Cxcl10* | 1.9348790147840323e-8 | 0.8129494 | TAM II early versus naïve |
| *Trim30a* | 2.2360573054800137e-8 | 0.81077036 | TAM II early versus naïve |
| *Sash1* | 4.362537262724173e-14 | 0.80977367 | TAM II early versus naïve |
| *Psmb6* | 3.606496346116916e-11 | 0.80844015 | TAM II early versus naïve |
| *Taldo1* | 8.738079081737389e-10 | 0.80691672 | TAM II early versus naïve |
| *Rps10-ps2* | 1.895230287060197e-11 | 0.80253437 | TAM II early versus naïve |
| *Myof* | 1.458932370098195e-13 | 0.80234335 | TAM II early versus naïve |
| *Zbp1* | 6.63427414546807e-15 | 0.80094052 | TAM II early versus naïve |
| *Litaf* | 1.0192119279268113e-11 | 0.79910591 | TAM II early versus naïve |
| *Cd300lf* | 1.821947943723965e-16 | 0.79768347 | TAM II early versus naïve |
| *Fcgr4* | 2.9860927597623974e-13 | 0.79553511 | TAM II early versus naïve |
| *Tuba1b* | 2.8649903316859446e-7 | 0.79268816 | TAM II early versus naïve |
| *Chd4* | 3.13596250883505e-9 | 0.79186049 | TAM II early versus naïve |
| *Il1a* | 1.6321321838277312e-9 | 0.7901034 | TAM II early versus naïve |
| *Rpl13* | 5.568906331280381e-11 | 0.78782434 | TAM II early versus naïve |
| *Rnf149* | 6.902933874703137e-12 | 0.78631275 | TAM II early versus naïve |
| *Tpm3-rs7* | 4.60719634571215e-6 | 0.78602153 | TAM II early versus naïve |
| *Txnrd1* | 7.159348832312543e-12 | 0.78419908 | TAM II early versus naïve |
| *Junb* | 2.4677070893942654e-5 | 0.78393426 | TAM II early versus naïve |
| *Rpl37a* | 8.269852701145412e-13 | 0.78380712 | TAM II early versus naïve |
| *Eprs* | 3.383806430042468e-8 | 0.78351162 | TAM II early versus naïve |
| *Atp1b3* | 3.78096970614707e-9 | 0.78314034 | TAM II early versus naïve |
| *Tapbp* | 2.3524095679033025e-10 | 0.78225611 | TAM II early versus naïve |
| *Tpi1* | 5.925266964710172e-11 | 0.77975535 | TAM II early versus naïve |
| *Rpl10a-ps1* | 1.8925577229481506e-8 | 0.77872724 | TAM II early versus naïve |
| *Ifitm2* | 4.8738794835717355e-14 | 0.77736812 | TAM II early versus naïve |
| *Rbms1* | 2.040526164645964e-13 | 0.77708598 | TAM II early versus naïve |
| *Ndufa13* | 1.9125687350755216e-9 | 0.77404783 | TAM II early versus naïve |
| *Got1* | 1.5261147666009045e-14 | 0.77127353 | TAM II early versus naïve |
| *Rpl3* | 2.4538834219955717e-9 | 0.76935628 | TAM II early versus naïve |
| *Gatm* | 5.906203219726021e-9 | 0.76833669 | TAM II early versus naïve |
| *Gbp8* | 9.680654020467011e-14 | 0.76406921 | TAM II early versus naïve |
| *Slk* | 1.1881804930016021e-11 | 0.76224941 | TAM II early versus naïve |
| *Tax1bp1* | 6.495380325370837e-6 | 0.75770515 | TAM II early versus naïve |
| *Lamp1* | 5.276293079904251e-7 | 0.75744346 | TAM II early versus naïve |
| *Bag1* | 3.7211913713883184e-8 | 0.75584427 | TAM II early versus naïve |
| *Spop* | 1.0439836138921856e-9 | 0.75535386 | TAM II early versus naïve |
| *Rps15a-ps5* | 2.2048366416894763e-10 | 0.75275385 | TAM II early versus naïve |
| *Zfp622* | 2.900003652802599e-9 | 0.75228454 | TAM II early versus naïve |
| *Gm12338* | 1.8307865634258365e-10 | 0.75170773 | TAM II early versus naïve |
| *Ifi211* | 4.877703038620863e-14 | 0.75071474 | TAM II early versus naïve |
| *Atp5g3* | 1.487490562107067e-9 | 0.74749134 | TAM II early versus naïve |
| *Axl* | 1.7422979266129495e-13 | 0.74668908 | TAM II early versus naïve |
| *Lst1* | 2.5202674215761062e-8 | 0.74462382 | TAM II early versus naïve |
| *Irf7* | 6.320653459546458e-15 | 0.74398676 | TAM II early versus naïve |
| *Uqcrb* | 3.2237643419126584e-10 | 0.74178858 | TAM II early versus naïve |
| *Pomp* | 2.0472561036799157e-10 | 0.73929766 | TAM II early versus naïve |
| *Psmb10* | 1.499147864633912e-9 | 0.73829641 | TAM II early versus naïve |
| *Psma1* | 1.9676255973941069e-10 | 0.73662926 | TAM II early versus naïve |
| *Uqcrq* | 1.1724591236725544e-9 | 0.73537677 | TAM II early versus naïve |
| *Hspe1* | 1.2739759872172626e-9 | 0.7353592 | TAM II early versus naïve |
| *Gda* | 1.8965417087825434e-13 | 0.73265666 | TAM II early versus naïve |
| *Ctss* | 3.7580338885575975e-6 | 0.7317027 | TAM II early versus naïve |
| *Uqcr10* | 9.828703464623717e-12 | 0.73064956 | TAM II early versus naïve |
| *Gm12254* | 1.1716863655114962e-10 | 0.72945044 | TAM II early versus naïve |
| *Rps24-ps2* | 1.5001455923207467e-12 | 0.72917937 | TAM II early versus naïve |
| *Atp5h* | 7.309389028782337e-10 | 0.72879659 | TAM II early versus naïve |
| *1810037I17Rik* | 1.4464831098747493e-10 | 0.72848417 | TAM II early versus naïve |
| *Psma4* | 2.03267481718529e-10 | 0.72591075 | TAM II early versus naïve |
| *Atp5c1* | 1.0406320724028325e-8 | 0.72532455 | TAM II early versus naïve |
| *Gm10443* | 1.723536755472063e-11 | 0.72408599 | TAM II early versus naïve |
| *Nfkbiz* | 5.541696422497206e-7 | 0.72384412 | TAM II early versus naïve |
| *Tpd52* | 3.788674305388545e-9 | 0.72065549 | TAM II early versus naïve |
| *Gm6204* | 1.0845495488592403e-7 | 0.7205402 | TAM II early versus naïve |
| *Ass1* | 1.8335485109505227e-13 | 0.7190293 | TAM II early versus naïve |
| *Mpeg1* | 1.5736643599596346e-5 | 0.71784806 | TAM II early versus naïve |
| *Pdia6* | 4.914538364788709e-7 | 0.71763926 | TAM II early versus naïve |
| *Clta* | 2.2525566943807875e-6 | 0.71518565 | TAM II early versus naïve |
| *Csf2ra* | 8.958673992283674e-12 | 0.71390517 | TAM II early versus naïve |
| *Atp5d* | 4.811842083878587e-9 | 0.7134857 | TAM II early versus naïve |
| *Myo5a* | 1.0056985754144523e-11 | 0.71246658 | TAM II early versus naïve |
| *Sp140* | 3.8857305980939574e-10 | 0.71184603 | TAM II early versus naïve |
| *Cd274* | 3.568783921966234e-13 | 0.71175989 | TAM II early versus naïve |
| *Clic4* | 9.1428140907914e-14 | 0.71038369 | TAM II early versus naïve |
| *Gm10073* | 7.337648783145732e-6 | 0.71015276 | TAM II early versus naïve |
| *H2-T22* | 6.0597670691022055e-12 | 0.70984346 | TAM II early versus naïve |
| *Sod2* | 1.9935157244867062e-11 | 0.70825262 | TAM II early versus naïve |
| *Irf1* | 1.864606054974777e-10 | 0.70620256 | TAM II early versus naïve |
| *Atp5b* | 7.033039647481734e-7 | 0.70243616 | TAM II early versus naïve |
| *Ccl12* | 9.16211675141301e-7 | 0.70198184 | TAM II early versus naïve |
| *Parp14* | 4.743759505984975e-9 | 0.69998955 | TAM II early versus naïve |
| *Atp2b1* | 1.4939045903096337e-6 | 0.69876908 | TAM II early versus naïve |
| *Ccnd2* | 5.3199691849401906e-11 | 0.69746754 | TAM II early versus naïve |
| *Malt1* | 1.4306884587639375e-11 | 0.69702567 | TAM II early versus naïve |
| *Myl12b* | 4.875038147957517e-9 | 0.69317208 | TAM II early versus naïve |
| *Samd9l* | 6.953134640217693e-13 | 0.69255759 | TAM II early versus naïve |
| *Oaz1* | 1.3475688845633286e-8 | 0.69205112 | TAM II early versus naïve |
| *Cd83* | 1.7292112433865303e-6 | 0.691102 | TAM II early versus naïve |
| *Tceb2* | 2.1034740482257443e-8 | 0.69039556 | TAM II early versus naïve |
| *Emb* | 2.2209615034477905e-14 | 0.68816536 | TAM II early versus naïve |
| *Eif5a* | 3.203088587506105e-7 | 0.68804346 | TAM II early versus naïve |
| *Sdhb* | 8.933901719905929e-9 | 0.6866766 | TAM II early versus naïve |
| *Gbp3* | 1.8438785793838353e-8 | 0.68601455 | TAM II early versus naïve |
| *Gm11560* | 1.0087959315228282e-8 | 0.68600669 | TAM II early versus naïve |
| *Msn* | 6.784803767877121e-7 | 0.68436084 | TAM II early versus naïve |
| *Ranbp2* | 1.8186344958761156e-9 | 0.68369146 | TAM II early versus naïve |
| *Trim25* | 9.41268794770319e-14 | 0.68297973 | TAM II early versus naïve |
| *Calr* | 4.990836616942417e-5 | 0.68154619 | TAM II early versus naïve |
| *Oasl2* | 4.767426182261997e-14 | 0.68083058 | TAM II early versus naïve |
| *Atp5f1* | 5.728118557369848e-7 | 0.68047845 | TAM II early versus naïve |
| *AU020206* | 6.169599623596896e-7 | 0.6802403 | TAM II early versus naïve |
| *Atp5a1* | 4.273656565650746e-6 | 0.67998021 | TAM II early versus naïve |
| *Gm10169* | 6.888428459264282e-11 | 0.67936884 | TAM II early versus naïve |
| *Mt1* | 4.56846931911181e-4 | 0.67855645 | TAM II early versus naïve |
| *Gm13835* | 1.6401375605421924e-8 | 0.67708259 | TAM II early versus naïve |
| *Ifi207* | 4.5695974965069854e-11 | 0.67541893 | TAM II early versus naïve |
| *Csf2rb* | 4.0858154223180974e-10 | 0.6732299 | TAM II early versus naïve |
| *Ddx21* | 1.210507986851052e-7 | 0.6721526 | TAM II early versus naïve |
| *Glrx* | 2.97587464684889e-11 | 0.67184127 | TAM II early versus naïve |
| *Ndufc1* | 2.40210755119351e-10 | 0.67068589 | TAM II early versus naïve |
| *Ctsh* | 9.217270741255966e-7 | 0.66999152 | TAM II early versus naïve |
| *Hsp90b1* | 1.739413498988485e-4 | 0.66978503 | TAM II early versus naïve |
| *Rpl18* | 2.8014296997193157e-11 | 0.66875779 | TAM II early versus naïve |
| *Psmb4* | 1.6833902486837743e-9 | 0.66855411 | TAM II early versus naïve |
| *Ran* | 1.600602214604118e-8 | 0.6670152 | TAM II early versus naïve |
| *Smarca5* | 4.945288665114459e-9 | 0.66684732 | TAM II early versus naïve |
| *Sirpb1c* | 1.2984593839971316e-12 | 0.66609156 | TAM II early versus naïve |
| *Naca* | 1.694327597204986e-8 | 0.66589261 | TAM II early versus naïve |
| *Vasp* | 3.668114548520111e-8 | 0.66550496 | TAM II early versus naïve |
| *Grn* | 8.825978306228213e-5 | 0.66383368 | TAM II early versus naïve |
| *Tmbim6* | 6.164256966707488e-6 | 0.66248799 | TAM II early versus naïve |
| *Tubb5* | 5.156040821591912e-7 | 0.66180922 | TAM II early versus naïve |
| *Atp6v1f* | 2.92591735002036e-8 | 0.66129611 | TAM II early versus naïve |
| *Snx3* | 3.9448515476752136e-8 | 0.66009701 | TAM II early versus naïve |
| *H2-Q6* | 4.473699661649518e-12 | 0.65959584 | TAM II early versus naïve |
| *Cct2* | 1.4432212126892267e-8 | 0.65947731 | TAM II early versus naïve |
| *Eif4a1* | 2.002905480728118e-8 | 0.65455465 | TAM II early versus naïve |
| *Sec61b* | 2.364169150087278e-7 | 0.65388647 | TAM II early versus naïve |
| *Nop58* | 3.5768760822738034e-8 | 0.65339188 | TAM II early versus naïve |
| *Nlrp3* | 1.1825301488898323e-9 | 0.65315357 | TAM II early versus naïve |
| *Smdt1* | 4.726576911308034e-9 | 0.65139522 | TAM II early versus naïve |
| *Gm6265* | 5.148865832124338e-9 | 0.65094061 | TAM II early versus naïve |
| *Ube2k* | 5.368895581158026e-8 | 0.65067444 | TAM II early versus naïve |
| *Ppig* | 4.437572507596155e-8 | 0.6505328 | TAM II early versus naïve |
| *Ndufa3* | 3.614574146711404e-8 | 0.65047087 | TAM II early versus naïve |
| *Cdc42* | 1.2661627331752198e-5 | 0.65035334 | TAM II early versus naïve |
| *Lyz1* | 3.2214165657472255e-11 | 0.64988721 | TAM II early versus naïve |
| *Shisa5* | 2.1226525255150864e-8 | 0.64946142 | TAM II early versus naïve |
| *Tmem256* | 2.9454519179261907e-8 | 0.6487959 | TAM II early versus naïve |
| *Snx10* | 6.195440317685907e-8 | 0.64814446 | TAM II early versus naïve |
| *Ak2* | 3.0576109370004824e-11 | 0.64786403 | TAM II early versus naïve |
| *Gm2a* | 2.0721631810980854e-7 | 0.64741334 | TAM II early versus naïve |
| *Ndufv3* | 2.4681969289752543e-10 | 0.64724348 | TAM II early versus naïve |
| *Sri* | 4.147575169939958e-12 | 0.64588534 | TAM II early versus naïve |
| *Ccnd1* | 5.536207089790803e-7 | 0.6455137 | TAM II early versus naïve |
| *Aprt* | 4.135382415498904e-12 | 0.64420983 | TAM II early versus naïve |
| *Ranbp1* | 2.7775876029136026e-6 | 0.64337058 | TAM II early versus naïve |
| *Prelid1* | 5.351924084108804e-11 | 0.64218832 | TAM II early versus naïve |
| *Zfp106* | 2.676572156970782e-8 | 0.64202753 | TAM II early versus naïve |
| *Bola2* | 2.8844641485853343e-10 | 0.64138565 | TAM II early versus naïve |
| *Rpsa* | 1.3719452643585724e-9 | 0.63956219 | TAM II early versus naïve |
| *Igsf6* | 1.6957024484115572e-7 | 0.63933312 | TAM II early versus naïve |
| *Cox5b* | 2.824582977517197e-8 | 0.63721745 | TAM II early versus naïve |
| *Eif3k* | 2.3606506073609527e-10 | 0.6368965 | TAM II early versus naïve |
| *Nap1l1* | 1.2516076681649618e-10 | 0.63485185 | TAM II early versus naïve |
| *Irgm1* | 2.1604438788115324e-8 | 0.63451822 | TAM II early versus naïve |
| *Caprin1* | 2.990364381012375e-8 | 0.63217749 | TAM II early versus naïve |
| *Gpnmb* | 5.594715653428564e-10 | 0.63174024 | TAM II early versus naïve |
| *Eif1a* | 2.759525647213445e-10 | 0.63169493 | TAM II early versus naïve |
| *Gm1966* | 1.8484922475580443e-9 | 0.63146908 | TAM II early versus naïve |
| *Dusp1* | 1.1972960089035077e-6 | 0.6307618 | TAM II early versus naïve |
| *Rel* | 5.581925341860577e-6 | 0.63069902 | TAM II early versus naïve |
| *Nr4a1* | 9.175537500036276e-10 | 0.63036913 | TAM II early versus naïve |
| *Gm43712* | 4.182885977477656e-10 | 0.62980369 | TAM II early versus naïve |
| *Hnrnpa2b1* | 1.182517540793971e-4 | 0.62970702 | TAM II early versus naïve |
| *Ifi205* | 5.294512437060878e-11 | 0.62965026 | TAM II early versus naïve |
| *Tap1* | 1.8909065587649023e-11 | 0.62890446 | TAM II early versus naïve |
| *Ier3* | 2.6425600988784148e-5 | 0.627279 | TAM II early versus naïve |
| *Gm9800* | 1.1300564585537553e-7 | 0.62468013 | TAM II early versus naïve |
| *Ndufb7* | 5.636325986982834e-9 | 0.62451729 | TAM II early versus naïve |
| *Ndufa1* | 3.372605146498003e-10 | 0.6245137 | TAM II early versus naïve |
| *Ndufa2* | 9.113859337959803e-7 | 0.62378726 | TAM II early versus naïve |
| *Tkt* | 2.643868522889144e-11 | 0.62369955 | TAM II early versus naïve |
| *Neat1* | 6.241916488888222e-7 | 0.62363401 | TAM II early versus naïve |
| *Fam96a* | 1.7370326737405928e-10 | 0.6190316 | TAM II early versus naïve |
| *Hspd1* | 4.483345719337071e-7 | 0.61805393 | TAM II early versus naïve |
| *Nfkbie* | 2.7021251734257723e-10 | 0.61616561 | TAM II early versus naïve |
| *Ywhab* | 3.0180455377642264e-5 | 0.61604877 | TAM II early versus naïve |
| *Tmem123* | 3.162996352425131e-10 | 0.61504558 | TAM II early versus naïve |
| *Gas5* | 4.583951311946277e-6 | 0.61477502 | TAM II early versus naïve |
| *Spp1* | 4.750529778923445e-8 | 0.61446262 | TAM II early versus naïve |
| *Kif5b* | 2.533556651065386e-7 | 0.61343214 | TAM II early versus naïve |
| *Pfdn5* | 3.583804706193178e-6 | 0.61335898 | TAM II early versus naïve |
| *Selenok* | 2.278979580514459e-7 | 0.6126817 | TAM II early versus naïve |
| *Sp100* | 1.1223552320297137e-10 | 0.61054304 | TAM II early versus naïve |
| *Eif2s2* | 8.253971915771078e-7 | 0.61030759 | TAM II early versus naïve |
| *Slc7a2* | 3.5657710884966083e-10 | 0.60859899 | TAM II early versus naïve |
| *Atp5o* | 4.5573121275995465e-7 | 0.60726097 | TAM II early versus naïve |
| *Cd44* | 2.714756513763142e-12 | 0.602957 | TAM II early versus naïve |
| *Atp6v1e1* | 2.8821638001272305e-8 | 0.60216756 | TAM II early versus naïve |
| *Myeov2* | 1.3688061444668843e-6 | 0.60205371 | TAM II early versus naïve |
| *Edf1* | 1.046638956361756e-6 | 0.60179844 | TAM II early versus naïve |
| *Klf6* | 9.307208110915813e-4 | 0.59950451 | TAM II early versus naïve |
| *Gadd45b* | 1.2683855604587472e-9 | 0.59941741 | TAM II early versus naïve |
| *Gm14681* | 5.347613163653645e-9 | 0.59912322 | TAM II early versus naïve |
| *Naaa* | 5.5471474148126614e-8 | 0.59879063 | TAM II early versus naïve |
| *Serp1* | 5.044369250217151e-7 | 0.59859544 | TAM II early versus naïve |
| *Gm15148* | 2.367277311120692e-8 | 0.59708766 | TAM II early versus naïve |
| *Rsad2* | 1.756109907320544e-9 | 0.59604388 | TAM II early versus naïve |
| *Atp6ap2* | 9.542898457979838e-7 | 0.59592291 | TAM II early versus naïve |
| *Calm3* | 5.545932571698977e-10 | 0.59325463 | TAM II early versus naïve |
| *Eea1* | 1.6605988017146077e-8 | 0.59196812 | TAM II early versus naïve |
| *Snx2* | 3.0304600495928017e-5 | 0.59067739 | TAM II early versus naïve |
| *Banf1* | 1.0995711306406737e-7 | 0.59061256 | TAM II early versus naïve |
| *Mki67* | 2.04869744205524e-8 | 0.58919731 | TAM II early versus naïve |
| *Pygl* | 5.3513950499505586e-11 | 0.58849158 | TAM II early versus naïve |
| *Bhlhe40* | 5.2658869509320463e-11 | 0.58769984 | TAM II early versus naïve |
| *Mrpl52* | 3.5048763513489403e-7 | 0.58705089 | TAM II early versus naïve |
| *Gnai2* | 1.4145987001882044e-5 | 0.5854889 | TAM II early versus naïve |
| *Il18bp* | 2.7499751492734667e-11 | 0.58381293 | TAM II early versus naïve |
| *Cdkn1b* | 7.406634658039881e-9 | 0.58378068 | TAM II early versus naïve |
| *Arf5* | 8.789250492078737e-9 | 0.58219459 | TAM II early versus naïve |
| *Dbi* | 3.188733411332712e-6 | 0.58218131 | TAM II early versus naïve |
| *Rps11-ps2* | 3.413772860342885e-8 | 0.58120443 | TAM II early versus naïve |
| *Eif4g1* | 2.040928891203579e-6 | 0.57903807 | TAM II early versus naïve |
| *Gsn* | 2.7128054421218598e-12 | 0.57875566 | TAM II early versus naïve |
| *Cfb* | 9.545628695351596e-11 | 0.57825941 | TAM II early versus naïve |
| *Chd7* | 1.2872255366939722e-5 | 0.57796285 | TAM II early versus naïve |
| *Eif3c* | 1.3773350432541323e-5 | 0.57778769 | TAM II early versus naïve |
| *Ndel1* | 2.4890463206337808e-9 | 0.5763484 | TAM II early versus naïve |
| *Eef1g* | 1.3435929160520103e-7 | 0.57562044 | TAM II early versus naïve |
| *Pitpna* | 1.815338202207092e-6 | 0.57408452 | TAM II early versus naïve |
| *Rpl22-ps1* | 1.3467525736519477e-7 | 0.57404678 | TAM II early versus naïve |
| *Csde1* | 2.3864890206536767e-007 | 0.57370328 | TAM II early versus naïve |
| *Lsm4* | 4.417328071835689e-9 | 0.57237382 | TAM II early versus naïve |
| *Hnrnpd* | 3.5446572394039175e-5 | 0.57102147 | TAM II early versus naïve |
| *Itgb2* | 5.644803871183521e-7 | 0.57088484 | TAM II early versus naïve |
| *Gm2000* | 2.976092839668323e-10 | 0.57007273 | TAM II early versus naïve |
| *Cd84* | 1.4006797970621303e-4 | 0.56897285 | TAM II early versus naïve |
| *Ptma* | 1.1306833847643249e-4 | 0.56877997 | TAM II early versus naïve |
| *Snrpd3* | 1.3269082085800584e-6 | 0.56720353 | TAM II early versus naïve |
| *Ninj1* | 2.7616703372086608e-8 | 0.56541743 | TAM II early versus naïve |
| *Ppib* | 1.2195584833870808e-5 | 0.56537482 | TAM II early versus naïve |
| *Zfas1* | 5.098572335131902e-8 | 0.56513368 | TAM II early versus naïve |
| *Rps25* | 5.529135541151184e-8 | 0.5650129 | TAM II early versus naïve |
| *Ccl9* | 1.2327351756837734e-5 | 0.56463237 | TAM II early versus naïve |
| *Sf3b5* | 1.4340306236044597e-9 | 0.56444477 | TAM II early versus naïve |
| *Nme1* | 1.8433362335678564e-10 | 0.56394753 | TAM II early versus naïve |
| *Tob2* | 5.588496484997429e-9 | 0.56356819 | TAM II early versus naïve |
| *Gm8186* | 4.9368349663966384e-11 | 0.56299181 | TAM II early versus naïve |
| *Ndufa5* | 1.4000009926716365e-8 | 0.56257491 | TAM II early versus naïve |
| *Cdc37* | 7.844528157294854e-8 | 0.56179463 | TAM II early versus naïve |
| *Ndufa7* | 1.1321743532256341e-7 | 0.56179206 | TAM II early versus naïve |
| *Oas1a* | 3.2714089519443105e-10 | 0.55940481 | TAM II early versus naïve |
| *Epsti1* | 1.7087978247510082e-7 | 0.5589892 | TAM II early versus naïve |
| *Ubl5* | 8.926846315835307e-7 | 0.55878841 | TAM II early versus naïve |
| *Nedd8* | 3.7880055152055094e-7 | 0.55875228 | TAM II early versus naïve |
| *Etf1* | 7.117081420157182e-7 | 0.55732352 | TAM II early versus naïve |
| *Dnajb11* | 2.559134992762075e-8 | 0.55727044 | TAM II early versus naïve |
| *Vamp8* | 1.032809755566394e-4 | 0.55692615 | TAM II early versus naïve |
| *Dnaja2* | 5.35361473378965e-6 | 0.55688527 | TAM II early versus naïve |
| *Tnfaip3* | 1.5437438049193313e-7 | 0.55624977 | TAM II early versus naïve |
| *Cmpk1* | 7.703076366112377e-7 | 0.55505499 | TAM II early versus naïve |
| *Ndufb8* | 7.68653459776838e-7 | 0.55369684 | TAM II early versus naïve |
| *Snrpg* | 7.370459161996819e-10 | 0.55209596 | TAM II early versus naïve |
| *Arl5c* | 7.864672032695491e-8 | 0.5517368 | TAM II early versus naïve |
| *Mir692-1* | 1.2531916195629662e-9 | 0.55130349 | TAM II early versus naïve |
| *Rps15a-ps7* | 2.949906188492529e-9 | 0.55073354 | TAM II early versus naïve |
| *Gpr171* | 9.877972216245489e-10 | 0.54979651 | TAM II early versus naïve |
| *Ssr3* | 6.741070082206029e-7 | 0.54767628 | TAM II early versus naïve |
| *Svbp* | 1.0741983281030263e-8 | 0.54758178 | TAM II early versus naïve |
| *Tgif1* | 4.358982310225764e-8 | 0.54745581 | TAM II early versus naïve |
| *Gm11966* | 4.319752209436528e-6 | 0.54741202 | TAM II early versus naïve |
| *D8Ertd738e* | 1.215511849012378e-6 | 0.54730234 | TAM II early versus naïve |
| *Arpp19* | 8.272487638628926e-8 | 0.54660071 | TAM II early versus naïve |
| *Klf4* | 3.453353984412346e-5 | 0.54520663 | TAM II early versus naïve |
| *Ell2* | 3.2410187615229556e-9 | 0.54505301 | TAM II early versus naïve |
| *Eif2ak2* | 3.7898873876084937e-8 | 0.54374008 | TAM II early versus naïve |
| *Ndufs6* | 7.065237609090034e-9 | 0.54336096 | TAM II early versus naïve |
| *Tmod3* | 8.17350240284944e-7 | 0.54245285 | TAM II early versus naïve |
| *Cct6a* | 2.080245473262565e-5 | 0.54203439 | TAM II early versus naïve |
| *Rpl6* | 8.73460639073483e-6 | 0.54114308 | TAM II early versus naïve |
| *Capzb* | 1.9796250293459505e-4 | 0.54084932 | TAM II early versus naïve |
| *Mir703* | 5.148181501724162e-9 | 0.53972078 | TAM II early versus naïve |
| *Gm6542* | 2.6902326231022977e-9 | 0.53937372 | TAM II early versus naïve |
| *Psma5* | 8.845934910376844e-7 | 0.53871073 | TAM II early versus naïve |
| *Eif3f* | 2.4960099630447544e-6 | 0.53856567 | TAM II early versus naïve |
| *Bnip2* | 3.1898078213773145e-6 | 0.53835248 | TAM II early versus naïve |
| *Tmem14c* | 5.536187420399878e-8 | 0.53768926 | TAM II early versus naïve |
| *Arrdc4* | 1.2519081556073055e-9 | 0.5360499 | TAM II early versus naïve |
| *Rpl22l1* | 4.85587333907745e-6 | 0.53439121 | TAM II early versus naïve |
| *Txndc17* | 5.399287073900255e-7 | 0.53389443 | TAM II early versus naïve |
| *Cnbp* | 1.7194717603390326e-4 | 0.53346527 | TAM II early versus naïve |
| *Abracl* | 6.493950021052047e-7 | 0.53341851 | TAM II early versus naïve |
| *Hspa9* | 3.89332839132116e-7 | 0.53219327 | TAM II early versus naïve |
| *Nop10* | 2.3113186686185155e-7 | 0.53125534 | TAM II early versus naïve |
| *Cwc15* | 1.0461908338466947e-5 | 0.53114861 | TAM II early versus naïve |
| *Atad2* | 3.5637705052112286e-8 | 0.53112526 | TAM II early versus naïve |
| *Supt16* | 6.24618161210921e-8 | 0.5304819 | TAM II early versus naïve |
| *Atp6v0e* | 1.8464255392154783e-5 | 0.53031708 | TAM II early versus naïve |
| *Gltscr2* | 1.0100811142258077e-8 | 0.52996587 | TAM II early versus naïve |
| *Ghitm* | 3.351892076445851e-6 | 0.52977608 | TAM II early versus naïve |
| *Lamtor2* | 1.5025616256656917e-7 | 0.52869133 | TAM II early versus naïve |
| *Mia2* | 7.557750879198649e-5 | 0.52850226 | TAM II early versus naïve |
| *Rhoa* | 1.7754755443456654e-5 | 0.52846647 | TAM II early versus naïve |
| *Cytip* | 9.622798106693466e-10 | 0.52835638 | TAM II early versus naïve |
| *Tpm3* | 2.9018299690460346e-4 | 0.52809514 | TAM II early versus naïve |
| *Ndufa11* | 2.906004593099961e-9 | 0.52724573 | TAM II early versus naïve |
| *Itga4* | 1.8391266678730524e-9 | 0.52713347 | TAM II early versus naïve |
| *Gm10269* | 4.9705941171599756e-8 | 0.52550827 | TAM II early versus naïve |
| *Manf* | 7.117268510981672e-6 | 0.52547802 | TAM II early versus naïve |
| *Clic1* | 1.3893487208562524e-4 | 0.52542431 | TAM II early versus naïve |
| *Snrpe* | 1.2324521774797671e-7 | 0.5253799 | TAM II early versus naïve |
| *Gm13436* | 1.0306790022352674e-8 | 0.52499415 | TAM II early versus naïve |
| *Cyth1* | 7.411201549241259e-10 | 0.52451752 | TAM II early versus naïve |
| *Uqcrh* | 1.2691462235660259e-6 | 0.52395348 | TAM II early versus naïve |
| *Atp5k* | 8.895746503366958e-7 | 0.52328521 | TAM II early versus naïve |
| *Cox7a2l* | 6.045350248848851e-6 | 0.5219621 | TAM II early versus naïve |
| *Ccl2* | 2.3051986809529792e-6 | 0.52162009 | TAM II early versus naïve |
| *Ifih1* | 6.758243930044007e-6 | 0.5208893 | TAM II early versus naïve |
| *Clec4e* | 1.591260778528378e-8 | 0.52047679 | TAM II early versus naïve |
| *Tmed5* | 6.15635928146913e-5 | 0.52047619 | TAM II early versus naïve |
| *Prpf40a* | 4.69323398948175e-5 | 0.52013865 | TAM II early versus naïve |
| *Slc11a1* | 6.260584248159769e-4 | 0.51973944 | TAM II early versus naïve |
| *Ifi213* | 9.208002615786028e-11 | 0.51953699 | TAM II early versus naïve |
| *Rpl29* | 1.5449628834246025e-5 | 0.51682971 | TAM II early versus naïve |
| *Psmb3* | 4.136117353263395e-7 | 0.51467975 | TAM II early versus naïve |
| *Ucp2* | 5.082839971724159e-7 | 0.51446197 | TAM II early versus naïve |
| *Gbp4* | 1.7212403021807492e-9 | 0.51253851 | TAM II early versus naïve |
| *Gtf2f1* | 3.5792413634761157e-6 | 0.51176504 | TAM II early versus naïve |
| *Ubb* | 5.490835314259217e-4 | 0.51120507 | TAM II early versus naïve |
| *Gm4604* | 1.4077032948599514e-6 | 0.51111019 | TAM II early versus naïve |
| *Ssr4* | 8.517519722160145e-5 | 0.51100862 | TAM II early versus naïve |
| *Gm7266* | 1.0410112416087186e-7 | 0.51079181 | TAM II early versus naïve |
| *AI662270* | 2.3141757562689275e-10 | 0.50928244 | TAM II early versus naïve |
| *RP23-123D6.12* | 6.480787869421791e-8 | 0.50920243 | TAM II early versus naïve |
| *Gm7776* | 1.593681475617545e-6 | 0.50909927 | TAM II early versus naïve |
| *Tceb1* | 1.7816179682783712e-6 | 0.5079691 | TAM II early versus naïve |
| *Scpep1* | 5.200206674882764e-7 | 0.50781458 | TAM II early versus naïve |
| *Mfsd1* | 6.039450167571043e-7 | 0.50688786 | TAM II early versus naïve |
| *P4hb* | 2.0534648909380256e-4 | 0.50663292 | TAM II early versus naïve |
| *Dynlrb1* | 4.178295264164546e-7 | 0.5063001 | TAM II early versus naïve |
| *Pla2g16* | 4.8709881424932985e-9 | 0.50626358 | TAM II early versus naïve |
| *Eif3e* | 1.240058006522687e-7 | 0.50599935 | TAM II early versus naïve |
| *Diaph1* | 4.603565743823831e-8 | 0.50555389 | TAM II early versus naïve |
| *Pf4* | 1.1329391244977712e-6 | 0.50459495 | TAM II early versus naïve |
| *Uqcr11* | 1.9553015857629833e-5 | 0.50387689 | TAM II early versus naïve |
| *Ccl7* | 1.3324956166219938e-5 | 0.50367539 | TAM II early versus naïve |
| *H2afj* | 1.3503389908768379e-7 | 0.50325381 | TAM II early versus naïve |
| *Selenow* | 1.5750333178089906e-7 | 0.50322336 | TAM II early versus naïve |
| *Mtpn* | 2.0981298861690137e-5 | 0.50285413 | TAM II early versus naïve |
| *Cdk11b* | 1.5409677058913782e-4 | 0.502495 | TAM II early versus naïve |
| *Ssb* | 2.2474982869742145e-4 | 0.50248004 | TAM II early versus naïve |
| *Cd47* | 1.3716356294266762e-4 | 0.50241001 | TAM II early versus naïve |
| *Set* | 7.296760846163363e-6 | 0.50231142 | TAM II early versus naïve |
| *Gdi2* | 0.0025817599261940887 | 0.50172359 | TAM II early versus naïve |
| *H2afy* | 7.183392317226065e-6 | 0.50136334 | TAM II early versus naïve |
| *Top2a* | 9.442538903766035e-6 | 0.50083251 | TAM II early versus naïve |
| *Lyz2* | 1.7822553339930554e-11 | 4.13129579 | TAM II late versus naïve |
| *Il1b* | 2.166513049595607e-22 | 3.83270441 | TAM II late versus naïve |
| *Apoe* | 8.32939160239943e-7 | 3.61168069 | TAM II late versus naïve |
| *Fth1* | 2.0160938461882277e-12 | 3.29217535 | TAM II late versus naïve |
| *Spp1* | 7.607409792151002e-17 | 3.06119789 | TAM II late versus naïve |
| *Srgn* | 8.551716972536307e-16 | 2.84396163 | TAM II late versus naïve |
| *Lgals1* | 9.9866958201446e-23 | 2.57946982 | TAM II late versus naïve |
| *Clec7a* | 9.302850274068141e-19 | 2.53685559 | TAM II late versus naïve |
| *H2-D1* | 3.082407950949785e-14 | 2.53653858 | TAM II late versus naïve |
| *Cd74* | 5.199984341855712e-13 | 2.46931532 | TAM II late versus naïve |
| *Nfkbia* | 6.234131038810675e-8 | 2.32730559 | TAM II late versus naïve |
| *Calm1* | 1.2838204984425835e-12 | 2.31235967 | TAM II late versus naïve |
| *Cdkn1a* | 7.334211634670675e-18 | 2.1827907 | TAM II late versus naïve |
| *Ifitm3* | 3.0057761330746077e-19 | 2.15014945 | TAM II late versus naïve |
| *H2-K1* | 3.5301865567315586e-12 | 2.12143328 | TAM II late versus naïve |
| *Lgals3* | 6.480713839479551e-20 | 2.09759704 | TAM II late versus naïve |
| *Rpl32* | 3.1048861507397885e-13 | 2.04643644 | TAM II late versus naïve |
| *H2-Ab1* | 2.1397349139302685e-10 | 1.99902067 | TAM II late versus naïve |
| *Cd14* | 2.1178225865512397e-7 | 1.98348127 | TAM II late versus naïve |
| *Thbs1* | 7.145197176030748e-19 | 1.89598718 | TAM II late versus naïve |
| *Ccrl2* | 3.6874864668040326e-14 | 1.88186966 | TAM II late versus naïve |
| *H2-Aa* | 3.858813583139938e-11 | 1.87952764 | TAM II late versus naïve |
| *Vim* | 3.167281473675296e-17 | 1.86836481 | TAM II late versus naïve |
| *Lilrb4a* | 5.604126228876127e-21 | 1.84202133 | TAM II late versus naïve |
| *Rps26* | 1.2995855544702942e-12 | 1.81759116 | TAM II late versus naïve |
| *Cxcl2* | 1.0954032493908115e-11 | 1.81630032 | TAM II late versus naïve |
| *Cstb* | 2.415008157868233e-12 | 1.7950175 | TAM II late versus naïve |
| *Rps20* | 2.7759489695969676e-12 | 1.79370189 | TAM II late versus naïve |
| *Cd52* | 2.014218900687477e-11 | 1.78206183 | TAM II late versus naïve |
| *Rpl8* | 3.189257206322288e-11 | 1.77408211 | TAM II late versus naïve |
| *H2-Eb1* | 3.660517089157669e-11 | 1.76185458 | TAM II late versus naïve |
| *Tmsb10* | 2.6120820196180787e-16 | 1.73345215 | TAM II late versus naïve |
| *Rplp1* | 1.3192078093794672e-10 | 1.73298228 | TAM II late versus naïve |
| *Arpc1b* | 3.8140713835943975e-13 | 1.73025504 | TAM II late versus naïve |
| *Rpl39* | 2.4006384199033362e-11 | 1.72865185 | TAM II late versus naïve |
| *Rpl4* | 4.084051368977901e-11 | 1.69984415 | TAM II late versus naïve |
| *B2m* | 1.7198813605400908e-10 | 1.67783157 | TAM II late versus naïve |
| *Wfdc17* | 2.8807968741908054e-15 | 1.67728183 | TAM II late versus naïve |
| *Gpx1* | 1.0511064715594869e-10 | 1.67031749 | TAM II late versus naïve |
| *Rps3* | 1.1600757641820585e-10 | 1.6554648 | TAM II late versus naïve |
| *Slfn2* | 3.731409352587182e-13 | 1.63803538 | TAM II late versus naïve |
| *Ifrd1* | 2.593798886882826e-16 | 1.62416668 | TAM II late versus naïve |
| *Rplp0* | 1.5282878365792142e-9 | 1.6096554 | TAM II late versus naïve |
| *Rps5* | 1.2951131077898416e-8 | 1.59740693 | TAM II late versus naïve |
| *Bst2* | 3.813239577373746e-13 | 1.59297786 | TAM II late versus naïve |
| *Crip1* | 6.016406323630608e-19 | 1.58867284 | TAM II late versus naïve |
| *Ncl* | 3.077247759243436e-8 | 1.58636769 | TAM II late versus naïve |
| *Cebpb* | 1.2800551798350566e-8 | 1.57084434 | TAM II late versus naïve |
| *Ifi27l2a* | 1.0224516821740245e-11 | 1.56049276 | TAM II late versus naïve |
| *Fxyd5* | 2.7096815143688386e-19 | 1.54229719 | TAM II late versus naïve |
| *Pfn1* | 2.197190060298028e-7 | 1.50424806 | TAM II late versus naïve |
| *Ms4a6c* | 1.4299428771106266e-12 | 1.49579866 | TAM II late versus naïve |
| *Ctsb* | 0.001068937785218456 | 1.47494855 | TAM II late versus naïve |
| *Pim1* | 5.873574541712737e-17 | 1.47292324 | TAM II late versus naïve |
| *Iqgap1* | 3.835518976068814e-13 | 1.47150949 | TAM II late versus naïve |
| *Rack1* | 6.251129801833987e-10 | 1.46897872 | TAM II late versus naïve |
| *Rps9* | 7.291449703208529e-10 | 1.46291945 | TAM II late versus naïve |
| *Prdx1* | 6.937107969440739e-9 | 1.45709159 | TAM II late versus naïve |
| *Gm9794* | 5.1280014632570815e-9 | 1.45187631 | TAM II late versus naïve |
| *Tmsb4x* | 9.231164878654169e-10 | 1.44702091 | TAM II late versus naïve |
| *Ccl6* | 5.52235139289816e-6 | 1.44294402 | TAM II late versus naïve |
| *Shfm1* | 4.673039360420287e-11 | 1.43473321 | TAM II late versus naïve |
| *Mcl1* | 1.5234561084218263e-10 | 1.43437512 | TAM II late versus naïve |
| *Sh3bgrl3* | 2.41638082253529e-9 | 1.43422228 | TAM II late versus naïve |
| *Wdr89* | 4.879808574302974e-8 | 1.43110287 | TAM II late versus naïve |
| *Tgfbi* | 2.060675406571873e-13 | 1.41651954 | TAM II late versus naïve |
| *Ifi30* | 3.373298735353179e-15 | 1.40927535 | TAM II late versus naïve |
| *Hsp90ab1* | 6.72925784589023e-8 | 1.40515945 | TAM II late versus naïve |
| *Arpc5* | 2.2595520911995092e-9 | 1.40232585 | TAM II late versus naïve |
| *Rps13-ps2* | 3.2129836242604706e-8 | 1.39462562 | TAM II late versus naïve |
| *Rps2* | 1.9310439931280393e-8 | 1.38941081 | TAM II late versus naïve |
| *Prdx5* | 1.087546804299404e-9 | 1.38691885 | TAM II late versus naïve |
| *Rps24* | 7.489223521812311e-9 | 1.38619109 | TAM II late versus naïve |
| *Plek* | 1.503744216402326e-7 | 1.38248482 | TAM II late versus naïve |
| *Fabp5* | 1.6288853550879508e-10 | 1.34566816 | TAM II late versus naïve |
| *Txn1* | 2.1327250371973702e-10 | 1.3434216 | TAM II late versus naïve |
| *Sub1* | 4.431980027152993e-12 | 1.33232954 | TAM II late versus naïve |
| *Ybx1* | 5.042042832783617e-7 | 1.3306289 | TAM II late versus naïve |
| *Ier3* | 1.4742591456753465e-7 | 1.32657859 | TAM II late versus naïve |
| *Rps14* | 4.240490271555946e-7 | 1.32033278 | TAM II late versus naïve |
| *Cyba* | 1.1972209500648884e-8 | 1.30735527 | TAM II late versus naïve |
| *Sdcbp* | 5.996601816576895e-10 | 1.30612418 | TAM II late versus naïve |
| *Rpl31-ps8* | 1.1730130636701628e-10 | 1.30278074 | TAM II late versus naïve |
| *Dab2* | 2.9695044919982687e-12 | 1.2979367 | TAM II late versus naïve |
| *Rps25-ps1* | 2.077203565679935e-9 | 1.29419467 | TAM II late versus naïve |
| *Atox1* | 2.9760437076218234e-11 | 1.29221884 | TAM II late versus naïve |
| *Ifitm2* | 5.929177756745502e-18 | 1.27974501 | TAM II late versus naïve |
| *Cox4i1* | 8.025754117179654e-8 | 1.26938653 | TAM II late versus naïve |
| *Rpl41* | 2.2725720194869963e-7 | 1.26753132 | TAM II late versus naïve |
| *Arpc2* | 9.698126794448089e-9 | 1.26467426 | TAM II late versus naïve |
| *Rpl22* | 1.634796534463386e-7 | 1.26147102 | TAM II late versus naïve |
| *Bcl2a1b* | 1.2598828058541124e-7 | 1.25423231 | TAM II late versus naïve |
| *Mif* | 1.3198698189907498e-11 | 1.24804893 | TAM II late versus naïve |
| *Ctsc* | 8.359474097172264e-6 | 1.24483822 | TAM II late versus naïve |
| *Akr1a1* | 8.270124207361552e-9 | 1.22581504 | TAM II late versus naïve |
| *Myl12a* | 1.1181272817218626e-10 | 1.22499714 | TAM II late versus naïve |
| *Rpl14* | 2.6640950078800614e-8 | 1.22435026 | TAM II late versus naïve |
| *Actb* | 1.2014921602763912e-8 | 1.2185176 | TAM II late versus naïve |
| *Cotl1* | 2.2235603831133366e-6 | 1.21514092 | TAM II late versus naïve |
| *Gm6977* | 3.452199320319138e-6 | 1.2124465 | TAM II late versus naïve |
| *Rps24-ps3* | 6.532520119847702e-7 | 1.20955973 | TAM II late versus naïve |
| *Msrb1* | 4.1916284495219964e-17 | 1.20601288 | TAM II late versus naïve |
| *Cybb* | 9.207884307416624e-15 | 1.19979954 | TAM II late versus naïve |
| *Gpnmb* | 2.866918792639347e-10 | 1.19882972 | TAM II late versus naïve |
| *Gabarap* | 1.026212291355818e-8 | 1.18586526 | TAM II late versus naïve |
| *Osm* | 1.9809324828922315e-12 | 1.18567169 | TAM II late versus naïve |
| *Rps16-ps2* | 1.7905295198920829e-9 | 1.18410226 | TAM II late versus naïve |
| *Npm1* | 3.514180581822716e-8 | 1.18241015 | TAM II late versus naïve |
| *Gm5963* | 5.5720656102093916e-8 | 1.17980595 | TAM II late versus naïve |
| *Pkm* | 1.4477409835522326e-7 | 1.17645211 | TAM II late versus naïve |
| *Ly6e* | 2.1056733381982286e-7 | 1.15536087 | TAM II late versus naïve |
| *Rpl26* | 4.750543573496396e-8 | 1.15121999 | TAM II late versus naïve |
| *Nr4a1* | 8.291094045465865e-12 | 1.15023255 | TAM II late versus naïve |
| *Rpsa-ps10* | 1.5144379303145351e-6 | 1.14720159 | TAM II late versus naïve |
| *Arg1* | 7.980613704570434e-7 | 1.14617573 | TAM II late versus naïve |
| *Id2* | 4.7276293255973425e-7 | 1.14496352 | TAM II late versus naïve |
| *Fcer1g* | 3.7723022692066756e-6 | 1.1395781 | TAM II late versus naïve |
| *Gm16580* | 3.705281689813879e-7 | 1.13743925 | TAM II late versus naïve |
| *Litaf* | 1.774620457768911e-9 | 1.13664573 | TAM II late versus naïve |
| *Aldoa* | 6.408074697201546e-10 | 1.13629077 | TAM II late versus naïve |
| *Rps10-ps1* | 3.996715097202816e-8 | 1.12200451 | TAM II late versus naïve |
| *Rps15* | 1.9044575975728322e-6 | 1.11856821 | TAM II late versus naïve |
| *Rpl23* | 5.769718739940353e-9 | 1.11187106 | TAM II late versus naïve |
| *Rps19* | 1.2550628008499758e-6 | 1.10329392 | TAM II late versus naïve |
| *S100a11* | 4.4863582634351386e-14 | 1.09862415 | TAM II late versus naïve |
| *Tagln2* | 2.807066343483097e-11 | 1.09480361 | TAM II late versus naïve |
| *Gm10275* | 3.0570362884780087e-6 | 1.09407881 | TAM II late versus naïve |
| *Nfkbiz* | 6.816765614240016e-8 | 1.09117847 | TAM II late versus naïve |
| *Ms4a7* | 2.949553715882809e-13 | 1.08813915 | TAM II late versus naïve |
| *Gm6030* | 2.4503902013596665e-9 | 1.08179822 | TAM II late versus naïve |
| *Anxa5* | 5.92525275425088e-11 | 1.07980294 | TAM II late versus naïve |
| *Rpl34* | 6.000091161735248e-7 | 1.07778826 | TAM II late versus naïve |
| *Cox7b* | 1.0719749905828206e-9 | 1.0774101 | TAM II late versus naïve |
| *Rps15a* | 1.666145116848356e-7 | 1.07700015 | TAM II late versus naïve |
| *Eef1b2* | 8.882732035598972e-7 | 1.07504904 | TAM II late versus naïve |
| *Plbd1* | 6.1630057366379e-14 | 1.07271526 | TAM II late versus naïve |
| *Tpr* | 1.607426358579326e-7 | 1.06737979 | TAM II late versus naïve |
| *Gm10288* | 8.18223185148377e-8 | 1.06658374 | TAM II late versus naïve |
| *Tyrobp* | 6.11144045864075e-7 | 1.06399484 | TAM II late versus naïve |
| *App* | 1.0013522343391668e-6 | 1.06007461 | TAM II late versus naïve |
| *Ptgs2* | 4.164953194635773e-11 | 1.05625924 | TAM II late versus naïve |
| *Plac8* | 3.655665778202591e-11 | 1.05192417 | TAM II late versus naïve |
| *Ccr1* | 4.476550985398039e-13 | 1.04955019 | TAM II late versus naïve |
| *Npc2* | 3.408007414822032e-4 | 1.04328289 | TAM II late versus naïve |
| *Serbp1* | 1.0027507423589583e-4 | 1.03961507 | TAM II late versus naïve |
| *Gm5805* | 3.20292069787331e-6 | 1.03742959 | TAM II late versus naïve |
| *Actr3* | 3.184416167983987e-8 | 1.03284257 | TAM II late versus naïve |
| *Ldha* | 4.624592547041453e-8 | 1.03203327 | TAM II late versus naïve |
| *2010107E04Rik* | 2.623206626514118e-11 | 1.03007056 | TAM II late versus naïve |
| *Cox6c* | 4.85766349919309e-7 | 1.0292623 | TAM II late versus naïve |
| *Hnrnpab* | 2.280136580598765e-6 | 1.02904701 | TAM II late versus naïve |
| *Psmb1* | 9.69477976905326e-8 | 1.02776876 | TAM II late versus naïve |
| *Sh3bgrl* | 3.858125190031684e-11 | 1.02725 | TAM II late versus naïve |
| *Psmb8* | 1.5502112373162992e-5 | 1.02110043 | TAM II late versus naïve |
| *H3f3b* | 1.0344274454927619e-4 | 1.02057494 | TAM II late versus naïve |
| *Hspa5* | 1.5040761614842771e-4 | 1.01928571 | TAM II late versus naïve |
| *Psap* | 1.7748724149486053e-4 | 1.01428097 | TAM II late versus naïve |
| *Pla2g7* | 1.4549601910668105e-8 | 1.0096947 | TAM II late versus naïve |
| *Rps27l* | 1.0323294382727642e-6 | 1.00685354 | TAM II late versus naïve |
| *Nfe2l2* | 9.620248320073889e-6 | 1.00663904 | TAM II late versus naïve |
| *Slc25a3* | 6.992925895503071e-8 | 0.9963682 | TAM II late versus naïve |
| *S100a6* | 7.3031136952071e-12 | 0.98394785 | TAM II late versus naïve |
| *Cox6a1* | 1.7235857448851582e-6 | 0.97867896 | TAM II late versus naïve |
| *Cox5a* | 8.498523048731485e-11 | 0.97726691 | TAM II late versus naïve |
| *Gm5835* | 4.528738873777642e-7 | 0.97117863 | TAM II late versus naïve |
| *Gm9843* | 2.3721033260756747e-5 | 0.96987281 | TAM II late versus naïve |
| *Atp5g3* | 4.579262408910547e-9 | 0.96914925 | TAM II late versus naïve |
| *Tspo* | 2.2277989788838712e-9 | 0.96088828 | TAM II late versus naïve |
| *Cd83* | 2.7431896703423166e-5 | 0.95579027 | TAM II late versus naïve |
| *Cfl1* | 1.5466694069394898e-5 | 0.95530312 | TAM II late versus naïve |
| *Acp5* | 4.4838301167274285e-12 | 0.9481109 | TAM II late versus naïve |
| *Pabpc1* | 3.3459925607894556e-5 | 0.94742352 | TAM II late versus naïve |
| *Taldo1* | 5.47905975975689e-8 | 0.94740683 | TAM II late versus naïve |
| *Gm14586* | 1.6451741136147004e-6 | 0.94605567 | TAM II late versus naïve |
| *Eif3a* | 2.607588685893487e-4 | 0.94497384 | TAM II late versus naïve |
| *Nr4a3* | 5.286243369452553e-13 | 0.94448446 | TAM II late versus naïve |
| *Arpc3* | 8.046870224557477e-8 | 0.94100182 | TAM II late versus naïve |
| *Tgm2* | 4.5046274781329574e-11 | 0.93789312 | TAM II late versus naïve |
| *Ly6c2* | 2.850135549039354e-10 | 0.93483209 | TAM II late versus naïve |
| *Anxa2* | 6.418922524006152e-14 | 0.93332089 | TAM II late versus naïve |
| *Dek* | 9.603756869310197e-5 | 0.933045 | TAM II late versus naïve |
| *Ifi207* | 5.764103391662062e-12 | 0.92727294 | TAM II late versus naïve |
| *Cox6b1* | 8.522943813006535e-6 | 0.92716009 | TAM II late versus naïve |
| *Cytip* | 6.02878618111576e-14 | 0.92462653 | TAM II late versus naïve |
| *Plin2* | 2.4655341081909034e-11 | 0.92300356 | TAM II late versus naïve |
| *Gm4204* | 1.4523446603095377e-8 | 0.9194568 | TAM II late versus naïve |
| *Gm5905* | 5.721038089959832e-6 | 0.9149607 | TAM II late versus naïve |
| *Rps26-ps1* | 6.056501105408392e-7 | 0.90052197 | TAM II late versus naïve |
| *Hnrnpm* | 2.2850086982076496e-5 | 0.89770451 | TAM II late versus naïve |
| *Gadd45b* | 2.657668879279112e-10 | 0.8963026 | TAM II late versus naïve |
| *Gm14303* | 9.137830258666542e-6 | 0.8956193 | TAM II late versus naïve |
| *Fosl2* | 6.832263254243955e-17 | 0.89115096 | TAM II late versus naïve |
| *Gm15427* | 3.2785142150729534e-5 | 0.89096221 | TAM II late versus naïve |
| *Grn* | 3.248171657142844e-4 | 0.88898252 | TAM II late versus naïve |
| *Clta* | 3.73195818917406e-5 | 0.88839454 | TAM II late versus naïve |
| *Anp32b* | 1.858786148452507e-5 | 0.88833559 | TAM II late versus naïve |
| *Clec4n* | 8.82969435635389e-12 | 0.88681257 | TAM II late versus naïve |
| *Rps8* | 1.6087467042052086e-8 | 0.88524498 | TAM II late versus naïve |
| *Tnfaip2* | 8.192118081280576e-9 | 0.88253673 | TAM II late versus naïve |
| *Chchd2* | 4.114736308179411e-5 | 0.8816793 | TAM II late versus naïve |
| *Slk* | 5.591639393156364e-7 | 0.87743661 | TAM II late versus naïve |
| *Tpd52* | 1.906568652296965e-9 | 0.87684778 | TAM II late versus naïve |
| *Rpl37* | 9.048377886231134e-7 | 0.87323396 | TAM II late versus naïve |
| *Alox5ap* | 3.970885423616397e-6 | 0.87316254 | TAM II late versus naïve |
| *Gm11478* | 7.020725357969109e-5 | 0.87241483 | TAM II late versus naïve |
| *Chmp4b* | 2.502882737537757e-6 | 0.86706028 | TAM II late versus naïve |
| *Mt1* | 1.8448762003719882e-5 | 0.86678598 | TAM II late versus naïve |
| *Rpl3-ps1* | 1.207137452757141e-4 | 0.86650952 | TAM II late versus naïve |
| *Tuba1c* | 4.066361265339673e-11 | 0.86445833 | TAM II late versus naïve |
| *Cox17* | 1.7277395046712915e-7 | 0.86374746 | TAM II late versus naïve |
| *Mdh2* | 3.4493177798925313e-6 | 0.85916085 | TAM II late versus naïve |
| *Hnrnpu* | 2.2305331811014236e-4 | 0.85652568 | TAM II late versus naïve |
| *Hint1* | 2.00951284648217e-6 | 0.85235422 | TAM II late versus naïve |
| *Emp3* | 2.6657179571768216e-8 | 0.85029775 | TAM II late versus naïve |
| *Msr1* | 8.257509680186814e-12 | 0.8501027 | TAM II late versus naïve |
| *Myo5a* | 1.3954259220787698e-9 | 0.84934544 | TAM II late versus naïve |
| *Psma2* | 1.4830699707750516e-6 | 0.83996937 | TAM II late versus naïve |
| *F13a1* | 5.741315034221296e-9 | 0.83540494 | TAM II late versus naïve |
| *Gm15500* | 5.6924022383372336e-5 | 0.83393611 | TAM II late versus naïve |
| *Nme1* | 5.768762275953269e-9 | 0.83142913 | TAM II late versus naïve |
| *Atp5e* | 4.527173923587372e-5 | 0.83042027 | TAM II late versus naïve |
| *Rpl9-ps6* | 7.635281424195791e-8 | 0.82072412 | TAM II late versus naïve |
| *Cox7a2* | 5.110839632099126e-6 | 0.8170828 | TAM II late versus naïve |
| *Ccl9* | 5.674715941808927e-5 | 0.81233282 | TAM II late versus naïve |
| *Cxcl16* | 9.92726476286671e-8 | 0.80872873 | TAM II late versus naïve |
| *Ccr2* | 2.855720080658108e-10 | 0.80619177 | TAM II late versus naïve |
| *Ubl5* | 9.931762859861847e-7 | 0.80302452 | TAM II late versus naïve |
| *Psma7* | 1.5441183298850553e-4 | 0.79688346 | TAM II late versus naïve |
| *Chd4* | 4.9939330282273466e-5 | 0.79418104 | TAM II late versus naïve |
| *Csf2rb* | 1.7352183181415413e-8 | 0.79338912 | TAM II late versus naïve |
| *Oaz1* | 4.419984670672574e-5 | 0.79141914 | TAM II late versus naïve |
| *Ndufb8* | 3.884073337545865e-7 | 0.78885237 | TAM II late versus naïve |
| *Nampt* | 2.9125272128943615e-9 | 0.78843399 | TAM II late versus naïve |
| *Slc25a5* | 4.298261252486024e-4 | 0.78820966 | TAM II late versus naïve |
| *Rpl14-ps1* | 0.0029564814039854358 | 0.78531635 | TAM II late versus naïve |
| *Mndal* | 5.361751879800269e-10 | 0.78495394 | TAM II late versus naïve |
| *Txnrd1* | 8.127913209129147e-7 | 0.78387219 | TAM II late versus naïve |
| *Atp5b* | 1.2901467982129656e-5 | 0.78288629 | TAM II late versus naïve |
| *Fcgr2b* | 1.5730859539519072e-4 | 0.78057695 | TAM II late versus naïve |
| *sept.07* | 0.002202472345371707 | 0.77947015 | TAM II late versus naïve |
| *Eprs* | 2.7606597565385727e-5 | 0.77928777 | TAM II late versus naïve |
| *Il1rn* | 2.854788639851385e-10 | 0.77357651 | TAM II late versus naïve |
| *Atp5a1* | 2.9415227419785503e-5 | 0.77302112 | TAM II late versus naïve |
| *H2afy* | 2.0547362679054667e-5 | 0.77292487 | TAM II late versus naïve |
| *Mmp14* | 1.1524589886860172e-8 | 0.77287121 | TAM II late versus naïve |
| *Eif3f* | 2.663398652858344e-7 | 0.77088394 | TAM II late versus naïve |
| *Rpl3* | 6.373800939865624e-7 | 0.76821876 | TAM II late versus naïve |
| *Bcl2a1a* | 4.981587188429684e-8 | 0.76818807 | TAM II late versus naïve |
| *Dusp1* | 5.842021380732086e-4 | 0.76525944 | TAM II late versus naïve |
| *Zfp106* | 1.3386929851449674e-6 | 0.76507977 | TAM II late versus naïve |
| *Eef1g* | 1.714546002552732e-6 | 0.76481677 | TAM II late versus naïve |
| *Capns1* | 5.675544532916574e-6 | 0.7622977 | TAM II late versus naïve |
| *Lcp1* | 0.0033182068514299124 | 0.76226843 | TAM II late versus naïve |
| *Atp5f1* | 1.4040488244395342e-4 | 0.76203136 | TAM II late versus naïve |
| *Eno1* | 1.2266090797626069e-6 | 0.75879467 | TAM II late versus naïve |
| *Eef1a1* | 7.038660431355379e-5 | 0.75863843 | TAM II late versus naïve |
| *Atp5h* | 4.695910479007529e-6 | 0.75612602 | TAM II late versus naïve |
| *Ndufa1* | 6.372333925823487e-9 | 0.75321802 | TAM II late versus naïve |
| *Atp6v1a* | 3.528711100492091e-5 | 0.74909661 | TAM II late versus naïve |
| *Atpif1* | 4.225421551489928e-4 | 0.74072795 | TAM II late versus naïve |
| *Csrnp1* | 6.2070712009616e-11 | 0.73854638 | TAM II late versus naïve |
| *S100a4* | 4.4221965920433456e-12 | 0.73730481 | TAM II late versus naïve |
| *Gm6377* | 4.457614049983529e-11 | 0.7371849 | TAM II late versus naïve |
| *Snx3* | 1.7703642064632994e-5 | 0.7367191 | TAM II late versus naïve |
| *Tpt1-ps3* | 1.7789232600572597e-4 | 0.73657106 | TAM II late versus naïve |
| *Hilpda* | 1.9708541027584148e-8 | 0.73547203 | TAM II late versus naïve |
| *Psmb4* | 2.9913507987661653e-6 | 0.73538138 | TAM II late versus naïve |
| *Kras* | 2.2554440209838465e-5 | 0.7347816 | TAM II late versus naïve |
| *Sod2* | 4.744920402583689e-10 | 0.73434373 | TAM II late versus naïve |
| *Cfp* | 2.418309712014749e-8 | 0.73296433 | TAM II late versus naïve |
| *Fn1* | 3.7102120236752915e-10 | 0.73235679 | TAM II late versus naïve |
| *Rps21* | 4.8538090722336527e-4 | 0.73234746 | TAM II late versus naïve |
| *Psme2b* | 8.657148212989639e-5 | 0.73233427 | TAM II late versus naïve |
| *Minos1* | 1.340420349684215e-5 | 0.7316393 | TAM II late versus naïve |
| *Hnrnpd* | 1.655179713046122e-4 | 0.73133013 | TAM II late versus naïve |
| *Hspa8* | 0.0014770721075466342 | 0.73047157 | TAM II late versus naïve |
| *Dusp5* | 2.859448588421073e-10 | 0.73014255 | TAM II late versus naïve |
| *Rnf149* | 8.554576379221445e-9 | 0.7292514 | TAM II late versus naïve |
| *D8Ertd738e* | 2.795412430196682e-4 | 0.7285941 | TAM II late versus naïve |
| *Rpsa* | 1.0703478815670956e-5 | 0.72733338 | TAM II late versus naïve |
| *Il1a* | 2.516601271928093e-7 | 0.72567027 | TAM II late versus naïve |
| *Nlrp3* | 2.5423166944585684e-8 | 0.72466585 | TAM II late versus naïve |
| *Calr* | 0.0015849496945930413 | 0.72451566 | TAM II late versus naïve |
| *Atp5c1* | 1.7217019435812502e-5 | 0.72438618 | TAM II late versus naïve |
| *Clec4e* | 3.591936811577285e-11 | 0.72134183 | TAM II late versus naïve |
| *Gm14681* | 1.270698447903441e-5 | 0.72064116 | TAM II late versus naïve |
| *1810037I17Rik* | 1.0975813541091151e-7 | 0.71846819 | TAM II late versus naïve |
| *Ninj1* | 2.8220945693310236e-6 | 0.71744793 | TAM II late versus naïve |
| *Arl5c* | 1.5438175506122755e-8 | 0.71706808 | TAM II late versus naïve |
| *Mxd1* | 2.7755993325799134e-9 | 0.7162495 | TAM II late versus naïve |
| *Rps10* | 4.304576238181796e-5 | 0.71565035 | TAM II late versus naïve |
| *Gm2a* | 3.4685962881612843e-4 | 0.71408003 | TAM II late versus naïve |
| *Tpi1* | 1.0806065063330952e-6 | 0.71368096 | TAM II late versus naïve |
| *Eef2* | 2.444710131360904e-4 | 0.712646 | TAM II late versus naïve |
| *Pfdn5* | 4.957531020822768e-4 | 0.70667627 | TAM II late versus naïve |
| *Sec61b* | 1.2141530264722719e-4 | 0.70643136 | TAM II late versus naïve |
| *Rpl35a* | 9.108961237894145e-4 | 0.70468818 | TAM II late versus naïve |
| *Atp5j* | 1.4156638889605892e-4 | 0.70466281 | TAM II late versus naïve |
| *Gm4149* | 5.2034788636864624e-5 | 0.70254619 | TAM II late versus naïve |
| *Rel* | 7.236195788262756e-4 | 0.69961261 | TAM II late versus naïve |
| *Bag1* | 2.667816062341089e-5 | 0.69894558 | TAM II late versus naïve |
| *Cfdp1* | 1.6298586446794006e-4 | 0.69839714 | TAM II late versus naïve |
| *Dbi* | 5.023713046194075e-4 | 0.69735368 | TAM II late versus naïve |
| *S100a10* | 3.7511040267782775e-10 | 0.69652286 | TAM II late versus naïve |
| *Sf3b1* | 8.780425497625753e-4 | 0.69532112 | TAM II late versus naïve |
| *Gm8730* | 4.920595852225529e-7 | 0.69396417 | TAM II late versus naïve |
| *Ranbp1* | 1.0728010599242237e-4 | 0.69372333 | TAM II late versus naïve |
| *Gda* | 5.81847961696692e-12 | 0.69218779 | TAM II late versus naïve |
| *Eif3c* | 6.76375142468578e-4 | 0.69057058 | TAM II late versus naïve |
| *Psmd14* | 1.2058504147767301e-6 | 0.69036051 | TAM II late versus naïve |
| *Aprt* | 1.254828625301589e-7 | 0.68991724 | TAM II late versus naïve |
| *Tob2* | 4.687383184330276e-7 | 0.6897663 | TAM II late versus naïve |
| *Runx3* | 5.827440248590963e-14 | 0.68941587 | TAM II late versus naïve |
| *Ifi204* | 1.5395337136108505e-8 | 0.68796599 | TAM II late versus naïve |
| *Rheb* | 9.49857086002121e-8 | 0.68632091 | TAM II late versus naïve |
| *Uqcrq* | 1.1338436173148533e-4 | 0.68556967 | TAM II late versus naïve |
| *Tomm7* | 2.5845323978939146e-6 | 0.68513181 | TAM II late versus naïve |
| *Gm11560* | 3.5355244033227794e-4 | 0.68504381 | TAM II late versus naïve |
| *AA467197* | 1.600081370780104e-8 | 0.6830246 | TAM II late versus naïve |
| *Rpl13* | 1.2859293241439508e-5 | 0.6825609 | TAM II late versus naïve |
| *Rnh1* | 7.827038860200282e-8 | 0.68152116 | TAM II late versus naïve |
| *Nceh1* | 5.504678839540335e-10 | 0.67702861 | TAM II late versus naïve |
| *Ndufa3* | 1.5066223020287862e-5 | 0.67622368 | TAM II late versus naïve |
| *Ostf1* | 1.577691968094321e-5 | 0.67539718 | TAM II late versus naïve |
| *Psme1* | 2.3577959766417554e-5 | 0.67345082 | TAM II late versus naïve |
| *Tpm4* | 3.8794904522850575e-6 | 0.67235676 | TAM II late versus naïve |
| *Hnrnpa2b1* | 0.002952137753985585 | 0.67222449 | TAM II late versus naïve |
| *Pcna* | 3.2704837789020366e-5 | 0.67222287 | TAM II late versus naïve |
| *Rps18* | 6.568153649232196e-5 | 0.67098732 | TAM II late versus naïve |
| *Stk17b* | 2.8708053180300825e-6 | 0.6681714 | TAM II late versus naïve |
| *Tnfaip3* | 4.907318915732571e-7 | 0.66056994 | TAM II late versus naïve |
| *Pde4b* | 5.751571183694838e-9 | 0.65962116 | TAM II late versus naïve |
| *Cox8a* | 4.347041747245833e-4 | 0.65873073 | TAM II late versus naïve |
| *Isg15* | 2.162475151681365e-9 | 0.65523745 | TAM II late versus naïve |
| *Sri* | 7.236799396202404e-7 | 0.65332274 | TAM II late versus naïve |
| *H2-DMa* | 5.363907090408475e-4 | 0.6515601 | TAM II late versus naïve |
| *Serp1* | 0.001106778284969692 | 0.65152005 | TAM II late versus naïve |
| *Hnrnpc* | 0.0013775215178011837 | 0.65106397 | TAM II late versus naïve |
| *Mir22hg* | 4.407282602058656e-12 | 0.65095433 | TAM II late versus naïve |
| *Nr4a2* | 2.166644647527866e-9 | 0.65095433 | TAM II late versus naïve |
| *Fosb* | 0.0026462149069358056 | 0.65009376 | TAM II late versus naïve |
| *Hmox1* | 4.3809710356695775e-5 | 0.64950777 | TAM II late versus naïve |
| *Rpl37rt* | 0.00203060739114488 | 0.6491519 | TAM II late versus naïve |
| *Gm6863* | 0.0010786793316085647 | 0.64816046 | TAM II late versus naïve |
| *BC005537* | 2.0939697032663638e-4 | 0.64278206 | TAM II late versus naïve |
| *Ddx21* | 5.039386903710139e-4 | 0.64047178 | TAM II late versus naïve |
| *Rpl38-ps2* | 3.781717444618658e-6 | 0.64038766 | TAM II late versus naïve |
| *Nop58* | 4.638519640596973e-5 | 0.63880867 | TAM II late versus naïve |
| *Anp32e* | 3.8976210104448285e-5 | 0.63867478 | TAM II late versus naïve |
| *Mir703* | 4.090528859185659e-8 | 0.63796729 | TAM II late versus naïve |
| *Gpr65* | 1.6950140460483157e-8 | 0.63787986 | TAM II late versus naïve |
| *Ms4a6d* | 9.09933414393226e-5 | 0.63747304 | TAM II late versus naïve |
| *Slc6a6* | 4.843028809243734e-5 | 0.63707284 | TAM II late versus naïve |
| *Arhgdib* | 0.0010347183901515404 | 0.63559474 | TAM II late versus naïve |
| *Kif5b* | 2.707554884035864e-4 | 0.63449178 | TAM II late versus naïve |
| *Col9a1* | 1.1426281813092756e-7 | 0.63430204 | TAM II late versus naïve |
| *Gm4332* | 5.781663417660179e-4 | 0.63375732 | TAM II late versus naïve |
| *Cxcl1* | 1.597555600534229e-8 | 0.63342819 | TAM II late versus naïve |
| *Metrnl* | 3.8329516198211444e-10 | 0.63279084 | TAM II late versus naïve |
| *Cndp2* | 2.1355612109878302e-5 | 0.63244866 | TAM II late versus naïve |
| *Mrc1* | 2.381073218034885e-6 | 0.63176687 | TAM II late versus naïve |
| *Ehd1* | 4.890247398319568e-13 | 0.62776647 | TAM II late versus naïve |
| *Lmnb1* | 3.5029641521521567e-10 | 0.62769851 | TAM II late versus naïve |
| *Psmb6* | 3.857171416352491e-4 | 0.62544493 | TAM II late versus naïve |
| *Smarca5* | 4.901777716806424e-5 | 0.62506203 | TAM II late versus naïve |
| *Cnbp* | 8.882914574479269e-4 | 0.62319859 | TAM II late versus naïve |
| *Rpl36a-ps2* | 6.991885590537244e-4 | 0.62110654 | TAM II late versus naïve |
| *Rpl37a* | 2.774435601735325e-6 | 0.62075611 | TAM II late versus naïve |
| *Ctsz* | 0.009627223680636572 | 0.61977815 | TAM II late versus naïve |
| *Gm10080* | 2.2508185790597552e-5 | 0.61194877 | TAM II late versus naïve |
| *Etf1* | 3.279206452083373e-5 | 0.61157815 | TAM II late versus naïve |
| *Selenok* | 4.7832856292315035e-4 | 0.61111672 | TAM II late versus naïve |
| *Lsp1* | 1.9473591196241963e-5 | 0.60732098 | TAM II late versus naïve |
| *Itgb1* | 0.0022941571226653733 | 0.60720044 | TAM II late versus naïve |
| *Spop* | 8.224304824761184e-4 | 0.60463009 | TAM II late versus naïve |
| *Ndufc1* | 9.77542772057931e-7 | 0.60399055 | TAM II late versus naïve |
| *Gnai2* | 0.004630368398851194 | 0.60175589 | TAM II late versus naïve |
| *Gm10250* | 0.0016071117508185862 | 0.59867646 | TAM II late versus naïve |
| *Gm9385* | 0.005842441077477294 | 0.59813122 | TAM II late versus naïve |
| *Rplp2* | 0.0021095681631007656 | 0.59775616 | TAM II late versus naïve |
| *Psma4* | 3.917535775683493e-5 | 0.59552443 | TAM II late versus naïve |
| *Actr2* | 0.002049573464368909 | 0.59343556 | TAM II late versus naïve |
| *Slfn4* | 1.5970508677212344e-8 | 0.59101239 | TAM II late versus naïve |
| *Ndufa13* | 9.333635508696109e-4 | 0.58446115 | TAM II late versus naïve |
| *Cox5b* | 3.2660375037380266e-4 | 0.58413367 | TAM II late versus naïve |
| *Bcl2a1d* | 2.8315915844972437e-10 | 0.58345865 | TAM II late versus naïve |
| *Park7* | 1.24168460746427e-4 | 0.58340558 | TAM II late versus naïve |
| *Samhd1* | 4.725495807437739e-4 | 0.58102951 | TAM II late versus naïve |
| *Bnip2* | 3.609304102159409e-4 | 0.5801815 | TAM II late versus naïve |
| *Coro1a* | 5.904521904546538e-5 | 0.57653573 | TAM II late versus naïve |
| *Lrrfip1* | 2.300434945201897e-4 | 0.57535549 | TAM II late versus naïve |
| *Lst1* | 8.791474363330218e-4 | 0.57457805 | TAM II late versus naïve |
| *Uqcr10* | 1.4357802809324127e-5 | 0.57329009 | TAM II late versus naïve |
| *Slfn5* | 1.0908397607688967e-7 | 0.57107349 | TAM II late versus naïve |
| *Ms4a6b* | 0.0024894393936607056 | 0.57085279 | TAM II late versus naïve |
| *Nap1l1* | 7.40485224747866e-6 | 0.57011456 | TAM II late versus naïve |
| *H2-Q7* | 2.9736150988468792e-9 | 0.56824962 | TAM II late versus naïve |
| *Clic4* | 2.2256820007783092e-8 | 0.56824962 | TAM II late versus naïve |
| *Ms4a4c* | 1.6094112307406126e-7 | 0.56824962 | TAM II late versus naïve |
| *Dynll1* | 1.2186641009595543e-4 | 0.56763764 | TAM II late versus naïve |
| *Hif1a* | 0.003862059213503166 | 0.56699376 | TAM II late versus naïve |
| *Pcbp1* | 8.414392381989983e-4 | 0.56488753 | TAM II late versus naïve |
| *Gm43712* | 1.248460030361496e-4 | 0.56159632 | TAM II late versus naïve |
| *Ets2* | 1.8554547223424466e-8 | 0.56095678 | TAM II late versus naïve |
| *Ccl2* | 1.6896806965287749e-4 | 0.56046466 | TAM II late versus naïve |
| *Rps10-ps2* | 6.601939065651417e-5 | 0.56021217 | TAM II late versus naïve |
| *Tceb2* | 0.004233168234275445 | 0.55947994 | TAM II late versus naïve |
| *Psmb3* | 2.7661482463381404e-4 | 0.55939251 | TAM II late versus naïve |
| *Gsn* | 1.4543010082814146e-7 | 0.55932899 | TAM II late versus naïve |
| *Cd93* | 1.142272521007938e-7 | 0.55806827 | TAM II late versus naïve |
| *Lect1* | 1.144407919075453e-7 | 0.55737897 | TAM II late versus naïve |
| *Dynlrb1* | 2.9356783004415396e-4 | 0.55715363 | TAM II late versus naïve |
| *Arpc1a* | 4.631367978096667e-6 | 0.55657526 | TAM II late versus naïve |
| *Cct6a* | 0.0026619373456625212 | 0.55550067 | TAM II late versus naïve |
| *Rps3a1* | 0.00744639149215471 | 0.5507479 | TAM II late versus naïve |
| *Gdi2* | 0.005645315783088912 | 0.54885248 | TAM II late versus naïve |
| *Ezr* | 2.7311142146686144e-9 | 0.54857493 | TAM II late versus naïve |
| *Rps15a-ps5* | 4.79674298276503e-4 | 0.54834629 | TAM II late versus naïve |
| *Atp6v0e* | 4.360713436215654e-4 | 0.54638294 | TAM II late versus naïve |
| *Slc25a4* | 0.0017171956588789066 | 0.54512222 | TAM II late versus naïve |
| *Slamf7* | 2.814995236058831e-10 | 0.54499712 | TAM II late versus naïve |
| *Ier2* | 3.584169705871318e-4 | 0.54486594 | TAM II late versus naïve |
| *Ifi205* | 2.1562343355924496e-9 | 0.54412327 | TAM II late versus naïve |
| *Atp6ap2* | 6.126007894252958e-4 | 0.54280512 | TAM II late versus naïve |
| *Ndufa4* | 0.003851603103512437 | 0.54096271 | TAM II late versus naïve |
| *Tmem14c* | 5.149057025649478e-6 | 0.54038765 | TAM II late versus naïve |
| *Ifi203* | 1.552058349155256e-7 | 0.53990479 | TAM II late versus naïve |
| *Atp5j2* | 0.0026358765621653526 | 0.53958105 | TAM II late versus naïve |
| *Gna13* | 0.0012647714402796088 | 0.53936076 | TAM II late versus naïve |
| *Dusp2* | 3.2921956825194305e-6 | 0.53921217 | TAM II late versus naïve |
| *Sp100* | 2.0266957523039164e-8 | 0.53845823 | TAM II late versus naïve |
| *Chmp2a* | 1.7562992218354373e-4 | 0.53823961 | TAM II late versus naïve |
| *Csf2ra* | 3.876035373679179e-5 | 0.53814546 | TAM II late versus naïve |
| *Cd44* | 2.3170176391269124e-8 | 0.53770428 | TAM II late versus naïve |
| *Arf4* | 2.454992651345959e-4 | 0.53704379 | TAM II late versus naïve |
| *Lgals3bp* | 8.454443110917787e-4 | 0.5365402 | TAM II late versus naïve |
| *Rhoa* | 0.0015779093906598844 | 0.53562225 | TAM II late versus naïve |
| *Naca* | 0.005261986946219744 | 0.53559723 | TAM II late versus naïve |
| *Nfil3* | 2.152082727662021e-9 | 0.53488041 | TAM II late versus naïve |
| *Slpi* | 7.970823651396056e-7 | 0.53488041 | TAM II late versus naïve |
| *Sdhd* | 8.792493737787167e-5 | 0.53247475 | TAM II late versus naïve |
| *Abracl* | 1.1026144901777471e-4 | 0.53247144 | TAM II late versus naïve |
| *Arhgap30* | 9.372248830484963e-4 | 0.53068627 | TAM II late versus naïve |
| *Atp5o* | 4.4454096824128006e-4 | 0.52982575 | TAM II late versus naïve |
| *Klf13* | 1.997098461933727e-5 | 0.52938457 | TAM II late versus naïve |
| *Cyp4f18* | 3.513992597118891e-11 | 0.52903413 | TAM II late versus naïve |
| *Atp5d* | 0.0013252765322015819 | 0.52624793 | TAM II late versus naïve |
| *Mia2* | 0.002196982336606803 | 0.52542073 | TAM II late versus naïve |
| *Sdhb* | 1.2705644745947126e-4 | 0.52540324 | TAM II late versus naïve |
| *Tgif1* | 7.395152192339508e-7 | 0.52429555 | TAM II late versus naïve |
| *Trim30a* | 0.0027413218832222667 | 0.52354751 | TAM II late versus naïve |
| *Uqcrh* | 0.0011995323546719683 | 0.52303638 | TAM II late versus naïve |
| *Capg* | 9.480865721717208e-7 | 0.52222893 | TAM II late versus naïve |
| *Ik* | 0.006678890486532914 | 0.52209576 | TAM II late versus naïve |
| *Hnrnpf* | 7.49625167458163e-4 | 0.52192869 | TAM II late versus naïve |
| *Emp1* | 1.5955375111543023e-8 | 0.52162471 | TAM II late versus naïve |
| *Atp5k* | 5.990452293481098e-4 | 0.52157163 | TAM II late versus naïve |
| *Smdt1* | 3.4425041960745197e-4 | 0.52146359 | TAM II late versus naïve |
| *Taok3* | 8.335437911843115e-5 | 0.52093428 | TAM II late versus naïve |
| *Rpl36al* | 0.0028782944136765626 | 0.51971235 | TAM II late versus naïve |
| *Tmem167* | 1.833196189961376e-5 | 0.51962492 | TAM II late versus naïve |
| *Trim25* | 2.152082727662021e-9 | 0.51891743 | TAM II late versus naïve |
| *Tnfsf9* | 7.978165239690587e-7 | 0.51891743 | TAM II late versus naïve |
| *Dazap2* | 2.724259445106145e-4 | 0.51888714 | TAM II late versus naïve |
| *Cct2* | 1.0322237835917406e-4 | 0.51761946 | TAM II late versus naïve |
| *Anxa4* | 2.3021983552284755e-8 | 0.51740623 | TAM II late versus naïve |
| *Myof* | 3.0187824142660825e-8 | 0.51713992 | TAM II late versus naïve |
| *Hspa9* | 0.0020307093382054254 | 0.51686343 | TAM II late versus naïve |
| *Srsf3* | 0.0029996274741329325 | 0.51642837 | TAM II late versus naïve |
| *Pdap1* | 0.0017128551263581892 | 0.51139926 | TAM II late versus naïve |
| *Rps24-ps2* | 2.3739549730623714e-5 | 0.51046714 | TAM II late versus naïve |
| *Set* | 2.934051744443254e-4 | 0.51006363 | TAM II late versus naïve |
| *Eif3k* | 9.943400338560503e-5 | 0.50999915 | TAM II late versus naïve |
| *Cope* | 0.0011243777248734672 | 0.5077307 | TAM II late versus naïve |
| *Eif5a* | 0.002700546853260453 | 0.50765466 | TAM II late versus naïve |
| *Ubxn1* | 0.008311478412114888 | 0.50756185 | TAM II late versus naïve |
| *Emb* | 1.6576876705405508e-7 | 0.50577832 | TAM II late versus naïve |
| *Psma1* | 2.9884197195233686e-4 | 0.50572856 | TAM II late versus naïve |
| *Mkrn1* | 3.684010839816837e-4 | 0.50572524 | TAM II late versus naïve |
| *Rac2* | 9.856958257701636e-4 | 0.5051279 | TAM II late versus naïve |
