# Supplementary material for: Elucidating tumour‐associated microglia/macrophage diversity along glioblastoma progression and under ACOD1 deficiency
Source: Mol Oncol. 2022 Aug 15;16(17):3167–91. doi: 10.1002/1878-0261.13287 (PMC9441003; doi:10.1002/1878-0261.13287)
Supplement: Supplementary file 6 — Table S5. Up‐regulated differentially expressed genes at late stage for TAM I KO and TAM II KO versus correspondent WT cells (p‐value < 0,001 and log2 FC > 0,5), related to Figure 5. [file MOL2-16-3167-s007.docx]

**Table S5. Up-regulated differentially expressed genes at late stage for TAM I KO and TAM II KO versus correspondent WT cells (p-value < 0.001 and log2 FC > 0.5), related to figure 5.**

| Gene symbol | p-value | logFC | Comparison |
| --- | --- | --- | --- |
| *Cd74* | 1.0201486727028029e-6 | 2.949 | TAM I KO versus TAM I WT |
| *H2-Ab1* | 3.532129264514336e-7 | 2.683 | TAM I KO versus TAM I WT |
| *H2-Eb1* | 3.154077095205742e-8 | 2.607 | TAM I KO versus TAM I WT |
| *H2-Aa* | 6.600882063676006e-7 | 2.442 | TAM I KO versus TAM I WT |
| *Gbp2* | 3.1023319225140386e-7 | 2.208 | TAM I KO versus TAM I WT |
| *Ly6a* | 0.004420776720357333 | 1.180 | TAM I KO versus TAM I WT |
| *Stat1* | 2.2090242247987807e-4 | 1.080 | TAM I KO versus TAM I WT |
| *Cxcl9* | 0.0027958632728675163 | 1.029 | TAM I KO versus TAM I WT |
| *Psmb8* | 0.006252801808472668 | 0.957 | TAM I KO versus TAM I WT |
| *Arhgap5* | 4.97346350871414e-4 | 0.937 | TAM I KO versus TAM I WT |
| *H2-Q7* | 0.002387753621307706 | 0.897 | TAM I KO versus TAM I WT |
| *Gbp8* | 5.999704919939999e-4 | 0.779 | TAM I KO versus TAM I WT |
| *H2-DMa* | 0.007564346749807322 | 0.778 | TAM I KO versus TAM I WT |
| *Psme1* | 0.007822400891671402 | 0.772 | TAM I KO versus TAM I WT |
| *Tap1* | 0.0021332582314801123 | 0.723 | TAM I KO versus TAM I WT |
| *Cd36* | 0.004729624705151469 | 0.641 | TAM I KO versus TAM I WT |
| *Tap2* | 0.0075642031521810545 | 0.627 | TAM I KO versus TAM I WT |
| *Fgl2* | 0.006801564978817804 | 0.612 | TAM I KO versus TAM I WT |
| *Il2rg* | 0.004947838855195913 | 0.585 | TAM I KO versus TAM I WT |
| *Casp1* | 0.0027236651074679053 | 0.574 | TAM I KO versus TAM I WT |
| *Clec7a* | 0.008946024218724858 | 0.545 | TAM I KO versus TAM I WT |
| *Myh10* | 5.620916678800994e-4 | 0.527 | TAM I KO versus TAM I WT |
| *Tcf12* | 0.003126962295352084 | 0.516 | TAM I KO versus TAM I WT |
| *Gm4951* | 0.0027779594635931933 | 0.508 | TAM I KO versus TAM I WT |
| *AW112010* | 1.51e-13 | 7.530 | TAM II KO versus TAM II WT |
| *Ccl8* | 0.000632 | 7.450 | TAM II KO versus TAM II WT |
| *Gbp2* | 3.93e-11 | 6.790 | TAM II KO versus TAM II WT |
| *Iigp1* | 1.73e-05 | 5.560 | TAM II KO versus TAM II WT |
| *Gbp8* | 7.05e-06 | 5.170 | TAM II KO versus TAM II WT |
| *Cxcl10* | 0.000595 | 4.980 | TAM II KO versus TAM II WT |
| *Irf1* | 0.000163 | 4.940 | TAM II KO versus TAM II WT |
| *Casp1* | 0.000209 | 4.400 | TAM II KO versus TAM II WT |
| *Gm9835* | 0.00036 | 4.090 | TAM II KO versus TAM II WT |
| *Ly6a* | 6.84e-11 | 3.900 | TAM II KO versus TAM II WT |
| *Ly6i* | 0.00119 | 3.770 | TAM II KO versus TAM II WT |
| *Atp11b* | 0.00297 | 3.770 | TAM II KO versus TAM II WT |
| *Fam26f* | 0.000407 | 3.750 | TAM II KO versus TAM II WT |
| *Stat1* | 3.12e-10 | 3.700 | TAM II KO versus TAM II WT |
| *Fgl2* | 4.12e-08 | 3.420 | TAM II KO versus TAM II WT |
| *Klra2* | 0.00125 | 3.320 | TAM II KO versus TAM II WT |
| *Ccdc109b* | 0.00724 | 3.280 | TAM II KO versus TAM II WT |
| *Gbp5* | 0.00131 | 3.230 | TAM II KO versus TAM II WT |
| *C3* | 0.00189 | 3.210 | TAM II KO versus TAM II WT |
| *Ifi47* | 0.000754 | 3.160 | TAM II KO versus TAM II WT |
| *Igtp* | 0.00194 | 2.940 | TAM II KO versus TAM II WT |
| *Fcgr4* | 1.95e-05 | 2.820 | TAM II KO versus TAM II WT |
| *Il18bp* | 0.00121 | 2.710 | TAM II KO versus TAM II WT |
| *Taf10* | 0.00957 | 2.540 | TAM II KO versus TAM II WT |
| *Tmem173* | 0.00197 | 2.370 | TAM II KO versus TAM II WT |
| *Uso1* | 0.00688 | 2.350 | TAM II KO versus TAM II WT |
| *Gbp7* | 1.16e-05 | 2.260 | TAM II KO versus TAM II WT |
| *Il2rg* | 3.14e-05 | 2.250 | TAM II KO versus TAM II WT |
| *Isoc1* | 0.00572 | 2.230 | TAM II KO versus TAM II WT |
| *Gatm* | 0.00144 | 2.220 | TAM II KO versus TAM II WT |
| *Tap1* | 0.000747 | 2.210 | TAM II KO versus TAM II WT |
| *Leprot* | 0.000826 | 2.210 | TAM II KO versus TAM II WT |
| *Ifi209* | 0.001 | 2.210 | TAM II KO versus TAM II WT |
| *Pirb* | 0.00229 | 2.210 | TAM II KO versus TAM II WT |
| *Clec12a* | 0.000263 | 2.110 | TAM II KO versus TAM II WT |
| *H2-Q6* | 0.00657 | 2.110 | TAM II KO versus TAM II WT |
| *Pla2g16* | 0.00213 | 2.070 | TAM II KO versus TAM II WT |
| *Ifi211* | 0.00407 | 1.990 | TAM II KO versus TAM II WT |
| *Gm1966* | 0.00166 | 1.980 | TAM II KO versus TAM II WT |
| *Snx10* | 0.000286 | 1.970 | TAM II KO versus TAM II WT |
| *Psmb9* | 0.000136 | 1.940 | TAM II KO versus TAM II WT |
| *Gbp3* | 0.00032 | 1.940 | TAM II KO versus TAM II WT |
| *Lair1* | 0.00275 | 1.940 | TAM II KO versus TAM II WT |
| *Parp9* | 0.00698 | 1.900 | TAM II KO versus TAM II WT |
| *Ifih1* | 0.00923 | 1.890 | TAM II KO versus TAM II WT |
| *Cd72* | 0.00762 | 1.880 | TAM II KO versus TAM II WT |
| *Gng2* | 0.00928 | 1.850 | TAM II KO versus TAM II WT |
| *Zbp1* | 0.00347 | 1.830 | TAM II KO versus TAM II WT |
| *Tapbp* | 0.00361 | 1.810 | TAM II KO versus TAM II WT |
| *Irgm1* | 0.00511 | 1.780 | TAM II KO versus TAM II WT |
| *AU020206* | 0.00582 | 1.780 | TAM II KO versus TAM II WT |
| *Psmb10* | 0.000256 | 1.720 | TAM II KO versus TAM II WT |
| *Irf2* | 0.00828 | 1.660 | TAM II KO versus TAM II WT |
| *Slc16a3* | 0.00883 | 1.640 | TAM II KO versus TAM II WT |
| *Trafd1* | 0.000957 | 1.630 | TAM II KO versus TAM II WT |
| *Ccdc50* | 0.00583 | 1.630 | TAM II KO versus TAM II WT |
| *H2-Q7* | 0.00422 | 1.600 | TAM II KO versus TAM II WT |
| *Mafb* | 0.00532 | 1.560 | TAM II KO versus TAM II WT |
| *Samhd1* | 0.000461 | 1.520 | TAM II KO versus TAM II WT |
| *H2-DMb1* | 0.00012 | 1.510 | TAM II KO versus TAM II WT |
| *H2-Aa* | 2.52e-06 | 1.470 | TAM II KO versus TAM II WT |
| *Tomm22* | 0.00721 | 1.460 | TAM II KO versus TAM II WT |
| *Myl12b* | 0.000764 | 1.450 | TAM II KO versus TAM II WT |
| *H2-T23* | 0.00463 | 1.400 | TAM II KO versus TAM II WT |
| *H2-Ab1* | 1.37e-06 | 1.370 | TAM II KO versus TAM II WT |
| *Oasl2* | 0.00571 | 1.360 | TAM II KO versus TAM II WT |
| *Fos* | 0.00365 | 1.280 | TAM II KO versus TAM II WT |
| *H2-Eb1* | 5.28e-06 | 1.270 | TAM II KO versus TAM II WT |
| *H2-K1* | 3.52e-05 | 1.260 | TAM II KO versus TAM II WT |
| *Pnrc1* | 0.00667 | 1.260 | TAM II KO versus TAM II WT |
| *Cd74* | 1.09e-05 | 1.220 | TAM II KO versus TAM II WT |
| *Tgfbi* | 0.00801 | 1.190 | TAM II KO versus TAM II WT |
| *H2-DMa* | 0.00134 | 1.160 | TAM II KO versus TAM II WT |
| *Rsrp1* | 0.00778 | 1.040 | TAM II KO versus TAM II WT |
| *Ccnd1* | 0.00486 | 1.000 | TAM II KO versus TAM II WT |
| *B2m* | 0.000121 | 0.982 | TAM II KO versus TAM II WT |
| *Aif1* | 0.000402 | 0.930 | TAM II KO versus TAM II WT |
| *Ptprc* | 0.00143 | 0.926 | TAM II KO versus TAM II WT |
| *Npc2* | 0.00174 | 0.879 | TAM II KO versus TAM II WT |
| *Cd52* | 0.00561 | 0.808 | TAM II KO versus TAM II WT |
| *Psmb8* | 0.00125 | 0.791 | TAM II KO versus TAM II WT |
| *Ssb* | 0.00508 | 0.740 | TAM II KO versus TAM II WT |
| *Irf8* | 0.00536 | 0.659 | TAM II KO versus TAM II WT |
