# Supplementary material for: Elucidating tumour‐associated microglia/macrophage diversity along glioblastoma progression and under ACOD1 deficiency
Source: Mol Oncol. 2022 Aug 15;16(17):3167–91. doi: 10.1002/1878-0261.13287 (PMC9441003; doi:10.1002/1878-0261.13287)
Supplement: Supplementary file 7 — Table S6. Up‐regulated differentially expressed genes at early stage comparing lymphocytes from KO and WT mice (p‐value < 0.01 and Log2 FC > 0.5), related to Supplementary Figure 9. [file MOL2-16-3167-s006.docx]

**Table S6. Up-regulated differentially expressed genes at early stage comparing lymphocytes from KO and WT mice (p-value < 0.01 and Log2 FC > 0.5), related to Supplementary figure 9.**

| **Gene symbol** | **p-value** | **logFC** |
| --- | --- | --- |
| *Dbi* | 1.6698987338919431e-6 | 0.990808404 |
| *Pfn1* | 8.41983827695326e-5 | 0.930855464 |
| *Lgals1* | 0.00380073049840353 | 0.854906338 |
| *Ifitm3* | 7.786832170997523e-4 | 0.722399821 |
| *Mt1* | 5.0711624191895e-6 | 0.654819671 |
| *Anxa2* | 0.00185970039681286 | 0.641724602 |
| *Myl12a* | 0.01369836882907546 | 0.633778143 |
| *Ldha* | 0.00493283154761393 | 0.61690132 |
| *Capg* | 6.776468283630539e-4 | 0.598599081 |
| *Arhgdib* | 0.00606238374247211 | 0.581080714 |
| *Ifng* | 0.02281081705230598 | 0.570763384 |
| *Dynll1* | 5.253161603696783e-4 | 0.562763533 |
| *Rgcc* | 1.1406215912483843e-4 | 0.555229654 |
| *Sh3bgrl3* | 0.01734825304815392 | 0.552410846 |
| *Sec61b* | 0.01054761857054867 | 0.527051216 |
| *Ly6a* | 0.03368308543626072 | 0.516968854 |
| *Tagln2* | 0.00844092046359648 | 0.505294359 |
